# Interplay of miR-137 and EZH2 contributes to the genome-wide redistribution of H3K27me3 underlying the Pb-induced memory impairment

**DOI:** 10.1038/s41419-019-1912-7

**Published:** 2019-09-11

**Authors:** Xiaozhen Gu, Yi Xu, Wei-Zhen Xue, Yulan Wu, Zi Ye, Guiran Xiao, Hui-Li Wang

**Affiliations:** 1 School of Food and Bioengineering, Hefei University of Technology, Hefei, Anhui 230009 People’s Republic of China; 2 College of Life Science and Bio-engineering, Beijing University of Technology, Beijing, 100022 People’s Republic of China

**Keywords:** Epigenetic memory, Epigenetic memory, Molecular neuroscience, Molecular neuroscience, Neurological disorders

## Abstract

Compromised learning and memory is a common feature of multiple neurodegenerative disorders. A paradigm spatial memory impairment could be caused by developmental lead (Pb) exposure. Growing evidence implicates epigenetic modifications in the Pb-mediated memory deficits; however, how histone modifications exemplified by H3K27me3 (H3 Lys27 trimethylation) contribute to this pathogenesis remains poorly understood. Here we found that Pb exposure diminished H3K27me3 levels in vivo by suppressing EZH2 (enhancer of zeste homolog 2) expression at an early stage. EZH2 overexpression in Pb-treated rats rescued the H3K27me3 abundance and partially restored the normal spatial memory, as manifested by the rat performance in a Morris water maze test, and structural analysis of hippocampal spine densities. Furthermore, miR-137 and EZH2 constitute mutually inhibitory loop to regulate the H3K27me3 level, and this feedback regulation could be specifically activated by Pb treatment. Considering genes targeted by H3K27me3, ChIP-chip (chromatin immunoprecipitation on chip) studies revealed that Pb could remodel the genome-wide distribution of H3K27me3, represented by pathways like transcriptional regulation, developmental regulation, cell motion, and apoptosis, as well as a novel *Wnt9b* locus. As a Wnt isoform associated with canonical and noncanonical signaling, Wnt9b was regulated by the opposite modifications of H3K4me3 (H3 Lys4 trimethylation) and H3K27me3 in Pb-exposed neurons. Rescue trials further validated the contribution of Wnt9b to Pb-induced neuronal impairments, wherein canonical or noncanonical Wnt signaling potentially exhibited destructive or protective roles, respectively. In summary, the study reveals an epigenetic-based molecular change underlying Pb-triggered spatial memory deficits, and provides new potential avenues for our understanding of neurodegenerative diseases with environmental etiology.

## Introduction

Learning and memory deficits are the important symptoms contributing to the development of an array of neurodegenerative diseases, such as Alzheimer’s disease^1^ and non-motor aspects of Parkinson’s disease^2^. The hippocampal region stores information about allocentric spaces^3,4^; thus, damage to hippocampal development frequently leads to memory dysfunction. Lead (Pb) prevails as a causative agent of hippocampal neuronal death and cognitive dysfunction, leading to the typical neurotoxicity characterized by region-specific, long-lasting, altered spine morphology, among others^5–7^. Given that Pb exposure results in typical spatial memory deficit, efforts were made to investigate the molecular pathways involved in this specific pathogenesis^8–10^.

Epigenetic modifications regulate gene expression by altering accessibility of genomic loci to the transcription machinery^11,12^. Epigenetic dysregulation underlies a number of neurological disorders accompanied by cognitive dysfunction^13^. For instance, disruption of histone acetylation leads to cognitive abnormalities^14^, and inhibition of HDAC (histone deacetylase) activity can serve as a powerful tool to treat the neurodegeneration-related cognitive decline^15^. In terms of histone methylations, Snigdha et al.^16^ discovered that H3K9me3 inhibition improved memory and promotes spine formation in the aged hippocampus. Despite this finding, compared to acetylation, roles of histone methylation are less understood.

Among the various modifications, histone methylations are relatively stable, and therefore viewed as potential epigenetic markers for transgenerational transmission^17,18^. H3K27me3 (H3 Lys27 trimethylation) is deposited by Polycomb group (PcG) proteins. In mammals, PRC2 (PcG complex 2) consists of three core PcG components: an enhancer of zeste homolog 2 (EZH2) or its close homolog EZH1, an embryonic ectoderm development (EED), and a suppressor of zeste 12^19–22^. H3K27me3 is regulated by the opposite activities of methyltransferases (EZH1 and EZH2) and demethylases (UTX and JMJD3)^19,23^. In terms of neuronal development, H3K27me3 controls temporal patterns of gene expression through lineage commitment to the terminal differentiated state^19,24^. In spite of its stable nature, the expression of H3K27me3 can be altered by environmental insults in some instances^25^.

Compelling evidence considered Pb as an epigenetic modifier^26,27^. A study with primate animals shows that infant exposure to Pb can elevate H3K4me (H3 Lys4 trimethylation) levels in the aging brain^28^. Although the adverse effect of Pb on cognitive memory has long been appreciated, whether H3K27me3 is involved in this process remains elusive.

Here, we investigated the epigenetic alterations underlying spatial memory deficits. We showed that genomic landscape of H3K27me3 occupancy is reprogrammed upon Pb treatment, and this reprogramming is due to the activated interplay of miR-137 and EZH2. The study provides new insight into the epigenetic changes involved in the Pb-induced memory deficits.

## Results

### Pb reduces H3K27me3 levels via suppressing EZH2 expression at early developmental stage

**Fig. 1:**
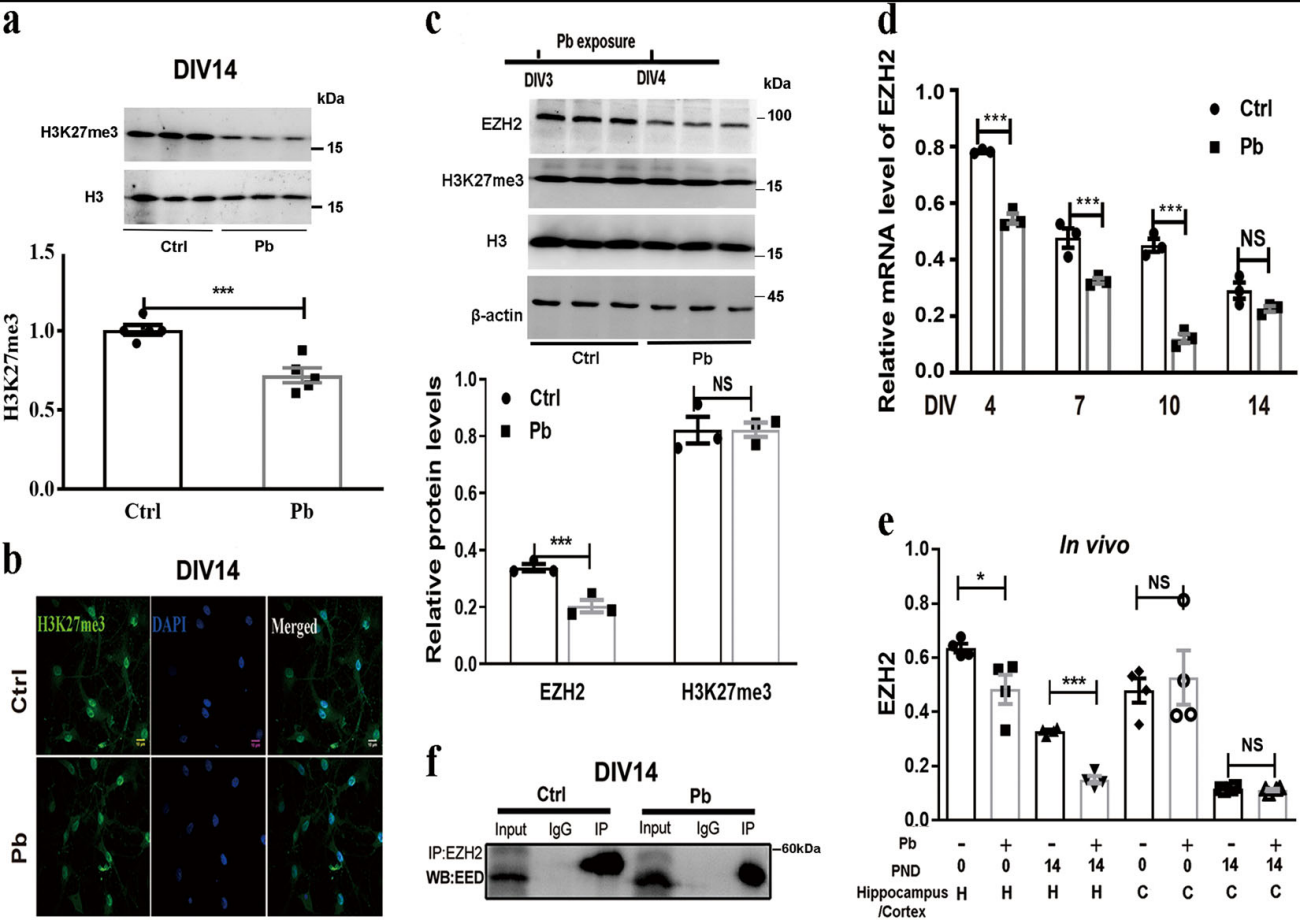
Pb reduces H3K27me3 levels via suppressing the expression of EZH2 at early culture stage. **a**, **b** Immunoblot, protein quantification, and immunostaining images of H3K27me3 in response to Pb (5 μM) in the primary hippocampal neurons at DIV14 (days in vitro 14) (*n* = 5, independent two-sample *t* test, two-tailed). H3K27me3 and nuclei were stained green and blue, respectively (scale bar: 10 μm, ~150 cells per experimental condition). **c** Immunoblots and quantification of protein levels of EZH2, H3K27me3 upon Pb treatment at DIV4 (*n* = 3, independent two-sample *t* test, two-tailed). **d** Relative mRNA levels of *EZH2* upon Pb treatment at various culture stages (*n* = 3, independent two-sample *t* test, two-tailed). **e** The alterations of EZH2 in response to Pb in vivo. SD rats were maternally and postnatally exposed to Pb till PND (postnatal day) 0 or PND14. They were then sacrificed and the hippocampal and cerebral cortex tissues were collected for the immunoblot analysis (*n* = 4, independent two-sample *t* test, two-tailed; column 1 vs. column 2, *P = 0.0355). **f** Co-immunoprecipitation (co-IP) of EZH2 in Pb-treated and untreated primary hippocampal neurons (*n* = 3, representative bands sampled from triplicate independent experiments). EED was determined by immunoblots with EZH2-IP samples. IgG represents a control antibody used for IP. Prior to IP experiments, one-tenth of total lysates were subjected to the immunoblots as input controls. The data are represented as mean ± SEM; ***P < 0.001, *P < 0.05, NS *P* > 0.05. Symbols show individual samples. The different types of symbols represent the different groups. In **a** and **b**, the solid circle and solid square represent control and Pb, respectively. In **e**, the solid circle, solid square, solid triangle, and inverted solid triangle represent control in PND 0, Pb in PND 0, control in PND14, and Pb in PDN14 of the hippocampus, respectively, and the solid diamond and open circle represent the control in PND 0 and Pb in PND 0 of the cortex, respectively

Since H3K27me3 is a stable epigenetic marker^21^, we first investigated if its level could be altered by Pb exposure. Primary hippocampal neurons were exposed to 5 μM Pb from days in vitro 3 (DIV3) and collected at DIV14 for protein extraction. Western blot analysis showed that Pb treatment significantly reduced the level of H3K27me3 (Fig. 1a). This result was further consolidated by immunostaining images of H3K27me3 at DIV14, which revealed the loss of the markers upon Pb exposure compared to the mock treatment (Fig. 1b).

To identify the potential causes of the decreased H3K27me3, the expression of the candidate histone methyltransferases or demethylases was examined. At DIV14, the expression of tested genes, namely *EZH1*, *UTX*, and *JMJD*3, was not changed by Pb. Besides, at this developmental stage, further alterations of *EZH2* by Pb exposure were not observed (Fig. S1).

The earlier stage along the neuronal developmental timeline was then subjected to further inspection. At DIV4, Western blot and quantitative reverse transcription PCR (RT-qPCR) assays showed that both protein and messenger RNA (mRNA) levels of EZH2 were markedly down-regulated within 24 h after Pb exposure (Fig. 1c, d), whereas H3K27me3 mark was not altered at the same stage (Fig. 1c). Further analysis of rats treated with or without Pb in vivo showed that the EZH2 accumulation was significantly decreased in the hippocampus, but not in the cerebral cortex at postnatal day 14 (PND14) (Fig. 1e). Thus, Pb treatment specifically affected EZH2 abundance in the hippocampus.

We also evaluated the existing profiles of PRC2 in primary neurons. EED is an essential subunit of PRC2 and required in EZH2 functioning^21,29^. Co-immunoprecipitation (co-IP) trials showed Pb exposure compromised the incorporation of EED into the EZH2/EED assembly (Fig. 1f). Together with the finding that EED level remains constant with Pb treatment (data not shown), it indicated that Pb exposure resulted in a diminished presence of PRC2 in primary hippocampal neurons. These results suggested that Pb exposure triggered the early reduction of EZH2, which might in turn cause the subsequent decrease of the H3K27me3 level.

### H3K27me3 mediates the Pb-led spatial memory deficits

**Fig. 2:**
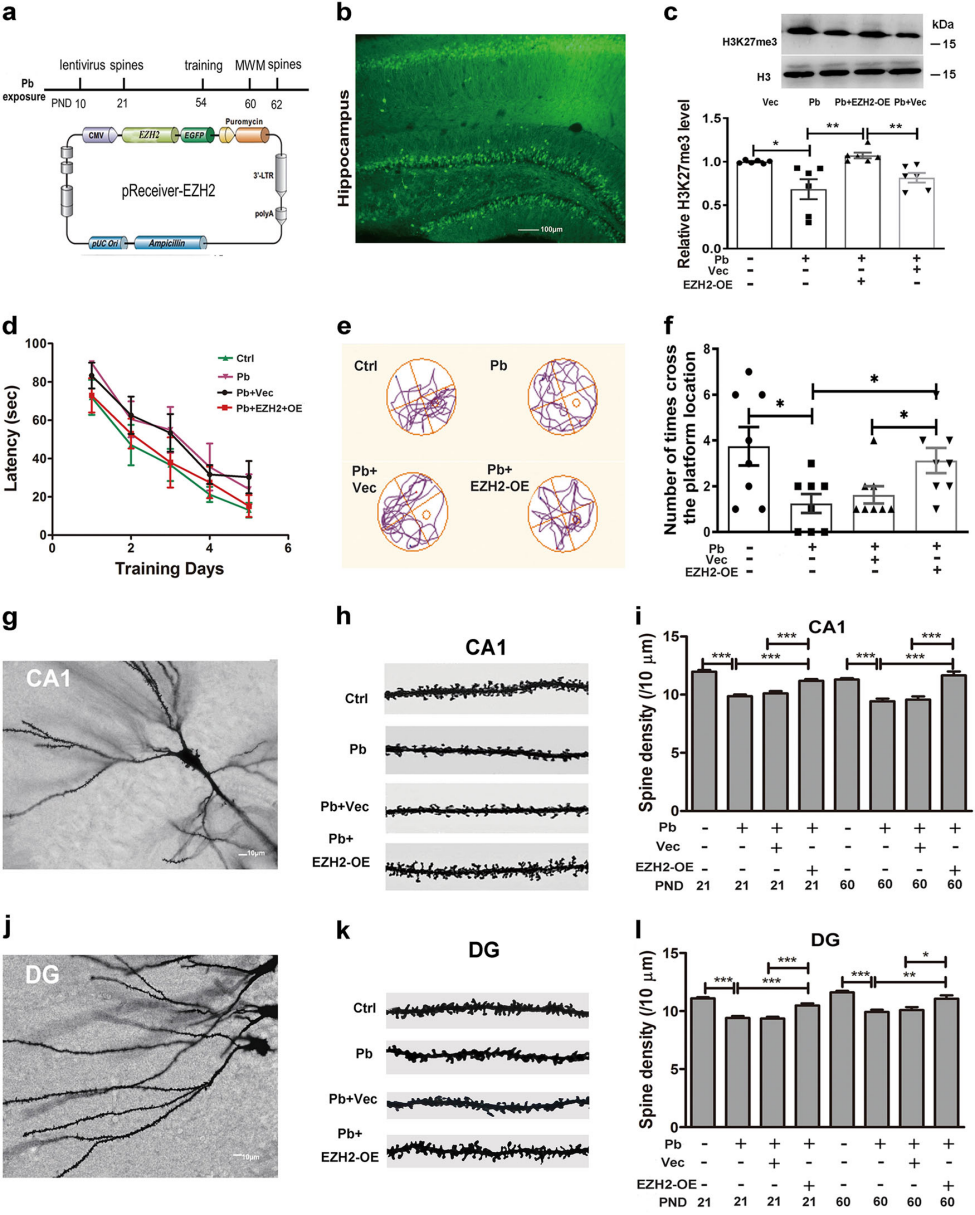
H3K27me3 mediates the spatial memory deficits induced by Pb exposure. **a** Schematic illustration of experimental design and construction of lentivectors. Pb-exposed rats were injected with repackaged lentivirus at PND10. Animals were trained for 5 days (four trials per day) to find the hidden platform, and followed by the probe trial 24 h later, in which the platform is not present. Spine examinations were conducted at PND21 and PND60, respectively. **b** Representative images of hippocampus following the injection of pReceiver-EZH2-containing virus (*n* = 8, scale bar: 100 μm). **c** Immunoblots and quantification of protein levels of H3K27me3 upon the overexpression of EZH2 at PND21 (*n* = 6, Mann–Whitney *U* test; column 1 vs. column 2; independent two-sample *t* test, two-tailed; column 2 vs. column 3 **P = 0.0088; column 3 vs. column 4 *P = 0.0025). **d**–**f** The Morris water maze (MWM) tests assessing the capacities to be trained to find the hidden platform (*n* = 8, independent two-sample *t* test, two-tailed; column 1 vs. column 2 *P = 0.0182; column 2 vs. column 4 *P = 0.0162; column 3 vs. column 4 *P = 0.0460). The latency **d** was recorded and analyzed (two-way ANOVA) during the training days. For assessment, based on their respective moving tracks (**e**), the number of times crossing the platform (**f**) were counted at PND60. **g**–**l** Morphological analysis of dendritic spines of hippocampal neurons (~150 dendritic spines per experimental condition). **g** Representative images of neuronal dendritic arborization in the region of CA1. Representative images (**h**) and quantification of spine densities were shown in the region of CA1 (i, independent two-sample *t* test, two-tailed for column 2 vs. column 4, column 7 vs. column 8; Mann–Whitney *U* test for other comparisons). **j** Representative images of neuronal dendritic arborization in the region of DG. Representative images (**k**) and quantification of spine densities were shown in the region DG (**l**, independent two-sample *t* test, two-tailed for column 1 vs. column 2, column 7 vs. column 8 *P = 0.0122; Mann–Whitney *U* test for other comparisons). Vec and EZH2-OE refer to the rats infected with lentivirus harboring the empty and pReceiver-EZH2 vector, respectively. The data are represented as mean ± SEM; ***P < 0.001, **P < 0.01, and *P < 0.05. Symbols show individual samples. The different types of symbols represent the different groups. In **c**, the solid circle, solid square, solid triangle, and inverted solid triangle represent control, Pb, Pb+EZH2-OE, and Pb+Vec, respectively. In **f**, the solid circle, solid square, solid triangle, and inverted solid triangle represent control, Pb, Pb+Vec, and Pb+EZH2-OE, respectively

H3K27me3 is implicated in neuronal development and cellular identity maintenance^24^. Given that Pb treatment changed H3K27me3 level, we next investigated whether Pb-triggered memory deficit is attributed to the deficiency in H3K27me3. To this end, we constructed a lentivirus vector to overexpress EZH2 with EGFP tagged at the C-terminal, and then the virus particles were bilaterally stereotaxically injected into the hippocampus of SD (Sprague–Dawley) rats at PND10 (Fig. 2a). Ten days later, rats were euthanized, and their brains were sectioned and imaged (Fig. 2b). The green fluorescent protein (GFP) detection in the hippocampus proved that the virus carrying pReceiver-EZH2 successfully infected the target tissues. According to Western blot analysis (Fig. 2c), the H3K27me3 level was increased through the lentivirus infection, which also validated the EZH2-overexpressing efficiency of the vector used. This result also validated the essential roles of EZH2 in regulating H3K27 methylations in the studied physiological context.

To study cognitive impairment by Pb exposure, we assessed the recovery effect of H3K27me3 on the spatial memory of Pb-exposed rats with Morris water maze (MWM) test. During the training session, the Pb-exposed rats infected with EZH2-OE, compared to the ones infected with the empty vector, tended to regain a better performance in the latency (Fig. 2d). According to two-way analysis of variance (ANOVA) analysis, the treatment and days both have the significant influence on rat latency to find the platform, but the factors do not have interactions. On the test day, the representative moving tracks of rats were presented in Fig. 2e. According to the statistics, the number of times that EZH2-OE rats crossed the hidden platform was more than the Pb-exposed groups (Fig. 2f). No differences were found in the average velocity of rats from various groups on the training and trial days (Fig. S2a, b). Because the crossing of the hidden platform is the paradigmatic representation of spatial memory strength, the data suggested that the impaired spatial memories were repaired to a variable degree with the ectopic recovery of H3K27me3.

Spines are dynamic structures for the excitatory synapse formation and an anatomical substrate for synaptic plasticity underlying learning and memory^30,31^. Previous report showed that Pb exposure impaired dendrite growth and maturity^8^. Thereby, we examined the hippocampal spine densities at both developmental and adult stages. Dendrites and spines were graphed with Golgi–Cox staining (Fig. 2g–l). The overexpression of EZH2 and the resulting H3K27me3 in the Pb-exposed rats increased the spine densities of CA1 (cornu ammonis area 1) (Fig. 2g–i) and DG (dentate gyrus) region (Fig. 2j–l). Of note, immature and mature neurons exhibited the similar responses under the tested circumstance. These findings stressed the important roles of H3K27me3 in spatial memory deficits induced by Pb exposure.

### Pb triggers mutual repression regulation between miR-137 and EZH2

**Fig. 3:**
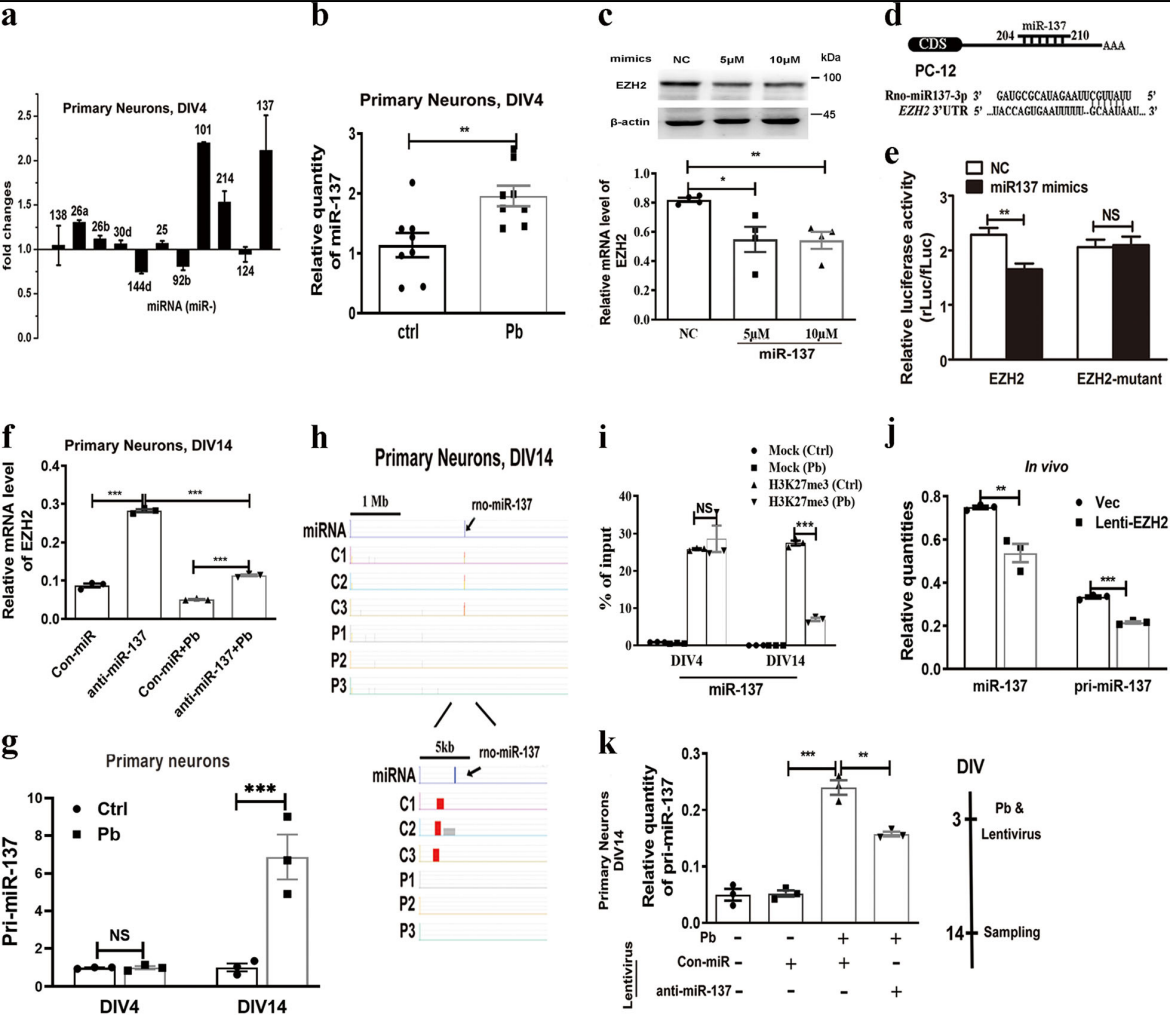
Pb exposure leads to an altered interaction of miR-137 and EZH2. **a**–**f** Up-regulation of miR-137 accelerates attenuation of EZH2 expression. **a** miRNA profiling in the primary hippocampal neurons upon Pb treatment (*n* = 3, independent two-sample *t* test, two-tailed). **b** Relative miR-137 levels in response to Pb exposure at DIV4 (*n* = 8, independent two-sample *t* test, two-tailed). **c** Immunoblots and quantification of protein levels of EZH2 in PC 12 cells transfected with miR-137 mimics or the scrambled control (NC) (*n* = 4, Mann–Whitney *U* test; NC vs. 5 μM *P = 0.0214, NC vs. 10 μM **P = 0.0036). **d** Schematic illustration showing the seed region where rno-miR-137 is expected to bind the 3′-UTR of *EZH2*. **e** DLR (Dual Luciferase Reporter) assay of PC 12 cells co-transfected with psiCHECK-2 vector harboring normal or mutated 3′-UTR of *EZH2*, as well as miR-137 mimics or the scrambled control (NC), ~25 fluorescence intensity ratio was calculated for each group (independent two-sample *t* test, two-tailed; EZH2 **P = 0.0011). **f** Hippocampal neurons were transfected with lentivirus harboring the scrambled (Con-miR) and anti-miR-137 vector, and mRNA level of EZH2 (**f**) was subsequently examined, respectively (*n* = 3, independent two-sample *t* test, two-tailed). **g**–**k** The decrease of EZH2 up-regulates miR-137 expression through the diminished enrichment of H3K27me3. **g** Relative levels of pri-miR-137 at DIV4 and DIV14 in response to Pb exposure (*n* = 3, independent two-sample *t* test, two-tailed; DIV14 **P = 0.0083). **h**, **i** Enrichment peaks and ChIP-qPCR analysis depicting changes of H3K27me3 enrichment on the promoter region of miR-137. C1-3 and P1-3 represent the triplicate samples from hippocampal neurons in the absence and presence of Pb exposure. The graph following miRNA is generated from the chip-denoted database, while the others are originated from ChIP-on-chip data. For ChIP-qPCR, mock refers to the group immunoprecipitated with non-immune IgG protein (*n* = 3, independent two-sample *t* test, two-tailed). **j** Relative levels of miR-137 and pri-miR-137 of hippocampal tissues collected from rats infected with lentivirus harboring the empty (Vec) or pReceiver-EZH2 (EZH2-OE) vector (*n* = 3, independent two-sample *t* test, two-tailed, miR-137 **P = 0.0085). **k** Relative levels of pri-miR-137 in cultured neurons transfected with anti-miR-137-containing virus (*n* = 3, independent two-sample *t* test, two-tailed; column 3 vs. column 4 **P = 0.0037). The data are represented as mean ± SEM; ***P < 0.001, **P < 0.01, *P < 0.05, NS > 0.05. Symbols show individual samples. The different types of symbols represent the different groups. In **b**, the solid circle and solid square represent control and Pb, respectively. In **c**, the solid circle, solid square, and solid triangle represent control, 5 μM miR-137, and 10 μM miR-137, respectively. In **f**, the solid circle, solid square, solid triangle, and inverted solid triangle represent Con-miR, anti-miR-137, Con-miR-137+Pb, and anti-miR-137+Pb, respectively. In **k**, the solid circle, solid square, solid triangle, and inverted solid triangle represent control, Con-miR, Pb+Con-miR, and Pb+anti-miR-137, respectively

*EZH2* is a target for miR-25, miR-26, miR-138, and miR-137, and so on^24,25^. It is hypothesized that, in Pb-exposed neurons, a diminished EZH2 level might be attributed to inhibitory activity of microRNA (miRNA). To test this, stem-loop RT-PCR miRNA assays were performed. The candidate miRNAs were chosen because they are sequencing complementary to *EZH2* gene in rat brain. Among them, miR-137 displayed significantly increased accumulation upon Pb treatment in cultured hippocampal neurons at DIV4 (Fig. 3a, b). miR-101, although it was significantly upregulated, was ruled out due to its low abundance.

It was reported that miR-137 represses *EZH2* gene and protein levels in adult neurogenesis^32,33^, but the progressive repression does not take place during postmitotic neuronal maturation. Here, in our hands, miR-137 displayed a repressive contact with EZH2 in the neural context, as demonstrated by the intervention (Fig. 3c) and luciferase assays (Fig. 3d, e). With complementary sequences mutated, the EZH2-mutant construct failed to respond to the introduction of miR-137 (Fig. 3e), suggesting a direct repressive targeting for miR-137. Moreover, knockdown (KD) of miR-137 in the hippocampal neurons through a virus vector increased *EZH2* transcripts in hippocampal neurons in wild-type and Pb-exposed group (Fig. 3f). This result demonstrated that the Pb-induced EZH2 reduction could be reversed by the removal of miR-137. It also suggested that Pb treatment activates expression of miR-137, which in turn causes EZH2 reduction.

The miRNA genes are initially transcribed as primary miRNA transcripts by RNA polymerase II^34^. To further explore how Pb treatment alters miR-137 expression, we examined the level of pri-miR-137. Pb exposure promoted pri-miR-137 expression only at DIV14, but not DIV4 (Fig. 3g). One possibility is that Pb might alter the function of core microprocessor components to intervene the processing of pri-miR-137. To test this, we conducted RT-qPCR assay. The mRNA expression patterns of *Dicer*, *Drosha*, and *Exportin5* differed during neuronal maturation (Fig. S3). This implies that Pb might increase miR-137 at earlier stage probably through an indirect effect.

To further study how pri-miR-137 expression is regulated at a later stage, we conducted ChIP-chip (chromatin immunoprecipitation on chip) assays. In a ChIP-chip assay, we mapped H3K27me3 mark to the reference genome. Surprisingly, such a mapping unveiled the presence of H3K27me3 on the promoter region of miR-137 (Fig. 3h). Moreover, the ChIP-chip result was easily validated by the ChIP-qPCR analysis (Fig. 3i). Importantly, H3K27me3 enrichment on miR-137 promoter was decreased upon Pb exposure at DIV14. This result suggested that H3K27me3 is a repressive marker for miR-137 expression. To further test this hypothesis, we constitutively overexpress EZH2 in vivo. According to the results, overexpression of EZH2 reduced the amounts of both pri-miR-137 and miR-137 (Fig. 3j). Thus, EZH2 has repressive effect on miR-137, the very inhibitor for *EZH2* gene itself. Together, these results indicated the miR-137 and EZH2 constitute mutual inhibitory effect on each other, forming a self-feedback regulatory loop in vivo.

To further validate the interaction between miR-137 and EZH2, primary neurons were infected with miR-137-KD lentivirus at DIV3. Whereas Pb treatment significantly enhanced pri-miR-137 accumulation in the presence of a control anti-miRNA, the enhancement was substantially compromised when anti-miR-137 was administered (Fig. 3k). These results further indicated the presence of miR-137-EZH2 mutual regulatory loop. These findings also support that Pb activated the interplay of miR-137 and EZH2 in hippocampal neurons.

### Pb reshapes the genomic landscape of H3K27me3 occupancy

**Fig. 4:**
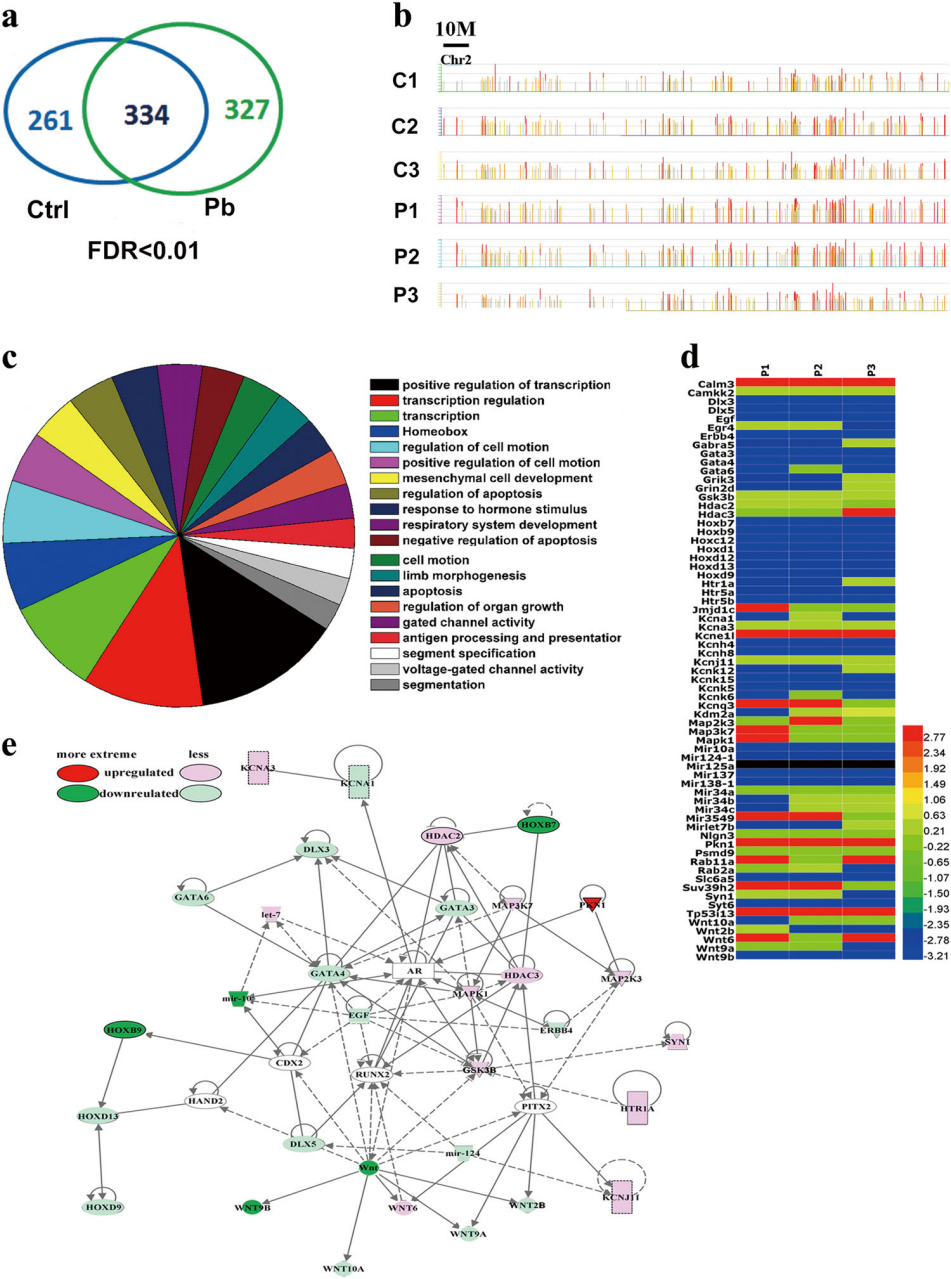
Genome-wide redistribution of H3K27me3 is elicited in hippocampal neurons by Pb exposure (*n* *=* 3). **a** Venn diagram of the ChIP-chip assay showing the number of genes bound differentially by H3K27me3 in the control and Pb-treated cultured neurons (FDR < 0.01). **b** Representative illustrations depicting changes of H3K27me3 enrichment across chromosome 2. C1-3 and P1-3 refer to the triplicate samples from hippocampal neurons in the absence and presence of Pb exposure. **c** Pie chart representing the top 20 differentially enriched pathways in response to Pb treatment, according to the ChIP-chip analysis (FDR < 0.01). **d** Heat maps showing the functional neural genes differentially enriched by the H3K27me3 upon Pb treatment (independent one-sample *t* test, two-tailed, *P* < 0.05). **e** Interaction networks of genes differentially regulated by H3K27me3, based on Ingenuity Pathway Analysis (independent one-sample *t* test, two-tailed, *P* < 0.05). The solid and dashed line refers to the direct and indirect interaction, respectively. These symbols represent distinct group of molecules: oval, transcription regulator; rectangle with solid borders, G protein coupled receptor; rectangle with dash borders, ion channel; triangle, phosphatase; inverted triangle, kinase; trapezoid, transporter; inverted trapezoid, miRNA; cycle, others

To investigate how Pb treatment might alter H3K27me3 dispositions in a genome-wide scale, ChIP-chip analysis was performed. With distinct binding peaks detected, 327 genes gained their H3K27me3 marks, while 261 genes lost them. Some of 334 common genes also experienced an enhanced or weakened occupancy (Fig. 4a; Fig. S4a). The occupancy profiles were exemplified by the binding maps of H3K27me3 at chromosome 2 (Fig. 4b). Gene Ontology analysis unveiled a complete picture of H3K27me3 regulatory targets in postmitotic neurons (Datasets S1–7).

We next determined if particular cellular pathways were enriched for H3K27me3 binding. The top 20 most highly enriched pathways were listed in Fig. S4b. Upon Pb exposure, distinct sets of genetic pathways were differentially enriched in cultured hippocampal neurons at DIV14 (Fig. 4c). Among them, the pathways involved in transcription regulation were preferentially remodeled, and other highlighted pathways include cell motion, apoptosis, and organ growth. Besides, the pathway of neuronal developmental regulation covered variable aspects of functional clusters (Fig. S4c).

We also analyzed the essential genes bound by H3K27me3. An array of differentially enriched genes and alterations of their relative enrichment were shown in a heatmap, according to the peaking data in chips (Fig. 4d). These functional genes included calmodulin (Calm3), glutamate [NMDA] receptor subunit epsilon-4 (Grin2d), neuroligin-3 precursor, synapsin-1 (Syn1), Syn2, and synaptotagmin-6 (Syt6). Moreover, Ingenuity Pathway Analysis analysis gave the entire picture of interactive network of target genes (Fig. 4e). Interestingly, a series of epigenetic elements, HDAC and miRNA for instance, showed a strong occupation by H3K27me3, suggestive of an interlaced regulatory network prone to Pb invasion. Moreover, many of the differentially expressed genes are involved in Wnt signaling, a consequence that theoretically makes sense based on their capacity of initiating a global response. Our analysis suggests that Pb exposure leads to the genome-wide redistribution of H3K27me3.

### Pb changes the bivalent state of the *Wnt9b* locus

**Fig. 5:**
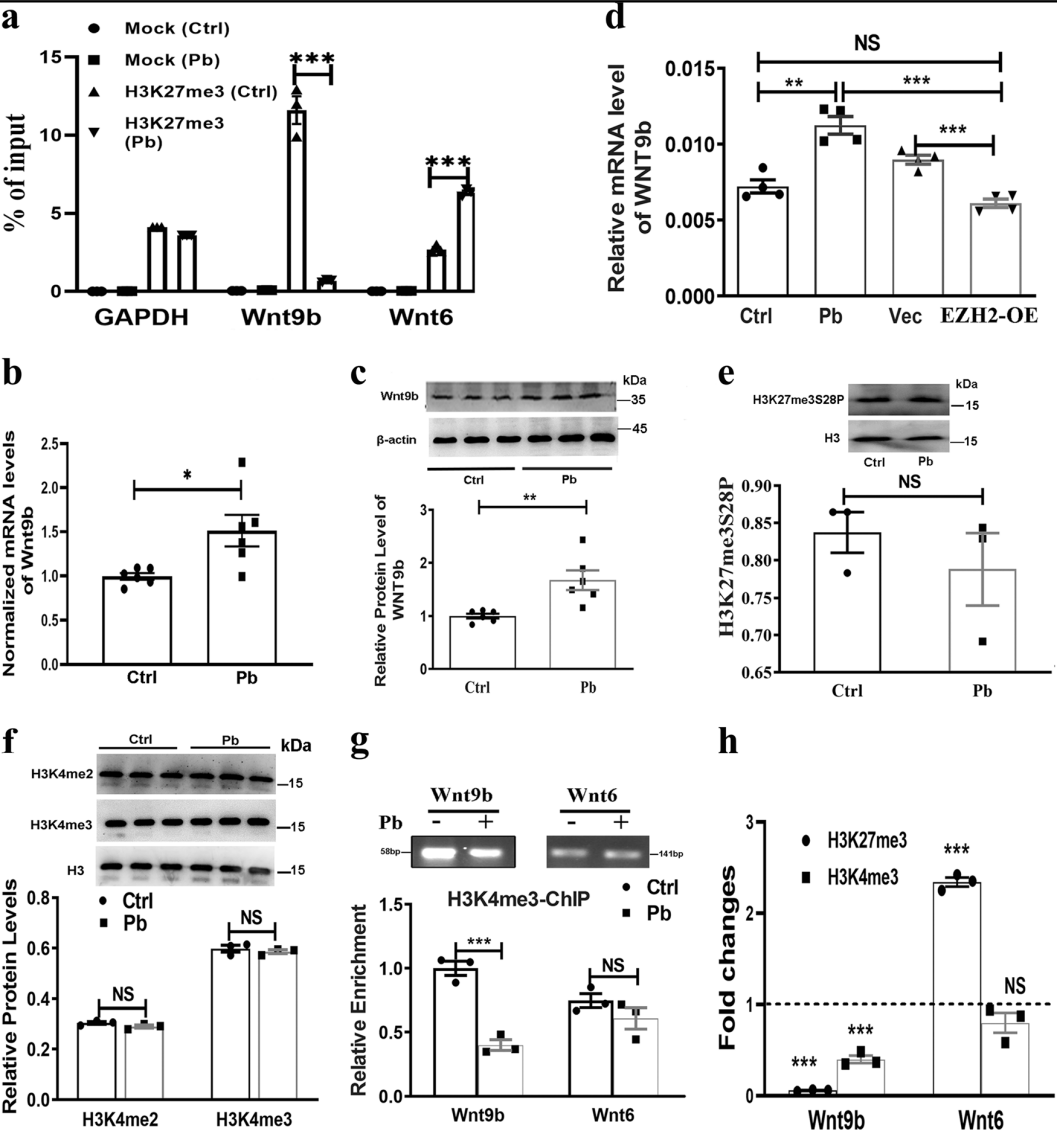
H3K27me3 targets Wnt9b in a bivalent manner. a–d H3K27me3 targets Wnt9b and influences its expression. **a** ChIP-qPCR analysis depicting changes of H3K27me3 enrichment on the promoter region of *Wnt9b* and *Wnt6*. Mock refers to the group immunoprecipitated with non-immune IgG protein (*n* = 3, Mann–Whitney *U* test; Wnt9b ***P = 0.0003). **b**, **c** Quantification of mRNA and protein levels of Wnt9b in response to Pb exposure (*n* = 6, mRNA: Mann–Whitney *U* test; Wnt9b *P = 0.0176; protein Mann-–Whitney *U* test; Ctrl vs. Pb **P = 0.0049). **d** Relative mRNA levels of *Wnt9b* of hippocampal tissues collected from rats infected with lentivirus harboring the empty (Vec) or pReceiver-EZH2 (EZH2-OE) vector (*n* *=* 4, independent two-sample *t* test, two-tailed; Ctrl vs. Pb **P = 0.0014). **e** Immunoblots and quantification of H3K27me3S28P protein level in cultured neurons exposed by Pb (*n* = 3, independent two-sample *t* test, two-tailed). **f**–**h** Bivalent occupancy of H3K27me3 and H3K4me3 on *Wnt9b* and *Wnt6*. **f** Immunoblots and quantification of levels of H3K4me2 and H3K4me3 in cultured neurons exposed by Pb (*n* = 3, independent two-sample *t* test, two-tailed, homoscedastic variance). **g** Validation and relative enrichment of H3K4me3 on *Wnt9b* and *Wnt6* in the context of Pb exposure (*n* = 3, independent two-sample *t* test, two-tailed). **h** Enrichment changes of H3K27me3 and H3K4me3 on *Wnt9b* and *Wnt6* in response to Pb exposure (*n* = 3, Mann–Whitney *U* test for H3K27me3, Wnt9b ***P = 0.0003; independent two-sample *t* test, two-tailed for H3K4me3). The data are represented as mean ± SEM; ***P < 0.001, **P < 0.01, *P < 0.05, NS > 0.05. Symbols show individual samples. The different types of symbols of figure 5 represent the different groups. In **b**, **c**, and **e**, the solid circle and solid square represent control and Pb, respectively. In **d**, the solid circle, solid square, solid triangle and inverted solid triangle represent control, Pb, Pb+Vec, and Pb+EZH2-OE, respectively

We previously reported that Wnt signaling (Wnt7a-dependent) is implicated in the Pb-led neuronal impairment^35^. Since ChIP-chip assays revealed new Wnt targets, we hypothesized that Pb might regulate Wnt signaling through changing H3K27me3 occupancy. To this end, we conducted ChIP-qPCR and found that H3K27me3 mark was bona fide enriched in the promoter region of *Wnt9b* gene in hippocampal neurons. Moreover, Pb treatment decreased the relative enrichment of this histone mark in the *Wnt9b* gene locus (Fig. 5a). In line with these results, the expressional levels of Wnt9b, both protein and transcript (Fig. 5b, c), were increased by Pb treatment. This result indicated Pb unleashed the negative regulation of H3K27me3 on Wnt9b expression. To further test this hypothesis, we infected rats with virus expressing EZH2. Upon the manual introduction of EZH2/H3K27me3, *Wnt9b* gene transcription was resumed to a comparable level in untreated rats, indicating that *Wnt9b* locus is the bona fide target for H3K27me3 (Fig. 5d). Besides, other Wnt-related proteins, GSK3β and Wnt6, were also regulated by H3K27me3, but GSK3β did not exhibit responses towards Pb exposure (Fig. S5a, b), and increased H3K27me3 occupancy did not repress Wnt6 expression as anticipated (Fig. 5a, Fig. S5c).

H3K27me3 is a stable marker, but still gains its plasticity with the modification of H3K27me3S28P, which removes the repressive roles of H3K27me3^36^. Thus, we need to find out if H3K27me3 enrichment indeed exerted repressive function in the studied context. Western blot assay did not reveal any significant differences of H3K27me3S28P level between the mock and Pb-treated cells (Fig. 5e). This result indicates that Pb led to changes of “active” H3K27me3, rather than a “dead” one with its original roles removed by S28 phosphorylation.

A common layer adding up to the complexity of histone regulation is bivalency. It is a co-localization of opposite modifications, typically H3K4me3 and H3K27me3. Bivalency prominently exists in pluripotent but also differentiated cells^17,37^. We explored whether Wnt9b were bivalently regulated. Pb treatment did not produce significant changes of total quantities of either H3K4me3 or H3K4me2 (Fig. 5f). However, H3K4me3 was detected at the promoters of both *Wnt9b* and *Wnt6* genes, and the enrichment on *Wnt9b*, but not on *Wnt6*, was altered by Pb treatment, a scenario reminiscent of H3K27me3 changes in response to Pb treatment (Fig. 5g). Overall, ChIP analysis confirmed the bivalent regulation of H3K4me3 and H3K27me3. The bivalent state on *Wnt9b* genes was simultaneously altered by Pb treatment (Fig. 5h). Besides, no significant differences were found in the promoter CpG methylation of Wnt gene (Fig. S6a–d).

These findings support that Wnt9b is bivalently regulated and this bivalency appears to respond to environmental cues of Pb invasion.

### Pb changes distinct roles of divergent Wnt signaling

**Fig. 6:**
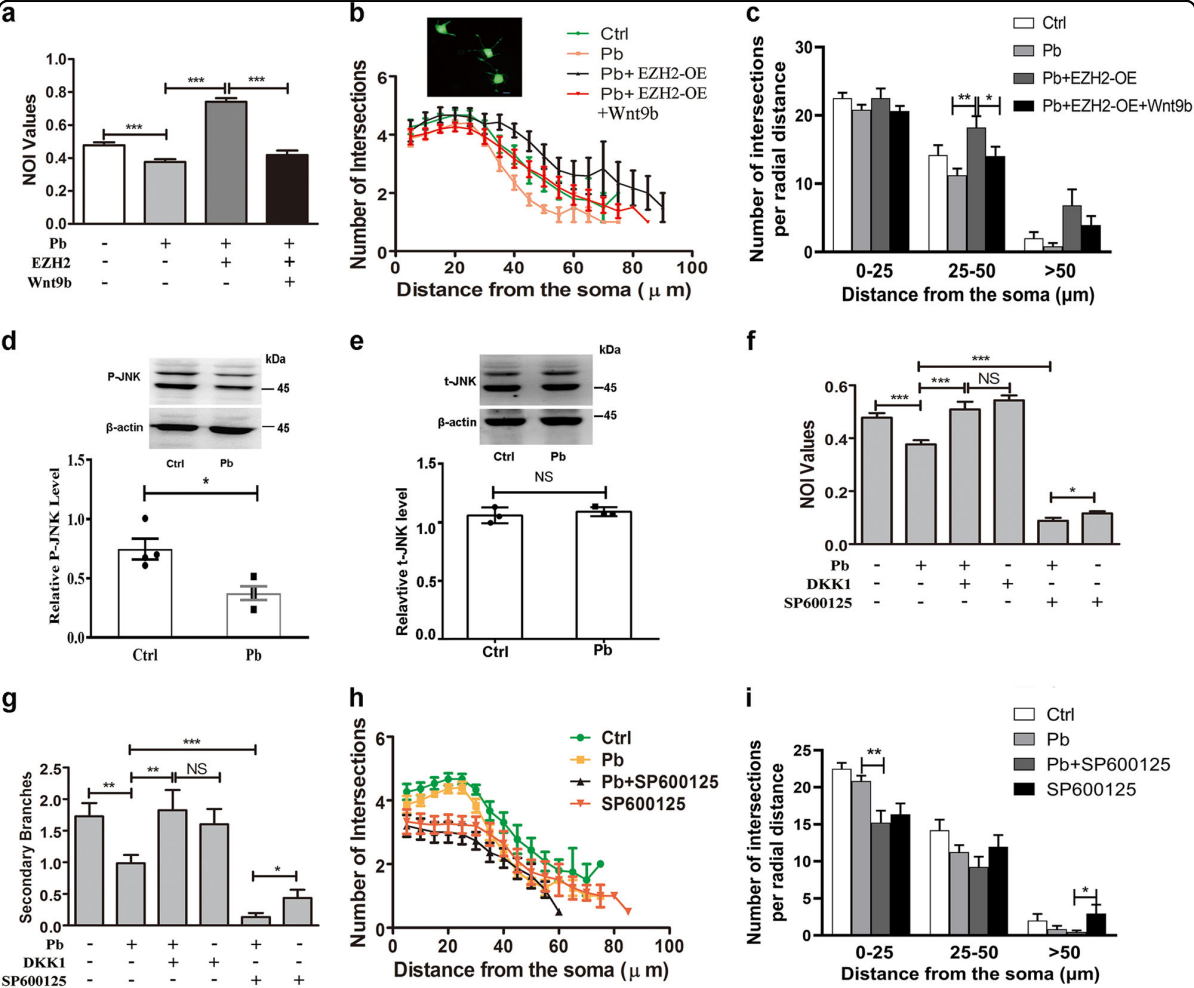
Wnt signaling divergently influences Pb-induced impairment. **a**–**c** NOI (neurite outgrowth index) values (**a**) and Sholl analysis (**b**, **c**) of differentiated PC 12 cells in response to Wnt9b recombinant proteins (200 ng/mL). Wnt9b was added to cell culture at 36 h prior to morphological analysis. EZH2 refers to the transfection of EZH2-overexpressing plasmid. The insert is the representative graph of transfected PC 12 cells (scale bar: 10 μm, ~50 graphs per experimental condition; **c** independent two-sample *t* test, two-tailed for column 6 vs. column 7 **P = 0.0011; Mann–Whitney *U* test for column 7 vs. column 8 *P *=* 0.0376). **d**, **e** Immunoblots and quantification of phosphorylated JNK (P-JNK) (*n* = 4, independent two-sample *t* test, two-tailed; Ctrl vs. Pb *P = 0.0124) and total JNK (*n* = 4, independent two-sample *t* test, two-tailed; Ctrl vs. Pb *P* > 0.05) in cultured neurons exposed by Pb. **f**–**i** NOI values (**f**, independent two-sample *t* test, two-tailed for column 1 vs. column 2, column 3 vs. column 4, column 5 vs. column 6 *P = 0.0375; Mann–Whitney *U* test for other comparisons), secondary branches (**g**, Mann–Whitney *U* test for column 1 vs. column 2 **P = 0.0034, column 2 vs. column 3 **P = 0.0074, column 2 vs. column 5, column 5 vs. column 6 *P = 0.0460; independent two-sample *t* test, two-*t*ailed for other comparisons) and Sholl analysis (**h**, **i**) of differentiated PC 12 cells in response to DKK-1 (400 ng/mL) or SP600125 (10 μM). The inhibitor was added to cell culture at 36 h prior to morphological analysis (*~*50 graphs per experimental condition; **i**, Mann–Whitney *U*-test for column 2 vs. column 3 **P = 0.0020, column11 vs. column12 *P = 0.0265). The data are represented as mean ± SEM; ***P < 0.001, **P < 0.01, *P < 0.05, NS > 0.05

To test if Wnt9b connects H3K27me3 with Pb-led impairments, we added Wnt9b to PC 12 cells previously transfected with EZH2-overexpressing vector, and measured NOI (neurite outgrowth index) values (Fig. 6a) and intersections defined by Sholl analysis (Fig. 6b, c). Overexpression of EZH2 resulted in an overwhelming rescue of neurite outgrowth deficits due to Pb exposure. Remarkably, with the addition of Wnt9b, the neural differentiation was re-damaged to a comparable level with the lead-exposed cells. Thus, Pb exerts its adverse effect on memory deficit through the changes of H3K27me3-Wnt9b pathway.

Depending on cell type and physiological environment, Wnt9b can signal through either canonical or noncanonical (Rho-JNK) pathway^38,39^. To delineate the contribution of individual Wnt signaling to Pb-led impairments, the protein levels of phosphorylated JNK (P-JNK) and total JNK (Fig. 6d, e) were examined. Total JNK level was not altered in the Pb-treated hippocampal neurons. P-JNK was decreased in response to Pb exposure, suggesting a repressive status of noncanonical pathway.

Interestingly, our previous study showed that Pb treatment damaged canonical Wnt signaling through destabilizing β-catenin^35^. To investigate roles of either pathway in Pb-induced neural adversity, we extended our analysis by using selective inhibitors: DKK-1 and SP600125 for canonical and noncanonical pathway, respectively. As evidenced by NOI (Fig. 6f) and secondary branching (Fig. 6g), the blockade of canonical pathway led to a repaired growth in the deficient cells, while the inhibition of noncanonical pathway only exacerbated the Pb-led consequences. Moreover, when canonical pathway was closed, detrimental outcomes were abolished. When Rho-JNK branch was ablated, Pb could still induce outgrowth deficits. Besides, a Sholl analysis with SP600125 validated the protective roles of noncanonical Wnt signaling (Fig. 6h, i).

In summary, Wnt signalings differ in their roles of mediating Pb-led deficits.

## Discussion

**Fig. 7:**
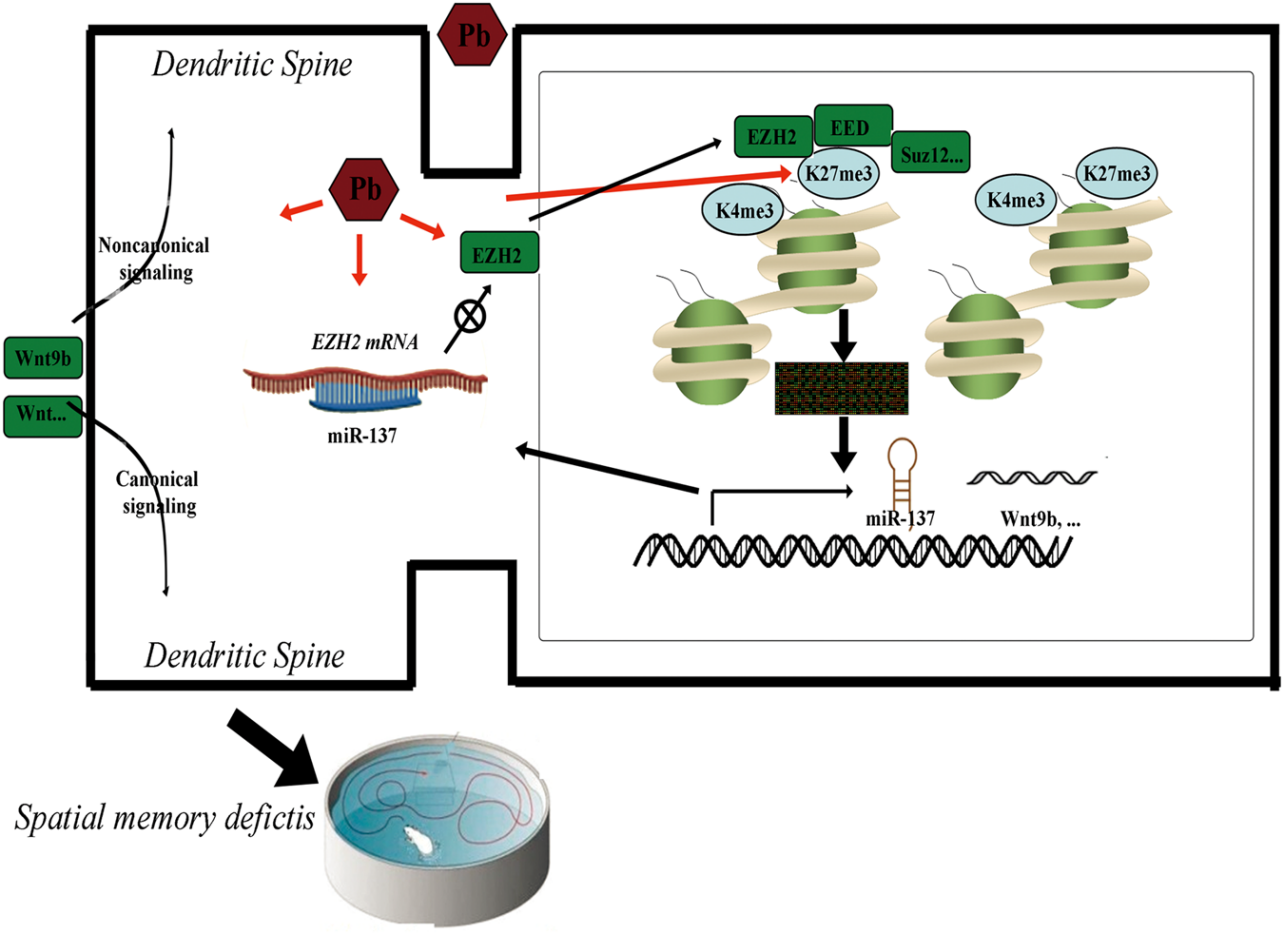
Proposed model of H3K27me3’s roles in spatial memory deficits caused by Pb exposure. Upon Pb exposure, a series of epigenetic events occur in hippocampal neurons: the prompted interplay between miR-137 and EZH2, a diminished H3K27me3 level, and genome-wide redistribution of its occupancy, as well as the divergently altered Wnt signaling

Pb interacts with diverse proteins to impair cell function and a variety of molecular mechanisms have been suggested to explain lead neurotoxicity. These include effects on proteins encoding synaptic plasticity and transmitter release, glutamate metabolism, calcium homeostasis, oxidative or proinflammatory stress, neuronal apoptosis, and so on^40–42^. Recently, accumulating evidence has implicated epigenetic mechanisms in the Pb-induced memory impairment^41,43,44^, since epigenetic modifications merit serious consideration as candidate mediators of “short-term exposure, long-term responsiveness,” and in governing multiple gene targets. The involvement of epigenetic mechanisms is supported by the findings in the present paper: one epigenetic pathway, centered at H3K27me3 and manifested as miR-137-EZH2-H3K27me3-Wnt9b, changed coincidentally in the context of Pb-dependent memory deficits (Fig. 7). This observation brings us a step closer to understanding the molecular mechanisms of Pb neurotoxicity. However, it per se cannot explain all the observed features.

Links might exist between the epigenetic mark and the alternative molecular pathways previously shown to participate in lead neurotoxicity. The essential genes bound and differentially enriched by H3K27me3 span various functional roles (Fig. 4d), such as calcium homeostasis (Calm3, Camkk2), NMDA receptors (Grin2d), cell apoptosis (Dataset S1–7, synaptic transmission (Syn1, Syn2, Syt6), and so on. Given the foundation of ChIP technique, these genes were dynamically occupied by H3K27me3 in the context of lead neurotoxicity, implying that their expression might be regulated by this epigenetic mark. Our recent finding supported this argument, as the transcription of Syn1 was negatively correlated with the Pb-triggered increase of H3K27me3 in the *Syn1* locus^45^. While this study demonstrated the regulatory relations of H3K27me3-Wnt9b, this relay should not be regarded as the sole pathway that H3K27me3 governed in regulating memory deficits.

One of the interesting findings here is the reciprocal loop of miR-137 and EZH2. Of note, miRNAs play important roles in neural function, and multiple miRNAs target EZH2^46,47^. According to miRNA profiling, the role of miR-137 was primarily studied in the Pb-treated neurons. As a brain-enriched miRNA, miR-137 was known to regulate neuronal maturation, but not through the inhibition of EZH2^33^. However, this silent pathway was aberrantly stimulated in postmitotic hippocampal neurons in a time-dependent fashion by Pb exposure. A similar loop was also proposed recently: MYC-miR-26a-EZH2 in aggressive B cell lymphoma^48^. Since multiple miRNAs can bind the same target, we could not exclude the possibility that other miRNAs also participated in the inhibition of EZH2 expression. Despite it, the formation of miR-137-EZH2 loop in the studied context is anticipated to be exclusive, because miR-137 is the only EZH2-targeting miRNA identified by H3K27me3 ChIP-chip analysis. A predicted advantage of this activated crosstalk is to accomplish and maintain the timely and efficient modulation of stable methyl marks.

Although traditionally regarded as a repressive mark, H3K27me3 in promoters is also suggested to activate gene expression^49^. Thus, the relation between histone methylation and transcription activity is not a simple one, and probably dependent on individual genetic microenvironment. Furthermore, gene expression is not decided by H3K27me3 alone, but rather a combinatorial consequence of multiple histone factors, like bivalency. In pluripotent cells, bivalent chromatin state allows genes to be “primed” for expression but “held in check” by opposing chromatin modifications. Bivalent regulation was also found present in differentiated cells, but its physiological significance is elusive. One hypothesis is that bivalency confers plasticity on the cellular epigenetic memory^50^. It is supported by the findings that Pb exposure, an environmental insult, interferes with bivalent gene expression exemplified by Wnt9b. Alternative chromatin states were specified by “histone code,” a “language” controlling gene expression with combinations of diverse modifications. Therefore, future efforts are warranted to decipher the explicit histone code involved in the memory deficits.

Our findings may have pharmaceutical relevance. Histone modifications are reversible, hence they might be suitable targets for therapeutic intervention^51^. Considering the pivotal roles of H3K27me3, a selective and potent modifier may be therapeutic in treating Pb-induced spatial memory deficits. Additionally, because memory deficits are paradigm symptoms of a range of psychiatric abnormalities, such interventions may also potentially benefit patients suffering from the widespread disorders, such as Alzheimer’s or Parkinson’s disease.

Taken together, among all the possible molecular targets, a new epigenetic pathway centered at H3K27me3 was suggested to mediate Pb-dependent memory impairment, and the mediating relations were demonstrated by the in vivo rescue trials, which could thereby be viewed as a further step towards understanding the molecular development of neurodegenerative disease with major environmental cues.

## Materials and methods

### Antibodies and reagents

Antibodies of anti-H3K27me3 (ab6002), anti-H3K4me3 (ab8580), and anti-H3K4me2 (ab7766) were purchased from Abcam (Shanghai, China). Anti-EZH2 (07-689) antibody was purchased from Millipore (Shanghai, China). Anti-EED antibody was purchased from Proteintech (Anhui, China). Anti-H3 (9715S), anti-P-JNK (#4668), and anti-JNK (#9252) antibodies were purchased from Cell Signaling Technology (Boston, MA, USA). Anti-IgG antibody was purchased from Invitrogen (Shanghai, China). Anti-H3K27me3S28P (49-1015) antibody was purchased from Thermo Fisher Scientific (Beijing, China). Wnt9b recombinant protein was purchased from Sigma-Aldrich (Shanghai, China). DAPI (4′,6-diamidino-2-phenylindole) was purchased from Sinopharm Group (Beijing, China), and miR-137 mimics was purchased from RiboBio (Guangzhou, China). All other reagents were of the highest analytical grade.

### Plasmids and lentivirus

Lentivirus vector pReceiver-Lv122C was purchased from GeneCopoeia (Guangzhou, China) and dual luciferase vector psiCHECK-2 was purchased from Promega Corporation (Beijing, China). The mutated psiCHECK-EZH2 vector was constructed by Site-Directed Mutagenesis Kit (TransGen Biotech, Beijing, China). The lentivirus was packaged by GeneCopoeia with virus titer reaching at least 10^8^ transducing units (TU)/mL. Lentivirus harboring miR-137-KD vector HYFW-lenti-KD137 was purchased from Obio Technology (Shanghai, China) with virus titer reaching at least 10^8^ TU/ml.

### Experimental animals and treatment, behavioral test, and spine counting

SD rats were obtained from the Laboratory Animal Center of Anhui Medical University. All animal procedures were carried out in accordance with National Institute of Health Guide for the Care and Use of Laboratory Animals and were approved by the Institutional Animal Care and Use Committee of Hefei University of Technology, China.

The lead acetate (PbAc) (250 p.p.m.) was administered ad libitum in drinking water. Three-month old female (250 ± 20 g) SD rats (*n* = 8) were co-housed with sexually mature males (2:1) for a week. After a week, each dam was separated and placed in an individual cage. Dams subjected to Pb treatment started to receive 250 p.p.m. PbAc in drinking water ad libitum. The control dams received drinking water until weaning of the offspring (PND21). Pups were weaned at PND21 and placed in new cages. From that moment, pups from the Pb-treated groups directly received PbAc (250 p.p.m.) in drinking water ad libitum until PND60. The Pb-exposed model was referenced to the previous studies^52,53^. Given the observation that male pups did not show significant memory damage in the studied context (data not shown), female pups were randomly selected for the subsequent behavioral assessment and other trials.

The rats were subjected to the behavioral assessment at PND60. MWM test was performed as described previously^8^. Briefly, experiments were performed in a circular pool with a diameter of 1600 mm and depth of 700 mm, which was filled with water to a depth of 400 mm. The temperature was maintained at 25 °C. The maze was filled with water and rats were trained to find the hidden platform for 5 consecutive days, and underwent four training rounds per day with a 30-s interval. Each round of training was designed as follows: upon reaching the platform, the rat was allowed to stay on it for 30 s. If it failed to touch the platform within 90 s, it was guided to stay there for 30 s. The platform was removed on the sixth day, namely the test day, and each rat was subjected to a 90 s trial to test its memory retention. The moving tracks were video-recorded and automatically scored by Smart tracking software (ANY-maze; Stoelting, USA). The platform-crossing times, time spent on the target quadrant, and mean traveling speed were analyzed.

Hippocampus was removed from the decapitated rats within 1 min at PND21 and PND60, and then subjected to Golgi–Cox staining for spine density analysis, as described previously^35^.

### Cell cultures

Primary neuronal cultures were prepared from rat brains at PND0. Briefly, rat hippocampus was dissected and dissociated with trypsin (0.03%) for 19 min, triturated, and plated on dishes (100 cells per mm^2^) with serum-free neurobasal media (supplemented with B27 and glutamax) precoated with poly-l-lysine (0.5 mg/mL). At DIV7, half of the media were renewed with fresh neurobasal media supplemented with Ara-C (2 μL/mL). For Pb exposure, PbAc (5 μM) was added to culture at DIV3^45^. The Pb dosage used in the cell experiment is selected to establish a replicable neuronal deficit model, based on its performance in the previous reports^45,54^ and inability to cause excessive cell death (data not shown).

PC 12 cell line was used to profile neurite outgrowth upon plasmid transfection and Wnt9b supplement due to its availability for multiple genetic manipulations^55^. Undifferentiated PC 12 cells were cultured with RPMI-1640 medium supplemented with 5% fetal bovine serum (FBS), 10%horse serum, and 1% penicillin–streptomycin. Cells were plated onto 6-well culture coated with poly-d-lysine/lamine. For Pb exposure, PbAc (5 μM) was added to culture at 24 h of growth. Undifferentiated cell lines were re-inoculated with fresh media supplemented with NGF (50 ng/mL) at 24 h. Following another 72 h of incubation (with medium changed every 36 h), morphological analysis was performed according to images from fluorescence microscopy (Nikon, Tokyo, Japan), and manifested by NOI^56^, secondary branching and Sholl (Image J software) analysis. EZH2-OE plasmid (500 ng) was transfected into cells using Lipofectamine 3000 (Thermo Fisher Scientific, Beijing, China) at 24 h of growth.

### Stereotactic injections and lentivirus transfection

A small craniotomy was performed, and 1 μL lentivirus particle (10^8^ TU/mL) was stereotaxically injected into the bilateral hippocampus (relative to Bregma: anteroposterior, −2.3 mm; lateromedial, ±1.7 mm; dorsoventral, −2.0 mm) during a time window of 10–20 min at PND10. After infusion, the capillary was left in place for additional 10 min to allow complete diffusion of the virus. The skin incision was closed carefully after lentiviral injection to minimize inflammation. Rats were allowed for 10 days recovery prior to fluorescence examination through Virtual Slide Microscope VS120 (Olympus, Tokyo, Japan).

For neuronal infection, primary hippocampal neurons were inoculated into 24-well plate with the concentration of 10^5^ cells/well. At DIV4, primary neurons were infected using a multiplicity of infection of 20. After 12 h of incubation, the medium was removed and replaced with fresh medium. Samples were collected 10 days later and serial assays were performed.

### Quantitative RT-PCR analysis

The transcriptional levels of the objective genes were measured through quantitative real-time RT-PCR protocols. First, the total RNAs were extracted from rat hippocampus using the PureLink RNA mini kit (Thermo, Shanghai, China). Subsequently, the RT reaction was completed according to the manufacturer’s instruction (TransGen, Beijing, China), resulting in the first strand of total complementary DNA (cDNA). Real-time PCR was performed on cDNA templates using the Roche LightCycler 96 (Shanghai, China). The 20 μL reaction pool of qPCR was composed of: 10 μL of SYBR premix Extaq; 0.6 μL of forward and reverse primer each; 1 μL of cDNA template (10 times dilution); and 7.8 μL of deionized water. The primers used in this study were listed in Table S1. The results were normalized against β-actin as an internal control.

### Immunoblots, co-IP, and immunofluorescence

Rat hippocampi were homogenized in the ice lysis buffer (137 mM NaCl, 2.7 mM KCl, 10 mM Na_2_HPO_4_, 2 mM KH_2_PO_4_, 21 μg/mL aprotinin, 0.5 μg/mL leupeptin, 4.9 mM MgCl_2_, 1 mM NaVO_3_, 10% Triton, 1 mM phenylmethylsulfonyl fluoride) and dissociated for 1 h. Cultured cell lysates were prepared in Laemmli lysis buffer. Western blotting was then performed as described previously^8^. Co-IP assay was carried out using Nuclear Complex Co-IP Kit (Active Motif, Carlsbad, CA, USA) according to the manufacturer’s instructions.

For immunofluorescence assay, neuronal cells grown on coverslips were rinsed in phosphate-buffered saline (PBS) and fixed in 4% paraformaldehyde at DIV14, followed by additional fixation in ice-cold methanol. A measure of 0.2% of Triton X-100 was added to penetrate the membranes for 30 min and cells were washed three times before being blocked by 5% FBS. Fixed cells were sequentially incubated in anti-H3K27me3 antibody overnight and second antibody for 1 h at 4 °C. Coverslips were mounted onto slides in Prolong Gold mounting media with DAPI. Fluorescent imaging was performed using Zeiss LSM 710 Confocal Microscope (Oberkochen, Germany) according to the manufacturer’s instructions.

### Chromatin immunoprecipitation

The collected neuronal cells were cross-linked by 1% formaldehyde at 37 °C for 10 min. The fixed cells were rinsed twice with PBS and lysed using lysis buffer (0.1% sodium dodecyl sulfate, 0.5% Triton X-100, 20 mM Tris-HCl [pH 8.1], 150 mM NaCl, protease inhibitor, Roche, Shanghai, China). Lysed cells were sonicated using a Diagenode Bioruptor UCD-200 at high power in ice water. Sonicated fragments ranged from 200 to 1000 bp. Samples were then precleared at 13,000 × *g* for 10 min, pre-absorbed by 50 μL protein A/G beads (Santa Cruz, Dallas, TX, USA) and incubated with H3K27me3 (or H3K4me3) antibodies overnight at 4 °C. The complex was sequentially washed by lysis buffer, LiCl buffer, TE buffer, and eluted using fresh elution buffer (10 mM Tris-HCl, pH 8.0; 5 mM EDTA; 300 mM NaCl; 0.5% SDS) and reversely cross-linked. Immunoprecipitated genomic DNA was then purified using a QIAGEN Purification Kit (Shanghai, China) and dissolved in EB buffer for the following ChIP-chip analysis and qPCR assay.

For ChIP-chip analysis, the enriched DNA was amplified using a Whole Genome Amplification Kit (Sigma-Aldrich, Shanghai, China). The amplified DNA samples were purified with QIAGEN Purification Kit, labeled using NimbleGen Dual-Color DNA Labeling Kit, and then subjected to the ArrayStar Rat RefSeq Promoter Array for hybridization. Hybridization was performed at 42 °C during 16–20 h with 4 μg of Cy3/5-labeled DNA. A permutation-based peak-finding algorithm provided by NimbleScan v2.5 (Roche) was applied to find peaks, which represent significantly positive enrichment. qPCR assay was performed using LightCycler 96 system with SYBR Green Mix, and primers used in this study were listed in Table S1.

### miRNA profiling and quantification

Total small RNAs were extracted from cultured neurons at DIV14 using mirVana miRNA Isolation Kit (Life Technologies, Shanghai, China). Multiplex miRNA profiling was performed as described previously^57^ with some modifications. Briefly, RT was performed using multiple stem-loop primers (200 nM for each miRNA) from TransGen miRNA First-Strand cDNA Synthesis Kit, according to the manufacturer’s instructions. Subsequently, a pre-PCR protocol with multiple forward primers and single consensus reverse primer (shown in Table S1) was conducted as follows: 1 cycle of 95 °C for 10 min and 55 °C for 2 min; 18 cycles of 95 °C for 1 s and 65 °C for 1 min. The resultants were diluted 100 times prior to the standard qPCR protocol with primers corresponding to the single miRNA. Quantification of miR-137 was conducted with the standard qPCR protocol, using the first-strand cDNA synthesized with the miR-137-specific stem-loop primer.

### Luciferase assay

When PC 12 cell number reached 10^6^/well on a 96-well plate, the luciferase vector (psiCHECK-2 with normal or mutated 3′-UTR of *EZH2*, 3 μg) was co-transfected with miR-137 mimics (10 μM) using Lipofectamine 3000 (Thermo Fisher Scientific). The cells continued to grow for 48 h before being collected and lysed with Dual-Luciferase Reporter Assay System Kit (Promega, Shanghai, China). The luciferase activity was measured using Thermo Scientific Varioskan Flash. rLuc activity was normalized to fLuc activity to account for variation in transfection efficiencies. Luciferase experiments were repeated at least three times.

### CpG methylation

CpG methylation in the promoter region of *Wnt6* was examined as described previously^58^. The primer pairs used in the nested PCR were TGTYGTAAGGTTAGGGAGYA-AATACRAATCCCRCCTRC (first set) and TTTGTTGGGATAGAGGYTGAAGATAACTATAAARARARAAAACTTCCCA (second set).

### Statistical analysis

Graph data was presented as means ± SEM. Statistical analysis was performed using the SPSS software. Unpaired, two-tailed *t* test was used to perform two group comparisons. Two-way ANOVA was applied in the training data in MWM. Nonparametric Wilcoxon’s two-sample tests were conducted in explaining CpG methylation data. ChIP-chip peaks were presented by SignalMap Software and data were analyzed using DAVID Bioinformatics Resources 6.8 (NIH). The number of samples examined in each analysis was shown in the legends.

## Supplementary information

Supplementary Materials

Supplementary Table 1

Supplementary Figure 1

Supplementary Figure 2

Supplementary Figure 3

Supplementary Figure 4

Supplementary Figure 5

Supplementary Figure 6

Dataset 1

Dataset 2

Dataset 3

Dataset 4

Dataset 5

Dataset 6

Dataset 7
