# Supplementary material for: Interplay of miR-137 and EZH2 contributes to the genome-wide redistribution of H3K27me3 underlying the Pb-induced memory impairment
Source: Cell Death Dis. 2019 Sep 11;10(9):671. doi: 10.1038/s41419-019-1912-7 (PMC6739382; doi:10.1038/s41419-019-1912-7)
Supplement: Supplementary file 1 — Supplementary Materials [file 41419_2019_1912_MOESM1_ESM.docx]

**Supplementary Figures**

**Fig. S1**


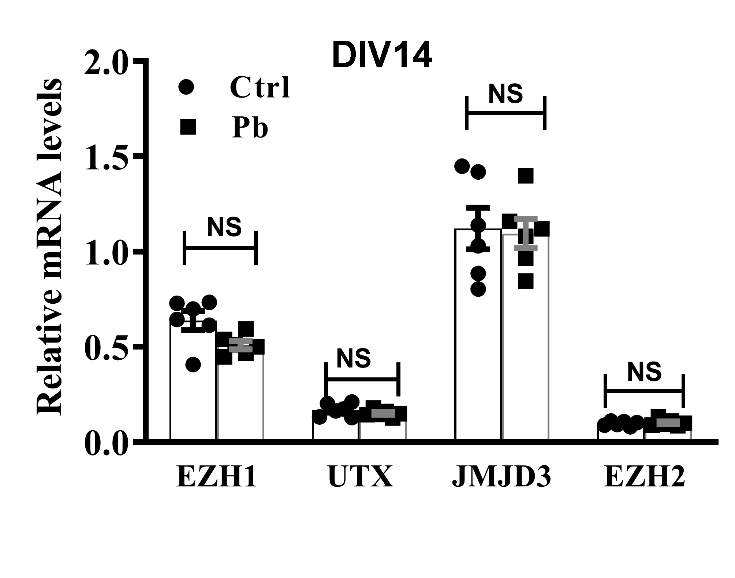


**Fig. S1.** Relative mRNA levels of *EZH1*, *EZH2*, *UTX* and *JMJD3* upon Pb treatment at DIV 14 (*n* = 6). The data are represented as Mean ± SEM, NS, *P*>0.05.

**Fig. S2**


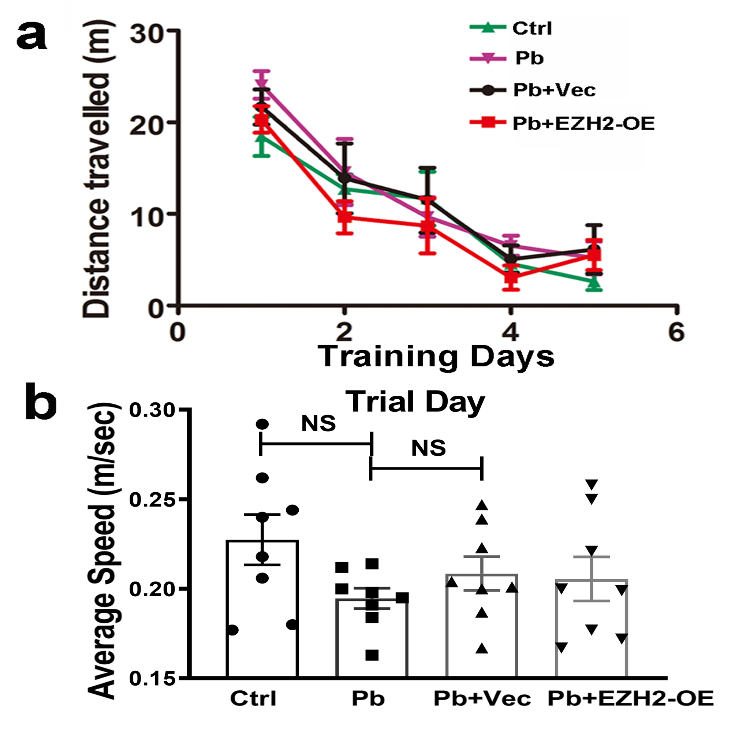


**Fig. S2.** Behavioral tests assessing rats’ capacities to be trained to find the hidden/removed platform (*n* = 8). Distance travelled (a) were recorded and analyzed during the training days. On trial day, the average speed (b) during the entire movement was recorded and calculated. Vec and EZH2-OE refer to the rats infected with lentivirus harboring the empty and pReceiver-EZH2 vector, respectively. The data are represented as Mean ± SEM, NS, *P*>0.05.

**Fig. S3**


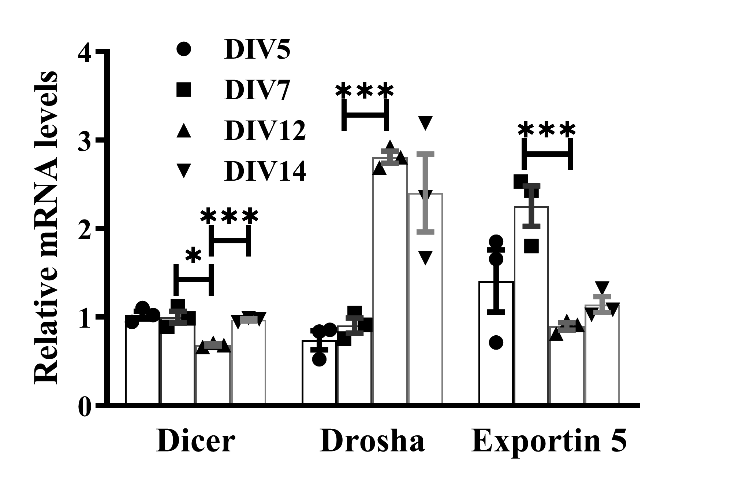


**Fig. S3.** Relative mRNA levels of *Dicer*, *Drosha* and *Exportin5* at various culture phases of primary hippocampal neurons (*n* = 3). The data are represented as Mean ± SEM; ****P* < 0.001, **P* < 0.05.

**Fig. S4**

**
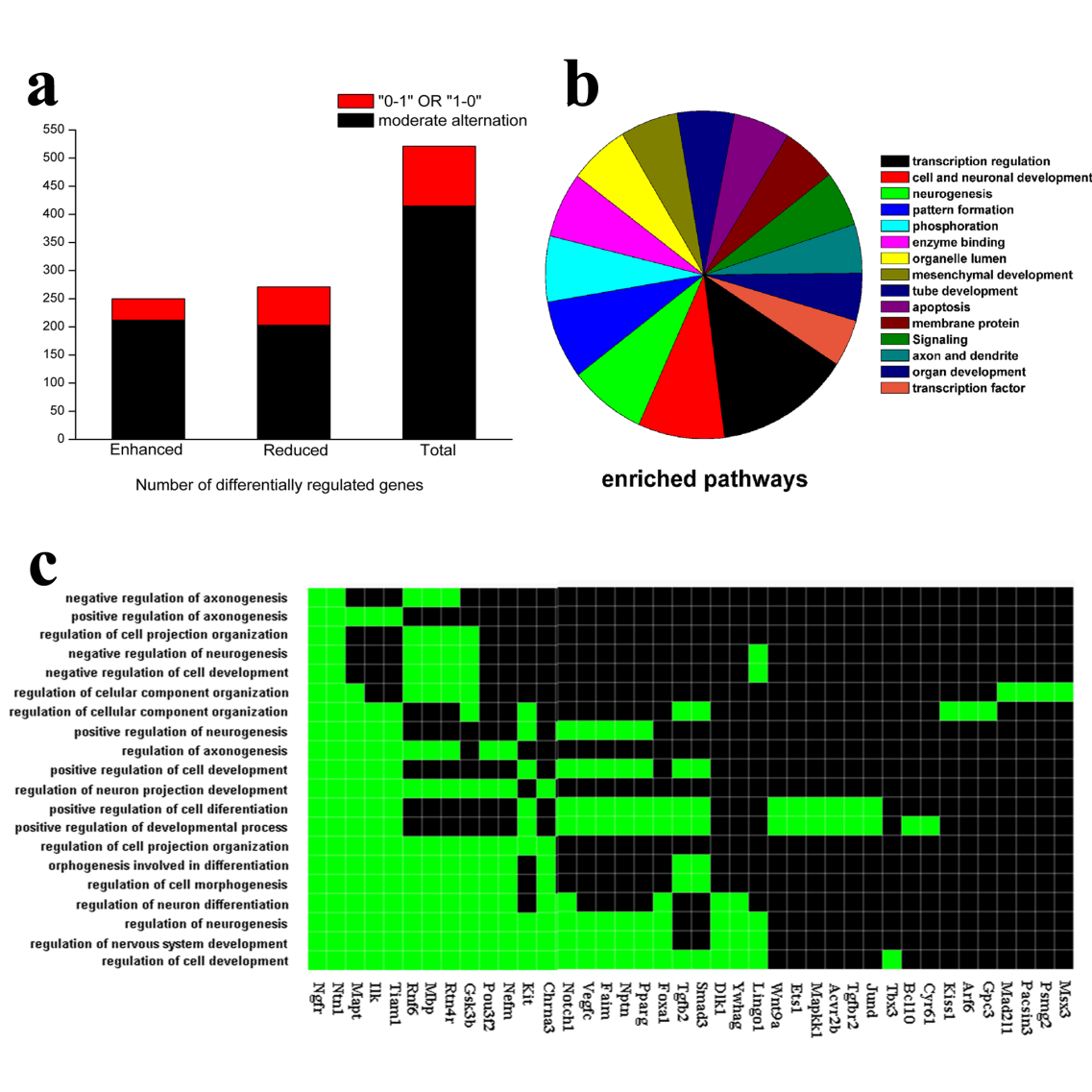
**

**Fig. S4.** Analysis of genes and pathways regulated by H3K27me3 in primary neurons (*n* = 3). (a) Number of genes differentially regulated by H3K27me3 upon Pb exposure in hippocampal neurons (*n* = 3). The red area represents the genes only regulated under either studied condition. The black area represents the genes enriched to a variable extent in the presence and absence of Pb exposure; (b) Venn diagram of the ChIP-chip assay showing the number of genes bound by H3K27me3 in the Pb-treated neurons. FDR < 0.01; (c) Functional clusters of H3K27me3-enriched genes involved in the neuronal developmental regulation in the Pb-treated neurons. The genes were obtained from the ChIP-chip trials of H3K27me3 and analyzed using DAVID Gene Functional Classification Tool. The green box represents the genes clustered to the corresponding functions. The black box represents the genes not clustered to the corresponding functions.

**Fig. S5**


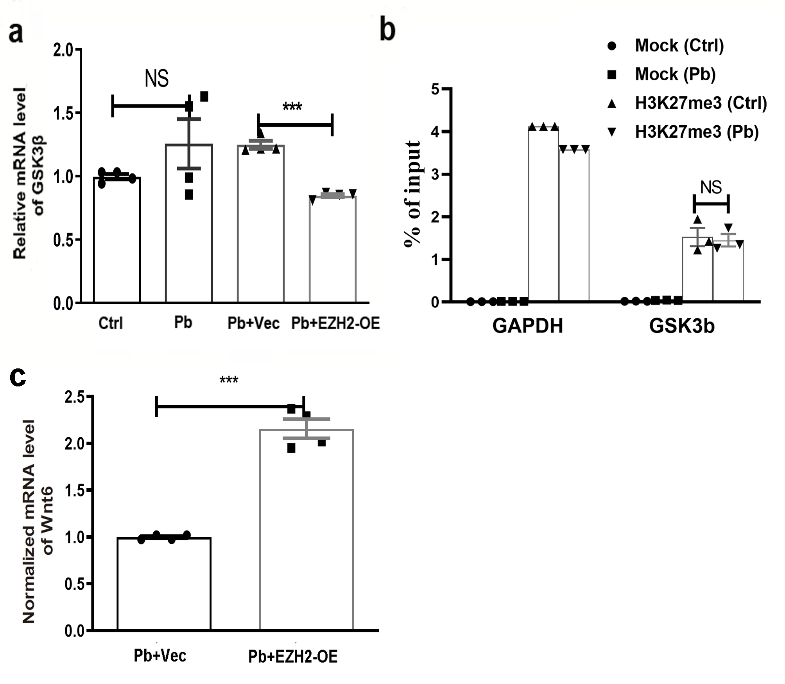


**Fig. S5.** H3K27me3 regulation of the expression of *GSK3β* and *Wnt6*. (a) Relative mRNA levels of Gsk3β in rats hippocampus injected with EZH2-overexpressing lentivirus (*n* = 4). (b) ChIP-qPCR analysis depicting changes of H3K27me3 enrichment on the promoter of Gsk3β in cultured neurons. GAPDH was used as a positive control, and mock refers to the group immunoprecipitated with non-immune IgG protein (*n* = 3). (c) Relative mRNA levels of *Wnt6* in the hippocampus of rats injected with EZH2-overpressing lentivirus at PND20 (*n* = 4). The data are represented as Mean ± SEM; ****P* < 0.001, NS *P* > 0.05.

**Fig. S6**

**
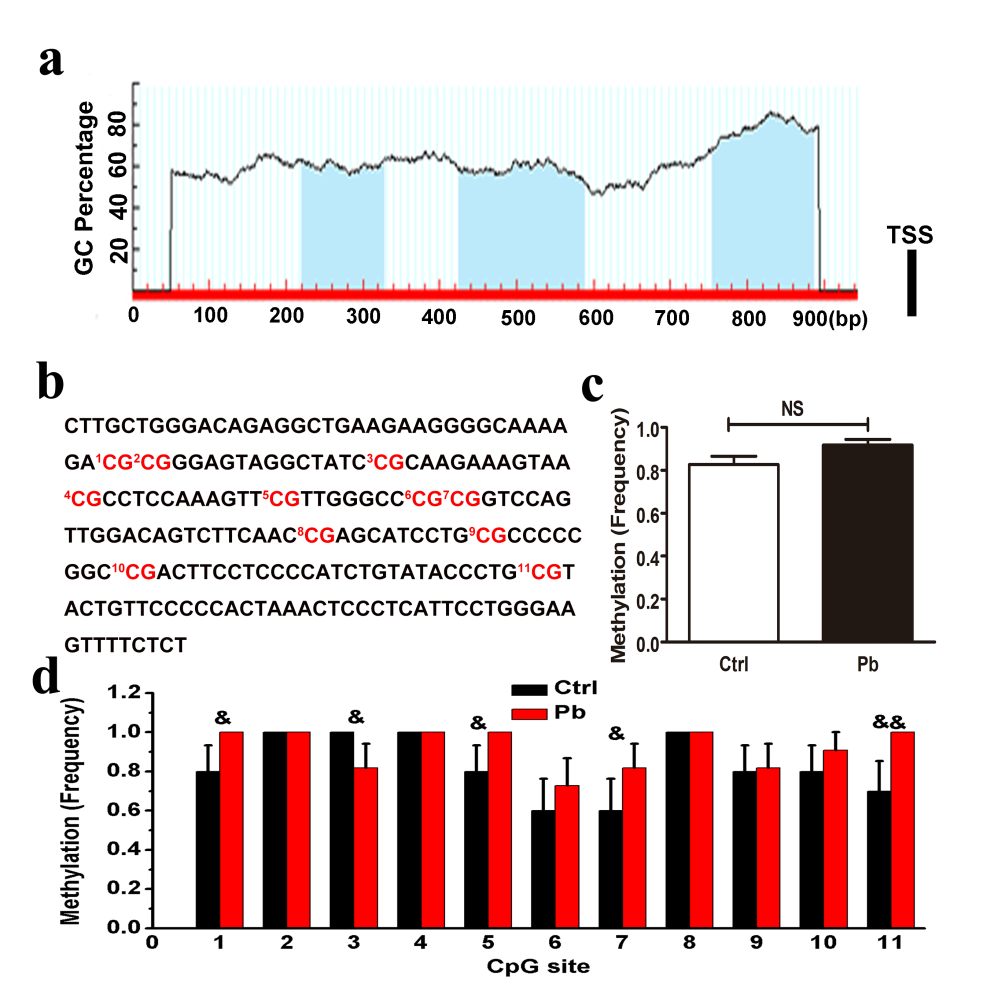
**

**Fig. S6.** CpG methylations in the promoter region of *Wnt6* upon Pb exposure. (a) “Methprimer” was used to perform CpG island prediction of *Wnt6*’s promoter region, termed as the “-1000 ~ 0” region of TSS (Transcription Start Site). The predicted CpG islands were marked as the blue shadow; Total (b) and individual (c) CpG site methylation was measured using bisulfite sequencing, in terms of 11 critical CpG sites from the CpG Island: 5’-AGGGGCAAAAGACGCGGGAGTAGGCTATCCGCAAGAAAGTAACGCCTCCAAAGTTCGTTGGGCCCGCGGTCCAGTTGGACAGTCTTCAACCGAGCATCCTGCGCCCCCGGCCGACTTCCTCCCCATCTGTATATCCCTGCGTACTGTTCCCCC-3’. The data are represented as Mean ± SEM; NS indicates *P* > 0.05, & indicates differences no less than 1.5 SEM, && indicates differences no less than 2 SEM.

**Supplementary Table**

**Table S1. Primers used in this study**

| **Primers** | **Sequences (5’-3’)** | **Methods** |
| --- | --- | --- |
| EZH1F | CCAATACATCCGCCTCTGCTA | qPCR |
| EZH1R | CTGGACTAGCTTTCTGTTTCGTG | qPCR |
| UTXF | TCTAAGAATCTCGGGTAATGAGG | qPCR |
| UTXR | ATAGGCTCAAGAACCCGAAGA | qPCR |
| JMJD3F | ACTGCAACGAATGCGATGTG | qPCR |
| JMJD3R | GGCTGCATTCTCACTTGTAAC | qPCR |
| EZH2F | TGGGAAGAAATCTGAGAAGG | qPCR |
| EZH2R | TGGGTCTGCTACTGTTATTCG | qPCR |
| DicerF | GGGAAATGTGACCCAGACGAA | qPCR |
| DicerR | CAATCCACCACAATCTCACAAGG | qPCR |
| DroshaF | GCAAGAGTATGCCATCACCAA | qPCR |
| DroshaR | CTCAAGTGCGTCCATTGCTG | qPCR |
| Exportin5F | CCACCCAAGTCAGTTTCTACG | qPCR |
| Exportin5R | ACAGGTCCAGTGTCAATAGCAG | qPCR |
| ActinF | CCTGAAGTACCCCATTGAAC | qPCR |
| ActinR | GAGGTCTTTACGGATGTCAAC | qPCR |
| Pri-137F | CAAGAGTTCTTTCTGGTGGTG | qPCR |
| Pri-137R | GAAGATCCAGAACGAAACCA | qPCR |
| Wnt9bF | CCTGCCCTCTTCAACTTTACC | qPCR |
| Wnt9bR | AGCGGCGTTATTGGTCTGTC | qPCR |
| Wnt6F | GGGGTGGATGGGTGAGTTTAG | qPCR |
| Wnt6R | AAGGAGGGATGCGAGGTTTC | qPCR |
| Gsk3bF | CCTGCCCTCTTCAACTTTACC | qPCR |
| Gsk3bR | AGCGGCGTTATTGGTCTGTC | qPCR |
| miR-124-3p | GCTAAGGCACGCGGTG | miRNA profiling |
| miR-137-3p | GCCGGCTTATTGCTTAAGAATAC | miRNA profiling |
| miR-101-3p | GCGCGCTACAGTACTGTGATA | miRNA profiling |
| miR-26b-5p | GCGCCTTCAAGTAATTCAGG | miRNA profiling |
| miR-26a-5p | GCGCTTCAAGTAATCCAGGA | miRNA profiling |
| miR-138-5p | GCCAGCTGGTGTTGTGAATC | miRNA profiling |
| miR-144-3p | GCGCGCCTACAGTATAGATGA | miRNA profiling |
| miR-25-3p | CGCATTGCACTTGTCTCG | miRNA profiling |
| miR-214-3p | CTTGACAGCAGGCACAGAC | miRNA profiling |
| miR-92b-3p | GCTATTGCACTCGTCCCG | miRNA profiling |
| miR-30d-5p | GGCTGTAAACATCCCCGAC | miRNA profiling |
| miRNA-R | GTGCAGGGTCCGAGGT | miRNA profiling |
| U6 snRNAF | CTCGCTTCGGCAGCACA | miRNA profiling |
| U6 snRNAR | AACGCTTCACGAATTTGCGT | miRNA profiling |
| 5S rRNAF | TCTCGTCTGATCTCGGAAGC | miRNA profiling |
| 5S rRNAR | AGCCTACAGCACCCGGTATT | miRNA profiling |
| Wnt9bCF | GCCATCGACCAAGGTGTCA | ChIP |
| Wnt9bCR | AGTCTGGTTCCTCCTCCTGTG | ChIP |
| Wnt6CF | GTCAAACGTCTCCCAGCTAGTC | ChIP |
| Wnt6CR | TTGCCTCCGTAGGGTTGTC | ChIP |
| miR-137CF | TAGGCTGTAGTCGGTGGGAAG | ChIP |
| miR-137CR | CAGGCAGACCAACTCACTCATC | ChIP |
| GAPDHCF | CTCCATTTCCCTGGTTCCTG | ChIP |
| GAPDHCR | TCCAGGACCCAGAAACCAGA | ChIP |

| **Supplementary Dataset**  **Dataset S1. List of genes regulated by H3K27me3 in the absence of Pb** | | |
| --- | --- | --- |
| **Accession** | **Symbol** | **Gene Name** |
| NM_133411 | Abcc4 | multidrug resistance-associated protein 4 |
| NM_001107186 | Abl2 | tyrosine-protein kinase ABL2 |
| NM_022190 | Acan | aggrecan core protein |
| NM_001012013 | Acbd4 | acyl-CoA-binding domain-containing protein 4 |
| NM_001126079 | Acbd7 | acyl-CoA-binding domain-containing protein 7 |
| NM_001106508 | Acoxl | acyl-coenzyme A oxidase-like protein |
| NM_012893 | Actg2 | actin, gamma-enteric smooth muscle |
| NM_001170325 | Actn2 | actinin alpha 2 |
| NM_001039028 | Actr1b | ARP1 actin-related protein 1 homolog B |
| NM_001107239 | Adcy1 | adenylate cyclase type 1 |
| NM_133511 | Adcyap1r1 | pituitary adenylate cyclase-activating |
| NM_001013054 | Adprhl1 | [Protein ADP-ribosylarginine] hydrolase-like |
| NM_001134744 | Agpat5 | 1-acyl-sn-glycerol-3-phosphate acyltransferase |
| NM_001007654 | Agtrap | type-1 angiotensin II receptor-associated |
| NM_030986 | Ak2 | adenylate kinase 2, mitochondrial isoform a |
| NM_001033967 | Ak2 | adenylate kinase 2, mitochondrial isoform b |
| NM_001001801 | Akap7 | A-kinase anchoring protein 18 ,isoform delta |
| NM_053896 | Aldh1a2 | retinal dehydrogenase 2 |
| NM_153301 | Alox15b | arachidonate 15-lipoxygenase B |
| NM_012902 | Amh | muellerian-inhibiting factor precursor |
| NM_001191565 | Ankrd33b | ankyrin repeat domain-containing protein 33B |
| NM_001008523 | Aox4 | aldehyde oxidase 4 |
| NM_031008 | Ap2a2 | AP-2 complex subunit alpha-2 |
| NM_031779 | Apba1 | amyloid beta A4 precursor protein-binding family |
| NM_012779 | Aqp5 | aquaporin-5 |
| NM_024152 | Arf6 | ADP-ribosylation factor 6 |
| NM_001004242 | Arhgap8 | rho GTPase-activating protein 8 |
| NM_001012198 | Arhgap9 | rho GTPase-activating protein 9 isoform 2 |
| NM_001080789 | Arhgap9 | rho GTPase-activating protein 9 isoform 1 |
| NM_001106061 | Arhgef3 | rho guanine nucleotide exchange factor 3 |
| NM_001173981 | Arid3c | AT-rich interactive domain-containing protein |
| NM_001037767 | Arpc5l | actin-related protein 2/3 complex subunit 5-like |
| NM_001047881 | Arsi | arylsulfatase I precursor |
| NM_198735 | Art2b | ADP-ribosyltransferase 2b |
| NM_053397 | Artn | artemin precursor |
| NM_001108420 | Asb13 | ankyrin repeat and SOCS box-containing 13 |
| NM_001106389 | Asf1a | ASF1 anti-silencing function 1 homolog A |
| NM_001035002 | Atad1 | ATPase family AAA domain-containing protein 1 |
| NM_024403 | Atf4 | cyclic AMP-dependent transcription factor ATF-4 |
| NM_012913 | Atp1b3 | sodium/potassium-transporting ATPase subunit |
| NM_023093 | Atp5a1 | ATP synthase subunit alpha, mitochondrial |
| NM_001106068 | B3gnt3 | UDP-GlcNAc:betaGal |
| NM_001107113 | Bach1 | transcription regulator protein BACH1 |
| NM_022300 | Basp1 | brain acid soluble protein 1 |
| NM_001025767 | Blnk | B-cell linker protein |
| NM_001128187 | Bnipl | BCL2/adenovirus E1B 19kD interacting protein |
| NM_001007707 | Brp16 | brain protein 16 |
| NM_001166344 | Btnl4 | butyrophilin subfamily 3 member A2 |
| NM_212489 | Btnl8 | butyrophilin-like 8 |
| NM_001105949 | C1ql2 | complement C1q-like protein 2 |
| NM_001106555 | C8g | complement component C8 gamma chain |
| NM_175595 | Cacna2d3 | voltage-dependent calcium channel subunit |
| NM_053351 | Cacng2 | voltage-dependent calcium channel gamma-2 |
| NM_080694 | Cacng6 | voltage-dependent calcium channel gamma-6 |
| NM_138513 | Calcb | calcitonin gene-related peptide 2 precursor |
| NM_031338 | Camkk2 | calcium/calmodulin-dependent protein kinase |
| NM_019174 | Car4 | carbonic anhydrase 4 precursor |
| NM_001130554 | Card10 | caspase recruitment domain-containing protein |
| NM_001107071 | Cbx2 | chromobox protein homolog 2 |
| NM_199117 | Cbx7 | chromobox protein homolog 7 |
| NM_001014091 | Ccdc33 | coiled-coil domain-containing protein 33 |
| NM_053662 | Ccnl1 | cyclin-L1 |
| NM_001166577 | Cd300e | CMRF35-like molecule 2 |
| NM_017124 | Cd37 | leukocyte antigen CD37 |
| NM_013169 | Cd3d | T-cell surface glycoprotein CD3 delta chain |
| NM_001077646 | Cd3g | T-cell surface glycoprotein CD3 gamma chain |
| NM_134360 | Cd40 | tumor necrosis factor receptor superfamily |
| NM_022269 | Cd55 | decay accelerating factor 1 |
| NM_017125 | Cd63 | CD63 antigen |
| NM_001015016 | Cd72 | B-cell differentiation antigen CD72 |
| NM_001048044 | Cdc42ep3 | CDC42 effector protein (Rho GTPase binding) 3 |
| NM_138889 | Cdh13 | cadherin-13 |
| NM_131902 | Cdkn2c | cyclin-dependent kinase 4 inhibitor C |
| NM_001025682 | Cdr2 | cerebellar degeneration-related protein 2 |
| NM_024125 | Cebpb | CCAAT/enhancer-binding protein beta |
| NM_001100514 | Cep76 | centrosomal protein 76kDa |
| NM_019164 | Chad | chondroadherin precursor |
| NM_001170593 | Chat | choline O-acetyltransferase |
| NM_021655 | Chga | chromogranin-A |
| NM_052805 | Chrna3 | neuronal acetylcholine receptor subunit alpha-3 |
| NM_001106268 | Chsy1 | chondroitin sulfate synthase 1 |
| NM_001107307 | Cilp2 | cartilage intermediate layer protein 2 |
| NM_053327 | Clcnka | chloride channel protein ClC-Ka |
| NM_031702 | Cldn7 | claudin-7 |
| NM_001107501 | Clip3 | CAP-Gly domain-containing linker protein 3 |
| NM_022218 | Cmklr1 | chemokine-like receptor 1 |
| NM_001109300 | Cmtm7 | CKLF-like MARVEL transmembrane domain-containing |
| NM_001108355 | Cnot6l | CCR4-NOT transcription complex subunit 6-like |
| NM_001107236 | Cobl | protein cordon-bleu |
| NM_001108710 | Coch | cochlin |
| NM_001025721 | Colec12 | collectin-12 |
| NM_182473 | Corin | atrial natriuretic peptide-converting enzyme |
| NM_001109327 | Coro1c | coronin-1C |
| NM_001002808 | Cpa5 | carboxypeptidase A5 |
| NM_001105716 | Crabp1 | cellular retinoic acid-binding protein 1 |
| NM_001004085 | Crat | carnitine O-acetyltransferase |
| NM_133381 | Crebbp | CREB-binding protein |
| NM_001024783 | Creld1 | cysteine-rich with EGF-like domain protein 1 |
| NM_022501 | Crip2 | cysteine-rich protein 2 |
| NM_017074 | Cth | cystathionine gamma-lyase |
| NM_181087 | Cyp26b1 | cytochrome P450, family 26, subfamily b, |
| NM_001107495 | Cyp2s1 | cytochrome P450 2S1 |
| NM_031327 | Cyr61 | protein CYR61 precursor |
| NM_001009644 | Dbx1 | homeobox protein DBX1 |
| NM_030993 | Ddn | dendrin |
| NM_001108246 | Ddx3x | ATP-dependent RNA helicase DDX3X |
| NM_031801 | Deaf1 | deformed epidermal autoregulatory factor 1 |
| NM_001029916 | Depdc7 | DEP domain-containing protein 7 |
| NM_181088 | Dfnb31 | whirlin |
| NM_001105832 | Dlx3 | distal-less homeobox 3 |
| NM_012943 | Dlx5 | homeobox protein DLX-5 |
| NM_001173357 | Dmkn | dermokine |
| NM_053706 | Dmrt1 | doublesex- and mab-3-related transcription |
| NM_001107597 | Dmrt2 | doublesex- and mab-3-related transcription |
| NM_053693 | Dmtf1 | cyclin-D-binding Myb-like transcription factor |
| NM_001024342 | Dnai1 | dynein intermediate chain 1, axonemal |
| NM_001108694 | Dnajc11 | dnaJ homolog subfamily C member 11 |
| NM_001014194 | Dnajc16 | dnaJ homolog subfamily C member 16 precursor |
| NM_001130062 | Dok7 | protein Dok-7 |
| NM_012546 | Drd1a | D(1A) dopamine receptor |
| NM_001108141 | Dscaml1 | Down syndrome cell adhesion molecule-like 1 |
| NM_024141 | Duox2 | dual oxidase 2 precursor |
| NM_001107767 | Duoxa1 | dual oxidase maturation factor 1 |
| NM_001191965 | Duoxa2 | dual oxidase maturation factor 2 |
| NM_001007006 | Dusp13 | testis and skeletal muscle-specific dual |
| NM_001037973 | Dusp9 | dual specificity protein phosphatase 9 |
| NM_001172056 | Dvl2 | dishevelled 2 |
| NM_019226 | Dync1h1 | cytoplasmic dynein 1 heavy chain 1 |
| NM_001108506 | Ebf3 | transcription factor COE3 |
| NM_001191076 | Ebf4 | transcription factor COE4 |
| NM_001127541 | Efcab4a | EF-hand calcium-binding domain-containing |
| NM_012842 | Egf | pro-epidermal growth factor precursor |
| NM_053633 | Egr2 | early growth response protein 2 |
| NM_001107602 | Elovl3 | elongation of very long chain fatty acids |
| NM_053927 | Epb4.1l3 | band 4.1-like protein 3 |
| NM_138541 | Epcam | epithelial cell adhesion molecule precursor |
| NM_001105994 | Ephx4 | epoxide hydrolase 4 |
| NM_021687 | Erbb4 | receptor tyrosine-protein kinase erbB-4 |
| NM_022604 | Esm1 | endothelial cell-specific molecule 1 precursor |
| NM_001107423 | Esrp2 | epithelial splicing regulatory protein 2 |
| NM_001108343 | Etnk2 | ethanolamine kinase 2 |
| NM_133537 | Expi | extracellular peptidase inhibitor |
| NM_001109323 | F8a1 | factor VIII intron 22 protein |
| NM_001134834 | Fahd2a | fumarylacetoacetate hydrolase domain-containing |
| NM_001106296 | Fam57b | hypothetical protein LOC293493 |
| NM_001014178 | Fam69b | hypothetical protein LOC362090 |
| NM_001106566 | Fam73b | hypothetical protein LOC296623 |
| NM_001014046 | Fam82a2 | regulator of microtubule dynamics protein 3 |
| NM_001127578 | Fam91a1 | hypothetical protein LOC689997 |
| NM_001108233 | Farp2 | FERM, RhoGEF and pleckstrin domain-containing |
| NM_001025730 | Fbxw5 | F-box/WD repeat-containing protein 5 |
| NM_053843 | Fcgr2a | low affinity immunoglobulin gamma Fc region |
| NM_001100682 | Fcrla | Fc receptor-like A precursor |
| NM_144753 | Fev | protein FEV |
| NM_001109224 | Fezf1 | fez family zinc finger protein 1 |
| NM_130753 | Fgf15 | fibroblast growth factor 15 |
| NM_130752 | Fgf21 | fibroblast growth factor 21 |
| NM_133286 | Fgf8 | fibroblast growth factor 8 |
| NM_001011913 | Fignl1 | fidgetin-like protein 1 |
| NM_001013248 | Foxb1 | forkhead box B1 |
| NM_001168584 | Foxb2 | forkhead box B2 |
| NM_001191846 | Foxo1 | forkhead box protein O1 |
| NM_031236 | Fut1 | galactoside 2-alpha-L-fucosyltransferase 1 |
| NM_022005 | Fxyd6 | FXYD domain-containing ion transport regulator 6 |
| NM_031802 | Gabbr2 | gamma-aminobutyric acid type B receptor subunit |
| NM_001039036 | Gabpb1 | GA repeat binding protein, beta 1 |
| NM_024370 | Gabrg3 | gamma-aminobutyric acid receptor subunit gamma-3 |
| NM_001005888 | Galc | galactocerebrosidase |
| NM_001025053 | Galnt4 | polypeptide N-acetylgalactosaminyltransferase 4 |
| NM_022926 | Galnt7 | N-acetylgalactosaminyltransferase 7 |
| NM_001122644 | Galnt9 | polypeptide N-acetylgalactosaminyltransferase 9 |
| NM_133293 | Gata3 | GATA binding protein 3 |
| NM_144730 | Gata4 | transcription factor GATA-4 |
| NM_019185 | Gata6 | transcription factor GATA-6 |
| NM_053708 | Gbx2 | gastrulation brain homeobox 2 |
| NM_019216 | Gdf15 | growth/differentiation factor 15 precursor |
| NM_017276 | Gdi2 | rab GDP dissociation inhibitor beta |
| NM_001037210 | Gipc2 | PDZ domain-containing protein GIPC2 |
| NM_001004099 | Gjb2 | gap junction beta-2 protein |
| NM_019240 | Gjb3 | gap junction beta-3 protein |
| NM_013133 | Glra1 | glycine receptor subunit alpha-1 |
| NM_001134413 | Gltp | glycolipid transfer protein |
| NM_001107308 | Gmip | GEM-interacting protein |
| NM_001191836 | Gnal | guanine nucleotide-binding protein G(olf) |
| NM_012774 | Gpc3 | glypican-3 precursor |
| NM_001107285 | Gpc5 | glypican 5 precursor |
| NM_001025147 | Gpr160 | probable G-protein coupled receptor 160 |
| NM_001108646 | Gpr162 | probable G-protein coupled receptor 162 |
| NM_001191915 | Gpr50 | melatonin-related receptor |
| NM_001012057 | Gpt2 | alanine aminotransferase 2 |
| NM_019282 | Grem1 | gremlin-1 precursor |
| NM_017010 | Grin1 | glutamate [NMDA] receptor subunit zeta-1 |
| NM_012575 | Grin2c | glutamate [NMDA] receptor subunit epsilon-3 |
| NM_001109270 | Grrp1 | glycine/arginine-rich protein 1 |
| NM_001001512 | Gtf2i | general transcription factor II-I |
| NM_012578 | H1f0 | histone H1.0 |
| NM_022696 | Hand2 | heart- and neural crest derivatives-expressed |
| NM_013064 | Hcrtr1 | orexin receptor type 1 |
| NM_053447 | Hdac2 | histone deacetylase 2 |
| NM_001108631 | Herc3 | probable E3 ubiquitin-protein ligase HERC3 |
| NM_019236 | Hes2 | transcription factor HES-2 |
| NM_022528 | Hif3a | hypoxia-inducible factor 3-alpha |
| NM_017268 | Hmgcs1 | hydroxymethylglutaryl-CoA synthase, cytoplasmic |
| NM_001106303 | Hmx2 | homeobox protein HMX2 |
| NM_001129878 | Hoxa10 | homeo box A10 |
| NM_001191087 | Hoxa6 | homeobox protein Hox-A6 |
| NM_001109233 | Hoxa9 | homeobox protein Hox-A7 |
| NM_001107042 | Hoxb3 | homeo box B3 |
| NM_001100787 | Hoxb4 | homeo box B4 |
| NM_001191925 | Hoxb5 | homeo box B5 |
| NM_001017480 | Hoxb7 | homeobox protein Hox-B7 |
| NM_001100497 | Hoxb9 | homeo box B9 |
| NM_001106796 | Hoxc12 | homeobox protein Hox-C12 |
| NM_001105884 | Hoxd1 | homeobox protein Hox-D1 |
| NM_001107094 | Hoxd10 | homeo box D10 |
| NM_017122 | Hpca | neuron-specific calcium-binding protein |
| NM_001135762 | Hpse2 | heparanase-2 |
| NM_181370 | Hs3st2 | heparan sulfate glucosamine 3-O-sulfotransferase |
| NM_139329 | Hsd3b7 | 3 beta-hydroxysteroid dehydrogenase type 7 |
| NM_001106177 | Hsf4 | heat shock factor protein 4 |
| NM_053612 | Hspb8 | heat shock protein beta-8 |
| NM_024395 | Htr5b | 5-hydroxytryptamine receptor 5B |
| NM_022938 | Htr7 | 5-hydroxytryptamine receptor 7 |
| NM_031721 | Htra1 | serine protease HTRA1 |
| NM_001107321 | Htra4 | probable serine protease HTRA4 |
| NM_013159 | Ide | insulin-degrading enzyme |
| NM_001082477 | Igf1 | insulin-like growth factor I isoform a |
| NM_001190163 | Igf2 | insulin-like growth factor II isoform 2 |
| NM_031511 | Igf2 | insulin-like growth factor II isoform 1 |
| NM_001107197 | Igsf9 | protein turtle homolog A precursor |
| NM_001107237 | Ikzf1 | IKAROS family zinc finger 1 |
| NM_001107521 | Il20ra | interleukin-20 receptor subunit alpha |
| NM_133409 | Ilk | integrin-linked protein kinase |
| NM_172224 | Impa2 | inositol monophosphatase 2 |
| NM_134417 | Ipmk | inositol polyphosphate multikinase |
| NM_207617 | Iqsec3 | IQ motif and SEC7 domain-containing protein 3 |
| NM_001013880 | Isyna1 | inositol-3-phosphate synthase 1 |
| NM_001014116 | Jmjd8 | jmjC domain-containing protein 8 |
| NM_138875 | Jund | transcription factor jun-D |
| NM_001008814 | Kb21 | keratin, type II cuticular Hb1 |
| NM_173095 | Kcna1 | potassium voltage-gated channel subfamily A |
| NM_053630 | Kcnh4 | potassium voltage-gated channel subfamily H |
| NM_145095 | Kcnh8 | potassium voltage-gated channel subfamily H |
| NM_130813 | Kcnk15 | potassium channel subfamily K member 15 |
| NM_001039516 | Kcnk5 | potassium channel subfamily K member 5 |
| NM_053806 | Kcnk6 | potassium channel, subfamily K, member 6 |
| NM_023021 | Kcnn4 | intermediate conductance calcium-activated |
| NM_057202 | Kif5b | kinesin-1 heavy chain |
| NM_001048215 | Kirrel3 | kin of IRRE-like protein 3 |
| NM_023992 | Kiss1r | kiSS-1 receptor |
| NM_022264 | Kit | mast/stem cell growth factor receptor |
| NM_001107164 | Klf1 | Krueppel-like factor 1 |
| NM_001106054 | Klhl1 | kelch-like protein 1 |
| NM_001106252 | Klk11 | kallikrein-11 |
| NM_017063 | Kpnb1 | importin subunit beta-1 |
| NM_001109326 | Krtap14 | keratin-associated protein 14 |
| NM_053538 | Laptm5 | lysosomal-associated transmembrane protein 5 |
| NM_001007556 | Lefty2 | left-right determination factor 2 |
| NM_001106784 | Lgr5 | leucine-rich repeat-containing G-protein coupled |
| NM_139036 | Lhx5 | LIM/homeobox protein Lhx5 |
| NM_001107837 | Lhx6 | LIM/homeobox protein Lhx6 |
| NM_001100722 | Lingo1 | leucine rich repeat and Ig domain containing 1 |
| NM_001143803 | LOC100233213 | hypothetical protein LOC100233213 |
| NM_001177829 | LOC100365935 | hypothetical protein LOC100365935 |
| NM_001013941 | LOC298795 | hypothetical protein LOC298795 |
| NM_001013979 | LOC304131 | TAK1-like protein |
| NM_001135992 | LOC498276 | Fc gamma receptor II beta |
| NM_001109221 | LOC500034 | hypothetical protein LOC500034 |
| NM_001162931 | LOC502128 | POM121 membrane glycoprotein-like 2 isoform 2 |
| NM_001162930 | LOC502128 | POM121 membrane glycoprotein-like 2 isoform 1 |
| NM_001195277 | LOC679651 | transmembrane protein 178-like |
| NM_001109418 | LOC680531 | hypothetical protein LOC680531 |
| NM_001109489 | LOC685964 | hypothetical protein LOC685964 |
| NM_001163002 | LOC689926 | hypothetical protein LOC689926 |
| NM_001109595 | LOC690478 | hypothetical protein LOC690478 |
| NM_001109616 | LOC691024 | hypothetical protein LOC691024 |
| NM_001170434 | Lrrc32 | leucine rich repeat containing 32 |
| NM_021656 | Ltb4r | leukotriene B4 receptor 1 |
| NM_001109391 | Mab21l2 | protein mab-21-like 2 |
| NM_019318 | Maf | transcription factor Maf |
| NM_138503 | Map3k2 | mitogen-activated protein kinase kinase kinase |
| NM_001198638 | Map7 | ensconsin isoform 2 |
| NM_017212 | Mapt | microtubule-associated protein tau |
| NM_001107590 | Marveld1 | MARVEL domain-containing protein 1 |
| NM_001109132 | Marveld3 | MARVEL domain-containing protein 3 |
| NM_181089 | MAST1 | microtubule-associated serine/threonine-protein |
| NM_001108013 | Matn3 | matrilin-3 |
| NM_001025289 | Mbp | Golli-Mbp isoform 1 |
| NM_001039005 | Mcoln2 | mucolipin-2 |
| NM_030859 | Mdk | midkine precursor |
| NM_001108837 | Meox1 | homeobox protein MOX-1 |
| NM_017149 | Meox2 | homeobox protein MOX-2 |
| NM_022943 | Mertk | tyrosine-protein kinase Mer precursor |
| NM_001107531 | Mesp1 | mesoderm posterior protein 1 |
| NM_001008518 | MGC105649 | normal mucosa of esophagus-specific gene 1 |
| NM_001024890 | MGC114520 | hypothetical protein LOC315915 |
| NM_001191889 | Mid2 | midline-2 |
| NM_001108737 | Mier2 | mesoderm induction early response protein 2 |
| NR_031814 | Mir10a |  |
| NR_031865 | Mir124-3 |  |
| NR_031878 | Mir132 |  |
| NR_031883 | Mir137 |  |
| NR_031897 | Mir181c |  |
| NR_032266 | Mir181d |  |
| NR_031909 | Mir193 |  |
| NR_031925 | Mir212 |  |
| NR_031850 | Mir34a |  |
| NM_020102 | Mos | proto-oncogene serine/threonine-protein kinase |
| NM_001034022 | Mprip | myosin phosphatase Rho-interacting protein |
| NM_022529 | Mrpl23 | 39S ribosomal protein L23, mitochondrial |
| NM_001108635 | Mrpl53 | 39S ribosomal protein L53, mitochondrial |
| NM_001106628 | Mrps35 | 28S ribosomal protein S35, mitochondrial |
| NM_053712 | Msx3 | homeo box, msh-like 3 |
| NM_001100833 | Mtch1 | mitochondrial carrier homolog 1 |
| NM_001191558 | Mtss1l | MTSS1-like protein |
| NM_001106257 | Mybpc2 | myosin-binding protein C, fast-type |
| NM_057209 | Mylk2 | myosin light chain kinase 2, skeletal/cardiac |
| NM_053888 | Myt1l | myelin transcription factor 1-like protein |
| NM_001013059 | Ndfip1 | NEDD4 family-interacting protein 1 |
| NM_017029 | Nefm | neurofilament medium polypeptide |
| NM_001013134 | Nek4 | serine/threonine-protein kinase Nek4 |
| NM_001002851 | Nenf | neudesin precursor |
| NM_012865 | Nfya | nuclear transcription factor Y subunit alpha |
| NM_012610 | Ngfr | tumor necrosis factor receptor superfamily |
| NM_001191733 | Nhs | Nance-Horan syndrome protein |
| NM_001170476 | Nkx1-2 | NK1 homeobox 2 |
| NM_013093 | Nkx2-1 | homeobox protein Nkx-2.1 |
| NM_001107594 | Nkx2-3 | homeobox protein Nkx-2.3 |
| NM_053651 | Nkx2-5 | homeobox protein Nkx-2.5 |
| NM_134336 | Nlgn3 | neuroligin-3 precursor |
| NM_001105721 | Notch1 | neurogenic locus notch homolog protein 1 |
| NM_153293 | Npb | neuropeptide B precursor |
| NM_203340 | Npm2 | nucleoplasmin-2 |
| NM_019380 | Nptn | neuroplastin |
| NM_031628 | Nr4a3 | nuclear receptor subfamily 4 group A member 3 |
| NM_024140 | Nrgn | neurogranin |
| NM_001107337 | Nsd1 | histone-lysine N-methyltransferase, H3 lysine-36 |
| NM_001017452 | Nsun7 | NOL1/NOP2/Sun domain family, member 7 |
| NM_001106465 | Ntng1 | netrin-G1 |
| NM_001011891 | Nubp2 | cytosolic Fe-S cluster assembly factor NUBP2 |
| NM_181363 | Nudt6 | nucleoside diphosphate-linked moiety X motif 6 |
| NM_021680 | Nxph4 | neurexophilin-4 precursor |
| NM_001106269 | Olig3 | oligodendrocyte transcription factor 2 |
| NM_001107848 | Ophn1 | oligophrenin-1 |
| NM_001014024 | Orai3 | protein orai-3 |
| NM_001107565 | Oraov1 | oral cancer overexpressed 1 |
| NM_001012118 | Osr2 | protein odd-skipped-related 2 |
| NM_012871 | Oxtr | oxytocin receptor |
| NM_134353 | Pabpc1 | polyadenylate-binding protein 1 |
| NM_017230 | Padi3 | protein-arginine deiminase type-3 |
| NM_133531 | Pank4 | pantothenate kinase 4 |
| NM_001191077 | Paqr6 | progestin and adipoQ receptor family member 6 |
| NM_001035249 | Parl | presenilins-associated rhomboid-like protein, |
| NM_001107787 | Pax1 | paired box protein Pax-1 |
| NM_053710 | Pax3 | paired box 3 |
| NM_001039539 | Pax9 | paired box protein Pax-9 |
| NM_001169129 | Pcdh19 | protocadherin-19 |
| NM_001129882 | Pcgf5 | polycomb group RING finger protein 5 |
| NM_001100506 | Pctk3 | cell division protein kinase 18 |
| NM_001009542 | Pdcd10 | programmed cell death protein 10 |
| NM_031317 | Pdgfc | platelet-derived growth factor C |
| NM_012802 | Pdgfra | alpha-type platelet-derived growth factor |
| NM_001004072 | Pdha1 | pyruvate dehydrogenase E1 component subunit |
| NM_053826 | Pdk1 | [Pyruvate dehydrogenase [lipoamide]] kinase |
| NM_019374 | Pdyn | proenkephalin-B preproprotein |
| NM_130401 | Pdzk1ip1 | PDZK1-interacting protein 1 |
| NM_001109487 | Pfn3 | profilin-3 |
| NM_001106198 | Pgbd5 | piggyBac transposable element-derived protein 5 |
| NM_031784 | Pias3 | E3 SUMO-protein ligase PIAS3 |
| NM_001105951 | Pik3c2b | phosphatidylinositol-4-phosphate 3-kinase C2 |
| NM_022602 | Pim3 | serine/threonine-protein kinase pim-3 |
| NM_001105845 | Plcd3 | 1-phosphatidylinositol-4,5-bisphosphate |
| NM_053758 | Plce1 | 1-phosphatidylinositol-4,5-bisphosphate |
| NM_001134972 | Plekhg2 | pleckstrin homology domain-containing family G |
| NM_001108036 | Plekhh1 | pleckstrin homology domain containing, family H |
| NM_022533 | Pllp | plasmolipin |
| NM_172085 | Pou3f2 | POU domain, class 3, transcription factor 2 |
| NM_001108889 | Pou4f3 | POU class 4 homeobox 3 |
| NM_022538 | Ppap2a | lipid phosphate phosphohydrolase 1 |
| NM_013196 | Ppara | peroxisome proliferator-activated receptor |
| NM_001105968 | Ppox | protoporphyrinogen oxidase |
| NM_144746 | Ppp2r2d | serine/threonine-protein phosphatase 2A 55 kDa |
| NM_001108577 | Ppp2r4 | serine/threonine-protein phosphatase 2A |
| NM_001106613 | Ppp4r2 | protein phosphatase 4, regulatory subunit 2 |
| NM_134449 | Prkcdbp | protein kinase C delta-binding protein |
| NM_001033963 | Prkx | serine/threonine-protein kinase PRKX |
| NM_001038588 | Prodh2 | probable proline dehydrogenase 2 |
| NM_001024305 | Prpf38b | pre-mRNA-splicing factor 38B |
| NM_001109027 | Prss33 | serine protease 33 |
| NM_001107395 | Psd2 | PH and SEC7 domain-containing protein 2 |
| NM_019126 | Psg19 | carcinoembryonic antigen gene family (CGM3) |
| NM_130430 | Psmd9 | 26S proteasome non-ATPase regulatory subunit 9 |
| NM_001106138 | Psmg2 | tumor necrosis factor superfamily, member |
| NM_022516 | Ptbp1 | polypyrimidine tract-binding protein 1 isoform |
| NM_053964 | Ptf1a | pancreas transcription factor 1 subunit alpha |
| NM_001108507 | Pwwp2b | PWWP domain-containing protein 2B |
| NM_001108962 | R3hdml | R3H domain (binds single-stranded nucleic acids) |
| NM_001109005 | Rab23 | ras-related protein Rab-23 |
| NM_031718 | Rab2a | ras-related protein Rab-2A |
| NM_053741 | Rap2a | RAS related protein 2a |
| NM_001108273 | Rasgef1c | ras-GEF domain-containing family member 1C |
| NM_001170531 | Rasgrf1 | ras-specific guanine nucleotide-releasing factor |
| NM_001105753 | Rasgrf1 | ras-specific guanine nucleotide-releasing factor |
| NM_001106261 | Rasip1 | ras-interacting protein 1 |
| NM_001106317 | Rassf7 | ras association domain-containing protein 7 |
| NM_053678 | Rax | retinal homeobox protein Rx |
| NM_013162 | Rbp4 | retinol-binding protein 4 precursor |
| NM_001127490 | Rfx7 | regulatory factor X domain containing 2 |
| NM_001004268 | RGD1303271 | hypothetical protein LOC313018 |
| NM_001134560 | RGD1305627 | hypothetical protein LOC314467 |
| NM_001106551 | RGD1306208 | hypothetical protein LOC296483 |
| NM_001107663 | RGD1307225 | hypothetical protein LOC310269 |
| NM_001134596 | RGD1308299 | hypothetical protein LOC367214 |
| NM_001108129 | RGD1309188 | hypothetical protein LOC315463 |
| NM_001107161 | RGD1310262 | hypothetical protein LOC304650 |
| NM_001079705 | RGD1311558 | shootin-1 |
| NM_001127526 | RGD1311605 | hypothetical protein LOC298841 |
| NM_001109262 | RGD1559493 | hypothetical protein LOC500516 |
| NM_001108678 | RGD1559909 | hypothetical protein LOC362592 |
| NM_001106014 | RGD1560394 | hypothetical protein LOC289728 |
| NM_001109345 | RGD1563349 | hypothetical protein LOC502727 |
| NM_001109311 | RGD1563692 | hypothetical protein LOC501185 |
| NM_001109234 | RGD1564419 | hypothetical protein LOC500128 |
| NM_001109292 | RGD1564560 | hypothetical protein LOC500988 |
| NM_001109067 | RGD1565883 | hypothetical protein LOC498193 |
| NM_001134589 | RGD1566265 | hypothetical protein LOC363487 |
| NM_001013133 | Rhobtb2 | rho-related BTB domain-containing protein 2 |
| NM_001100488 | Rimbp2 | RIMS-binding protein 2 |
| NM_053945 | Rims2 | regulating synaptic membrane exocytosis protein |
| NM_001106836 | Rnf111 | E3 ubiquitin-protein ligase Arkadia |
| NM_001173349 | Rnf128 | E3 ubiquitin-protein ligase RNF128 |
| NM_001191093 | Rnf150 | RING finger protein 150 |
| NM_053338 | Rrad | GTP-binding protein RAD |
| NM_001048184 | Rragc | ras-related GTP-binding protein C |
| NM_001106641 | Rragd | ras-related GTP-binding protein D |
| NM_001025740 | Rrm2 | ribonucleoside-diphosphate reductase subunit M2 |
| NM_001008346 | Rrp8 | ribosomal RNA-processing protein 8 |
| NM_001008827 | RT1-A1 | RT1 class Ia, locus A1 |
| NM_001008832 | RT1-CE1 | RT1 class I, locus CE1 |
| NM_001008833 | RT1-CE10 | RT1 class I, locus CE10 |
| NM_001033985 | RT1-CE14 | RT1 class I, locus CE14 isoform 2 |
| NM_001008840 | RT1-CE2 | RT1 class I, locus CE2 |
| NM_012645 | RT1-EC2 | class I histocompatibility antigen, Non-RT1.A |
| NM_001008848 | RT1-Ha | RT1 class II, locus Ha |
| NM_181380 | Rtn4rl2 | reticulon-4 receptor-like 2 precursor |
| NM_001109471 | S100a7a | protein S100-A15A |
| NM_022394 | Safb | scaffold attachment factor B1 |
| NM_001013985 | Sccpdh | probable saccharopine dehydrogenase |
| NM_198748 | Scin | adseverin |
| NM_017247 | Scn10a | sodium channel protein type 10 subunit alpha |
| NM_001008880 | Scn4b | sodium channel subunit beta-4 precursor |
| NM_012648 | Scnn1b | amiloride-sensitive sodium channel subunit beta |
| NM_022670 | Sct | secretin precursor |
| NM_177929 | Sdccag8 | serologically defined colon cancer antigen 8 |
| NM_001107637 | Sec63 | translocation protein SEC63 homolog |
| NM_001166396 | Selv | selenoprotein V |
| NM_017308 | Sema6c | semaphorin-6C precursor |
| NM_001173429 | Sept6 | septin-6 |
| NM_022616 | Sept7 | septin-7 isoform a |
| NM_001109104 | Serp2 | stress-associated endoplasmic reticulum protein |
| NM_001008776 | Serpina11 | serpin A11 isoform 1 |
| NM_053779 | Serpini1 | neuroserpin precursor |
| NM_031647 | Sfmbt1 | scm-like with four MBT domains protein 1 |
| NM_001105937 | Sgsm1 | small G protein signaling modulator 1 |
| NM_053360 | Sh3kbp1 | SH3 domain-containing kinase-binding protein 1 |
| NM_134457 | Siah2 | E3 ubiquitin-protein ligase SIAH2 |
| NM_021693 | Sik1 | serine/threonine-protein kinase SIK1 |
| NM_001107641 | Sim1 | single-minded homolog 1 |
| NM_001004089 | Sipa1 | signal-induced proliferation-associated protein |
| NM_053759 | Six1 | sine oculis-related homeobox 1 homolog |
| NM_023990 | Six3 | homeobox protein SIX3 |
| NM_031798 | Slc12a2 | solute carrier family 12 member 2 |
| NM_134363 | Slc12a5 | solute carrier family 12 member 5 |
| NM_153625 | Slc12a8 | solute carrier family 12 member 8 |
| NM_147216 | Slc16a2 | monocarboxylate transporter 8 |
| NM_053427 | Slc17a6 | vesicular glutamate transporter 2 |
| NM_031663 | Slc18a3 | vesicular acetylcholine transporter |
| NM_001106327 | Slc22a20 | solute carrier family 22 member 20 |
| NM_019230 | Slc22a3 | solute carrier family 22 member 3 |
| NM_017316 | Slc23a2 | solute carrier family 23 member 2 |
| NM_019214 | Slc26a4 | pendrin |
| NM_031736 | Slc27a2 | very long-chain acyl-CoA synthetase |
| NM_133600 | Slc31a1 | high affinity copper uptake protein 1 |
| NM_001107522 | Slc35d3 | solute carrier family 35 member D3 |
| NM_001105950 | Slc35f5 | solute carrier family 35 member F5 |
| NM_001191920 | Slc47a2 | multidrug and toxin extrusion protein 2 |
| NM_130746 | Slc5a6 | sodium-dependent multivitamin transporter |
| NM_203334 | Slc6a5 | sodium- and chloride-dependent glycine |
| NM_017206 | Slc6a6 | sodium- and chloride-dependent taurine |
| NM_078620 | Slc8a3 | sodium/calcium exchanger 3 precursor |
| NM_001113335 | Slc9a2 | sodium/hydrogen exchanger 2 isoform 1 |
| NM_022667 | Slco2a1 | solute carrier organic anion transporter family |
| NM_022953 | Slit1 | slit homolog 1 protein precursor |
| NM_030858 | Smad7 | mothers against decapentaplegic homolog 7 |
| NM_206851 | Smyd2 | SET and MYND domain-containing protein 2 |
| NM_001191563 | Sorcs1 | VPS10 domain-containing receptor SorCS1 |
| NM_001106367 | Sorcs3 | VPS10 domain-containing receptor SorCS3 |
| NM_019193 | Sox10 | transcription factor SOX-10 |
| NM_001106850 | Sox14 | SRY (sex determining region Y)-box 14 |
| NM_001107902 | Sox17 | transcription factor SOX-17 |
| NM_001106530 | Spag4l | SUN domain-containing protein 5 |
| NM_001106125 | Spag6l | sperm associated antigen 6-like |
| NM_199374 | Spata18 | spermatogenesis-associated protein 18 |
| NM_001108549 | Spata5 | spermatogenesis-associated protein 5 |
| NM_181388 | Spg7 | paraplegin |
| NM_133386 | Sphk1 | sphingosine kinase 1 |
| NM_001039208 | Spns1 | protein spinster homolog 1 |
| NM_172067 | Spon1 | spondin-1 precursor |
| NM_001106988 | Spsb3 | SPRY domain-containing SOCS box protein 3 |
| NM_001135711 | Srrp | 35 kDa SR repressor protein |
| NM_012659 | Sst | somatostatin precursor |
| NM_175597 | Ssx2ip | afadin- and alpha-actinin-binding protein |
| NM_031704 | Stx5 | syntaxin-5 |
| NM_031665 | Stx6 | syntaxin-6 |
| NM_001100750 | Suclg2 | succinyl-CoA ligase [GDP-forming] subunit beta, |
| NM_001025125 | Sumf2 | sulfatase-modifying factor 2 |
| NM_001107341 | Susd3 | sushi domain-containing protein 3 |
| NM_022191 | Syt6 | synaptotagmin-6 |
| NM_001025419 | Tax1bp3 | tax1-binding protein 3 |
| NM_001013245 | Tbca | tubulin-specific chaperone A |
| NM_001191070 | Tbr1 | T-box brain protein 1 |
| NM_001108322 | Tbx1 | T-box transcription factor TBX1 |
| NM_001108132 | Tbx20 | T-box 20 |
| NM_181638 | Tbx3 | T-box transcription factor TBX3 |
| NM_001107034 | Tbx4 | T-box transcription factor TBX4 |
| NM_001009964 | Tbx5 | T-box transcription factor TBX5 |
| NM_001032397 | Tcf21 | transcription factor 21 |
| NM_001106896 | Tcfap2b | transcription factor AP-2-beta |
| NM_201420 | Tcfap2c | transcription factor AP-2 gamma |
| NM_001098216 | Tead3 | TEA domain family member 3 |
| NM_201655 | Tepp | testis, prostate and placenta-expressed protein |
| NM_012671 | Tgfa | protransforming growth factor alpha |
| NM_001191840 | Tgfb1i1 | transforming growth factor beta-1-induced |
| NM_031131 | Tgfb2 | transforming growth factor beta-2 precursor |
| NM_019386 | Tgm2 | protein-glutamine gamma-glutamyltransferase 2 |
| NM_001100558 | Tiam1 | T-cell lymphoma invasion and metastasis 1 |
| NM_001172125 | Tlx2 | T-cell leukemia, homeobox 2 |
| NM_001107015 | Tm4sf5 | transmembrane 4 L6 family member 5 |
| NM_001108795 | Tmeff2 | tomoregulin-2 |
| NM_001159625 | Tmem116 | transmembrane protein 116 |
| NM_001106280 | Tmem126b | transmembrane protein 126B |
| NM_001107476 | Tmem150b | transmembrane protein 150B |
| NM_001191668 | Tmem185b | transmembrane protein 185B |
| NM_001108045 | Tmem63c | transmembrane protein 63C |
| NM_001017455 | Tmem80 | transmembrane protein 80 |
| NM_001105806 | Tmem93 | transmembrane protein 93 |
| NM_001127528 | Tmprss13 | transmembrane protease serine 13 |
| NM_001108998 | Tmprss4 | transmembrane protease serine 4 |
| NM_153311 | Tmprss5 | transmembrane protease serine 5 |
| NM_001108873 | Tnfrsf10b | tumor necrosis factor receptor superfamily, |
| NM_001191810 | Tns1 | tensin 1 |
| NR_024118 | Tnxa |  |
| NM_019180 | Tpsb2 | tryptase beta-2 precursor |
| NM_013046 | Trh | prothyroliberin |
| NM_130420 | Trim9 | E3 ubiquitin-protein ligase TRIM9 |
| NM_001134837 | Trps1 | zinc finger transcription factor Trps1 |
| NM_199088 | Tsks | testis-specific serine kinase substrate |
| NM_001109227 | Tspan33 | tetraspanin-33 |
| NM_001108815 | Tspan7 | tetraspanin-7 |
| NM_012808 | Tst | thiosulfate sulfurtransferase |
| NM_001109119 | Tubb2a | tubulin beta-2A chain |
| NM_001025675 | Tubb6 | tubulin, beta 6 |
| NM_001039163 | Tusc5 | tumor suppressor candidate 5 homolog |
| NM_001105723 | Ubtf | nucleolar transcription factor 1 isoform 1 |
| NM_001077660 | Urg4 | up-regulated gene 4 |
| NM_022637 | Vax2 | ventral anterior homeobox 2 |
| NM_001109546 | Vsx1 | visual system homeobox 1 |
| NM_001169128 | Vsx2 | visual system homeobox 2 |
| NM_001109312 | Vwc2 | brorin |
| NM_053751 | Wap | whey acidic protein precursor |
| NM_001135894 | Wdr25l | WD repeat domain 25-like |
| NM_001110489 | Wdr86 | WD repeat-containing protein 86 |
| NM_031716 | Wisp1 | WNT1-inducible-signaling pathway protein 1 |
| NM_001191556 | Wnk2 | serine/threonine-protein kinase WNK2 |
| NM_175579 | Wnk4 | serine/threonine-protein kinase WNK4 |
| NM_001108227 | Wnt10a | protein Wnt-10a |
| NM_001191848 | Wnt2b | protein Wnt-2b |
| NM_001105783 | Wnt9a | protein Wnt-9a |
| NM_001107055 | Wnt9b | protein Wnt-9b |
| NM_001106184 | Wwp2 | NEDD4-like E3 ubiquitin-protein ligase WWP2 |
| NM_199383 | Yipf1 | protein YIPF1 |
| NM_001014208 | Yipf2 | protein YIPF2 |
| NM_001025747 | Yipf6 | protein YIPF6 |
| NM_175604 | Yrdc | yrdC domain-containing protein, mitochondrial |
| NM_019377 | Ywhab | 14-3-3 protein beta/alpha |
| NM_001130537 | Zbtb39 | zinc finger and BTB domain-containing protein |
| NM_001170577 | Zfp167 | zinc finger protein 167 |
| NM_001135088 | Zfp385a | zinc finger protein 385A isoform 1 |
| NM_001135089 | Zfp385a | zinc finger protein 385A isoform 3 |
| NM_001109470 | Zfp385a | zinc finger protein 385A isoform 2 |
| NM_001108725 | Zfyve21 | zinc finger FYVE domain-containing protein 21 |
| NM_203369 | Zmynd11 | zinc finger MYND domain-containing protein 11 |
| NM_001030038 | Znf518a | zinc finger protein 518A |
| NM_001024878 | Znrf4 | zinc/RING finger protein 4 |
| NM_031616 | Zranb2 | zinc finger Ran-binding domain-containing |

**Dataset S2 List of genes regulated by H3K27me3 upon Pb exposure**

| **Accession** | **Symbol** | **GeneName** |
| --- | --- | --- |
| NM_012690 | Abcb4 | multidrug resistance protein 2 |
| NM_080582 | Abcb6 | ATP-binding cassette sub-family B member 6, |
| NM_133411 | Abcc4 | multidrug resistance-associated protein 4 |
| NM_001014133 | Abcg3l2 | ATP-binding cassette, sub-family G (WHITE), |
| NM_001107186 | Abl2 | tyrosine-protein kinase ABL2 |
| NM_001105814 | Abr | active breakpoint cluster region-related |
| NM_001005902 | Abtb1 | ankyrin repeat and BTB/POZ domain-containing |
| NM_022190 | Acan | aggrecan core protein |
| NM_001012013 | Acbd4 | acyl-CoA-binding domain-containing protein 4 |
| NM_001170325 | Actn2 | actinin alpha 2 |
| NM_001039028 | Actr1b | ARP1 actin-related protein 1 homolog B |
| NM_031554 | Acvr2b | activin receptor type-2B |
| NM_001108433 | Adamts19 | A disintegrin and metalloproteinase with |
| NM_198761 | Adamts5 | A disintegrin and metalloproteinase with |
| NM_001111057 | Adarb1 | double-stranded RNA-specific editase 1 isoform |
| NM_001107239 | Adcy1 | adenylate cyclase type 1 |
| NM_138506 | Adra2c | alpha-2C adrenergic receptor |
| NM_001134744 | Agpat5 | 1-acyl-sn-glycerol-3-phosphate acyltransferase |
| NM_001007654 | Agtrap | type-1 angiotensin II receptor-associated |
| NM_001134956 | Ahdc1 | A.T hook DNA-binding motif-containing protein 1 |
| NM_001001801 | Akap7 | A-kinase anchoring protein 18 ,isoform delta |
| NM_053896 | Aldh1a2 | retinal dehydrogenase 2 |
| NM_013059 | Alpl | alkaline phosphatase, tissue-nonspecific isozyme |
| NM_012902 | Amh | muellerian-inhibiting factor precursor |
| NM_001191565 | Ankrd33b | ankyrin repeat domain-containing protein 33B |
| NM_001134699 | Ankrd40 | ankyrin repeat domain-containing protein 40 |
| NM_001009676 | Anks3 | ankyrin repeat and SAM domain-containing protein |
| NM_001108331 | Ap1s1 | adaptor protein complex AP-1, sigma 1 |
| NM_031008 | Ap2a2 | AP-2 complex subunit alpha-2 |
| NM_031779 | Apba1 | amyloid beta A4 precursor protein-binding family |
| NM_012500 | Apeh | acylamino-acid-releasing enzyme |
| NM_012778 | Aqp1 | aquaporin-1 |
| NM_173105 | Aqp11 | aquaporin-11 |
| NM_012779 | Aqp5 | aquaporin-5 |
| NM_024152 | Arf6 | ADP-ribosylation factor 6 |
| NM_001106061 | Arhgef3 | rho guanine nucleotide exchange factor 3 |
| NM_001173981 | Arid3c | AT-rich interactive domain-containing protein |
| NM_001013108 | Arih1 | ariadne ubiquitin-conjugating enzyme E2 binding |
| NM_001024906 | Arl2bp | ADP-ribosylation factor-like protein 2-binding |
| NM_001106919 | Arpc2 | actin-related protein 2/3 complex subunit 2 |
| NM_001106615 | Arpc4 | actin-related protein 2/3 complex subunit 4 |
| NM_001037767 | Arpc5l | actin-related protein 2/3 complex subunit 5-like |
| NM_001108420 | Asb13 | ankyrin repeat and SOCS box-containing 13 |
| NM_001106389 | Asf1a | ASF1 anti-silencing function 1 homolog A |
| NM_001035002 | Atad1 | ATPase family AAA domain-containing protein 1 |
| NM_024403 | Atf4 | cyclic AMP-dependent transcription factor ATF-4 |
| NM_012913 | Atp1b3 | sodium/potassium-transporting ATPase subunit |
| NM_012914 | Atp2a3 | sarcoplasmic/endoplasmic reticulum calcium |
| NM_023093 | Atp5a1 | ATP synthase subunit alpha, mitochondrial |
| NM_001106068 | B3gnt3 | UDP-GlcNAc:betaGal |
| NM_001012018 | B4galt4 | beta-1,4-galactosyltransferase 4 |
| NM_001107113 | Bach1 | transcription regulator protein BACH1 |
| NM_001106191 | Banp | protein BANP |
| NM_022300 | Basp1 | brain acid soluble protein 1 |
| NM_001191586 | Bcor | BCL6 co-repressor |
| NM_031555 | Bfsp1 | filensin |
| NM_001009604 | Bri3 | brain protein I3 |
| NM_001007707 | Brp16 | brain protein 16 |
| NM_017259 | Btg2 | protein BTG2 |
| NM_001008524 | C1qc | complement C1q subcomponent subunit C precursor |
| NM_001108838 | C1ql1 | C1q-related factor |
| NM_001106555 | C8g | complement component C8 gamma chain |
| NM_001107404 | Cables1 | CDK5 and ABL1 enzyme substrate 1 |
| NM_175595 | Cacna2d3 | voltage-dependent calcium channel subunit |
| NM_012518 | Calm3 | calmodulin |
| NM_031338 | Camkk2 | calcium/calmodulin-dependent protein kinase |
| NM_001168549 | Camsap1 | calmodulin regulated spectrin-associated protein |
| NM_001013191 | Cbfb | core-binding factor subunit beta |
| NM_001107071 | Cbx2 | chromobox protein homolog 2 |
| NM_001191667 | Ccdc64 | bicaudal D-related protein 1 |
| NM_001105725 | Ccng2 | cyclin-G2 |
| NM_053662 | Ccnl1 | cyclin-L1 |
| NM_022269 | Cd55 | decay accelerating factor 1 |
| NM_001013103 | Cdc34 | ubiquitin-conjugating enzyme Cdc34 |
| NM_053743 | Cdc37 | hsp90 co-chaperone Cdc37 |
| NM_053620 | Cdc42bpb | serine/threonine-protein kinase MRCK beta |
| NM_001048044 | Cdc42ep3 | CDC42 effector protein (Rho GTPase binding) 3 |
| NM_053891 | Cdk5r1 | cyclin-dependent kinase 5 activator 1 |
| NM_131902 | Cdkn2c | cyclin-dependent kinase 4 inhibitor C |
| NM_001025682 | Cdr2 | cerebellar degeneration-related protein 2 |
| NM_024125 | Cebpb | CCAAT/enhancer-binding protein beta |
| NM_012831 | Cebpg | CCAAT/enhancer-binding protein gamma |
| NM_001100514 | Cep76 | centrosomal protein 76kDa |
| NM_001105900 | Cggbp1 | CGG triplet repeat-binding protein 1 |
| NM_019164 | Chad | chondroadherin precursor |
| NM_021655 | Chga | chromogranin-A |
| NM_017127 | Chka | choline kinase alpha |
| NM_052805 | Chrna3 | neuronal acetylcholine receptor subunit alpha-3 |
| NM_001011955 | Chst1 | carbohydrate sulfotransferase 1 |
| NM_001107307 | Cilp2 | cartilage intermediate layer protein 2 |
| NM_053327 | Clcnka | chloride channel protein ClC-Ka |
| NM_031818 | Clic4 | chloride intracellular channel protein 4 |
| NM_001107501 | Clip3 | CAP-Gly domain-containing linker protein 3 |
| NM_001109300 | Cmtm7 | CKLF-like MARVEL transmembrane domain-containing |
| NM_001011942 | Cnnm2 | metal transporter CNNM2 |
| NM_001007003 | Cnot10 | CCR4-NOT transcription complex subunit 10 |
| NM_001108355 | Cnot6l | CCR4-NOT transcription complex subunit 6-like |
| NM_001014232 | Cnrip1 | CB1 cannabinoid receptor-interacting protein 1 |
| NM_182473 | Corin | atrial natriuretic peptide-converting enzyme |
| NM_001109327 | Coro1c | coronin-1C |
| NM_001002808 | Cpa5 | carboxypeptidase A5 |
| NM_012836 | Cpd | carboxypeptidase D precursor |
| NM_031766 | Cpz | carboxypeptidase Z precursor |
| NM_001004085 | Crat | carnitine O-acetyltransferase |
| NM_133381 | Crebbp | CREB-binding protein |
| NM_001024783 | Creld1 | cysteine-rich with EGF-like domain protein 1 |
| NM_022501 | Crip2 | cysteine-rich protein 2 |
| NM_001014258 | Crls1 | cardiolipin synthase |
| NM_053335 | Ctbp2 | C-terminal-binding protein 2 |
| NM_022266 | Ctgf | connective tissue growth factor precursor |
| NM_001100661 | Ctr9 | Ctr9, Paf1/RNA polymerase II complex component, |
| NM_013156 | Ctsl1 | cathepsin L1 preproprotein |
| NM_057101 | Cyp21a1 | steroid 21-hydroxylase |
| NM_001107495 | Cyp2s1 | cytochrome P450 2S1 |
| NM_001024779 | Cyp2u1 | cytochrome P450 2U1 |
| NM_031327 | Cyr61 | protein CYR61 precursor |
| NM_031024 | Dbn1 | drebrin |
| NM_022297 | Ddah1 | N(G),N(G)-dimethylarginine |
| NM_001108246 | Ddx3x | ATP-dependent RNA helicase DDX3X |
| NM_001013198 | Ddx50 | DEAD (Asp-Glu-Ala-Asp) box polypeptide 50 |
| NM_031801 | Deaf1 | deformed epidermal autoregulatory factor 1 |
| NM_001029916 | Depdc7 | DEP domain-containing protein 7 |
| NM_001109577 | Derl3 | derlin-3 |
| NM_181088 | Dfnb31 | whirlin |
| NM_001191597 | Dhx15 | DEAH (Asp-Glu-Ala-His) box polypeptide 15 |
| NM_032063 | Dll1 | delta-like protein 1 precursor |
| NM_001173357 | Dmkn | dermokine |
| NM_053693 | Dmtf1 | cyclin-D-binding Myb-like transcription factor |
| NM_001024342 | Dnai1 | dynein intermediate chain 1, axonemal |
| NM_001108694 | Dnajc11 | dnaJ homolog subfamily C member 11 |
| NM_001105759 | Dock9 | dedicator of cytokinesis protein 9 |
| NM_001130062 | Dok7 | protein Dok-7 |
| NM_001108141 | Dscaml1 | Down syndrome cell adhesion molecule-like 1 |
| NM_001107767 | Duoxa1 | dual oxidase maturation factor 1 |
| NM_001105734 | Dusp10 | dual specificity protein phosphatase 10 |
| NM_001162408 | Dusp13 | muscle-restricted dual specificity phosphatase |
| NM_001007006 | Dusp13 | testis and skeletal muscle-specific dual |
| NM_001172056 | Dvl2 | dishevelled 2 |
| NM_019226 | Dync1h1 | cytoplasmic dynein 1 heavy chain 1 |
| NM_145772 | Dync1li1 | cytoplasmic dynein 1 light intermediate chain 1 |
| NM_080697 | Dynll2 | dynein light chain 2, cytoplasmic |
| NM_001108506 | Ebf3 | transcription factor COE3 |
| NM_001191076 | Ebf4 | transcription factor COE4 |
| NM_001127541 | Efcab4a | EF-hand calcium-binding domain-containing |
| NM_053903 | Efna5 | ephrin-A5 precursor |
| NM_053633 | Egr2 | early growth response protein 2 |
| NM_019137 | Egr4 | early growth response protein 4 |
| NM_001008773 | Eif1a | eukaryotic translation initiation factor 1A |
| NM_001106867 | Eif1b | eukaryotic translation initiation factor 1b |
| NM_001009391 | Enoph1 | enolase-phosphatase E1 |
| NM_138541 | Epcam | epithelial cell adhesion molecule precursor |
| NM_001105994 | Ephx4 | epoxide hydrolase 4 |
| NM_001108343 | Etnk2 | ethanolamine kinase 2 |
| NM_012555 | Ets1 | protein C-ets-1 |
| NM_001109323 | F8a1 | factor VIII intron 22 protein |
| NM_080895 | Faim | fas apoptotic inhibitory molecule 1 |
| NM_080895 | Faim | fas apoptotic inhibitory molecule 1 |
| NM_001109885 | Fam129b | niban-like protein 1 |
| NM_001012238 | Fam20c | dentin matrix protein 4 |
| NM_001014178 | Fam69b | hypothetical protein LOC362090 |
| NM_001106566 | Fam73b | hypothetical protein LOC296623 |
| NM_001108233 | Farp2 | FERM, RhoGEF and pleckstrin domain-containing |
| NM_022272 | Fbxl20 | F-box/LRR-repeat protein 20 |
| NM_001107203 | Fbxo28 | F-box only protein 28 |
| NM_001011998 | Fbxo9 | F-box only protein 9 |
| NM_001107600 | Fbxw4 | F-box/WD repeat-containing protein 4 |
| NM_001025730 | Fbxw5 | F-box/WD repeat-containing protein 5 |
| NM_001109224 | Fezf1 | fez family zinc finger protein 1 |
| NM_130753 | Fgf15 | fibroblast growth factor 15 |
| NM_130817 | Fgf3 | fibroblast growth factor 3 |
| NM_001106484 | Fign | fidgetin |
| NM_001108955 | Fjx1 | four-jointed box protein 1 |
| NM_001134599 | Flna | filamin-A |
| NM_012742 | Foxa1 | hepatocyte nuclear factor 3-alpha |
| NM_001013248 | Foxb1 | forkhead box B1 |
| NM_001191846 | Foxo1 | forkhead box protein O1 |
| NM_024366 | Freq | neuronal calcium sensor 1 |
| NM_001039337 | Fubp3 | far upstream element-binding protein 3 |
| NM_001025738 | Fusip1 | FUS interacting protein (serine-arginine rich) |
| NM_024370 | Gabrg3 | gamma-aminobutyric acid receptor subunit gamma-3 |
| NM_012563 | Gad2 | glutamate decarboxylase 2 |
| NM_001005888 | Galc | galactocerebrosidase |
| NM_001025053 | Galnt4 | polypeptide N-acetylgalactosaminyltransferase 4 |
| NM_022926 | Galnt7 | N-acetylgalactosaminyltransferase 7 |
| NM_012958 | Galr1 | galanin receptor type 1 |
| NM_019172 | Galr2 | galanin receptor type 2 |
| NM_053708 | Gbx2 | gastrulation brain homeobox 2 |
| NM_019216 | Gdf15 | growth/differentiation factor 15 precursor |
| NM_017088 | Gdi1 | rab GDP dissociation inhibitor alpha |
| NM_017276 | Gdi2 | rab GDP dissociation inhibitor beta |
| NM_001100519 | Gga2 | golgi associated, gamma adaptin ear containing, |
| NM_012960 | Ggh | gamma-glutamyl hydrolase precursor |
| NM_001004273 | Ggnbp2 | gametogenetin-binding protein 2 |
| NM_001037210 | Gipc2 | PDZ domain-containing protein GIPC2 |
| NM_001004099 | Gjb2 | gap junction beta-2 protein |
| NM_019240 | Gjb3 | gap junction beta-3 protein |
| NM_001107308 | Gmip | GEM-interacting protein |
| NM_001191836 | Gnal | guanine nucleotide-binding protein G(olf) |
| NM_053765 | Gne | bifunctional UDP-N-acetylglucosamine |
| NM_001007720 | Gorasp2 | Golgi reassembly-stacking protein 2 |
| NM_012774 | Gpc3 | glypican-3 precursor |
| NM_001014108 | Gpc4 | glypican-4 |
| NM_001034855 | Gpr153 | probable G-protein coupled receptor 153 |
| NM_001108646 | Gpr162 | probable G-protein coupled receptor 162 |
| NM_001191915 | Gpr50 | melatonin-related receptor |
| NM_001012057 | Gpt2 | alanine aminotransferase 2 |
| NM_019282 | Grem1 | gremlin-1 precursor |
| NM_012575 | Grin2c | glutamate [NMDA] receptor subunit epsilon-3 |
| NM_001191873 | Gsc | homeobox protein goosecoid |
| NM_032080 | Gsk3b | glycogen synthase kinase-3 beta |
| NM_001003978 | Gspt1 | eukaryotic peptide chain release factor |
| NM_001001512 | Gtf2i | general transcription factor II-I |
| NM_023956 | Gucy1a2 | guanylate cyclase soluble subunit alpha-2 |
| NM_012578 | H1f0 | histone H1.0 |
| NM_022674 | H2afz | histone H2A.Z |
| NM_013064 | Hcrtr1 | orexin receptor type 1 |
| NM_053447 | Hdac2 | histone deacetylase 2 |
| NM_001108631 | Herc3 | probable E3 ubiquitin-protein ligase HERC3 |
| NM_001012074 | Herc4 | probable E3 ubiquitin-protein ligase HERC4 |
| NM_019236 | Hes2 | transcription factor HES-2 |
| NM_022528 | Hif3a | hypoxia-inducible factor 3-alpha |
| NM_001100986 | Hipk1 | homeodomain-interacting protein kinase 1 |
| NM_031787 | Hipk3 | homeodomain-interacting protein kinase 3 |
| NM_017268 | Hmgcs1 | hydroxymethylglutaryl-CoA synthase, cytoplasmic |
| NM_031330 | Hnrnpab | heterogeneous nuclear ribonucleoprotein A/B |
| NM_001033696 | Hnrpdl | heterogeneous nuclear ribonucleoprotein D-like |
| NM_013075 | Hoxa1 | homeobox protein Hox-A1 |
| NM_017112 | Hpn | serine protease hepsin |
| NM_001106392 | Hs3st5 | heparan sulfate glucosamine 3-O-sulfotransferase |
| NM_001107778 | Hspa12b | heat shock 70 kDa protein 12B |
| NM_053612 | Hspb8 | heat shock protein beta-8 |
| NM_022938 | Htr7 | 5-hydroxytryptamine receptor 7 |
| NM_031721 | Htra1 | serine protease HTRA1 |
| NM_013159 | Ide | insulin-degrading enzyme |
| NM_017183 | Il8rb | C-X-C chemokine receptor type 2 |
| NM_133409 | Ilk | integrin-linked protein kinase |
| NM_172224 | Impa2 | inositol monophosphatase 2 |
| NM_001106083 | Ing2 | inhibitor of growth protein 2 |
| NM_134417 | Ipmk | inositol polyphosphate multikinase |
| NM_001025422 | Irak2 | interleukin-1 receptor-associated kinase-like 2 |
| NM_032074 | Irs3 | insulin receptor substrate 3 |
| NM_181626 | Isca1 | iron-sulfur cluster assembly 1 homolog, |
| NM_001014242 | Isoc1 | isochorismatase domain-containing protein 1 |
| NM_001013880 | Isyna1 | inositol-3-phosphate synthase 1 |
| NM_001014116 | Jmjd8 | jmjC domain-containing protein 8 |
| NM_001106630 | Jph1 | junctophilin-1 |
| NM_001107437 | Jph3 | junctophilin-3 |
| NM_138875 | Jund | transcription factor jun-D |
| NM_031047 | Jup | junction plakoglobin |
| NM_019270 | Kcna3 | potassium voltage-gated channel subfamily A |
| NM_031739 | Kcnd3 | potassium voltage-gated channel subfamily D |
| NM_001101003 | Kcne1l | potassium voltage-gated channel subfamily E |
| NM_031358 | Kcnj11 | ATP-sensitive inward rectifier potassium channel |
| NM_023021 | Kcnn4 | intermediate conductance calcium-activated |
| NM_031597 | Kcnq3 | potassium voltage-gated channel subfamily KQT |
| NM_001108515 | Kdm2a | lysine-specific demethylase 2A |
| NM_001109079 | Kif26b | kinesin family member 26B |
| NM_057202 | Kif5b | kinesin-1 heavy chain |
| NM_001048215 | Kirrel3 | kin of IRRE-like protein 3 |
| NM_023992 | Kiss1r | kiSS-1 receptor |
| NM_022264 | Kit | mast/stem cell growth factor receptor |
| NM_001037354 | Klf11 | Krueppel-like factor 11 |
| NM_057211 | Klf9 | Krueppel-like factor 9 |
| NM_001106054 | Klhl1 | kelch-like protein 1 |
| NM_001106735 | Klhl28 | kelch-like protein 28 |
| NM_001106252 | Klk11 | kallikrein-11 |
| NM_017063 | Kpnb1 | importin subunit beta-1 |
| NM_017068 | Lamp2 | lysosome-associated membrane glycoprotein 2 |
| NM_001108439 | Large | glycosyltransferase-like protein LARGE1 |
| NM_133393 | Lfng | beta-1,3-N-acetylglucosaminyltransferase lunatic |
| NM_173328 | Lgr4 | leucine-rich repeat-containing G-protein coupled |
| NM_031713 | Lilrb3 | leukocyte immunoglobulin-like receptor, |
| NM_001100722 | Lingo1 | leucine rich repeat and Ig domain containing 1 |
| NM_053905 | Lmnb1 | lamin-B1 |
| NM_001103356 | LOC100125364 | hypothetical protein LOC100125364 precursor |
| NM_001143803 | LOC100233213 | hypothetical protein LOC100233213 |
| NM_001177829 | LOC100365935 | hypothetical protein LOC100365935 |
| NM_001013979 | LOC304131 | TAK1-like protein |
| NM_001014007 | LOC306766 | hypothetical protein LOC306766 |
| NM_001014115 | LOC360479 | hypothetical protein LOC360479 |
| NM_001037205 | LOC360997 | similar to ATP-binding cassette, sub-family G |
| NM_001162931 | LOC502128 | POM121 membrane glycoprotein-like 2 isoform 2 |
| NM_001162930 | LOC502128 | POM121 membrane glycoprotein-like 2 isoform 1 |
| NM_001195277 | LOC679651 | transmembrane protein 178-like |
| NM_001109418 | LOC680531 | hypothetical protein LOC680531 |
| NM_001109595 | LOC690478 | hypothetical protein LOC690478 |
| NM_001109616 | LOC691024 | hypothetical protein LOC691024 |
| NM_001109627 | LOC691153 | hypothetical protein LOC691153 |
| NM_030827 | Lrp2 | low-density lipoprotein receptor-related protein |
| NM_001008519 | Lrpprc | leucine-rich PPR motif-containing protein, |
| NM_017242 | Lsamp | limbic system-associated membrane protein |
| NM_001106594 | Mad2l1 | mitotic spindle assembly checkpoint protein |
| NM_139084 | Magi3 | membrane-associated guanylate kinase, WW and PDZ |
| NM_001134971 | Man2b2 | mannosidase, alpha, class 2B, member 2 |
| NM_031643 | Map2k1 | dual specificity mitogen-activated protein |
| NM_138503 | Map3k2 | mitogen-activated protein kinase kinase kinase |
| NM_017212 | Mapt | microtubule-associated protein tau |
| NM_001107590 | Marveld1 | MARVEL domain-containing protein 1 |
| NM_001109132 | Marveld3 | MARVEL domain-containing protein 3 |
| NM_181089 | MAST1 | microtubule-associated serine/threonine-protein |
| NM_021859 | Matk | megakaryocyte-associated tyrosine-protein |
| NM_001108013 | Matn3 | matrilin-3 |
| NM_001108934 | Mblac2 | metallo-beta-lactamase domain-containing protein |
| NM_001025289 | Mbp | Golli-Mbp isoform 1 |
| NM_001107618 | Mdga1 | MAM domain-containing |
| NM_001191727 | Med14 | mediator of RNA polymerase II transcription |
| NM_030860 | Mef2d | myocyte-specific enhancer factor 2D |
| NM_022943 | Mertk | tyrosine-protein kinase Mer precursor |
| NM_001013149 | Mesdc1 | mesoderm development candidate 1 |
| NM_001191626 | Mex3b | RNA-binding protein MEX3B |
| NM_001024267 | MGC109340 | signal peptidase complex subunit 3 |
| NM_001024890 | MGC114520 | hypothetical protein LOC315915 |
| NM_001044292 | MGC116202 | hypothetical protein LOC688736 |
| NM_001007746 | MGC94199 | hypothetical protein LOC362483 |
| NM_001191889 | Mid2 | midline-2 |
| NM_001108737 | Mier2 | mesoderm induction early response protein 2 |
| NR_031878 | Mir132 |  |
| NR_031897 | Mir181c |  |
| NR_032266 | Mir181d |  |
| NR_031909 | Mir193 |  |
| NR_031925 | Mir212 |  |
| NR_031850 | Mir34a |  |
| NR_031848 | Mir34b |  |
| NR_031849 | Mir34c |  |
| NR_037325 | Mir3549 |  |
| NM_001044267 | Mknk1 | MAP kinase-interacting serine/threonine-protein |
| NM_001011985 | Mknk2 | MAP kinase-interacting serine/threonine-protein |
| NM_001108425 | Mocos | molybdenum cofactor sulfurase |
| NM_001034022 | Mprip | myosin phosphatase Rho-interacting protein |
| NM_022529 | Mrpl23 | 39S ribosomal protein L23, mitochondrial |
| NM_001108635 | Mrpl53 | 39S ribosomal protein L53, mitochondrial |
| NM_001106628 | Mrps35 | 28S ribosomal protein S35, mitochondrial |
| NM_053712 | Msx3 | homeo box, msh-like 3 |
| NM_001100833 | Mtch1 | mitochondrial carrier homolog 1 |
| NM_001006960 | Mtp18 | mitochondrial 18 kDa protein |
| NM_001100667 | Mtx1 | metaxin 1 |
| NM_145773 | Mxd3 | max dimerization protein 3 |
| NM_001106257 | Mybpc2 | myosin-binding protein C, fast-type |
| NM_001107344 | Mylip | E3 ubiquitin-protein ligase MYLIP |
| NM_001109678 | Nadk | NAD kinase |
| NM_001107674 | Narg1 | N-alpha-acetyltransferase 15, NatA auxiliary |
| NM_001014785 | Ncbp1 | nuclear cap-binding protein subunit 1 |
| NM_001013059 | Ndfip1 | NEDD4 family-interacting protein 1 |
| NM_012607 | Nefh | neurofilament heavy polypeptide |
| NM_017029 | Nefm | neurofilament medium polypeptide |
| NM_053691 | Nek2 | NIMA-related kinase 2 |
| NM_001013134 | Nek4 | serine/threonine-protein kinase Nek4 |
| NM_001002851 | Nenf | neudesin precursor |
| NM_012987 | Nes | nestin |
| NM_031789 | Nfe2l2 | nuclear factor erythroid 2-related factor 2 |
| NM_012865 | Nfya | nuclear transcription factor Y subunit alpha |
| NM_012610 | Ngfr | tumor necrosis factor receptor superfamily |
| NM_001191733 | Nhs | Nance-Horan syndrome protein |
| NM_013093 | Nkx2-1 | homeobox protein Nkx-2.1 |
| NM_134336 | Nlgn3 | neuroligin-3 precursor |
| NM_001024360 | nod3l | hypothetical protein LOC501101 |
| NM_001012356 | Nono | non-POU domain-containing octamer-binding |
| NM_001105721 | Notch1 | neurogenic locus notch homolog protein 1 |
| NM_001007800 | N-pac | putative oxidoreductase GLYR1 |
| NM_153293 | Npb | neuropeptide B precursor |
| NM_001004231 | Npdc1 | neural proliferation differentiation and control |
| NM_019380 | Nptn | neuroplastin |
| NM_024388 | Nr4a1 | nuclear receptor subfamily 4 group A member 1 |
| NM_031628 | Nr4a3 | nuclear receptor subfamily 4 group A member 3 |
| NM_001100708 | Nrf1 | nuclear respiratory factor 1 |
| NM_001107337 | Nsd1 | histone-lysine N-methyltransferase, H3 lysine-36 |
| NM_053731 | Ntn1 | netrin-1 precursor |
| NM_001106465 | Ntng1 | netrin-G1 |
| NM_001011891 | Nubp2 | cytosolic Fe-S cluster assembly factor NUBP2 |
| NM_181363 | Nudt6 | nucleoside diphosphate-linked moiety X motif 6 |
| NM_021680 | Nxph4 | neurexophilin-4 precursor |
| NM_001025708 | Ogfrl1 | opioid growth factor receptor-like protein 1 |
| NM_001107848 | Ophn1 | oligophrenin-1 |
| NM_001014024 | Orai3 | protein orai-3 |
| NM_001107565 | Oraov1 | oral cancer overexpressed 1 |
| NM_001013079 | Osbpl2 | oxysterol-binding protein-related protein 2 |
| NM_001191700 | Otud4 | OTU domain-containing protein 4 |
| NM_001037496 | Otud5 | OTU domain-containing protein 5 |
| NM_012721 | P2rx6 | P2X purinoceptor 6 |
| NM_134353 | Pabpc1 | polyadenylate-binding protein 1 |
| NM_001009966 | Pacsin3 | protein kinase C and casein kinase substrate in |
| NM_017230 | Padi3 | protein-arginine deiminase type-3 |
| NM_001108937 | Paip1 | polyadenylate-binding protein-interacting |
| NM_133531 | Pank4 | pantothenate kinase 4 |
| NM_199409 | Panx2 | pannexin-2 |
| NM_001191077 | Paqr6 | progestin and adipoQ receptor family member 6 |
| NM_001035249 | Parl | presenilins-associated rhomboid-like protein, |
| NM_033485 | Pawr | PRKC apoptosis WT1 regulator protein |
| NM_001169129 | Pcdh19 | protocadherin-19 |
| NM_001129882 | Pcgf5 | polycomb group RING finger protein 5 |
| NM_001009542 | Pdcd10 | programmed cell death protein 10 |
| NM_031317 | Pdgfc | platelet-derived growth factor C |
| NM_001004072 | Pdha1 | pyruvate dehydrogenase E1 component subunit |
| NM_053826 | Pdk1 | [Pyruvate dehydrogenase [lipoamide]] kinase |
| NM_001013231 | Pea15a | astrocytic phosphoprotein PEA-15 |
| NM_030873 | Pfn2 | profilin-2 |
| NM_001106198 | Pgbd5 | piggyBac transposable element-derived protein 5 |
| NM_001106577 | Phtf2 | putative homeodomain transcription factor 2 |
| NM_031083 | Pi4kb | phosphatidylinositol 4-kinase beta |
| NM_031784 | Pias3 | E3 SUMO-protein ligase PIAS3 |
| NM_001105951 | Pik3c2b | phosphatidylinositol-4-phosphate 3-kinase C2 |
| NM_017034 | Pim1 | proto-oncogene serine/threonine-protein kinase |
| NM_022602 | Pim3 | serine/threonine-protein kinase pim-3 |
| NM_001008369 | Pitpnm1 | membrane-associated phosphatidylinositol |
| NM_053624 | Pitx1 | pituitary homeobox 1 |
| NM_019334 | Pitx2 | pituitary homeobox 2 isoform 2 |
| NM_001042505 | Pitx2 | pituitary homeobox 2 isoform 1 |
| NM_017175 | Pkn1 | serine/threonine-protein kinase N1 |
| NM_001105845 | Plcd3 | 1-phosphatidylinositol-4,5-bisphosphate |
| NM_053758 | Plce1 | 1-phosphatidylinositol-4,5-bisphosphate |
| NM_001134972 | Plekhg2 | pleckstrin homology domain-containing family G |
| NM_001108036 | Plekhh1 | pleckstrin homology domain containing, family H |
| NM_001134637 | Plin5 | perilipin-5 |
| NM_001142915 | Plod2 | procollagen-lysine,2-oxoglutarate 5-dioxygenase |
| NM_001107922 | Pm20d2 | peptidase M20 domain-containing protein 2 |
| NM_001107272 | Pnma2 | paraneoplastic antigen MA2 |
| NM_001109468 | Polr3g | DNA-directed RNA polymerase III subunit RPC7 |
| NM_172085 | Pou3f2 | POU domain, class 3, transcription factor 2 |
| NM_022538 | Ppap2a | lipid phosphate phosphohydrolase 1 |
| NM_013196 | Ppara | peroxisome proliferator-activated receptor |
| NM_001145367 | Pparg | peroxisome proliferator-activated receptor gamma |
| NM_001145366 | Pparg | peroxisome proliferator-activated receptor gamma |
| NM_176075 | Ppargc1b | peroxisome proliferator-activated receptor gamma |
| NM_198773 | Ppm1e | protein phosphatase 1E |
| NM_001191072 | Ppp1r16b | protein phosphatase 1 regulatory inhibitor |
| NM_144746 | Ppp2r2d | serine/threonine-protein phosphatase 2A 55 kDa |
| NM_001108577 | Ppp2r4 | serine/threonine-protein phosphatase 2A |
| NM_181379 | Ppp2r5b | serine/threonine-protein phosphatase 2A 56 kDa |
| NM_001106740 | Ppp2r5e | serine/threonine-protein phosphatase 2A 56 kDa |
| NM_017309 | Ppp3r1 | calcineurin subunit B type 1 |
| NM_001106613 | Ppp4r2 | protein phosphatase 4, regulatory subunit 2 |
| NM_001013957 | Pqbp1 | polyglutamine-binding protein 1 |
| NM_134449 | Prkcdbp | protein kinase C delta-binding protein |
| NM_001033963 | Prkx | serine/threonine-protein kinase PRKX |
| NM_001038588 | Prodh2 | probable proline dehydrogenase 2 |
| NM_021751 | Prom1 | prominin 1 isoform 1 |
| NM_001012121 | Prr5 | proline-rich protein 5 |
| NM_001109116 | Prr7 | proline-rich protein 7 |
| NM_001109226 | Prrt4 | proline-rich transmembrane protein 4 |
| NM_001109027 | Prss33 | serine protease 33 |
| NM_019126 | Psg19 | carcinoembryonic antigen gene family (CGM3) |
| NM_130430 | Psmd9 | 26S proteasome non-ATPase regulatory subunit 9 |
| NM_001106138 | Psmg2 | tumor necrosis factor superfamily, member |
| NM_022516 | Ptbp1 | polypyrimidine tract-binding protein 1 isoform |
| NM_053566 | Ptch1 | protein patched homolog 1 |
| NM_020073 | Pth1r | parathyroid hormone/parathyroid hormone-related |
| NM_031579 | Ptp4a1 | protein tyrosine phosphatase type IVA 1 |
| NM_013088 | Ptpn11 | tyrosine-protein phosphatase non-receptor type |
| NM_019253 | Ptpn5 | tyrosine-protein phosphatase non-receptor type |
| NM_012763 | Ptpra | receptor-type tyrosine-protein phosphatase alpha |
| NM_001108684 | Pum1 | pumilio homolog 1 |
| NM_001106715 | Pum2 | pumilio homolog 2 |
| NM_001108507 | Pwwp2b | PWWP domain-containing protein 2B |
| NM_031152 | Rab11a | ras-related protein Rab-11A |
| NM_001109005 | Rab23 | ras-related protein Rab-23 |
| NM_031718 | Rab2a | ras-related protein Rab-2A |
| NM_053741 | Rap2a | RAS related protein 2a |
| NM_001170531 | Rasgrf1 | ras-specific guanine nucleotide-releasing factor |
| NM_001105753 | Rasgrf1 | ras-specific guanine nucleotide-releasing factor |
| NM_001106261 | Rasip1 | ras-interacting protein 1 |
| NM_001108862 | Rasl10a | ras-like protein family member 10A |
| NM_001106317 | Rassf7 | ras association domain-containing protein 7 |
| NM_031816 | Rbbp7 | histone-binding protein RBBP7 |
| NM_001198584 | RGD1303117 | hypothetical protein LOC292764 |
| NM_001004268 | RGD1303271 | hypothetical protein LOC313018 |
| NM_001108652 | RGD1306151 | hypothetical protein LOC362455 |
| NM_001107663 | RGD1307225 | hypothetical protein LOC310269 |
| NM_001108308 | RGD1307394 | hypothetical protein LOC360667 |
| NM_001017454 | RGD1307799 | IST1 homolog |
| NM_001134596 | RGD1308299 | hypothetical protein LOC367214 |
| NM_001108129 | RGD1309188 | hypothetical protein LOC315463 |
| NM_001079705 | RGD1311558 | shootin-1 |
| NM_001108286 | RGD1311564 | hypothetical protein LOC360590 |
| NM_001127526 | RGD1311605 | hypothetical protein LOC298841 |
| NM_001108678 | RGD1559909 | hypothetical protein LOC362592 |
| NM_001106014 | RGD1560394 | hypothetical protein LOC289728 |
| NM_001109345 | RGD1563349 | hypothetical protein LOC502727 |
| NM_001109311 | RGD1563692 | hypothetical protein LOC501185 |
| NM_001109292 | RGD1564560 | hypothetical protein LOC500988 |
| NM_001030034 | Rhbdf1 | rhomboid family member 1 |
| NM_001191665 | Rilpl1 | RILP-like protein 1 |
| NM_053945 | Rims2 | regulating synaptic membrane exocytosis protein |
| NM_145881 | Rims2 | regulating synaptic membrane exocytosis protein |
| NM_001108052 | Rin3 | ras and Rab interactor 3 |
| NM_001007641 | Rnd3 | rho-related GTP-binding protein RhoE precursor |
| NM_001106836 | Rnf111 | E3 ubiquitin-protein ligase Arkadia |
| NM_001173349 | Rnf128 | E3 ubiquitin-protein ligase RNF128 |
| NM_001191093 | Rnf150 | RING finger protein 150 |
| NM_001107118 | Rnf6 | RING finger protein 6 |
| NM_001012124 | Rpp25 | ribonuclease P protein subunit p25 |
| NM_001048184 | Rragc | ras-related GTP-binding protein C |
| NM_001106641 | Rragd | ras-related GTP-binding protein D |
| NM_001008346 | Rrp8 | ribosomal RNA-processing protein 8 |
| NM_001107980 | Rspo1 | R-spondin-1 |
| NM_001008845 | RT1-CE7 | RT1 class I, locus CE7 |
| NM_053613 | Rtn4r | reticulon-4 receptor precursor |
| NM_181377 | Rtn4rl1 | reticulon-4 receptor-like 1 precursor |
| NM_181380 | Rtn4rl2 | reticulon-4 receptor-like 2 precursor |
| NM_001105870 | Rtp1 | receptor-transporting protein 1 |
| NM_022394 | Safb | scaffold attachment factor B1 |
| NM_001097581 | Sav1 | protein salvador homolog 1 |
| NM_001013985 | Sccpdh | probable saccharopine dehydrogenase |
| NM_001008880 | Scn4b | sodium channel subunit beta-4 precursor |
| NM_177929 | Sdccag8 | serologically defined colon cancer antigen 8 |
| NM_001107637 | Sec63 | translocation protein SEC63 homolog |
| NM_001014253 | Selt | selenoprotein T precursor |
| NM_001107091 | Sema5b | sema domain, seven thrombospondin repeats (type |
| NM_022616 | Sept7 | septin-7 isoform a |
| NM_001109104 | Serp2 | stress-associated endoplasmic reticulum protein |
| NM_053779 | Serpini1 | neuroserpin precursor |
| NM_001007735 | Sertad1 | SERTA domain-containing protein 1 |
| NM_031647 | Sfmbt1 | scm-like with four MBT domains protein 1 |
| NM_001009720 | Sfrs2 | serine/arginine-rich splicing factor 2 |
| NM_001105937 | Sgsm1 | small G protein signaling modulator 1 |
| NM_001137647 | Sh3bgrl2 | SH3 domain-binding glutamic acid-rich-like |
| NM_053360 | Sh3kbp1 | SH3 domain-containing kinase-binding protein 1 |
| NM_001191936 | Shisa2 | protein shisa-2 homolog |
| NM_001191922 | Shisa6 | protein shisa-6 homolog |
| NM_134457 | Siah2 | E3 ubiquitin-protein ligase SIAH2 |
| NM_021693 | Sik1 | serine/threonine-protein kinase SIK1 |
| NM_031798 | Slc12a2 | solute carrier family 12 member 2 |
| NM_134363 | Slc12a5 | solute carrier family 12 member 5 |
| NM_001013144 | Slc12a7 | solute carrier family 12 member 7 |
| NM_153625 | Slc12a8 | solute carrier family 12 member 8 |
| NM_012716 | Slc16a1 | monocarboxylate transporter 1 |
| NM_147216 | Slc16a2 | monocarboxylate transporter 8 |
| NM_032065 | Slc1a6 | excitatory amino acid transporter 4 |
| NM_177421 | Slc22a17 | solute carrier family 22 member 17 |
| NM_001106327 | Slc22a20 | solute carrier family 22 member 20 |
| NM_017316 | Slc23a2 | solute carrier family 23 member 2 |
| NM_001108051 | Slc24a4 | sodium/potassium/calcium exchanger 4 |
| NM_001013996 | Slc25a37 | mitoferrin-1 |
| NM_001100515 | Slc25a46 | solute carrier family 25 member 46 |
| NM_001105985 | Slc30a10 | zinc transporter 10 |
| NM_133600 | Slc31a1 | high affinity copper uptake protein 1 |
| NM_001134687 | Slc35e3 | solute carrier family 35 member E3 |
| NM_001105950 | Slc35f5 | solute carrier family 35 member F5 |
| NM_001011952 | Slc39a8 | zinc transporter ZIP8 |
| NM_001191920 | Slc47a2 | multidrug and toxin extrusion protein 2 |
| NM_053424 | Slc4a4 | electrogenic sodium bicarbonate cotransporter 1 |
| NM_130746 | Slc5a6 | sodium-dependent multivitamin transporter |
| NM_017206 | Slc6a6 | sodium- and chloride-dependent taurine |
| NM_001113335 | Slc9a2 | sodium/hydrogen exchanger 2 isoform 1 |
| NM_022667 | Slco2a1 | solute carrier organic anion transporter family |
| NM_013095 | Smad3 | mothers against decapentaplegic homolog 3 |
| NM_030858 | Smad7 | mothers against decapentaplegic homolog 7 |
| NM_001107419 | Smarca5 | SWI/SNF-related matrix-associated |
| NM_001108752 | Smarcd1 | SWI/SNF-related matrix-associated |
| NM_206851 | Smyd2 | SET and MYND domain-containing protein 2 |
| NM_001106832 | Snx22 | sorting nexin-22 |
| NM_001191563 | Sorcs1 | VPS10 domain-containing receptor SorCS1 |
| NM_001106367 | Sorcs3 | VPS10 domain-containing receptor SorCS3 |
| NM_019193 | Sox10 | transcription factor SOX-10 |
| NM_001106850 | Sox14 | SRY (sex determining region Y)-box 14 |
| NM_031792 | Spag4 | sperm-associated antigen 4 protein |
| NM_001106530 | Spag4l | SUN domain-containing protein 5 |
| NM_001108549 | Spata5 | spermatogenesis-associated protein 5 |
| NM_181388 | Spg7 | paraplegin |
| NM_001039208 | Spns1 | protein spinster homolog 1 |
| NM_172067 | Spon1 | spondin-1 precursor |
| NM_001106988 | Spsb3 | SPRY domain-containing SOCS box protein 3 |
| NM_175843 | Sqstm1 | sequestosome-1 isoform 1 |
| NM_181550 | Sqstm1 | sequestosome-1 isoform 2 |
| NM_001135711 | Srrp | 35 kDa SR repressor protein |
| NM_012747 | Stat3 | signal transducer and activator of transcription |
| NM_031704 | Stx5 | syntaxin-5 |
| NM_031665 | Stx6 | syntaxin-6 |
| NM_001100750 | Suclg2 | succinyl-CoA ligase [GDP-forming] subunit beta, |
| NM_001025125 | Sumf2 | sulfatase-modifying factor 2 |
| NM_001108883 | Suv39h2 | histone-lysine N-methyltransferase SUV39H2 |
| NM_019133 | Syn1 | synapsin-1 isoform a |
| NM_001004107 | Tacc1 | transforming, acidic coiled-coil containing |
| NM_001170455 | Tada2b | transcriptional adaptor 2B |
| NM_001025734 | Tada3l | transcriptional adapter 3 |
| NM_001013127 | Tagln2 | transgelin-2 |
| NM_001025419 | Tax1bp3 | tax1-binding protein 3 |
| NM_001015022 | Tbc1d10a | TBC1 domain family member 10A |
| NM_181638 | Tbx3 | T-box transcription factor TBX3 |
| NM_001130077 | Tcerg1l | transcription elongation regulator 1-like |
| NM_201420 | Tcfap2c | transcription factor AP-2 gamma |
| NM_001098216 | Tead3 | TEA domain family member 3 |
| NM_019194 | Tef | thyrotroph embryonic factor |
| NM_201655 | Tepp | testis, prostate and placenta-expressed protein |
| NM_031131 | Tgfb2 | transforming growth factor beta-2 precursor |
| NM_031132 | Tgfbr2 | TGF-beta receptor type-2 precursor |
| NM_001100558 | Tiam1 | T-cell lymphoma invasion and metastasis 1 |
| NM_001108249 | Timm17b | translocase of inner mitochondrial membrane 17 |
| NM_001172125 | Tlx2 | T-cell leukemia, homeobox 2 |
| NM_001107015 | Tm4sf5 | transmembrane 4 L6 family member 5 |
| NM_001105758 | Tmed7 | transmembrane emp24 domain-containing protein 7 |
| NM_001134410 | Tmem132e | transmembrane protein 132E |
| NM_001008774 | Tmem170b | transmembrane protein 170B |
| NM_001135712 | Tmem185a | transmembrane protein 185A |
| NM_001191668 | Tmem185b | transmembrane protein 185B |
| NM_001109480 | Tmem229a | transmembrane protein 229A |
| NM_001191610 | Tmem60 | transmembrane protein 60 |
| NM_001017455 | Tmem80 | transmembrane protein 80 |
| NM_001109485 | Tmem90a | capucin |
| NM_001105806 | Tmem93 | transmembrane protein 93 |
| NM_153311 | Tmprss5 | transmembrane protease serine 5 |
| NR_024118 | Tnxa |  |
| NM_001105818 | Tp53i13 | tumor protein p53-inducible protein 13 |
| NM_001044295 | Tpd52l1 | tumor protein D52-like 1 |
| NM_001106788 | Trabd | traB domain-containing protein |
| NM_053916 | Trim28 | transcription intermediary factor 1-beta |
| NM_031786 | Trim3 | tripartite motif-containing protein 3 |
| NM_001106453 | Trim45 | tripartite motif-containing protein 45 |
| NM_130420 | Trim9 | E3 ubiquitin-protein ligase TRIM9 |
| NM_001109912 | Tsc22d1 | TSC22 domain family protein 1 isoform 1 |
| NM_022589 | Tspan2 | tetraspanin-2 |
| NM_012808 | Tst | thiosulfate sulfurtransferase |
| NM_001108663 | Tstd2 | thiosulfate sulfurtransferase/rhodanese-like |
| NM_001025675 | Tubb6 | tubulin, beta 6 |
| NM_001039163 | Tusc5 | tumor suppressor candidate 5 homolog |
| NM_001037643 | Ube2z | ubiquitin-conjugating enzyme E2 Z |
| NM_001105723 | Ubtf | nucleolar transcription factor 1 isoform 1 |
| NM_019354 | Ucp2 | mitochondrial uncoupling protein 2 |
| NM_001003709 | Ufc1 | ubiquitin-fold modifier-conjugating enzyme 1 |
| NM_001077660 | Urg4 | up-regulated gene 4 |
| NM_145184 | Usp15 | ubiquitin carboxyl-terminal hydrolase 15 |
| NM_001106120 | Usp6nl | USP6 N-terminal-like protein |
| NM_022637 | Vax2 | ventral anterior homeobox 2 |
| NM_001107248 | Vcl | vinculin |
| NM_053653 | Vegfc | vascular endothelial growth factor C precursor |
| NM_203338 | Vkorc1l1 | vitamin K epoxide reductase complex subunit |
| NM_013155 | Vldlr | very low-density lipoprotein receptor precursor |
| NM_001106092 | Vps36 | vacuolar protein-sorting-associated protein 36 |
| NM_001108479 | Vstm2b | V-set and transmembrane domain-containing |
| NM_001169128 | Vsx2 | visual system homeobox 2 |
| NM_001135894 | Wdr25l | WD repeat domain 25-like |
| NM_001037791 | Wdr43 | WD repeat domain 43 |
| NM_001110489 | Wdr86 | WD repeat-containing protein 86 |
| NM_001191556 | Wnk2 | serine/threonine-protein kinase WNK2 |
| NM_001108227 | Wnt10a | protein Wnt-10a |
| NM_001108226 | Wnt6 | protein Wnt-6 |
| NM_001105783 | Wnt9a | protein Wnt-9a |
| NM_001106184 | Wwp2 | NEDD4-like E3 ubiquitin-protein ligase WWP2 |
| NM_022231 | Xiap | baculoviral IAP repeat-containing protein 4 |
| NM_001105992 | Xpr1 | xenotropic and polytropic retrovirus receptor 1 |
| NM_022296 | Xylt2 | xylosyltransferase 2 |
| NM_199383 | Yipf1 | protein YIPF1 |
| NM_001014208 | Yipf2 | protein YIPF2 |
| NM_001025747 | Yipf6 | protein YIPF6 |
| NM_175604 | Yrdc | yrdC domain-containing protein, mitochondrial |
| NM_019377 | Ywhab | 14-3-3 protein beta/alpha |
| NM_019376 | Ywhag | 14-3-3 protein gamma |
| NM_001106129 | Zadh2 | zinc-binding alcohol dehydrogenase |
| NM_001107097 | Zbtb11 | zinc finger and BTB domain-containing protein |
| NM_001130537 | Zbtb39 | zinc finger and BTB domain-containing protein |
| NM_001170577 | Zfp167 | zinc finger protein 167 |
| NM_001034831 | Zfp384 | zinc finger protein 384 isoform 2 |
| NM_133429 | Zfp384 | zinc finger protein 384 isoform 1 |
| NM_001034830 | Zfp384 | zinc finger protein 384 isoform 1 |
| NM_001012093 | Zfp64 | zinc finger protein 64 |
| NM_001109225 | Zfp800 | zinc finger protein 800 |
| NM_001109017 | Zfx | zinc finger X-chromosomal protein |
| NM_001108725 | Zfyve21 | zinc finger FYVE domain-containing protein 21 |
| NM_203369 | Zmynd11 | zinc finger MYND domain-containing protein 11 |
| NM_001024878 | Znrf4 | zinc/RING finger protein 4 |
| NM_031616 | Zranb2 | zinc finger Ran-binding domain-containing |
| NM_053761 | Zyx | zyxin |

**Dataset S3 List of H3K27me3-enriched genes shared by control and Pb-exposed neurons**

| **Accession** | **Symbol** | **Gene Name** |
| --- | --- | --- |
| NM_133411 | Abcc4 | multidrug resistance-associated protein 4 |
| NM_001107186 | Abl2 | tyrosine-protein kinase ABL2 |
| NM_022190 | Acan | aggrecan core protein |
| NM_001012013 | Acbd4 | acyl-CoA-binding domain-containing protein 4 |
| NM_001170325 | Actn2 | actinin alpha 2 |
| NM_001039028 | Actr1b | ARP1 actin-related protein 1 homolog B |
| NM_001107239 | Adcy1 | adenylate cyclase type 1 |
| NM_001134744 | Agpat5 | 1-acyl-sn-glycerol-3-phosphate acyltransferase |
| NM_001007654 | Agtrap | type-1 angiotensin II receptor-associated |
| NM_001001801 | Akap7 | A-kinase anchoring protein 18 ,isoform delta |
| NM_053896 | Aldh1a2 | retinal dehydrogenase 2 |
| NM_012902 | Amh | muellerian-inhibiting factor precursor |
| NM_001191565 | Ankrd33b | ankyrin repeat domain-containing protein 33B |
| NM_031008 | Ap2a2 | AP-2 complex subunit alpha-2 |
| NM_031779 | Apba1 | amyloid beta A4 precursor protein-binding family |
| NM_012779 | Aqp5 | aquaporin-5 |
| NM_024152 | Arf6 | ADP-ribosylation factor 6 |
| NM_001106061 | Arhgef3 | rho guanine nucleotide exchange factor 3 |
| NM_001173981 | Arid3c | AT-rich interactive domain-containing protein |
| NM_001037767 | Arpc5l | actin-related protein 2/3 complex subunit 5-like |
| NM_001047881 | Arsi | arylsulfatase I precursor |
| NM_001108420 | Asb13 | ankyrin repeat and SOCS box-containing 13 |
| NM_001106389 | Asf1a | ASF1 anti-silencing function 1 homolog A |
| NM_001035002 | Atad1 | ATPase family AAA domain-containing protein 1 |
| NM_024403 | Atf4 | cyclic AMP-dependent transcription factor ATF-4 |
| NM_012913 | Atp1b3 | sodium/potassium-transporting ATPase subunit |
| NM_023093 | Atp5a1 | ATP synthase subunit alpha, mitochondrial |
| NM_001106068 | B3gnt3 | UDP-GlcNAc:betaGal |
| NM_001107113 | Bach1 | transcription regulator protein BACH1 |
| NM_022300 | Basp1 | brain acid soluble protein 1 |
| NM_001007707 | Brp16 | brain protein 16 |
| NM_001106555 | C8g | complement component C8 gamma chain |
| NM_175595 | Cacna2d3 | voltage-dependent calcium channel subunit |
| NM_031338 | Camkk2 | calcium/calmodulin-dependent protein kinase |
| NM_001107071 | Cbx2 | chromobox protein homolog 2 |
| NM_053662 | Ccnl1 | cyclin-L1 |
| NM_022269 | Cd55 | decay accelerating factor 1 |
| NM_001048044 | Cdc42ep3 | CDC42 effector protein (Rho GTPase binding) 3 |
| NM_131902 | Cdkn2c | cyclin-dependent kinase 4 inhibitor C |
| NM_001025682 | Cdr2 | cerebellar degeneration-related protein 2 |
| NM_024125 | Cebpb | CCAAT/enhancer-binding protein beta |
| NM_001100514 | Cep76 | centrosomal protein 76kDa |
| NM_019164 | Chad | chondroadherin precursor |
| NM_021655 | Chga | chromogranin-A |
| NM_052805 | Chrna3 | neuronal acetylcholine receptor subunit alpha-3 |
| NM_001107307 | Cilp2 | cartilage intermediate layer protein 2 |
| NM_053327 | Clcnka | chloride channel protein ClC-Ka |
| NM_001107501 | Clip3 | CAP-Gly domain-containing linker protein 3 |
| NM_001109300 | Cmtm7 | CKLF-like MARVEL transmembrane domain-containing |
| NM_001108355 | Cnot6l | CCR4-NOT transcription complex subunit 6-like |
| NM_182473 | Corin | atrial natriuretic peptide-converting enzyme |
| NM_001109327 | Coro1c | coronin-1C |
| NM_001002808 | Cpa5 | carboxypeptidase A5 |
| NM_001004085 | Crat | carnitine O-acetyltransferase |
| NM_133381 | Crebbp | CREB-binding protein |
| NM_001024783 | Creld1 | cysteine-rich with EGF-like domain protein 1 |
| NM_022501 | Crip2 | cysteine-rich protein 2 |
| NM_001107495 | Cyp2s1 | cytochrome P450 2S1 |
| NM_031327 | Cyr61 | protein CYR61 precursor |
| NM_001108246 | Ddx3x | ATP-dependent RNA helicase DDX3X |
| NM_031801 | Deaf1 | deformed epidermal autoregulatory factor 1 |
| NM_001029916 | Depdc7 | DEP domain-containing protein 7 |
| NM_181088 | Dfnb31 | whirlin |
| NM_001173357 | Dmkn | dermokine |
| NM_053693 | Dmtf1 | cyclin-D-binding Myb-like transcription factor |
| NM_001024342 | Dnai1 | dynein intermediate chain 1, axonemal |
| NM_001108694 | Dnajc11 | dnaJ homolog subfamily C member 11 |
| NM_001130062 | Dok7 | protein Dok-7 |
| NM_001108141 | Dscaml1 | Down syndrome cell adhesion molecule-like 1 |
| NM_001107767 | Duoxa1 | dual oxidase maturation factor 1 |
| NM_001007006 | Dusp13 | testis and skeletal muscle-specific dual |
| NM_001172056 | Dvl2 | dishevelled 2 |
| NM_019226 | Dync1h1 | cytoplasmic dynein 1 heavy chain 1 |
| NM_001108506 | Ebf3 | transcription factor COE3 |
| NM_001191076 | Ebf4 | transcription factor COE4 |
| NM_001127541 | Efcab4a | EF-hand calcium-binding domain-containing |
| NM_053633 | Egr2 | early growth response protein 2 |
| NM_138541 | Epcam | epithelial cell adhesion molecule precursor |
| NM_001105994 | Ephx4 | epoxide hydrolase 4 |
| NM_001108343 | Etnk2 | ethanolamine kinase 2 |
| NM_001109323 | F8a1 | factor VIII intron 22 protein |
| NM_001014178 | Fam69b | hypothetical protein LOC362090 |
| NM_001106566 | Fam73b | hypothetical protein LOC296623 |
| NM_001108233 | Farp2 | FERM, RhoGEF and pleckstrin domain-containing |
| NM_001025730 | Fbxw5 | F-box/WD repeat-containing protein 5 |
| NM_001109224 | Fezf1 | fez family zinc finger protein 1 |
| NM_130753 | Fgf15 | fibroblast growth factor 15 |
| NM_001013248 | Foxb1 | forkhead box B1 |
| NM_001191846 | Foxo1 | forkhead box protein O1 |
| NM_024370 | Gabrg3 | gamma-aminobutyric acid receptor subunit gamma-3 |
| NM_001005888 | Galc | galactocerebrosidase |
| NM_001025053 | Galnt4 | polypeptide N-acetylgalactosaminyltransferase 4 |
| NM_022926 | Galnt7 | N-acetylgalactosaminyltransferase 7 |
| NM_053708 | Gbx2 | gastrulation brain homeobox 2 |
| NM_019216 | Gdf15 | growth/differentiation factor 15 precursor |
| NM_017276 | Gdi2 | rab GDP dissociation inhibitor beta |
| NM_001037210 | Gipc2 | PDZ domain-containing protein GIPC2 |
| NM_001004099 | Gjb2 | gap junction beta-2 protein |
| NM_019240 | Gjb3 | gap junction beta-3 protein |
| NM_001107308 | Gmip | GEM-interacting protein |
| NM_001191836 | Gnal | guanine nucleotide-binding protein G(olf) |
| NM_012774 | Gpc3 | glypican-3 precursor |
| NM_001108646 | Gpr162 | probable G-protein coupled receptor 162 |
| NM_001191915 | Gpr50 | melatonin-related receptor |
| NM_001012057 | Gpt2 | alanine aminotransferase 2 |
| NM_019282 | Grem1 | gremlin-1 precursor |
| NM_012575 | Grin2c | glutamate [NMDA] receptor subunit epsilon-3 |
| NM_001001512 | Gtf2i | general transcription factor II-I |
| NM_012578 | H1f0 | histone H1.0 |
| NM_013064 | Hcrtr1 | orexin receptor type 1 |
| NM_053447 | Hdac2 | histone deacetylase 2 |
| NM_001108631 | Herc3 | probable E3 ubiquitin-protein ligase HERC3 |
| NM_019236 | Hes2 | transcription factor HES-2 |
| NM_022528 | Hif3a | hypoxia-inducible factor 3-alpha |
| NM_017268 | Hmgcs1 | hydroxymethylglutaryl-CoA synthase, cytoplasmic |
| NM_001107094 | Hoxd10 | homeo box D10 |
| NM_053612 | Hspb8 | heat shock protein beta-8 |
| NM_022938 | Htr7 | 5-hydroxytryptamine receptor 7 |
| NM_031721 | Htra1 | serine protease HTRA1 |
| NM_013159 | Ide | insulin-degrading enzyme |
| NM_133409 | Ilk | integrin-linked protein kinase |
| NM_172224 | Impa2 | inositol monophosphatase 2 |
| NM_134417 | Ipmk | inositol polyphosphate multikinase |
| NM_001013880 | Isyna1 | inositol-3-phosphate synthase 1 |
| NM_001014116 | Jmjd8 | jmjC domain-containing protein 8 |
| NM_138875 | Jund | transcription factor jun-D |
| NM_023021 | Kcnn4 | intermediate conductance calcium-activated |
| NM_057202 | Kif5b | kinesin-1 heavy chain |
| NM_001048215 | Kirrel3 | kin of IRRE-like protein 3 |
| NM_023992 | Kiss1r | kiSS-1 receptor |
| NM_022264 | Kit | mast/stem cell growth factor receptor |
| NM_001106054 | Klhl1 | kelch-like protein 1 |
| NM_001106252 | Klk11 | kallikrein-11 |
| NM_017063 | Kpnb1 | importin subunit beta-1 |
| NM_001100722 | Lingo1 | leucine rich repeat and Ig domain containing 1 |
| NM_001143803 | LOC100233213 | hypothetical protein LOC100233213 |
| NM_001177829 | LOC100365935 | hypothetical protein LOC100365935 |
| NM_001013979 | LOC304131 | TAK1-like protein |
| NM_001162931 | LOC502128 | POM121 membrane glycoprotein-like 2 isoform 2 |
| NM_001162930 | LOC502128 | POM121 membrane glycoprotein-like 2 isoform 1 |
| NM_001195277 | LOC679651 | transmembrane protein 178-like |
| NM_001109418 | LOC680531 | hypothetical protein LOC680531 |
| NM_001109595 | LOC690478 | hypothetical protein LOC690478 |
| NM_001109616 | LOC691024 | hypothetical protein LOC691024 |
| NM_138503 | Map3k2 | mitogen-activated protein kinase kinase kinase |
| NM_017212 | Mapt | microtubule-associated protein tau |
| NM_001107590 | Marveld1 | MARVEL domain-containing protein 1 |
| NM_001109132 | Marveld3 | MARVEL domain-containing protein 3 |
| NM_181089 | MAST1 | microtubule-associated serine/threonine-protein |
| NM_001108013 | Matn3 | matrilin-3 |
| NM_001025289 | Mbp | Golli-Mbp isoform 1 |
| NM_022943 | Mertk | tyrosine-protein kinase Mer precursor |
| NM_001024890 | MGC114520 | hypothetical protein LOC315915 |
| NM_001191889 | Mid2 | midline-2 |
| NM_001108737 | Mier2 | mesoderm induction early response protein 2 |
| NR_031865 | Mir124-3 |  |
| NR_031878 | Mir132 |  |
| NR_031897 | Mir181c |  |
| NR_032266 | Mir181d |  |
| NR_031909 | Mir193 |  |
| NR_031925 | Mir212 |  |
| NR_031850 | Mir34a |  |
| NM_001034022 | Mprip | myosin phosphatase Rho-interacting protein |
| NM_022529 | Mrpl23 | 39S ribosomal protein L23, mitochondrial |
| NM_001108635 | Mrpl53 | 39S ribosomal protein L53, mitochondrial |
| NM_001106628 | Mrps35 | 28S ribosomal protein S35, mitochondrial |
| NM_053712 | Msx3 | homeo box, msh-like 3 |
| NM_001100833 | Mtch1 | mitochondrial carrier homolog 1 |
| NM_001106257 | Mybpc2 | myosin-binding protein C, fast-type |
| NM_001013059 | Ndfip1 | NEDD4 family-interacting protein 1 |
| NM_017029 | Nefm | neurofilament medium polypeptide |
| NM_001013134 | Nek4 | serine/threonine-protein kinase Nek4 |
| NM_001002851 | Nenf | neudesin precursor |
| NM_012865 | Nfya | nuclear transcription factor Y subunit alpha |
| NM_012610 | Ngfr | tumor necrosis factor receptor superfamily |
| NM_001191733 | Nhs | Nance-Horan syndrome protein |
| NM_013093 | Nkx2-1 | homeobox protein Nkx-2.1 |
| NM_134336 | Nlgn3 | neuroligin-3 precursor |
| NM_001105721 | Notch1 | neurogenic locus notch homolog protein 1 |
| NM_153293 | Npb | neuropeptide B precursor |
| NM_019380 | Nptn | neuroplastin |
| NM_031628 | Nr4a3 | nuclear receptor subfamily 4 group A member 3 |
| NM_001107337 | Nsd1 | histone-lysine N-methyltransferase, H3 lysine-36 |
| NM_001106465 | Ntng1 | netrin-G1 |
| NM_001011891 | Nubp2 | cytosolic Fe-S cluster assembly factor NUBP2 |
| NM_181363 | Nudt6 | nucleoside diphosphate-linked moiety X motif 6 |
| NM_021680 | Nxph4 | neurexophilin-4 precursor |
| NM_001107848 | Ophn1 | oligophrenin-1 |
| NM_001014024 | Orai3 | protein orai-3 |
| NM_001107565 | Oraov1 | oral cancer overexpressed 1 |
| NM_134353 | Pabpc1 | polyadenylate-binding protein 1 |
| NM_017230 | Padi3 | protein-arginine deiminase type-3 |
| NM_133531 | Pank4 | pantothenate kinase 4 |
| NM_001191077 | Paqr6 | progestin and adipoQ receptor family member 6 |
| NM_001035249 | Parl | presenilins-associated rhomboid-like protein, |
| NM_001169129 | Pcdh19 | protocadherin-19 |
| NM_001129882 | Pcgf5 | polycomb group RING finger protein 5 |
| NM_001009542 | Pdcd10 | programmed cell death protein 10 |
| NM_031317 | Pdgfc | platelet-derived growth factor C |
| NM_001004072 | Pdha1 | pyruvate dehydrogenase E1 component subunit |
| NM_053826 | Pdk1 | [Pyruvate dehydrogenase [lipoamide]] kinase |
| NM_001106198 | Pgbd5 | piggyBac transposable element-derived protein 5 |
| NM_031784 | Pias3 | E3 SUMO-protein ligase PIAS3 |
| NM_001105951 | Pik3c2b | phosphatidylinositol-4-phosphate 3-kinase C2 |
| NM_022602 | Pim3 | serine/threonine-protein kinase pim-3 |
| NM_001105845 | Plcd3 | 1-phosphatidylinositol-4,5-bisphosphate |
| NM_053758 | Plce1 | 1-phosphatidylinositol-4,5-bisphosphate |
| NM_001134972 | Plekhg2 | pleckstrin homology domain-containing family G |
| NM_001108036 | Plekhh1 | pleckstrin homology domain containing, family H |
| NM_172085 | Pou3f2 | POU domain, class 3, transcription factor 2 |
| NM_022538 | Ppap2a | lipid phosphate phosphohydrolase 1 |
| NM_013196 | Ppara | peroxisome proliferator-activated receptor |
| NM_144746 | Ppp2r2d | serine/threonine-protein phosphatase 2A 55 kDa |
| NM_001108577 | Ppp2r4 | serine/threonine-protein phosphatase 2A |
| NM_001106613 | Ppp4r2 | protein phosphatase 4, regulatory subunit 2 |
| NM_134449 | Prkcdbp | protein kinase C delta-binding protein |
| NM_001033963 | Prkx | serine/threonine-protein kinase PRKX |
| NM_001038588 | Prodh2 | probable proline dehydrogenase 2 |
| NM_001024305 | Prpf38b | pre-mRNA-splicing factor 38B |
| NM_001109027 | Prss33 | serine protease 33 |
| NM_019126 | Psg19 | carcinoembryonic antigen gene family (CGM3) |
| NM_130430 | Psmd9 | 26S proteasome non-ATPase regulatory subunit 9 |
| NM_001106138 | Psmg2 | tumor necrosis factor superfamily, member |
| NM_022516 | Ptbp1 | polypyrimidine tract-binding protein 1 isoform |
| NM_001108507 | Pwwp2b | PWWP domain-containing protein 2B |
| NM_001109005 | Rab23 | ras-related protein Rab-23 |
| NM_031718 | Rab2a | ras-related protein Rab-2A |
| NM_053741 | Rap2a | RAS related protein 2a |
| NM_001170531 | Rasgrf1 | ras-specific guanine nucleotide-releasing factor |
| NM_001105753 | Rasgrf1 | ras-specific guanine nucleotide-releasing factor |
| NM_001106261 | Rasip1 | ras-interacting protein 1 |
| NM_001106317 | Rassf7 | ras association domain-containing protein 7 |
| NM_001004268 | RGD1303271 | hypothetical protein LOC313018 |
| NM_001107663 | RGD1307225 | hypothetical protein LOC310269 |
| NM_001134596 | RGD1308299 | hypothetical protein LOC367214 |
| NM_001108129 | RGD1309188 | hypothetical protein LOC315463 |
| NM_001079705 | RGD1311558 | shootin-1 |
| NM_001127526 | RGD1311605 | hypothetical protein LOC298841 |
| NM_001108678 | RGD1559909 | hypothetical protein LOC362592 |
| NM_001106014 | RGD1560394 | hypothetical protein LOC289728 |
| NM_001109345 | RGD1563349 | hypothetical protein LOC502727 |
| NM_001109311 | RGD1563692 | hypothetical protein LOC501185 |
| NM_001109292 | RGD1564560 | hypothetical protein LOC500988 |
| NM_053945 | Rims2 | regulating synaptic membrane exocytosis protein |
| NM_001106836 | Rnf111 | E3 ubiquitin-protein ligase Arkadia |
| NM_001173349 | Rnf128 | E3 ubiquitin-protein ligase RNF128 |
| NM_001191093 | Rnf150 | RING finger protein 150 |
| NM_001048184 | Rragc | ras-related GTP-binding protein C |
| NM_001106641 | Rragd | ras-related GTP-binding protein D |
| NM_001008346 | Rrp8 | ribosomal RNA-processing protein 8 |
| NM_181380 | Rtn4rl2 | reticulon-4 receptor-like 2 precursor |
| NM_022394 | Safb | scaffold attachment factor B1 |
| NM_001013985 | Sccpdh | probable saccharopine dehydrogenase |
| NM_001008880 | Scn4b | sodium channel subunit beta-4 precursor |
| NM_177929 | Sdccag8 | serologically defined colon cancer antigen 8 |
| NM_001107637 | Sec63 | translocation protein SEC63 homolog |
| NM_022616 | Sept7 | septin-7 isoform a |
| NM_001109104 | Serp2 | stress-associated endoplasmic reticulum protein |
| NM_053779 | Serpini1 | neuroserpin precursor |
| NM_031647 | Sfmbt1 | scm-like with four MBT domains protein 1 |
| NM_001105937 | Sgsm1 | small G protein signaling modulator 1 |
| NM_053360 | Sh3kbp1 | SH3 domain-containing kinase-binding protein 1 |
| NM_134457 | Siah2 | E3 ubiquitin-protein ligase SIAH2 |
| NM_021693 | Sik1 | serine/threonine-protein kinase SIK1 |
| NM_031798 | Slc12a2 | solute carrier family 12 member 2 |
| NM_134363 | Slc12a5 | solute carrier family 12 member 5 |
| NM_153625 | Slc12a8 | solute carrier family 12 member 8 |
| NM_147216 | Slc16a2 | monocarboxylate transporter 8 |
| NM_001106327 | Slc22a20 | solute carrier family 22 member 20 |
| NM_017316 | Slc23a2 | solute carrier family 23 member 2 |
| NM_133600 | Slc31a1 | high affinity copper uptake protein 1 |
| NM_001105950 | Slc35f5 | solute carrier family 35 member F5 |
| NM_001191920 | Slc47a2 | multidrug and toxin extrusion protein 2 |
| NM_130746 | Slc5a6 | sodium-dependent multivitamin transporter |
| NM_017206 | Slc6a6 | sodium- and chloride-dependent taurine |
| NM_001113335 | Slc9a2 | sodium/hydrogen exchanger 2 isoform 1 |
| NM_022667 | Slco2a1 | solute carrier organic anion transporter family |
| NM_030858 | Smad7 | mothers against decapentaplegic homolog 7 |
| NM_206851 | Smyd2 | SET and MYND domain-containing protein 2 |
| NM_001191563 | Sorcs1 | VPS10 domain-containing receptor SorCS1 |
| NM_001106367 | Sorcs3 | VPS10 domain-containing receptor SorCS3 |
| NM_019193 | Sox10 | transcription factor SOX-10 |
| NM_001106850 | Sox14 | SRY (sex determining region Y)-box 14 |
| NM_001106530 | Spag4l | SUN domain-containing protein 5 |
| NM_001108549 | Spata5 | spermatogenesis-associated protein 5 |
| NM_181388 | Spg7 | paraplegin |
| NM_001039208 | Spns1 | protein spinster homolog 1 |
| NM_172067 | Spon1 | spondin-1 precursor |
| NM_001106988 | Spsb3 | SPRY domain-containing SOCS box protein 3 |
| NM_001135711 | Srrp | 35 kDa SR repressor protein |
| NM_031704 | Stx5 | syntaxin-5 |
| NM_031665 | Stx6 | syntaxin-6 |
| NM_001100750 | Suclg2 | succinyl-CoA ligase [GDP-forming] subunit beta, |
| NM_001025125 | Sumf2 | sulfatase-modifying factor 2 |
| NM_001107341 | Susd3 | sushi domain-containing protein 3 |
| NM_001025419 | Tax1bp3 | tax1-binding protein 3 |
| NM_181638 | Tbx3 | T-box transcription factor TBX3 |
| NM_201420 | Tcfap2c | transcription factor AP-2 gamma |
| NM_001098216 | Tead3 | TEA domain family member 3 |
| NM_201655 | Tepp | testis, prostate and placenta-expressed protein |
| NM_031131 | Tgfb2 | transforming growth factor beta-2 precursor |
| NM_001100558 | Tiam1 | T-cell lymphoma invasion and metastasis 1 |
| NM_001172125 | Tlx2 | T-cell leukemia, homeobox 2 |
| NM_001107015 | Tm4sf5 | transmembrane 4 L6 family member 5 |
| NM_001191668 | Tmem185b | transmembrane protein 185B |
| NM_001017455 | Tmem80 | transmembrane protein 80 |
| NM_001105806 | Tmem93 | transmembrane protein 93 |
| NM_153311 | Tmprss5 | transmembrane protease serine 5 |
| NR_024118 | Tnxa |  |
| NM_130420 | Trim9 | E3 ubiquitin-protein ligase TRIM9 |
| NM_012808 | Tst | thiosulfate sulfurtransferase |
| NM_001025675 | Tubb6 | tubulin, beta 6 |
| NM_001039163 | Tusc5 | tumor suppressor candidate 5 homolog |
| NM_001105723 | Ubtf | nucleolar transcription factor 1 isoform 1 |
| NM_001077660 | Urg4 | up-regulated gene 4 |
| NM_022637 | Vax2 | ventral anterior homeobox 2 |
| NM_001169128 | Vsx2 | visual system homeobox 2 |
| NM_001135894 | Wdr25l | WD repeat domain 25-like |
| NM_001110489 | Wdr86 | WD repeat-containing protein 86 |
| NM_001191556 | Wnk2 | serine/threonine-protein kinase WNK2 |
| NM_001108227 | Wnt10a | protein Wnt-10a |
| NM_001105783 | Wnt9a | protein Wnt-9a |
| NM_001106184 | Wwp2 | NEDD4-like E3 ubiquitin-protein ligase WWP2 |
| NM_199383 | Yipf1 | protein YIPF1 |
| NM_001014208 | Yipf2 | protein YIPF2 |
| NM_001025747 | Yipf6 | protein YIPF6 |
| NM_175604 | Yrdc | yrdC domain-containing protein, mitochondrial |
| NM_019377 | Ywhab | 14-3-3 protein beta/alpha |
| NM_001130537 | Zbtb39 | zinc finger and BTB domain-containing protein |
| NM_001170577 | Zfp167 | zinc finger protein 167 |
| NM_001108725 | Zfyve21 | zinc finger FYVE domain-containing protein 21 |
| NM_203369 | Zmynd11 | zinc finger MYND domain-containing protein 11 |
| NM_001024878 | Znrf4 | zinc/RING finger protein 4 |
| NM_031616 | Zranb2 | zinc finger Ran-binding domain-containing |

**Dataset S4. List of genes differentially regulated by H3K27me3 upon Pb exposure**

| **Accession** | **Symbol** | **Gene Name** |
| --- | --- | --- |
| NM_012690 | Abcb4 | multidrug resistance protein 2 |
| NM_080582 | Abcb6 | ATP-binding cassette sub-family B member 6, |
| NM_001005902 | Abtb1 | ankyrin repeat and BTB/POZ domain-containing |
| NM_001126079 | Acbd7 | acyl-CoA-binding domain-containing protein 7 |
| NM_001106508 | Acoxl | acyl-coenzyme A oxidase-like protein |
| NM_001134956 | Ahdc1 | A.T hook DNA-binding motif-containing protein 1 |
| NM_030986 | Ak2 | adenylate kinase 2, mitochondrial isoform a |
| NM_001033967 | Ak2 | adenylate kinase 2, mitochondrial isoform b |
| NM_053896 | Aldh1a2 | retinal dehydrogenase 2 |
| NM_153301 | Alox15b | arachidonate 15-lipoxygenase B |
| NM_013059 | Alpl | alkaline phosphatase, tissue-nonspecific isozyme |
| NM_001134699 | Ankrd40 | ankyrin repeat domain-containing protein 40 |
| NM_001009676 | Anks3 | ankyrin repeat and SAM domain-containing protein |
| NM_001008523 | Aox4 | aldehyde oxidase 4 |
| NM_001108331 | Ap1s1 | adaptor protein complex AP-1, sigma 1 |
| NM_012500 | Apeh | acylamino-acid-releasing enzyme |
| NM_001004242 | Arhgap8 | rho GTPase-activating protein 8 |
| NM_001080789 | Arhgap9 | rho GTPase-activating protein 9 isoform 1 |
| NM_001012198 | Arhgap9 | rho GTPase-activating protein 9 isoform 2 |
| NM_001013108 | Arih1 | ariadne ubiquitin-conjugating enzyme E2 binding |
| NM_001106919 | Arpc2 | actin-related protein 2/3 complex subunit 2 |
| NM_001037767 | Arpc5l | actin-related protein 2/3 complex subunit 5-like |
| NM_198735 | Art2b | ADP-ribosyltransferase 2b |
| NM_053397 | Artn | artemin precursor |
| NM_012914 | Atp2a3 | sarcoplasmic/endoplasmic reticulum calcium |
| NM_001025767 | Blnk | B-cell linker protein |
| NM_001128187 | Bnipl | BCL2/adenovirus E1B 19kD interacting protein |
| NM_017259 | Btg2 | protein BTG2 |
| NM_001166344 | Btnl4 | butyrophilin subfamily 3 member A2 |
| NM_212489 | Btnl8 | butyrophilin-like 8 |
| NM_001107404 | Cables1 | CDK5 and ABL1 enzyme substrate 1 |
| NM_080694 | Cacng6 | voltage-dependent calcium channel gamma-6 |
| NM_138513 | Calcb | calcitonin gene-related peptide 2 precursor |
| NM_012518 | Calm3 | calmodulin |
| NM_019174 | Car4 | carbonic anhydrase 4 precursor |
| NM_001130554 | Card10 | caspase recruitment domain-containing protein |
| NM_001013191 | Cbfb | core-binding factor subunit beta |
| NM_199117 | Cbx7 | chromobox protein homolog 7 |
| NM_001105725 | Ccng2 | cyclin-G2 |
| NM_001166577 | Cd300e | CMRF35-like molecule 2 |
| NM_017124 | Cd37 | leukocyte antigen CD37 |
| NM_013169 | Cd3d | T-cell surface glycoprotein CD3 delta chain |
| NM_001077646 | Cd3g | T-cell surface glycoprotein CD3 gamma chain |
| NM_134360 | Cd40 | tumor necrosis factor receptor superfamily |
| NM_001015016 | Cd72 | B-cell differentiation antigen CD72 |
| NM_001013103 | Cdc34 | ubiquitin-conjugating enzyme Cdc34 |
| NM_053620 | Cdc42bpb | serine/threonine-protein kinase MRCK beta |
| NM_001048044 | Cdc42ep3 | CDC42 effector protein (Rho GTPase binding) 3 |
| NM_138889 | Cdh13 | cadherin-13 |
| NM_053891 | Cdk5r1 | cyclin-dependent kinase 5 activator 1 |
| NM_012831 | Cebpg | CCAAT/enhancer-binding protein gamma |
| NM_001105900 | Cggbp1 | CGG triplet repeat-binding protein 1 |
| NM_001170593 | Chat | choline O-acetyltransferase |
| NM_017127 | Chka | choline kinase alpha |
| NM_001011955 | Chst1 | carbohydrate sulfotransferase 1 |
| NM_031702 | Cldn7 | claudin-7 |
| NM_031818 | Clic4 | chloride intracellular channel protein 4 |
| NM_022218 | Cmklr1 | chemokine-like receptor 1 |
| NM_001011942 | Cnnm2 | metal transporter CNNM2 |
| NM_001014232 | Cnrip1 | CB1 cannabinoid receptor-interacting protein 1 |
| NM_001107236 | Cobl | protein cordon-bleu |
| NM_001108710 | Coch | cochlin |
| NM_001025721 | Colec12 | collectin-12 |
| NM_031766 | Cpz | carboxypeptidase Z precursor |
| NM_001105716 | Crabp1 | cellular retinoic acid-binding protein 1 |
| NM_001004085 | Crat | carnitine O-acetyltransferase |
| NM_001024783 | Creld1 | cysteine-rich with EGF-like domain protein 1 |
| NM_001014258 | Crls1 | cardiolipin synthase |
| NM_017074 | Cth | cystathionine gamma-lyase |
| NM_001100661 | Ctr9 | Ctr9, Paf1/RNA polymerase II complex component, |
| NM_013156 | Ctsl1 | cathepsin L1 preproprotein |
| NM_022297 | Ddah1 | N(G),N(G)-dimethylarginine |
| NM_030993 | Ddn | dendrin |
| NM_001109577 | Derl3 | derlin-3 |
| NM_032063 | Dll1 | delta-like protein 1 precursor |
| NM_001105832 | Dlx3 | distal-less homeobox 3 |
| NM_012943 | Dlx5 | homeobox protein DLX-5 |
| NM_053706 | Dmrt1 | doublesex- and mab-3-related transcription |
| NM_001107597 | Dmrt2 | doublesex- and mab-3-related transcription |
| NM_001105759 | Dock9 | dedicator of cytokinesis protein 9 |
| NM_012546 | Drd1a | D(1A) dopamine receptor |
| NM_024141 | Duox2 | dual oxidase 2 precursor |
| NM_001191965 | Duoxa2 | dual oxidase maturation factor 2 |
| NM_001037973 | Dusp9 | dual specificity protein phosphatase 9 |
| NM_012842 | Egf | pro-epidermal growth factor precursor |
| NM_001008773 | Eif1a | eukaryotic translation initiation factor 1A |
| NM_001107602 | Elovl3 | elongation of very long chain fatty acids |
| NM_001009391 | Enoph1 | enolase-phosphatase E1 |
| NM_021687 | Erbb4 | receptor tyrosine-protein kinase erbB-4 |
| NM_022604 | Esm1 | endothelial cell-specific molecule 1 precursor |
| NM_012555 | Ets1 | protein C-ets-1 |
| NM_133537 | Expi | extracellular peptidase inhibitor |
| NM_001109885 | Fam129b | niban-like protein 1 |
| NM_001012238 | Fam20c | dentin matrix protein 4 |
| NM_001106296 | Fam57b | hypothetical protein LOC293493 |
| NM_001014046 | Fam82a2 | regulator of microtubule dynamics protein 3 |
| NM_022272 | Fbxl20 | F-box/LRR-repeat protein 20 |
| NM_001107203 | Fbxo28 | F-box only protein 28 |
| NM_001011998 | Fbxo9 | F-box only protein 9 |
| NM_001107600 | Fbxw4 | F-box/WD repeat-containing protein 4 |
| NM_053843 | Fcgr2a | low affinity immunoglobulin gamma Fc region |
| NM_001100682 | Fcrla | Fc receptor-like A precursor |
| NM_144753 | Fev | protein FEV |
| NM_001109224 | Fezf1 | fez family zinc finger protein 1 |
| NM_130752 | Fgf21 | fibroblast growth factor 21 |
| NM_130817 | Fgf3 | fibroblast growth factor 3 |
| NM_001106484 | Fign | fidgetin |
| NM_001168584 | Foxb2 | forkhead box B2 |
| NM_024366 | Freq | neuronal calcium sensor 1 |
| NM_001025738 | Fusip1 | FUS interacting protein (serine-arginine rich) |
| NM_031236 | Fut1 | galactoside 2-alpha-L-fucosyltransferase 1 |
| NM_022005 | Fxyd6 | FXYD domain-containing ion transport regulator 6 |
| NM_001039036 | Gabpb1 | GA repeat binding protein, beta 1 |
| NM_012563 | Gad2 | glutamate decarboxylase 2 |
| NM_001025053 | Galnt4 | polypeptide N-acetylgalactosaminyltransferase 4 |
| NM_001122644 | Galnt9 | polypeptide N-acetylgalactosaminyltransferase 9 |
| NM_133293 | Gata3 | GATA binding protein 3 |
| NM_144730 | Gata4 | transcription factor GATA-4 |
| NM_019185 | Gata6 | transcription factor GATA-6 |
| NM_001004273 | Ggnbp2 | gametogenetin-binding protein 2 |
| NM_001004099 | Gjb2 | gap junction beta-2 protein |
| NM_013133 | Glra1 | glycine receptor subunit alpha-1 |
| NM_001134413 | Gltp | glycolipid transfer protein |
| NM_053765 | Gne | bifunctional UDP-N-acetylglucosamine |
| NM_001007720 | Gorasp2 | Golgi reassembly-stacking protein 2 |
| NM_001014108 | Gpc4 | glypican-4 |
| NM_001034855 | Gpr153 | probable G-protein coupled receptor 153 |
| NM_017010 | Grin1 | glutamate [NMDA] receptor subunit zeta-1 |
| NM_001109270 | Grrp1 | glycine/arginine-rich protein 1 |
| NM_001191873 | Gsc | homeobox protein goosecoid |
| NM_032080 | Gsk3b | glycogen synthase kinase-3 beta |
| NM_001003978 | Gspt1 | eukaryotic peptide chain release factor |
| NM_001001512 | Gtf2i | general transcription factor II-I |
| NM_023956 | Gucy1a2 | guanylate cyclase soluble subunit alpha-2 |
| NM_022674 | H2afz | histone H2A.Z |
| NM_022696 | Hand2 | heart- and neural crest derivatives-expressed |
| NM_001012074 | Herc4 | probable E3 ubiquitin-protein ligase HERC4 |
| NM_001100986 | Hipk1 | homeodomain-interacting protein kinase 1 |
| NM_031787 | Hipk3 | homeodomain-interacting protein kinase 3 |
| NM_001106303 | Hmx2 | homeobox protein HMX2 |
| NM_031330 | Hnrnpab | heterogeneous nuclear ribonucleoprotein A/B |
| NM_001033696 | Hnrpdl | heterogeneous nuclear ribonucleoprotein D-like |
| NM_001191087 | Hoxa6 | homeobox protein Hox-A6 |
| NM_001109233 | Hoxa9 | homeobox protein Hox-A7 |
| NM_001107042 | Hoxb3 | homeo box B3 |
| NM_001100787 | Hoxb4 | homeo box B4 |
| NM_001191925 | Hoxb5 | homeo box B5 |
| NM_001017480 | Hoxb7 | homeobox protein Hox-B7 |
| NM_001100497 | Hoxb9 | homeo box B9 |
| NM_001106796 | Hoxc12 | homeobox protein Hox-C12 |
| NM_001105884 | Hoxd1 | homeobox protein Hox-D1 |
| NM_017122 | Hpca | neuron-specific calcium-binding protein |
| NM_001135762 | Hpse2 | heparanase-2 |
| NM_181370 | Hs3st2 | heparan sulfate glucosamine 3-O-sulfotransferase |
| NM_001106177 | Hsf4 | heat shock factor protein 4 |
| NM_024395 | Htr5b | 5-hydroxytryptamine receptor 5B |
| NM_031721 | Htra1 | serine protease HTRA1 |
| NM_001107321 | Htra4 | probable serine protease HTRA4 |
| NM_001082477 | Igf1 | insulin-like growth factor I isoform a |
| NM_001107197 | Igsf9 | protein turtle homolog A precursor |
| NM_001107237 | Ikzf1 | IKAROS family zinc finger 1 |
| NM_001107521 | Il20ra | interleukin-20 receptor subunit alpha |
| NM_133409 | Ilk | integrin-linked protein kinase |
| NM_001106083 | Ing2 | inhibitor of growth protein 2 |
| NM_207617 | Iqsec3 | IQ motif and SEC7 domain-containing protein 3 |
| NM_001025422 | Irak2 | interleukin-1 receptor-associated kinase-like 2 |
| NM_181626 | Isca1 | iron-sulfur cluster assembly 1 homolog, |
| NM_001014242 | Isoc1 | isochorismatase domain-containing protein 1 |
| NM_001106630 | Jph1 | junctophilin-1 |
| NM_001107437 | Jph3 | junctophilin-3 |
| NM_031047 | Jup | junction plakoglobin |
| NM_001008814 | Kb21 | keratin, type II cuticular Hb1 |
| NM_019270 | Kcna3 | potassium voltage-gated channel subfamily A |
| NM_053630 | Kcnh4 | potassium voltage-gated channel subfamily H |
| NM_145095 | Kcnh8 | potassium voltage-gated channel subfamily H |
| NM_031358 | Kcnj11 | ATP-sensitive inward rectifier potassium channel |
| NM_130813 | Kcnk15 | potassium channel subfamily K member 15 |
| NM_001039516 | Kcnk5 | potassium channel subfamily K member 5 |
| NM_031597 | Kcnq3 | potassium voltage-gated channel subfamily KQT |
| NM_001109079 | Kif26b | kinesin family member 26B |
| NM_001107164 | Klf1 | Krueppel-like factor 1 |
| NM_057211 | Klf9 | Krueppel-like factor 9 |
| NM_001106735 | Klhl28 | kelch-like protein 28 |
| NM_001109326 | Krtap14 | keratin-associated protein 14 |
| NM_053538 | Laptm5 | lysosomal-associated transmembrane protein 5 |
| NM_001007556 | Lefty2 | left-right determination factor 2 |
| NM_133393 | Lfng | beta-1,3-N-acetylglucosaminyltransferase lunatic |
| NM_139036 | Lhx5 | LIM/homeobox protein Lhx5 |
| NM_001107837 | Lhx6 | LIM/homeobox protein Lhx6 |
| NM_001100722 | Lingo1 | leucine rich repeat and Ig domain containing 1 |
| NM_053905 | Lmnb1 | lamin-B1 |
| NM_001103356 | LOC100125364 | hypothetical protein LOC100125364 precursor |
| NM_001013941 | LOC298795 | hypothetical protein LOC298795 |
| NM_001014007 | LOC306766 | hypothetical protein LOC306766 |
| NM_001014115 | LOC360479 | hypothetical protein LOC360479 |
| NM_001135992 | LOC498276 | Fc gamma receptor II beta |
| NM_001109221 | LOC500034 | hypothetical protein LOC500034 |
| NM_001195277 | LOC679651 | transmembrane protein 178-like |
| NM_001109489 | LOC685964 | hypothetical protein LOC685964 |
| NM_001109627 | LOC691153 | hypothetical protein LOC691153 |
| NM_001008519 | Lrpprc | leucine-rich PPR motif-containing protein, |
| NM_001170434 | Lrrc32 | leucine rich repeat containing 32 |
| NM_017242 | Lsamp | limbic system-associated membrane protein |
| NM_021656 | Ltb4r | leukotriene B4 receptor 1 |
| NM_001109391 | Mab21l2 | protein mab-21-like 2 |
| NM_139084 | Magi3 | membrane-associated guanylate kinase, WW and PDZ |
| NM_181089 | MAST1 | microtubule-associated serine/threonine-protein |
| NM_001039005 | Mcoln2 | mucolipin-2 |
| NM_001107618 | Mdga1 | MAM domain-containing |
| NM_030859 | Mdk | midkine precursor |
| NM_001191727 | Med14 | mediator of RNA polymerase II transcription |
| NM_030860 | Mef2d | myocyte-specific enhancer factor 2D |
| NM_001108837 | Meox1 | homeobox protein MOX-1 |
| NM_017149 | Meox2 | homeobox protein MOX-2 |
| NM_001107531 | Mesp1 | mesoderm posterior protein 1 |
| NM_001191626 | Mex3b | RNA-binding protein MEX3B |
| NM_001008518 | MGC105649 | normal mucosa of esophagus-specific gene 1 |
| NM_001044292 | MGC116202 | hypothetical protein LOC688736 |
| NM_001007746 | MGC94199 | hypothetical protein LOC362483 |
| NR_031814 | Mir10a |  |
| NR_031883 | Mir137 |  |
| NR_037325 | Mir3549 |  |
| NM_001108425 | Mocos | molybdenum cofactor sulfurase |
| NM_020102 | Mos | proto-oncogene serine/threonine-protein kinase |
| NM_001191558 | Mtss1l | MTSS1-like protein |
| NM_001100667 | Mtx1 | metaxin 1 |
| NM_057209 | Mylk2 | myosin light chain kinase 2, skeletal/cardiac |
| NM_053888 | Myt1l | myelin transcription factor 1-like protein |
| NM_001109678 | Nadk | NAD kinase |
| NM_001014785 | Ncbp1 | nuclear cap-binding protein subunit 1 |
| NM_053691 | Nek2 | NIMA-related kinase 2 |
| NM_001013134 | Nek4 | serine/threonine-protein kinase Nek4 |
| NM_012987 | Nes | nestin |
| NM_031789 | Nfe2l2 | nuclear factor erythroid 2-related factor 2 |
| NM_012865 | Nfya | nuclear transcription factor Y subunit alpha |
| NM_001170476 | Nkx1-2 | NK1 homeobox 2 |
| NM_001107594 | Nkx2-3 | homeobox protein Nkx-2.3 |
| NM_053651 | Nkx2-5 | homeobox protein Nkx-2.5 |
| NM_001024360 | nod3l | hypothetical protein LOC501101 |
| NM_001007800 | N-pac | putative oxidoreductase GLYR1 |
| NM_203340 | Npm2 | nucleoplasmin-2 |
| NM_024388 | Nr4a1 | nuclear receptor subfamily 4 group A member 1 |
| NM_001100708 | Nrf1 | nuclear respiratory factor 1 |
| NM_053731 | Ntn1 | netrin-1 precursor |
| NM_001025708 | Ogfrl1 | opioid growth factor receptor-like protein 1 |
| NM_001106269 | Olig3 | oligodendrocyte transcription factor 2 |
| NM_001191700 | Otud4 | OTU domain-containing protein 4 |
| NM_001037496 | Otud5 | OTU domain-containing protein 5 |
| NM_012721 | P2rx6 | P2X purinoceptor 6 |
| NM_001009966 | Pacsin3 | protein kinase C and casein kinase substrate in |
| NM_001108937 | Paip1 | polyadenylate-binding protein-interacting |
| NM_033485 | Pawr | PRKC apoptosis WT1 regulator protein |
| NM_001107787 | Pax1 | paired box protein Pax-1 |
| NM_053710 | Pax3 | paired box 3 |
| NM_001039539 | Pax9 | paired box protein Pax-9 |
| NM_012802 | Pdgfra | alpha-type platelet-derived growth factor |
| NM_130401 | Pdzk1ip1 | PDZK1-interacting protein 1 |
| NM_001013231 | Pea15a | astrocytic phosphoprotein PEA-15 |
| NM_001109487 | Pfn3 | profilin-3 |
| NM_017034 | Pim1 | proto-oncogene serine/threonine-protein kinase |
| NM_053624 | Pitx1 | pituitary homeobox 1 |
| NM_019334 | Pitx2 | pituitary homeobox 2 isoform 2 |
| NM_017175 | Pkn1 | serine/threonine-protein kinase N1 |
| NM_022533 | Pllp | plasmolipin |
| NM_001142915 | Plod2 | procollagen-lysine,2-oxoglutarate 5-dioxygenase |
| NM_001107922 | Pm20d2 | peptidase M20 domain-containing protein 2 |
| NM_001108889 | Pou4f3 | POU class 4 homeobox 3 |
| NM_176075 | Ppargc1b | peroxisome proliferator-activated receptor gamma |
| NM_198773 | Ppm1e | protein phosphatase 1E |
| NM_001191072 | Ppp1r16b | protein phosphatase 1 regulatory inhibitor |
| NM_001108577 | Ppp2r4 | serine/threonine-protein phosphatase 2A |
| NM_181379 | Ppp2r5b | serine/threonine-protein phosphatase 2A 56 kDa |
| NM_017309 | Ppp3r1 | calcineurin subunit B type 1 |
| NM_001106613 | Ppp4r2 | protein phosphatase 4, regulatory subunit 2 |
| NM_021751 | Prom1 | prominin 1 isoform 1 |
| NM_001012121 | Prr5 | proline-rich protein 5 |
| NM_001109226 | Prrt4 | proline-rich transmembrane protein 4 |
| NM_053566 | Ptch1 | protein patched homolog 1 |
| NM_053964 | Ptf1a | pancreas transcription factor 1 subunit alpha |
| NM_020073 | Pth1r | parathyroid hormone/parathyroid hormone-related |
| NM_031579 | Ptp4a1 | protein tyrosine phosphatase type IVA 1 |
| NM_001108684 | Pum1 | pumilio homolog 1 |
| NM_001106715 | Pum2 | pumilio homolog 2 |
| NM_001108962 | R3hdml | R3H domain (binds single-stranded nucleic acids) |
| NM_031152 | Rab11a | ras-related protein Rab-11A |
| NM_053678 | Rax | retinal homeobox protein Rx |
| NM_013162 | Rbp4 | retinol-binding protein 4 precursor |
| NM_001127490 | Rfx7 | regulatory factor X domain containing 2 |
| NM_001134560 | RGD1305627 | hypothetical protein LOC314467 |
| NM_001108652 | RGD1306151 | hypothetical protein LOC362455 |
| NM_001106551 | RGD1306208 | hypothetical protein LOC296483 |
| NM_001017454 | RGD1307799 | IST1 homolog |
| NM_001134596 | RGD1308299 | hypothetical protein LOC367214 |
| NM_001108286 | RGD1311564 | hypothetical protein LOC360590 |
| NM_001109262 | RGD1559493 | hypothetical protein LOC500516 |
| NM_001109345 | RGD1563349 | hypothetical protein LOC502727 |
| NM_001134589 | RGD1566265 | hypothetical protein LOC363487 |
| NM_001030034 | Rhbdf1 | rhomboid family member 1 |
| NM_001013133 | Rhobtb2 | rho-related BTB domain-containing protein 2 |
| NM_001191665 | Rilpl1 | RILP-like protein 1 |
| NM_001100488 | Rimbp2 | RIMS-binding protein 2 |
| NM_001108052 | Rin3 | ras and Rab interactor 3 |
| NM_001107118 | Rnf6 | RING finger protein 6 |
| NM_001025740 | Rrm2 | ribonucleoside-diphosphate reductase subunit M2 |
| NM_001008346 | Rrp8 | ribosomal RNA-processing protein 8 |
| NM_001008827 | RT1-A1 | RT1 class Ia, locus A1 |
| NM_001008832 | RT1-CE1 | RT1 class I, locus CE1 |
| NM_001008833 | RT1-CE10 | RT1 class I, locus CE10 |
| NM_001033985 | RT1-CE14 | RT1 class I, locus CE14 isoform 2 |
| NM_012645 | RT1-EC2 | class I histocompatibility antigen, Non-RT1.A |
| NM_001109471 | S100a7a | protein S100-A15A |
| NM_001013985 | Sccpdh | probable saccharopine dehydrogenase |
| NM_198748 | Scin | adseverin |
| NM_017247 | Scn10a | sodium channel protein type 10 subunit alpha |
| NM_012648 | Scnn1b | amiloride-sensitive sodium channel subunit beta |
| NM_022670 | Sct | secretin precursor |
| NM_001107637 | Sec63 | translocation protein SEC63 homolog |
| NM_001166396 | Selv | selenoprotein V |
| NM_001107091 | Sema5b | sema domain, seven thrombospondin repeats (type |
| NM_017308 | Sema6c | semaphorin-6C precursor |
| NM_001137647 | Sh3bgrl2 | SH3 domain-binding glutamic acid-rich-like |
| NM_001191936 | Shisa2 | protein shisa-2 homolog |
| NM_001191922 | Shisa6 | protein shisa-6 homolog |
| NM_134457 | Siah2 | E3 ubiquitin-protein ligase SIAH2 |
| NM_001107641 | Sim1 | single-minded homolog 1 |
| NM_001004089 | Sipa1 | signal-induced proliferation-associated protein |
| NM_053759 | Six1 | sine oculis-related homeobox 1 homolog |
| NM_023990 | Six3 | homeobox protein SIX3 |
| NM_001013144 | Slc12a7 | solute carrier family 12 member 7 |
| NM_012716 | Slc16a1 | monocarboxylate transporter 1 |
| NM_053427 | Slc17a6 | vesicular glutamate transporter 2 |
| NM_031663 | Slc18a3 | vesicular acetylcholine transporter |
| NM_177421 | Slc22a17 | solute carrier family 22 member 17 |
| NM_019230 | Slc22a3 | solute carrier family 22 member 3 |
| NM_017316 | Slc23a2 | solute carrier family 23 member 2 |
| NM_001108051 | Slc24a4 | sodium/potassium/calcium exchanger 4 |
| NM_001013996 | Slc25a37 | mitoferrin-1 |
| NM_019214 | Slc26a4 | pendrin |
| NM_031736 | Slc27a2 | very long-chain acyl-CoA synthetase |
| NM_001107522 | Slc35d3 | solute carrier family 35 member D3 |
| NM_001134687 | Slc35e3 | solute carrier family 35 member E3 |
| NM_053424 | Slc4a4 | electrogenic sodium bicarbonate cotransporter 1 |
| NM_203334 | Slc6a5 | sodium- and chloride-dependent glycine |
| NM_078620 | Slc8a3 | sodium/calcium exchanger 3 precursor |
| NM_022953 | Slit1 | slit homolog 1 protein precursor |
| NM_013095 | Smad3 | mothers against decapentaplegic homolog 3 |
| NM_001107419 | Smarca5 | SWI/SNF-related matrix-associated |
| NM_001108752 | Smarcd1 | SWI/SNF-related matrix-associated |
| NM_001106832 | Snx22 | sorting nexin-22 |
| NM_001107902 | Sox17 | transcription factor SOX-17 |
| NM_031792 | Spag4 | sperm-associated antigen 4 protein |
| NM_001106125 | Spag6l | sperm associated antigen 6-like |
| NM_199374 | Spata18 | spermatogenesis-associated protein 18 |
| NM_133386 | Sphk1 | sphingosine kinase 1 |
| NM_175843 | Sqstm1 | sequestosome-1 isoform 1 |
| NM_181550 | Sqstm1 | sequestosome-1 isoform 2 |
| NM_012659 | Sst | somatostatin precursor |
| NM_012747 | Stat3 | signal transducer and activator of transcription |
| NM_001108883 | Suv39h2 | histone-lysine N-methyltransferase SUV39H2 |
| NM_022191 | Syt6 | synaptotagmin-6 |
| NM_001004107 | Tacc1 | transforming, acidic coiled-coil containing |
| NM_001170455 | Tada2b | transcriptional adaptor 2B |
| NM_001013127 | Tagln2 | transgelin-2 |
| NM_001013245 | Tbca | tubulin-specific chaperone A |
| NM_001191070 | Tbr1 | T-box brain protein 1 |
| NM_001108322 | Tbx1 | T-box transcription factor TBX1 |
| NM_001108132 | Tbx20 | T-box 20 |
| NM_001107034 | Tbx4 | T-box transcription factor TBX4 |
| NM_001009964 | Tbx5 | T-box transcription factor TBX5 |
| NM_001130077 | Tcerg1l | transcription elongation regulator 1-like |
| NM_001032397 | Tcf21 | transcription factor 21 |
| NM_001106896 | Tcfap2b | transcription factor AP-2-beta |
| NM_019194 | Tef | thyrotroph embryonic factor |
| NM_012671 | Tgfa | protransforming growth factor alpha |
| NM_031132 | Tgfbr2 | TGF-beta receptor type-2 precursor |
| NM_001105758 | Tmed7 | transmembrane emp24 domain-containing protein 7 |
| NM_001108795 | Tmeff2 | tomoregulin-2 |
| NM_001106280 | Tmem126b | transmembrane protein 126B |
| NM_001134410 | Tmem132e | transmembrane protein 132E |
| NM_001107476 | Tmem150b | transmembrane protein 150B |
| NM_001109480 | Tmem229a | transmembrane protein 229A |
| NM_001108045 | Tmem63c | transmembrane protein 63C |
| NM_001127528 | Tmprss13 | transmembrane protease serine 13 |
| NM_001108998 | Tmprss4 | transmembrane protease serine 4 |
| NM_001108873 | Tnfrsf10b | tumor necrosis factor receptor superfamily, |
| NM_001191810 | Tns1 | tensin 1 |
| NM_001105818 | Tp53i13 | tumor protein p53-inducible protein 13 |
| NM_013046 | Trh | prothyroliberin |
| NM_053916 | Trim28 | transcription intermediary factor 1-beta |
| NM_031786 | Trim3 | tripartite motif-containing protein 3 |
| NM_001106453 | Trim45 | tripartite motif-containing protein 45 |
| NM_001109912 | Tsc22d1 | TSC22 domain family protein 1 isoform 1 |
| NM_001109227 | Tspan33 | tetraspanin-33 |
| NM_001108663 | Tstd2 | thiosulfate sulfurtransferase/rhodanese-like |
| NM_001109119 | Tubb2a | tubulin beta-2A chain |
| NM_001037643 | Ube2z | ubiquitin-conjugating enzyme E2 Z |
| NM_001003709 | Ufc1 | ubiquitin-fold modifier-conjugating enzyme 1 |
| NM_145184 | Usp15 | ubiquitin carboxyl-terminal hydrolase 15 |
| NM_001106120 | Usp6nl | USP6 N-terminal-like protein |
| NM_013155 | Vldlr | very low-density lipoprotein receptor precursor |
| NM_001109312 | Vwc2 | brorin |
| NM_175579 | Wnk4 | serine/threonine-protein kinase WNK4 |
| NM_001191848 | Wnt2b | protein Wnt-2b |
| NM_001108226 | Wnt6 | protein Wnt-6 |
| NM_001107055 | Wnt9b | protein Wnt-9b |
| NM_022231 | Xiap | baculoviral IAP repeat-containing protein 4 |
| NM_001105992 | Xpr1 | xenotropic and polytropic retrovirus receptor 1 |
| NM_022296 | Xylt2 | xylosyltransferase 2 |
| NM_001034831 | Zfp384 | zinc finger protein 384 isoform 2 |
| NM_001034830 | Zfp384 | zinc finger protein 384 isoform 1 |
| NM_001109470 | Zfp385a | zinc finger protein 385A isoform 2 |
| NM_001012093 | Zfp64 | zinc finger protein 64 |
| NM_001109225 | Zfp800 | zinc finger protein 800 |
| NM_001030038 | Znf518a | zinc finger protein 518A |
| NM_053761 | Zyx | zyxin |

**Dataset S5. Enriched GO terms upon Pb exposure**

| **Category** | **Term** | **Count** | **%** | **P-Value** |
| --- | --- | --- | --- | --- |
| SP_PIR_KEYWORDS | phosphoprotein | 165 | 27.1 | 1.40E-04 |
| SP_PIR_KEYWORDS | membrane | 147 | 24.1 | 5.20E-02 |
| GOTERM_CC_FAT | plasma membrane | 115 | 18.9 | 3.90E-06 |
| SP_PIR_KEYWORDS | nucleus | 87 | 14.3 | 3.60E-03 |
| GOTERM_BP_FAT | regulation of transcription | 86 | 14.1 | 3.90E-06 |
| GOTERM_MF_FAT | nucleotide binding | 82 | 13.5 | 1.10E-02 |
| SP_PIR_KEYWORDS | cytoplasm | 82 | 13.5 | 3.20E-02 |
| GOTERM_MF_FAT | DNA binding | 70 | 11.5 | 7.80E-05 |
| GOTERM_MF_FAT | purine ribonucleotide binding | 69 | 11.3 | 6.20E-03 |
| GOTERM_MF_FAT | ribonucleotide binding | 69 | 11.3 | 6.40E-03 |
| GOTERM_MF_FAT | purine nucleotide binding | 69 | 11.3 | 1.80E-02 |
| GOTERM_CC_FAT | plasma membrane part | 66 | 10.8 | 7.40E-04 |
| GOTERM_MF_FAT | transcription regulator activity | 64 | 10.5 | 1.20E-06 |
| GOTERM_BP_FAT | regulation of RNA metabolic process | 62 | 10.2 | 1.10E-03 |
| SP_PIR_KEYWORDS | transport | 60 | 9.9 | 1.30E-02 |
| GOTERM_BP_FAT | regulation of transcription, DNA-dependent | 59 | 9.7 | 2.60E-03 |
| GOTERM_MF_FAT | purine nucleoside binding | 58 | 9.5 | 2.90E-02 |
| GOTERM_MF_FAT | nucleoside binding | 58 | 9.5 | 3.40E-02 |
| GOTERM_MF_FAT | adenyl ribonucleotide binding | 57 | 9.4 | 1.10E-02 |
| GOTERM_MF_FAT | adenyl nucleotide binding | 57 | 9.4 | 3.10E-02 |
| GOTERM_MF_FAT | ATP binding | 56 | 9.2 | 1.10E-02 |
| GOTERM_CC_FAT | membrane-enclosed lumen | 56 | 9.2 | 1.20E-02 |
| SP_PIR_KEYWORDS | transcription regulation | 55 | 9 | 1.20E-08 |
| SP_PIR_KEYWORDS | Transcription | 55 | 9 | 1.50E-07 |
| GOTERM_BP_FAT | positive regulation of macromolecule metabolic process | 55 | 9 | 7.70E-07 |
| GOTERM_CC_FAT | cytosol | 55 | 9 | 1.80E-03 |
| GOTERM_CC_FAT | organelle lumen | 55 | 9 | 1.10E-02 |
| SP_PIR_KEYWORDS | cell membrane | 54 | 8.9 | 8.80E-02 |
| GOTERM_BP_FAT | transcription | 53 | 8.7 | 1.10E-08 |
| GOTERM_CC_FAT | intracellular organelle lumen | 53 | 8.7 | 1.30E-02 |
| SP_PIR_KEYWORDS | alternative splicing | 52 | 8.5 | 6.60E-03 |
| UP_SEQ_FEATURE | splice variant | 51 | 8.4 | 1.90E-02 |
| GOTERM_BP_FAT | intracellular signaling cascade | 48 | 7.9 | 1.70E-03 |
| GOTERM_CC_FAT | cytoskeleton | 48 | 7.9 | 1.80E-03 |
| GOTERM_CC_FAT | nuclear lumen | 46 | 7.6 | 4.00E-03 |
| GOTERM_BP_FAT | positive regulation of nucleobase, nucleoside, nucleotide and nucleic acid metabolic process | 44 | 7.2 | 1.60E-06 |
| GOTERM_BP_FAT | positive regulation of nitrogen compound metabolic process | 44 | 7.2 | 3.40E-06 |
| GOTERM_BP_FAT | positive regulation of biosynthetic process | 44 | 7.2 | 2.20E-05 |
| SP_PIR_KEYWORDS | dna-binding | 44 | 7.2 | 1.20E-04 |
| GOTERM_BP_FAT | positive regulation of macromolecule biosynthetic process | 43 | 7.1 | 8.60E-06 |
| GOTERM_MF_FAT | transcription factor activity | 43 | 7.1 | 1.20E-05 |
| GOTERM_BP_FAT | positive regulation of cellular biosynthetic process | 43 | 7.1 | 3.10E-05 |
| GOTERM_BP_FAT | phosphate metabolic process | 43 | 7.1 | 5.00E-03 |
| GOTERM_BP_FAT | phosphorus metabolic process | 43 | 7.1 | 5.10E-03 |
| SP_PIR_KEYWORDS | atp-binding | 42 | 6.9 | 3.90E-02 |
| GOTERM_BP_FAT | positive regulation of gene expression | 40 | 6.6 | 4.10E-06 |
| GOTERM_BP_FAT | positive regulation of transcription | 39 | 6.4 | 5.80E-06 |
| GOTERM_CC_FAT | nucleoplasm | 39 | 6.4 | 1.10E-03 |
| GOTERM_BP_FAT | regulation of transcription from RNA polymerase II promoter | 38 | 6.2 | 1.90E-04 |
| GOTERM_CC_FAT | cell projection | 38 | 6.2 | 4.20E-03 |
| GOTERM_BP_FAT | neuron differentiation | 37 | 6.1 | 1.00E-06 |
| GOTERM_BP_FAT | positive regulation of RNA metabolic process | 37 | 6.1 | 1.20E-06 |
| GOTERM_BP_FAT | phosphorylation | 37 | 6.1 | 6.90E-03 |
| GOTERM_CC_FAT | Golgi apparatus | 36 | 5.9 | 3.30E-03 |
| GOTERM_BP_FAT | positive regulation of transcription, DNA-dependent | 35 | 5.7 | 6.80E-06 |
| GOTERM_BP_FAT | ion transport | 35 | 5.7 | 7.40E-03 |
| GOTERM_MF_FAT | transcription activator activity | 33 | 5.4 | 6.50E-10 |
| GOTERM_BP_FAT | protein amino acid phosphorylation | 33 | 5.4 | 7.10E-03 |
| GOTERM_CC_FAT | cytoskeletal part | 33 | 5.4 | 2.90E-02 |
| GOTERM_MF_FAT | sequence-specific DNA binding | 32 | 5.3 | 4.60E-04 |
| GOTERM_BP_FAT | positive regulation of molecular function | 32 | 5.3 | 6.40E-04 |
| GOTERM_BP_FAT | regulation of cell proliferation | 32 | 5.3 | 3.10E-02 |
| GOTERM_MF_FAT | protein dimerization activity | 31 | 5.1 | 2.10E-03 |
| SP_PIR_KEYWORDS | kinase | 31 | 5.1 | 5.60E-03 |
| GOTERM_BP_FAT | negative regulation of macromolecule metabolic process | 31 | 5.1 | 3.30E-02 |
| GOTERM_BP_FAT | homeostatic process | 31 | 5.1 | 1.00E-01 |
| GOTERM_CC_FAT | vesicle | 30 | 4.9 | 3.70E-02 |
| GOTERM_CC_FAT | cytoplasmic vesicle | 29 | 4.8 | 3.00E-02 |
| UP_SEQ_FEATURE | nucleotide phosphate-binding region:ATP | 29 | 4.8 | 3.30E-02 |
| GOTERM_BP_FAT | protein localization | 29 | 4.8 | 9.50E-02 |
| GOTERM_BP_FAT | death | 28 | 4.6 | 6.70E-04 |
| GOTERM_BP_FAT | positive regulation of catalytic activity | 28 | 4.6 | 1.50E-03 |
| GOTERM_MF_FAT | enzyme binding | 28 | 4.6 | 5.50E-03 |
| GOTERM_MF_FAT | protein kinase activity | 28 | 4.6 | 2.80E-02 |
| SP_PIR_KEYWORDS | ion transport | 28 | 4.6 | 3.30E-02 |
| GOTERM_CC_FAT | endomembrane system | 28 | 4.6 | 9.60E-02 |
| GOTERM_BP_FAT | programmed cell death | 27 | 4.4 | 2.80E-04 |
| GOTERM_BP_FAT | positive regulation of transcription from RNA polymerase II promoter | 27 | 4.4 | 3.90E-04 |
| GOTERM_BP_FAT | cell death | 27 | 4.4 | 1.10E-03 |
| GOTERM_BP_FAT | regulation of phosphorus metabolic process | 27 | 4.4 | 1.20E-03 |
| GOTERM_BP_FAT | regulation of phosphate metabolic process | 27 | 4.4 | 1.20E-03 |
| GOTERM_CC_FAT | nucleoplasm part | 27 | 4.4 | 1.70E-03 |
| GOTERM_BP_FAT | response to endogenous stimulus | 27 | 4.4 | 5.90E-02 |
| GOTERM_BP_FAT | cell projection organization | 26 | 4.3 | 3.60E-04 |
| GOTERM_BP_FAT | apoptosis | 26 | 4.3 | 4.70E-04 |
| GOTERM_CC_FAT | neuron projection | 26 | 4.3 | 4.20E-03 |
| GOTERM_CC_FAT | cell junction | 26 | 4.3 | 4.40E-03 |
| SP_PIR_KEYWORDS | lipoprotein | 26 | 4.3 | 9.30E-03 |
| GOTERM_CC_FAT | membrane-bounded vesicle | 26 | 4.3 | 5.00E-02 |
| GOTERM_BP_FAT | regulation of cell development | 25 | 4.1 | 7.50E-07 |
| GOTERM_MF_FAT | protein domain specific binding | 25 | 4.1 | 3.20E-04 |
| GOTERM_BP_FAT | neuron development | 25 | 4.1 | 4.80E-04 |
| GOTERM_BP_FAT | response to hormone stimulus | 25 | 4.1 | 4.90E-02 |
| GOTERM_CC_FAT | cytoplasmic membrane-bounded vesicle | 25 | 4.1 | 5.20E-02 |
| GOTERM_BP_FAT | regulation of phosphorylation | 24 | 3.9 | 6.50E-03 |
| GOTERM_BP_FAT | negative regulation of gene expression | 24 | 3.9 | 1.90E-02 |
| GOTERM_BP_FAT | negative regulation of nucleobase, nucleoside, nucleotide and nucleic acid metabolic process | 24 | 3.9 | 2.50E-02 |
| GOTERM_BP_FAT | negative regulation of nitrogen compound metabolic process | 24 | 3.9 | 2.90E-02 |
| GOTERM_BP_FAT | intracellular transport | 24 | 3.9 | 5.60E-02 |
| SP_PIR_KEYWORDS | activator | 23 | 3.8 | 5.30E-06 |
| GOTERM_BP_FAT | neuron projection development | 23 | 3.8 | 9.60E-05 |
| GOTERM_BP_FAT | regulation of cellular protein metabolic process | 23 | 3.8 | 1.90E-02 |
| GOTERM_BP_FAT | chemical homeostasis | 23 | 3.8 | 8.80E-02 |
| GOTERM_BP_FAT | regulation of neurogenesis | 22 | 3.6 | 4.40E-06 |
| GOTERM_BP_FAT | regulation of nervous system development | 22 | 3.6 | 2.00E-05 |
| GOTERM_BP_FAT | positive regulation of developmental process | 22 | 3.6 | 1.40E-03 |
| GOTERM_BP_FAT | embryonic morphogenesis | 22 | 3.6 | 2.20E-03 |
| GOTERM_BP_FAT | cell morphogenesis | 22 | 3.6 | 4.10E-03 |
| GOTERM_BP_FAT | cellular component morphogenesis | 22 | 3.6 | 1.20E-02 |
| INTERPRO | Protein kinase, core | 22 | 3.6 | 2.10E-02 |
| GOTERM_BP_FAT | negative regulation of transcription | 22 | 3.6 | 2.70E-02 |
| GOTERM_BP_FAT | pattern specification process | 21 | 3.4 | 1.80E-04 |
| GOTERM_BP_FAT | tube development | 21 | 3.4 | 2.00E-04 |
| KEGG_PATHWAY | Pathways in cancer | 21 | 3.4 | 3.40E-03 |
| GOTERM_CC_FAT | cell surface | 21 | 3.4 | 7.40E-03 |
| INTERPRO | Protein kinase, ATP binding site | 21 | 3.4 | 1.10E-02 |
| UP_SEQ_FEATURE | binding site:ATP | 21 | 3.4 | 2.30E-02 |
| GOTERM_BP_FAT | chordate embryonic development | 21 | 3.4 | 2.30E-02 |
| GOTERM_BP_FAT | embryonic development ending in birth or egg hatching | 21 | 3.4 | 2.60E-02 |
| GOTERM_MF_FAT | protein serine/threonine kinase activity | 21 | 3.4 | 3.20E-02 |
| GOTERM_BP_FAT | cell morphogenesis involved in differentiation | 20 | 3.3 | 4.00E-04 |
| GOTERM_BP_FAT | positive regulation of cell differentiation | 20 | 3.3 | 6.60E-04 |
| GOTERM_BP_FAT | regulation of transferase activity | 20 | 3.3 | 4.60E-03 |
| GOTERM_MF_FAT | transcription factor binding | 20 | 3.3 | 5.90E-03 |
| GOTERM_BP_FAT | negative regulation of RNA metabolic process | 20 | 3.3 | 1.20E-02 |
| SP_PIR_KEYWORDS | ubl conjugation | 20 | 3.3 | 3.30E-02 |
| GOTERM_BP_FAT | positive regulation of cell proliferation | 20 | 3.3 | 5.30E-02 |
| SP_PIR_KEYWORDS | golgi apparatus | 20 | 3.3 | 6.60E-02 |
| KEGG_PATHWAY | MAPK signaling pathway | 19 | 3.1 | 2.50E-03 |
| INTERPRO | Serine/threonine protein kinase, active site | 19 | 3.1 | 3.30E-03 |
| GOTERM_BP_FAT | regulation of protein kinase activity | 19 | 3.1 | 3.70E-03 |
| GOTERM_BP_FAT | regulation of kinase activity | 19 | 3.1 | 6.20E-03 |
| GOTERM_BP_FAT | regulation of growth | 19 | 3.1 | 9.60E-03 |
| GOTERM_BP_FAT | negative regulation of transcription, DNA-dependent | 19 | 3.1 | 2.00E-02 |
| GOTERM_BP_FAT | cell-cell signaling | 19 | 3.1 | 2.50E-02 |
| GOTERM_BP_FAT | protein catabolic process | 19 | 3.1 | 2.70E-02 |
| SP_PIR_KEYWORDS | cytoskeleton | 19 | 3.1 | 3.10E-02 |
| GOTERM_MF_FAT | passive transmembrane transporter activity | 19 | 3.1 | 4.60E-02 |
| GOTERM_MF_FAT | channel activity | 19 | 3.1 | 4.60E-02 |
| SP_PIR_KEYWORDS | cell junction | 19 | 3.1 | 5.30E-02 |
| GOTERM_CC_FAT | synapse | 19 | 3.1 | 6.90E-02 |
| GOTERM_CC_FAT | intrinsic to plasma membrane | 19 | 3.1 | 7.40E-02 |
| GOTERM_MF_FAT | cytoskeletal protein binding | 19 | 3.1 | 8.80E-02 |
| GOTERM_BP_FAT | neuron projection morphogenesis | 18 | 3 | 7.30E-04 |
| GOTERM_BP_FAT | cell projection morphogenesis | 18 | 3 | 2.20E-03 |
| GOTERM_BP_FAT | cell part morphogenesis | 18 | 3 | 3.40E-03 |
| SP_PIR_KEYWORDS | serine/threonine-protein kinase | 18 | 3 | 8.30E-03 |
| GOTERM_BP_FAT | negative regulation of molecular function | 18 | 3 | 1.50E-02 |
| INTERPRO | Serine/threonine protein kinase-related | 18 | 3 | 2.00E-02 |
| GOTERM_BP_FAT | proteolysis involved in cellular protein catabolic process | 18 | 3 | 2.70E-02 |
| GOTERM_BP_FAT | cellular protein catabolic process | 18 | 3 | 2.90E-02 |
| UP_SEQ_FEATURE | domain:Protein kinase | 18 | 3 | 3.30E-02 |
| GOTERM_BP_FAT | negative regulation of programmed cell death | 18 | 3 | 5.40E-02 |
| GOTERM_BP_FAT | negative regulation of cell death | 18 | 3 | 5.50E-02 |
| GOTERM_BP_FAT | regionalization | 17 | 2.8 | 5.00E-04 |
| GOTERM_BP_FAT | negative regulation of cell differentiation | 17 | 2.8 | 1.70E-03 |
| GOTERM_BP_FAT | tissue morphogenesis | 17 | 2.8 | 3.40E-03 |
| UP_SEQ_FEATURE | compositionally biased region:Pro-rich | 17 | 2.8 | 3.90E-02 |
| GOTERM_MF_FAT | protein homodimerization activity | 17 | 2.8 | 5.60E-02 |
| GOTERM_BP_FAT | negative regulation of apoptosis | 17 | 2.8 | 8.30E-02 |
| GOTERM_MF_FAT | substrate specific channel activity | 17 | 2.8 | 1.00E-01 |
| GOTERM_BP_FAT | regulation of neuron differentiation | 16 | 2.6 | 4.00E-04 |
| GOTERM_BP_FAT | axonogenesis | 16 | 2.6 | 1.20E-03 |
| GOTERM_BP_FAT | cell morphogenesis involved in neuron differentiation | 16 | 2.6 | 3.50E-03 |
| GOTERM_BP_FAT | regulation of cellular component size | 16 | 2.6 | 9.30E-03 |
| GOTERM_BP_FAT | epithelium development | 16 | 2.6 | 1.50E-02 |
| GOTERM_BP_FAT | regulation of hydrolase activity | 16 | 2.6 | 2.70E-02 |
| GOTERM_BP_FAT | skeletal system development | 16 | 2.6 | 2.80E-02 |
| GOTERM_BP_FAT | negative regulation of cell proliferation | 16 | 2.6 | 2.80E-02 |
| GOTERM_BP_FAT | regulation of protein modification process | 16 | 2.6 | 4.20E-02 |
| GOTERM_BP_FAT | enzyme linked receptor protein signaling pathway | 16 | 2.6 | 4.30E-02 |
| GOTERM_BP_FAT | modification-dependent macromolecule catabolic process | 16 | 2.6 | 4.80E-02 |
| GOTERM_BP_FAT | modification-dependent protein catabolic process | 16 | 2.6 | 4.80E-02 |
| GOTERM_BP_FAT | tube morphogenesis | 15 | 2.5 | 5.80E-04 |
| GOTERM_BP_FAT | regulation of cell motion | 15 | 2.5 | 3.30E-03 |
| GOTERM_BP_FAT | regulation of locomotion | 15 | 2.5 | 3.60E-03 |
| GOTERM_BP_FAT | positive regulation of protein kinase activity | 15 | 2.5 | 4.20E-03 |
| GOTERM_CC_FAT | axon | 15 | 2.5 | 5.00E-03 |
| INTERPRO | Serine/threonine protein kinase | 15 | 2.5 | 5.90E-03 |
| GOTERM_BP_FAT | positive regulation of kinase activity | 15 | 2.5 | 6.00E-03 |
| GOTERM_BP_FAT | positive regulation of transferase activity | 15 | 2.5 | 8.90E-03 |
| GOTERM_BP_FAT | embryonic organ development | 15 | 2.5 | 1.20E-02 |
| GOTERM_MF_FAT | protein heterodimerization activity | 15 | 2.5 | 1.60E-02 |
| SMART | S_TKc | 15 | 2.5 | 2.30E-02 |
| GOTERM_BP_FAT | regulation of cell cycle | 15 | 2.5 | 3.30E-02 |
| GOTERM_CC_FAT | cell soma | 15 | 2.5 | 3.70E-02 |
| GOTERM_CC_FAT | cell projection part | 15 | 2.5 | 3.80E-02 |
| GOTERM_BP_FAT | protein kinase cascade | 15 | 2.5 | 5.70E-02 |
| GOTERM_BP_FAT | monovalent inorganic cation transport | 15 | 2.5 | 9.80E-02 |
| GOTERM_BP_FAT | regulation of cell morphogenesis involved in differentiation | 14 | 2.3 | 1.30E-05 |
| GOTERM_CC_FAT | anchored to membrane | 14 | 2.3 | 9.60E-05 |
| GOTERM_BP_FAT | regulation of cell morphogenesis | 14 | 2.3 | 3.20E-04 |
| GOTERM_MF_FAT | protein kinase binding | 14 | 2.3 | 1.80E-03 |
| GOTERM_BP_FAT | regulation of cell migration | 14 | 2.3 | 3.10E-03 |
| GOTERM_MF_FAT | kinase binding | 14 | 2.3 | 5.90E-03 |
| GOTERM_CC_FAT | basolateral plasma membrane | 14 | 2.3 | 7.30E-03 |
| GOTERM_CC_FAT | transcription factor complex | 14 | 2.3 | 8.30E-03 |
| GOTERM_BP_FAT | gland development | 14 | 2.3 | 1.50E-02 |
| SP_PIR_KEYWORDS | Apoptosis | 14 | 2.3 | 2.00E-02 |
| GOTERM_BP_FAT | negative regulation of transcription from RNA polymerase II promoter | 14 | 2.3 | 5.10E-02 |
| GOTERM_CC_FAT | Golgi apparatus part | 14 | 2.3 | 6.80E-02 |
| GOTERM_BP_FAT | chromatin organization | 14 | 2.3 | 7.10E-02 |
| GOTERM_BP_FAT | positive regulation of cell development | 13 | 2.1 | 2.90E-05 |
| GOTERM_BP_FAT | regulation of cell projection organization | 13 | 2.1 | 3.30E-04 |
| GOTERM_BP_FAT | regulation of cell growth | 13 | 2.1 | 9.90E-03 |
| GOTERM_BP_FAT | regulation of organelle organization | 13 | 2.1 | 1.10E-02 |
| GOTERM_MF_FAT | alkali metal ion binding | 13 | 2.1 | 2.10E-02 |
| SP_PIR_KEYWORDS | ATP | 13 | 2.1 | 2.80E-02 |
| GOTERM_MF_FAT | phosphatase activity | 13 | 2.1 | 5.00E-02 |
| GOTERM_CC_FAT | proteinaceous extracellular matrix | 13 | 2.1 | 5.50E-02 |
| GOTERM_BP_FAT | small GTPase mediated signal transduction | 13 | 2.1 | 7.30E-02 |
| GOTERM_BP_FAT | negative regulation of catalytic activity | 13 | 2.1 | 8.30E-02 |
| GOTERM_BP_FAT | regulation of neuron projection development | 12 | 2 | 2.30E-04 |
| SP_PIR_KEYWORDS | DNA binding | 12 | 2 | 9.20E-03 |
| GOTERM_BP_FAT | urogenital system development | 12 | 2 | 1.30E-02 |
| GOTERM_BP_FAT | second-messenger-mediated signaling | 12 | 2 | 2.40E-02 |
| GOTERM_BP_FAT | positive regulation of cellular component organization | 12 | 2 | 4.60E-02 |
| KEGG_PATHWAY | Focal adhesion | 12 | 2 | 5.50E-02 |
| GOTERM_BP_FAT | microtubule-based process | 12 | 2 | 5.70E-02 |
| GOTERM_CC_FAT | actin cytoskeleton | 12 | 2 | 8.30E-02 |
| GOTERM_BP_FAT | protein oligomerization | 12 | 2 | 8.80E-02 |
| SP_PIR_KEYWORDS | ubl conjugation pathway | 12 | 2 | 9.20E-02 |
| GOTERM_BP_FAT | heart development | 12 | 2 | 9.30E-02 |
| GOTERM_BP_FAT | growth | 12 | 2 | 9.70E-02 |
| GOTERM_BP_FAT | positive regulation of neurogenesis | 11 | 1.8 | 2.50E-04 |
| KEGG_PATHWAY | Neurotrophin signaling pathway | 11 | 1.8 | 8.00E-03 |
| GOTERM_BP_FAT | negative regulation of cellular component organization | 11 | 1.8 | 8.50E-03 |
| GOTERM_BP_FAT | anterior/posterior pattern formation | 11 | 1.8 | 1.40E-02 |
| GOTERM_MF_FAT | structure-specific DNA binding | 11 | 1.8 | 1.90E-02 |
| KEGG_PATHWAY | Wnt signaling pathway | 11 | 1.8 | 2.00E-02 |
| GOTERM_CC_FAT | ion channel complex | 11 | 1.8 | 2.40E-02 |
| GOTERM_BP_FAT | regulation of hormone levels | 11 | 1.8 | 3.40E-02 |
| GOTERM_CC_FAT | internal side of plasma membrane | 11 | 1.8 | 3.80E-02 |
| GOTERM_MF_FAT | transcription cofactor activity | 11 | 1.8 | 4.20E-02 |
| INTERPRO | Nucleotide-binding, alpha-beta plait | 11 | 1.8 | 4.50E-02 |
| GOTERM_CC_FAT | cell-cell junction | 11 | 1.8 | 4.60E-02 |
| SP_PIR_KEYWORDS | repressor | 11 | 1.8 | 4.70E-02 |
| GOTERM_BP_FAT | regulation of cell size | 11 | 1.8 | 8.40E-02 |
| GOTERM_MF_FAT | enzyme activator activity | 11 | 1.8 | 8.40E-02 |
| GOTERM_BP_FAT | regulation of axonogenesis | 10 | 1.6 | 3.60E-04 |
| GOTERM_BP_FAT | protein heterooligomerization | 10 | 1.6 | 2.20E-03 |
| KEGG_PATHWAY | Melanogenesis | 10 | 1.6 | 3.00E-03 |
| GOTERM_BP_FAT | regulation of protein transport | 10 | 1.6 | 5.90E-03 |
| GOTERM_BP_FAT | axon guidance | 10 | 1.6 | 7.60E-03 |
| GOTERM_BP_FAT | regulation of establishment of protein localization | 10 | 1.6 | 8.10E-03 |
| GOTERM_BP_FAT | regulation of cytoskeleton organization | 10 | 1.6 | 9.60E-03 |
| KEGG_PATHWAY | Oocyte meiosis | 10 | 1.6 | 1.00E-02 |
| GOTERM_BP_FAT | lung development | 10 | 1.6 | 1.00E-02 |
| GOTERM_MF_FAT | symporter activity | 10 | 1.6 | 1.10E-02 |
| GOTERM_BP_FAT | respiratory tube development | 10 | 1.6 | 1.10E-02 |
| GOTERM_BP_FAT | skeletal system morphogenesis | 10 | 1.6 | 1.30E-02 |
| GOTERM_BP_FAT | respiratory system development | 10 | 1.6 | 1.50E-02 |
| GOTERM_BP_FAT | regulation of protein localization | 10 | 1.6 | 2.60E-02 |
| GOTERM_BP_FAT | anion transport | 10 | 1.6 | 2.90E-02 |
| GOTERM_CC_FAT | apical plasma membrane | 10 | 1.6 | 3.00E-02 |
| GOTERM_BP_FAT | negative regulation of transport | 10 | 1.6 | 4.10E-02 |
| INTERPRO | Homeodomain-related | 10 | 1.6 | 4.60E-02 |
| GOTERM_BP_FAT | anti-apoptosis | 10 | 1.6 | 5.00E-02 |
| GOTERM_MF_FAT | chromatin binding | 10 | 1.6 | 5.60E-02 |
| GOTERM_BP_FAT | morphogenesis of an epithelium | 10 | 1.6 | 6.90E-02 |
| SP_PIR_KEYWORDS | Homeobox | 10 | 1.6 | 7.30E-02 |
| UP_SEQ_FEATURE | propeptide:Removed in mature form | 10 | 1.6 | 7.90E-02 |
| GOTERM_CC_FAT | microtubule | 10 | 1.6 | 8.60E-02 |
| GOTERM_BP_FAT | regulation of synaptic transmission | 10 | 1.6 | 9.40E-02 |
| GOTERM_BP_FAT | chromatin modification | 10 | 1.6 | 9.60E-02 |
| SP_PIR_KEYWORDS | gpi-anchor | 9 | 1.5 | 4.60E-03 |
| GOTERM_BP_FAT | branching morphogenesis of a tube | 9 | 1.5 | 6.20E-03 |
| GOTERM_BP_FAT | limb morphogenesis | 9 | 1.5 | 1.30E-02 |
| GOTERM_BP_FAT | appendage morphogenesis | 9 | 1.5 | 1.30E-02 |
| GOTERM_BP_FAT | limb development | 9 | 1.5 | 1.60E-02 |
| GOTERM_BP_FAT | appendage development | 9 | 1.5 | 1.60E-02 |
| GOTERM_BP_FAT | positive regulation of cell motion | 9 | 1.5 | 1.70E-02 |
| UP_SEQ_FEATURE | compositionally biased region:Poly-Gly | 9 | 1.5 | 1.80E-02 |
| GOTERM_BP_FAT | negative regulation of growth | 9 | 1.5 | 1.90E-02 |
| GOTERM_MF_FAT | RNA polymerase II transcription factor activity | 9 | 1.5 | 2.40E-02 |
| SP_PIR_KEYWORDS | Symport | 9 | 1.5 | 2.60E-02 |
| UP_SEQ_FEATURE | DNA-binding region:Basic motif | 9 | 1.5 | 2.80E-02 |
| INTERPRO | PDZ/DHR/GLGF | 9 | 1.5 | 3.20E-02 |
| GOTERM_BP_FAT | regulation of cellular component biogenesis | 9 | 1.5 | 3.50E-02 |
| GOTERM_BP_FAT | morphogenesis of a branching structure | 9 | 1.5 | 3.50E-02 |
| GOTERM_BP_FAT | regulation of MAP kinase activity | 9 | 1.5 | 3.50E-02 |
| GOTERM_BP_FAT | kidney development | 9 | 1.5 | 4.00E-02 |
| GOTERM_BP_FAT | regulation of gene-specific transcription | 9 | 1.5 | 5.00E-02 |
| KEGG_PATHWAY | Cell cycle | 9 | 1.5 | 5.40E-02 |
| SP_PIR_KEYWORDS | phosphotransferase | 9 | 1.5 | 6.60E-02 |
| SMART | PDZ | 9 | 1.5 | 7.30E-02 |
| GOTERM_MF_FAT | growth factor activity | 9 | 1.5 | 7.30E-02 |
| GOTERM_BP_FAT | positive regulation of hydrolase activity | 9 | 1.5 | 8.40E-02 |
| GOTERM_BP_FAT | cell fate commitment | 9 | 1.5 | 9.00E-02 |
| GOTERM_BP_FAT | potassium ion transport | 9 | 1.5 | 9.60E-02 |
| GOTERM_BP_FAT | mesenchymal cell development | 8 | 1.3 | 7.90E-04 |
| GOTERM_BP_FAT | mesenchymal cell differentiation | 8 | 1.3 | 9.00E-04 |
| GOTERM_BP_FAT | mesenchyme development | 8 | 1.3 | 1.00E-03 |
| KEGG_PATHWAY | Hedgehog signaling pathway | 8 | 1.3 | 1.40E-03 |
| KEGG_PATHWAY | Long-term potentiation | 8 | 1.3 | 5.90E-03 |
| GOTERM_BP_FAT | cartilage development | 8 | 1.3 | 9.90E-03 |
| GOTERM_BP_FAT | gliogenesis | 8 | 1.3 | 1.10E-02 |
| UP_SEQ_FEATURE | domain:Leucine-zipper | 8 | 1.3 | 1.20E-02 |
| GOTERM_BP_FAT | embryonic appendage morphogenesis | 8 | 1.3 | 1.90E-02 |
| GOTERM_BP_FAT | embryonic limb morphogenesis | 8 | 1.3 | 1.90E-02 |
| KEGG_PATHWAY | TGF-beta signaling pathway | 8 | 1.3 | 2.20E-02 |
| GOTERM_MF_FAT | transcription coactivator activity | 8 | 1.3 | 2.60E-02 |
| GOTERM_BP_FAT | positive regulation of cell migration | 8 | 1.3 | 3.10E-02 |
| GOTERM_MF_FAT | manganese ion binding | 8 | 1.3 | 3.70E-02 |
| GOTERM_BP_FAT | activation of protein kinase activity | 8 | 1.3 | 4.40E-02 |
| GOTERM_BP_FAT | positive regulation of locomotion | 8 | 1.3 | 5.00E-02 |
| GOTERM_BP_FAT | protein amino acid dephosphorylation | 8 | 1.3 | 5.30E-02 |
| GOTERM_BP_FAT | epidermis development | 8 | 1.3 | 5.30E-02 |
| GOTERM_BP_FAT | sodium ion transport | 8 | 1.3 | 6.70E-02 |
| GOTERM_BP_FAT | epithelial cell differentiation | 8 | 1.3 | 6.70E-02 |
| GOTERM_BP_FAT | ectoderm development | 8 | 1.3 | 7.60E-02 |
| GOTERM_MF_FAT | anion transmembrane transporter activity | 8 | 1.3 | 9.90E-02 |
| GOTERM_BP_FAT | regulation of cell adhesion | 8 | 1.3 | 1.00E-01 |
| GOTERM_BP_FAT | gut development | 7 | 1.1 | 3.70E-03 |
| KEGG_PATHWAY | Inositol phosphate metabolism | 7 | 1.1 | 6.60E-03 |
| GOTERM_BP_FAT | negative regulation of neurogenesis | 7 | 1.1 | 9.00E-03 |
| GOTERM_BP_FAT | negative regulation of cell development | 7 | 1.1 | 1.10E-02 |
| GOTERM_BP_FAT | glial cell differentiation | 7 | 1.1 | 1.30E-02 |
| GOTERM_BP_FAT | regulation of actin cytoskeleton organization | 7 | 1.1 | 1.80E-02 |
| GOTERM_BP_FAT | regulation of actin filament-based process | 7 | 1.1 | 2.20E-02 |
| GOTERM_MF_FAT | small GTPase binding | 7 | 1.1 | 3.50E-02 |
| KEGG_PATHWAY | Chronic myeloid leukemia | 7 | 1.1 | 3.50E-02 |
| GOTERM_BP_FAT | positive regulation of gene-specific transcription | 7 | 1.1 | 3.60E-02 |
| GOTERM_BP_FAT | endocrine system development | 7 | 1.1 | 3.60E-02 |
| GOTERM_MF_FAT | kinase regulator activity | 7 | 1.1 | 4.10E-02 |
| GOTERM_BP_FAT | negative regulation of cell growth | 7 | 1.1 | 4.50E-02 |
| GOTERM_MF_FAT | solute:cation symporter activity | 7 | 1.1 | 4.60E-02 |
| KEGG_PATHWAY | Gap junction | 7 | 1.1 | 4.90E-02 |
| GOTERM_BP_FAT | regulation of synaptic plasticity | 7 | 1.1 | 5.00E-02 |
| GOTERM_BP_FAT | inorganic anion transport | 7 | 1.1 | 5.00E-02 |
| GOTERM_MF_FAT | GTPase binding | 7 | 1.1 | 5.60E-02 |
| GOTERM_BP_FAT | placenta development | 7 | 1.1 | 6.60E-02 |
| GOTERM_BP_FAT | negative regulation of cell size | 7 | 1.1 | 6.60E-02 |
| GOTERM_BP_FAT | regulation of specific transcription from RNA polymerase II promoter | 7 | 1.1 | 7.90E-02 |
| GOTERM_BP_FAT | microtubule-based movement | 7 | 1.1 | 8.20E-02 |
| GOTERM_MF_FAT | neurotransmitter binding | 7 | 1.1 | 9.20E-02 |
| GOTERM_MF_FAT | double-stranded DNA binding | 7 | 1.1 | 1.00E-01 |
| GOTERM_BP_FAT | negative regulation of cell projection organization | 6 | 1 | 6.80E-03 |
| GOTERM_BP_FAT | regulation of muscle development | 6 | 1 | 1.50E-02 |
| KEGG_PATHWAY | Notch signaling pathway | 6 | 1 | 2.10E-02 |
| INTERPRO | Basic-leucine zipper (bZIP) transcription factor | 6 | 1 | 2.70E-02 |
| GOTERM_BP_FAT | chloride transport | 6 | 1 | 2.70E-02 |
| GOTERM_CC_FAT | chromatin remodeling complex | 6 | 1 | 2.80E-02 |
| GOTERM_BP_FAT | phosphoinositide-mediated signaling | 6 | 1 | 4.90E-02 |
| SMART | BRLZ | 6 | 1 | 5.00E-02 |
| GOTERM_BP_FAT | positive regulation of cell projection organization | 6 | 1 | 5.20E-02 |
| GOTERM_MF_FAT | protein kinase regulator activity | 6 | 1 | 6.00E-02 |
| GOTERM_CC_FAT | growth cone | 6 | 1 | 6.60E-02 |
| GOTERM_CC_FAT | site of polarized growth | 6 | 1 | 6.60E-02 |
| UP_SEQ_FEATURE | domain:PDZ | 6 | 1 | 6.80E-02 |
| KEGG_PATHWAY | Pancreatic cancer | 6 | 1 | 7.40E-02 |
| KEGG_PATHWAY | Renal cell carcinoma | 6 | 1 | 7.40E-02 |
| GOTERM_BP_FAT | hindbrain development | 6 | 1 | 7.60E-02 |
| KEGG_PATHWAY | Phosphatidylinositol signaling system | 6 | 1 | 7.80E-02 |
| SP_PIR_KEYWORDS | myristate | 6 | 1 | 7.90E-02 |
| GOTERM_BP_FAT | heart morphogenesis | 6 | 1 | 8.00E-02 |
| GOTERM_BP_FAT | regulation of protein complex assembly | 6 | 1 | 8.30E-02 |
| UP_SEQ_FEATURE | compositionally biased region:Poly-Gln | 6 | 1 | 8.40E-02 |
| GOTERM_CC_FAT | focal adhesion | 6 | 1 | 8.80E-02 |
| GOTERM_MF_FAT | SH3 domain binding | 6 | 1 | 8.90E-02 |
| KEGG_PATHWAY | Adherens junction | 6 | 1 | 9.40E-02 |
| GOTERM_BP_FAT | neural tube development | 6 | 1 | 1.00E-01 |
| GOTERM_BP_FAT | epithelial to mesenchymal transition | 5 | 0.8 | 1.20E-03 |
| GOTERM_MF_FAT | transforming growth factor beta receptor binding | 5 | 0.8 | 1.50E-03 |
| GOTERM_MF_FAT | specific RNA polymerase II transcription factor activity | 5 | 0.8 | 3.50E-03 |
| GOTERM_CC_FAT | anchored to plasma membrane | 5 | 0.8 | 5.00E-03 |
| GOTERM_BP_FAT | negative regulation of axonogenesis | 5 | 0.8 | 1.60E-02 |
| GOTERM_BP_FAT | digestive system development | 5 | 0.8 | 1.80E-02 |
| GOTERM_BP_FAT | positive regulation of axonogenesis | 5 | 0.8 | 2.00E-02 |
| GOTERM_BP_FAT | pituitary gland development | 5 | 0.8 | 2.20E-02 |
| KEGG_PATHWAY | Prion diseases | 5 | 0.8 | 2.60E-02 |
| GOTERM_BP_FAT | determination of bilateral symmetry | 5 | 0.8 | 2.90E-02 |
| GOTERM_BP_FAT | determination of symmetry | 5 | 0.8 | 2.90E-02 |
| GOTERM_BP_FAT | regulation of cell division | 5 | 0.8 | 3.50E-02 |
| GOTERM_CC_FAT | microvillus | 5 | 0.8 | 4.00E-02 |
| GOTERM_BP_FAT | negative regulation of specific transcription from RNA polymerase II promoter | 5 | 0.8 | 4.10E-02 |
| GOTERM_BP_FAT | cell junction organization | 5 | 0.8 | 4.50E-02 |
| GOTERM_MF_FAT | SMAD binding | 5 | 0.8 | 5.00E-02 |
| GOTERM_BP_FAT | osteoblast differentiation | 5 | 0.8 | 5.20E-02 |
| GOTERM_BP_FAT | regulation of striated muscle tissue development | 5 | 0.8 | 5.20E-02 |
| GOTERM_BP_FAT | diencephalon development | 5 | 0.8 | 5.20E-02 |
| GOTERM_BP_FAT | activation of adenylate cyclase activity by G-protein signaling pathway | 5 | 0.8 | 5.50E-02 |
| GOTERM_BP_FAT | regulation of adenylate cyclase activity involved in G-protein signaling | 5 | 0.8 | 5.50E-02 |
| GOTERM_BP_FAT | positive regulation of adenylate cyclase activity by G-protein signaling pathway | 5 | 0.8 | 5.50E-02 |
| GOTERM_BP_FAT | glutamine family amino acid metabolic process | 5 | 0.8 | 5.90E-02 |
| GOTERM_BP_FAT | neuron fate commitment | 5 | 0.8 | 6.80E-02 |
| GOTERM_BP_FAT | negative regulation of gene-specific transcription | 5 | 0.8 | 6.80E-02 |
| GOTERM_CC_FAT | trans-Golgi network | 5 | 0.8 | 7.00E-02 |
| GOTERM_BP_FAT | carbohydrate homeostasis | 5 | 0.8 | 7.20E-02 |
| GOTERM_BP_FAT | glucose homeostasis | 5 | 0.8 | 7.20E-02 |
| GOTERM_BP_FAT | regulation of neuronal synaptic plasticity | 5 | 0.8 | 7.60E-02 |
| GOTERM_BP_FAT | regulation of protein catabolic process | 5 | 0.8 | 7.60E-02 |
| GOTERM_BP_FAT | negative regulation of hydrolase activity | 5 | 0.8 | 7.60E-02 |
| KEGG_PATHWAY | Basal cell carcinoma | 5 | 0.8 | 8.50E-02 |
| GOTERM_BP_FAT | activation of adenylate cyclase activity | 5 | 0.8 | 8.60E-02 |
| GOTERM_BP_FAT | positive regulation of specific transcription from RNA polymerase II promoter | 5 | 0.8 | 8.60E-02 |
| GOTERM_BP_FAT | regulation of developmental growth | 5 | 0.8 | 8.60E-02 |
| UP_SEQ_FEATURE | compositionally biased region:Ala-rich | 5 | 0.8 | 8.90E-02 |
| GOTERM_BP_FAT | regulation of protein polymerization | 5 | 0.8 | 9.10E-02 |
| GOTERM_BP_FAT | mesoderm development | 5 | 0.8 | 9.10E-02 |
| GOTERM_BP_FAT | monocarboxylic acid transport | 5 | 0.8 | 9.60E-02 |
| GOTERM_BP_FAT | hormone transport | 5 | 0.8 | 9.60E-02 |
| GOTERM_BP_FAT | positive regulation of protein transport | 5 | 0.8 | 9.60E-02 |
| GOTERM_BP_FAT | positive regulation of adenylate cyclase activity | 5 | 0.8 | 9.60E-02 |
| GOTERM_BP_FAT | regulation of proteolysis | 5 | 0.8 | 9.60E-02 |
| GOTERM_BP_FAT | regulation of mRNA processing | 4 | 0.7 | 9.80E-03 |
| GOTERM_BP_FAT | cartilage condensation | 4 | 0.7 | 1.70E-02 |
| GOTERM_BP_FAT | regulation of smoothened signaling pathway | 4 | 0.7 | 2.30E-02 |
| GOTERM_BP_FAT | positive regulation of developmental growth | 4 | 0.7 | 2.60E-02 |
| GOTERM_MF_FAT | kinase activator activity | 4 | 0.7 | 2.90E-02 |
| GOTERM_BP_FAT | autonomic nervous system development | 4 | 0.7 | 3.00E-02 |
| GOTERM_BP_FAT | microtubule-based transport | 4 | 0.7 | 3.00E-02 |
| GOTERM_BP_FAT | patterning of blood vessels | 4 | 0.7 | 3.40E-02 |
| GOTERM_BP_FAT | gut morphogenesis | 4 | 0.7 | 3.80E-02 |
| GOTERM_BP_FAT | cell-cell junction organization | 4 | 0.7 | 3.80E-02 |
| INTERPRO | Six-bladed beta-propeller, TolB-like | 4 | 0.7 | 4.20E-02 |
| GOTERM_BP_FAT | regulation of BMP signaling pathway | 4 | 0.7 | 4.30E-02 |
| SP_PIR_KEYWORDS | dynein | 4 | 0.7 | 4.50E-02 |
| GOTERM_MF_FAT | anion:cation symporter activity | 4 | 0.7 | 4.60E-02 |
| GOTERM_BP_FAT | oligodendrocyte differentiation | 4 | 0.7 | 4.70E-02 |
| GOTERM_BP_FAT | regulation of axon extension | 4 | 0.7 | 4.70E-02 |
| GOTERM_BP_FAT | regulation of dephosphorylation | 4 | 0.7 | 4.70E-02 |
| GOTERM_BP_FAT | positive regulation of protein catabolic process | 4 | 0.7 | 5.20E-02 |
| GOTERM_CC_FAT | stress fiber | 4 | 0.7 | 6.10E-02 |
| INTERPRO | Thrombospondin, type 1 repeat | 4 | 0.7 | 6.20E-02 |
| GOTERM_BP_FAT | digestive tract morphogenesis | 4 | 0.7 | 6.80E-02 |
| GOTERM_BP_FAT | peptidyl-serine phosphorylation | 4 | 0.7 | 6.80E-02 |
| GOTERM_CC_FAT | actin filament bundle | 4 | 0.7 | 7.20E-02 |
| INTERPRO | WW/Rsp5/WWP | 4 | 0.7 | 7.60E-02 |
| UP_SEQ_FEATURE | region of interest:Ligand-binding | 4 | 0.7 | 7.80E-02 |
| GOTERM_CC_FAT | actomyosin | 4 | 0.7 | 8.40E-02 |
| GOTERM_CC_FAT | dynein complex | 4 | 0.7 | 8.40E-02 |
| SMART | TSP1 | 4 | 0.7 | 9.30E-02 |
| GOTERM_BP_FAT | cytoskeleton-dependent intracellular transport | 4 | 0.7 | 1.00E-01 |
| GOTERM_BP_FAT | response to endoplasmic reticulum stress | 4 | 0.7 | 1.00E-01 |
| GOTERM_BP_FAT | regulation of pH | 4 | 0.7 | 1.00E-01 |
| GOTERM_BP_FAT | compartment specification | 3 | 0.5 | 6.20E-03 |
| GOTERM_BP_FAT | pathway-restricted SMAD protein phosphorylation | 3 | 0.5 | 1.00E-02 |
| GOTERM_MF_FAT | ammonia transporter activity | 3 | 0.5 | 1.50E-02 |
| GOTERM_BP_FAT | ammonium transport | 3 | 0.5 | 1.50E-02 |
| GOTERM_BP_FAT | somatic stem cell division | 3 | 0.5 | 2.00E-02 |
| INTERPRO | Yip1 domain | 3 | 0.5 | 2.30E-02 |
| GOTERM_BP_FAT | rhombomere development | 3 | 0.5 | 2.60E-02 |
| GOTERM_BP_FAT | regulation of myoblast differentiation | 3 | 0.5 | 2.60E-02 |
| GOTERM_BP_FAT | foregut morphogenesis | 3 | 0.5 | 2.60E-02 |
| GOTERM_BP_FAT | regulation of receptor biosynthetic process | 3 | 0.5 | 2.60E-02 |
| GOTERM_MF_FAT | protein phosphatase type 2A regulator activity | 3 | 0.5 | 3.30E-02 |
| INTERPRO | B-box, C-terminal | 3 | 0.5 | 4.10E-02 |
| GOTERM_BP_FAT | osteoblast development | 3 | 0.5 | 4.90E-02 |
| GOTERM_BP_FAT | stem cell division | 3 | 0.5 | 4.90E-02 |
| INTERPRO | Zinc finger, MYND-type | 3 | 0.5 | 5.10E-02 |
| SMART | BBC | 3 | 0.5 | 5.60E-02 |
| UP_SEQ_FEATURE | region of interest:Linker 2 | 3 | 0.5 | 6.10E-02 |
| GOTERM_BP_FAT | positive regulation of axon extension | 3 | 0.5 | 6.60E-02 |
| GOTERM_BP_FAT | thyroid gland development | 3 | 0.5 | 7.50E-02 |
| GOTERM_BP_FAT | regulation of skeletal muscle fiber development | 3 | 0.5 | 7.50E-02 |
| GOTERM_BP_FAT | glutamine family amino acid catabolic process | 3 | 0.5 | 7.50E-02 |
| PIR_SUPERFAMILY | PIRSF002350:calmodulin | 3 | 0.5 | 8.10E-02 |
| GOTERM_BP_FAT | ectodermal gut morphogenesis | 3 | 0.5 | 8.50E-02 |
| GOTERM_BP_FAT | ectodermal gut development | 3 | 0.5 | 8.50E-02 |
| GOTERM_BP_FAT | male genitalia development | 3 | 0.5 | 8.50E-02 |
| GOTERM_BP_FAT | hair follicle morphogenesis | 3 | 0.5 | 8.50E-02 |
| INTERPRO | Peptidase M14, carboxypeptidase A | 3 | 0.5 | 8.70E-02 |
| INTERPRO | Zinc finger, ZZ-type | 3 | 0.5 | 8.70E-02 |
| INTERPRO | Major intrinsic protein | 3 | 0.5 | 8.70E-02 |
| UP_SEQ_FEATURE | short sequence motif:NPA 2 | 3 | 0.5 | 8.80E-02 |
| UP_SEQ_FEATURE | short sequence motif:NPA 1 | 3 | 0.5 | 8.80E-02 |
| GOTERM_MF_FAT | ligand-dependent nuclear receptor transcription coactivator activity | 3 | 0.5 | 9.40E-02 |
| GOTERM_MF_FAT | protein kinase activator activity | 3 | 0.5 | 9.40E-02 |
| GOTERM_BP_FAT | placenta blood vessel development | 3 | 0.5 | 9.50E-02 |
| GOTERM_BP_FAT | hyperosmotic response | 3 | 0.5 | 9.50E-02 |
| GOTERM_BP_FAT | neuron recognition | 3 | 0.5 | 9.50E-02 |
| GOTERM_BP_FAT | inositol metabolic process | 3 | 0.5 | 9.50E-02 |
| INTERPRO | Speract/scavenger receptor | 3 | 0.5 | 1.00E-01 |
| GOTERM_MF_FAT | Rab GDP-dissociation inhibitor activity | 2 | 0.3 | 6.40E-02 |
| INTERPRO | Rab GDI protein | 2 | 0.3 | 8.20E-02 |
| INTERPRO | MAP Kinase Interacting Kinase | 2 | 0.3 | 8.20E-02 |
| PIR_SUPERFAMILY | PIRSF019647:GDP dissociation inhibitor XAP-4 | 2 | 0.3 | 8.60E-02 |
| GOTERM_CC_FAT | platelet dense granule membrane | 2 | 0.3 | 9.40E-02 |
| GOTERM_MF_FAT | galanin receptor activity | 2 | 0.3 | 9.40E-02 |
| GOTERM_MF_FAT | titin binding | 2 | 0.3 | 9.40E-02 |
| GOTERM_BP_FAT | lung cell differentiation | 2 | 0.3 | 9.50E-02 |
| GOTERM_BP_FAT | activation of transmembrane receptor protein tyrosine kinase activity | 2 | 0.3 | 9.50E-02 |
| GOTERM_BP_FAT | regulation of alkaline phosphatase activity | 2 | 0.3 | 9.50E-02 |
| GOTERM_BP_FAT | lung epithelial cell differentiation | 2 | 0.3 | 9.50E-02 |

**Dataset S6. Enriched GO term clusters upon lead exposure**

| **Cluster 1** | **Enrichment Score: 5.12** | **Count** | **P-value** |
| --- | --- | --- | --- |
| GOTERM_MF_FAT | transcription activator activity | 33 | 6.50E-10 |
| GOTERM_BP_FAT | transcription | 53 | 1.10E-08 |
| SP_PIR_KEYWORDS | transcription regulation | 55 | 1.20E-08 |
| SP_PIR_KEYWORDS | Transcription | 55 | 1.50E-07 |
| GOTERM_BP_FAT | positive regulation of macromolecule metabolic process | 55 | 7.70E-07 |
| GOTERM_BP_FAT | positive regulation of RNA metabolic process | 37 | 1.20E-06 |
| GOTERM_MF_FAT | transcription regulator activity | 64 | 1.20E-06 |
| GOTERM_BP_FAT | positive regulation of nucleobase, nucleoside, nucleotide and nucleic acid metabolic process | 44 | 1.60E-06 |
| GOTERM_BP_FAT | positive regulation of nitrogen compound metabolic process | 44 | 3.40E-06 |
| GOTERM_BP_FAT | regulation of transcription | 86 | 3.90E-06 |
| GOTERM_BP_FAT | positive regulation of gene expression | 40 | 4.10E-06 |
| SP_PIR_KEYWORDS | activator | 23 | 5.30E-06 |
| GOTERM_BP_FAT | positive regulation of transcription | 39 | 5.80E-06 |
| GOTERM_BP_FAT | positive regulation of transcription, DNA-dependent | 35 | 6.80E-06 |
| GOTERM_BP_FAT | positive regulation of macromolecule biosynthetic process | 43 | 8.60E-06 |
| GOTERM_MF_FAT | transcription factor activity | 43 | 1.20E-05 |
| GOTERM_BP_FAT | positive regulation of biosynthetic process | 44 | 2.20E-05 |
| GOTERM_BP_FAT | positive regulation of cellular biosynthetic process | 43 | 3.10E-05 |
| GOTERM_MF_FAT | DNA binding | 70 | 7.80E-05 |
| SP_PIR_KEYWORDS | dna-binding | 44 | 1.20E-04 |
| GOTERM_BP_FAT | regulation of transcription from RNA polymerase II promoter | 38 | 1.90E-04 |
| GOTERM_BP_FAT | positive regulation of transcription from RNA polymerase II promoter | 27 | 3.90E-04 |
| GOTERM_MF_FAT | sequence-specific DNA binding | 32 | 4.60E-04 |
| GOTERM_BP_FAT | regulation of RNA metabolic process | 62 | 1.10E-03 |
| GOTERM_BP_FAT | regulation of transcription, DNA-dependent | 59 | 2.60E-03 |
| SP_PIR_KEYWORDS | nucleus | 87 | 3.60E-03 |
|  |  |  |  |
| **Cluster 2** | **Enrichment Score:3.29** | **Count** | **P-value** |
| GOTERM_BP_FAT | regulation of cell development | 25 | 7.50E-07 |
| GOTERM_BP_FAT | regulation of neurogenesis | 22 | 4.40E-06 |
| GOTERM_BP_FAT | regulation of cell morphogenesis involved in differentiation | 14 | 1.30E-05 |
| GOTERM_BP_FAT | regulation of nervous system development | 22 | 2.00E-05 |
| GOTERM_BP_FAT | positive regulation of cell development | 13 | 2.90E-05 |
| GOTERM_BP_FAT | regulation of neuron projection development | 12 | 2.30E-04 |
| GOTERM_BP_FAT | positive regulation of neurogenesis | 11 | 2.50E-04 |
| GOTERM_BP_FAT | regulation of cell morphogenesis | 14 | 3.20E-04 |
| GOTERM_BP_FAT | regulation of cell projection organization | 13 | 3.30E-04 |
| GOTERM_BP_FAT | regulation of axonogenesis | 10 | 3.60E-04 |
| GOTERM_BP_FAT | regulation of neuron differentiation | 16 | 4.00E-04 |
| GOTERM_BP_FAT | positive regulation of cell differentiation | 20 | 6.60E-04 |
| GOTERM_BP_FAT | positive regulation of developmental process | 22 | 1.40E-03 |
| GOTERM_BP_FAT | negative regulation of cell projection organization | 6 | 6.80E-03 |
| GOTERM_BP_FAT | negative regulation of cellular component organization | 11 | 8.50E-03 |
| GOTERM_BP_FAT | negative regulation of neurogenesis | 7 | 9.00E-03 |
| GOTERM_BP_FAT | negative regulation of cell development | 7 | 1.10E-02 |
| GOTERM_BP_FAT | negative regulation of axonogenesis | 5 | 1.60E-02 |
| GOTERM_BP_FAT | positive regulation of axonogenesis | 5 | 2.00E-02 |
| GOTERM_BP_FAT | positive regulation of cellular component organization | 12 | 4.60E-02 |
|  |  |  |  |
| **Cluster 3** | **Enrichment Score:2.93** | **Count** | **P-value** |
| GOTERM_BP_FAT | neuron differentiation | 37 | 1.00E-06 |
| GOTERM_BP_FAT | neuron projection development | 23 | 9.60E-05 |
| GOTERM_BP_FAT | cell projection organization | 26 | 3.60E-04 |
| GOTERM_BP_FAT | cell morphogenesis involved in differentiation | 20 | 4.00E-04 |
| GOTERM_BP_FAT | neuron development | 25 | 4.80E-04 |
| GOTERM_BP_FAT | neuron projection morphogenesis | 18 | 7.30E-04 |
| GOTERM_BP_FAT | axonogenesis | 16 | 1.20E-03 |
| GOTERM_BP_FAT | cell projection morphogenesis | 18 | 2.20E-03 |
| GOTERM_BP_FAT | cell part morphogenesis | 18 | 3.40E-03 |
| GOTERM_BP_FAT | cell morphogenesis involved in neuron differentiation | 16 | 3.50E-03 |
| GOTERM_BP_FAT | cell morphogenesis | 22 | 4.10E-03 |
| GOTERM_BP_FAT | axon guidance | 10 | 7.60E-03 |
| GOTERM_BP_FAT | cellular component morphogenesis | 22 | 1.20E-02 |
| GOTERM_BP_FAT | cell motion | 19 | 1.40E-01 |
|  |  |  |  |
| **Cluster 4** | **Enrichment Score:2.89** | **Count** | **P-value** |
| GOTERM_BP_FAT | pattern specification process | 21 | 1.80E-04 |
| GOTERM_BP_FAT | regionalization | 17 | 5.00E-04 |
| GOTERM_BP_FAT | embryonic morphogenesis | 22 | 2.20E-03 |
| GOTERM_BP_FAT | anterior/posterior pattern formation | 11 | 1.40E-02 |
|  |  |  |  |
| **Cluster 5** | **Enrichment Score:2.42** | **Count** | **P-value** |
| GOTERM_BP_FAT | positive regulation of molecular function | 32 | 6.40E-04 |
| GOTERM_BP_FAT | regulation of phosphate metabolic process | 27 | 1.20E-03 |
| GOTERM_BP_FAT | regulation of phosphorus metabolic process | 27 | 1.20E-03 |
| GOTERM_BP_FAT | positive regulation of catalytic activity | 28 | 1.50E-03 |
| GOTERM_BP_FAT | regulation of protein kinase activity | 19 | 3.70E-03 |
| GOTERM_BP_FAT | positive regulation of protein kinase activity | 15 | 4.20E-03 |
| GOTERM_BP_FAT | regulation of transferase activity | 20 | 4.60E-03 |
| GOTERM_BP_FAT | positive regulation of kinase activity | 15 | 6.00E-03 |
| GOTERM_BP_FAT | regulation of kinase activity | 19 | 6.20E-03 |
| GOTERM_BP_FAT | regulation of phosphorylation | 24 | 6.50E-03 |
| GOTERM_BP_FAT | positive regulation of transferase activity | 15 | 8.90E-03 |
| GOTERM_BP_FAT | activation of protein kinase activity | 8 | 4.40E-02 |
|  |  |  |  |
| **Cluster 6** | **Enrichment Score:2.41** | **Count** | **P-value** |
| GOTERM_MF_FAT | protein kinase binding | 14 | 1.80E-03 |
| GOTERM_MF_FAT | enzyme binding | 28 | 5.50E-03 |
| GOTERM_MF_FAT | kinase binding | 14 | 5.90E-03 |
|  |  |  |  |
| **Cluster 7** | **Enrichment Score:2.28** | **Count** | **P-value** |
| GOTERM_CC_FAT | nucleoplasm | 39 | 1.10E-03 |
| GOTERM_CC_FAT | nucleoplasm part | 27 | 1.70E-03 |
| GOTERM_CC_FAT | nuclear lumen | 46 | 4.00E-03 |
| GOTERM_CC_FAT | transcription factor complex | 14 | 8.30E-03 |
| GOTERM_CC_FAT | organelle lumen | 55 | 1.10E-02 |
| GOTERM_CC_FAT | membrane-enclosed lumen | 56 | 1.20E-02 |
| GOTERM_CC_FAT | intracellular organelle lumen | 53 | 1.30E-02 |
|  |  |  |  |
| **Cluster 8** | **Enrichment Score:2.19** | **Count** | **P-value** |
| GOTERM_BP_FAT | mesenchymal cell development | 8 | 7.90E-04 |
| GOTERM_BP_FAT | mesenchymal cell differentiation | 8 | 9.00E-04 |
| GOTERM_BP_FAT | mesenchyme development | 8 | 1.00E-03 |
| GOTERM_BP_FAT | epithelial to mesenchymal transition | 5 | 1.20E-03 |
| GOTERM_BP_FAT | neural crest cell development | 3 | 2.80E-01 |
| GOTERM_BP_FAT | neural crest cell differentiation | 3 | 2.80E-01 |
|  |  |  |  |
| **Cluster 9** | **Enrichment Score:2.17** | **Count** | **P-value** |
| GOTERM_BP_FAT | tube development | 21 | 2.00E-04 |
| GOTERM_BP_FAT | tube morphogenesis | 15 | 5.80E-04 |
| GOTERM_BP_FAT | tissue morphogenesis | 17 | 3.40E-03 |
| GOTERM_BP_FAT | epithelium development | 16 | 1.50E-02 |
| GOTERM_BP_FAT | morphogenesis of an epithelium | 10 | 6.90E-02 |
| GOTERM_BP_FAT | epithelial tube morphogenesis | 6 | 2.30E-01 |
|  |  |  |  |
| **Cluster 10** | **Enrichment Score:2.15** | **Count** | **P-value** |
| GOTERM_BP_FAT | programmed cell death | 27 | 2.80E-04 |
| GOTERM_BP_FAT | apoptosis | 26 | 4.70E-04 |
| GOTERM_BP_FAT | death | 28 | 6.70E-04 |
| GOTERM_BP_FAT | cell death | 27 | 1.10E-03 |
| SP_PIR_KEYWORDS | Apoptosis | 14 | 2.00E-02 |
| GOTERM_BP_FAT | regulation of programmed cell death | 29 | 1.40E-01 |
| GOTERM_BP_FAT | regulation of cell death | 29 | 1.40E-01 |
| GOTERM_BP_FAT | regulation of apoptosis | 28 | 1.70E-01 |
|  |  |  |  |
| **Cluster 11** | **Enrichment Score:2.11** | **Count** | **P-value** |
| GOTERM_CC_FAT | anchored to membrane | 14 | 9.60E-05 |
| SP_PIR_KEYWORDS | gpi-anchor | 9 | 4.60E-03 |
| GOTERM_CC_FAT | anchored to plasma membrane | 5 | 5.00E-03 |
| SP_PIR_KEYWORDS | lipoprotein | 26 | 9.30E-03 |
| UP_SEQ_FEATURE | propeptide:Removed in mature form | 10 | 7.90E-02 |
| UP_SEQ_FEATURE | lipid moiety-binding region:GPI-anchor amidated serine | 4 | 1.30E-01 |
|  |  |  |  |
| **Cluster 12** | **Enrichment Score:2.04** | **Count** | **P-value** |
| KEGG_PATHWAY | Hedgehog signaling pathway | 8 | 1.40E-03 |
| KEGG_PATHWAY | Melanogenesis | 10 | 3.00E-03 |
| KEGG_PATHWAY | Wnt signaling pathway | 11 | 2.00E-02 |
| KEGG_PATHWAY | Basal cell carcinoma | 5 | 8.50E-02 |
|  |  |  |  |
| **Cluster 13** | **Enrichment Score:1.79** | **Count** | **P-value** |
| GOTERM_CC_FAT | neuron projection | 26 | 4.20E-03 |
| GOTERM_CC_FAT | cell projection | 38 | 4.20E-03 |
| GOTERM_CC_FAT | axon | 15 | 5.00E-03 |
| GOTERM_CC_FAT | cell soma | 15 | 3.70E-02 |
| GOTERM_CC_FAT | cell projection part | 15 | 3.80E-02 |
| GOTERM_CC_FAT | dendrite | 12 | 1.40E-01 |
|  |  |  |  |
| **Cluster 14** | **Enrichment Score:1.76** | **Count** | **P-value** |
| GOTERM_BP_FAT | tube development | 21 | 2.00E-04 |
| GOTERM_BP_FAT | gut development | 7 | 3.70E-03 |
| GOTERM_BP_FAT | lung development | 10 | 1.00E-02 |
| GOTERM_BP_FAT | respiratory tube development | 10 | 1.10E-02 |
| GOTERM_BP_FAT | respiratory system development | 10 | 1.50E-02 |
| GOTERM_BP_FAT | gland development | 14 | 1.50E-02 |
| GOTERM_BP_FAT | digestive system development | 5 | 1.80E-02 |
| GOTERM_BP_FAT | determination of bilateral symmetry | 5 | 2.90E-02 |
| GOTERM_BP_FAT | determination of symmetry | 5 | 2.90E-02 |
| GOTERM_BP_FAT | heart development | 12 | 9.30E-02 |
| GOTERM_BP_FAT | determination of left/right symmetry | 4 | 1.10E-01 |
| GOTERM_BP_FAT | gland morphogenesis | 5 | 2.90E-01 |
|  |  |  |  |
| **Cluster 15** | **Enrichment Score:1.73** | **Count** | **P-value** |
| GOTERM_MF_FAT | transcription factor binding | 20 | 5.90E-03 |
| GOTERM_MF_FAT | transcription coactivator activity | 8 | 2.60E-02 |
| GOTERM_MF_FAT | transcription cofactor activity | 11 | 4.20E-02 |

**Dataset S7. Enriched GO clusters for differentially-regulated genes by H3K27me3**

| **Cluster 1** | **Enrichment Score: 9.81** | **Count** | **P Value** |
| --- | --- | --- | --- |
| GOTERM_BP_FAT | positive regulation of nucleobase, nucleoside, nucleotide and nucleic acid metabolic process | 46 | 1.10E-12 |
| GOTERM_BP_FAT | positive regulation of nitrogen compound metabolic process | 46 | 3.00E-12 |
| GOTERM_BP_FAT | positive regulation of cellular biosynthetic process | 46 | 2.10E-11 |
| GOTERM_BP_FAT | positive regulation of macromolecule metabolic process | 52 | 2.50E-11 |
| GOTERM_BP_FAT | positive regulation of biosynthetic process | 46 | 3.80E-11 |
| GOTERM_BP_FAT | positive regulation of macromolecule biosynthetic process | 44 | 4.70E-11 |
| GOTERM_BP_FAT | positive regulation of RNA metabolic process | 37 | 5.40E-11 |
| GOTERM_BP_FAT | positive regulation of gene expression | 40 | 1.30E-10 |
| GOTERM_BP_FAT | positive regulation of transcription, DNA-dependent | 36 | 1.80E-10 |
| GOTERM_BP_FAT | positive regulation of transcription | 39 | 2.40E-10 |
| GOTERM_BP_FAT | positive regulation of transcription from RNA polymerase II promoter | 29 | 4.00E-08 |
| GOTERM_BP_FAT | regulation of transcription from RNA polymerase II promoter | 33 | 5.60E-06 |
|  |  |  |  |
| **Cluster 2** | **Enrichment Score: 8.02** | **Count** | **P Value** |
| GOTERM_BP_FAT | regulation of transcription | 73 | 3.60E-09 |
| GOTERM_BP_FAT | regulation of RNA metabolic process | 61 | 9.60E-09 |
| GOTERM_BP_FAT | regulation of transcription, DNA-dependent | 59 | 2.60E-08 |
|  |  |  |  |
| **Cluster 3** | **Enrichment Score: 6.42** | **Count** | **P Value** |
| SP_PIR_KEYWORDS | transcription regulation | 40 | 7.00E-08 |
| GOTERM_BP_FAT | transcription | 38 | 5.90E-07 |
| SP_PIR_KEYWORDS | Transcription | 39 | 1.40E-06 |
|  |  |  |  |
| **Cluster 4** | **Enrichment Score: 4.63** | **Count** | **P Value** |
| SP_PIR_KEYWORDS | Homeobox | 15 | 5.40E-06 |
| INTERPRO | Homeodomain-related | 14 | 1.20E-05 |
| INTERPRO | Homeobox | 14 | 1.50E-05 |
| INTERPRO | Homeobox, conserved site | 12 | 5.20E-05 |
| SMART | HOX | 14 | 1.40E-04 |
|  |  |  |  |
| **Cluster 5** | **Enrichment Score: 4.27** | **Count** | **P Value** |
| GOTERM_BP_FAT | regulation of cell motion | 16 | 2.00E-05 |
| GOTERM_BP_FAT | regulation of locomotion | 15 | 8.90E-05 |
| GOTERM_BP_FAT | regulation of cell migration | 14 | 9.00E-05 |
|  |  |  |  |
| **Cluster 6** | **Enrichment Score: 3.46** | **Count** | **P Value** |
| GOTERM_BP_FAT | positive regulation of cell motion | 11 | 8.20E-05 |
| GOTERM_BP_FAT | positive regulation of locomotion | 10 | 4.80E-04 |
| GOTERM_BP_FAT | positive regulation of cell migration | 9 | 1.10E-03 |
|  |  |  |  |
| **Cluster 7** | **Enrichment Score: 3.17** | **Count** | **P Value** |
| GOTERM_BP_FAT | mesenchymal cell development | 7 | 6.10E-04 |
| GOTERM_BP_FAT | mesenchymal cell differentiation | 7 | 6.80E-04 |
| GOTERM_BP_FAT | mesenchyme development | 7 | 7.60E-04 |
|  |  |  |  |
| **Cluster 8** | **Enrichment Score: 3.12** | **Count** | **P Value** |
| GOTERM_BP_FAT | regulation of apoptosis | 30 | 6.40E-04 |
| GOTERM_BP_FAT | regulation of programmed cell death | 30 | 7.90E-04 |
| GOTERM_BP_FAT | regulation of cell death | 30 | 8.40E-04 |
|  |  |  |  |
| **Cluster 9** | **Enrichment Score: 3.05** | **Count** | **P Value** |
| GOTERM_BP_FAT | response to steroid hormone stimulus | 19 | 1.00E-04 |
| GOTERM_BP_FAT | response to endogenous stimulus | 25 | 2.50E-03 |
| GOTERM_BP_FAT | response to hormone stimulus | 23 | 2.60E-03 |
|  |  |  |  |
| **Cluster 10** | **Enrichment Score: 3** | **Count** | **P Value** |
| GOTERM_BP_FAT | lung development | 10 | 8.30E-04 |
| GOTERM_BP_FAT | respiratory tube development | 10 | 9.40E-04 |
| GOTERM_BP_FAT | respiratory system development | 10 | 1.30E-03 |
|  |  |  |  |
| **Cluster 11** | **Enrichment Score: 2.79** | **Count** | **P Value** |
| GOTERM_BP_FAT | negative regulation of apoptosis | 18 | 1.40E-03 |
| GOTERM_BP_FAT | negative regulation of programmed cell death | 18 | 1.70E-03 |
| GOTERM_BP_FAT | negative regulation of cell death | 18 | 1.70E-03 |
|  |  |  |  |
| **Cluster 12** | **Enrichment Score: 2.66** | **Count** | **P Value** |
| GOTERM_BP_FAT | cell motion | 21 | 1.20E-03 |
| GOTERM_BP_FAT | cell migration | 15 | 2.20E-03 |
| GOTERM_BP_FAT | localization of cell | 17 | 3.00E-03 |
| GOTERM_BP_FAT | cell motility | 17 | 3.00E-03 |
|  |  |  |  |
| **Cluster 13** | **Enrichment Score: 2.55** | **Count** | **P Value** |
| GOTERM_BP_FAT | limb development | 10 | 3.80E-04 |
| GOTERM_BP_FAT | appendage development | 10 | 3.80E-04 |
| GOTERM_BP_FAT | appendage morphogenesis | 8 | 5.50E-03 |
| GOTERM_BP_FAT | limb morphogenesis | 8 | 5.50E-03 |
| GOTERM_BP_FAT | embryonic appendage morphogenesis | 7 | 1.10E-02 |
| GOTERM_BP_FAT | embryonic limb morphogenesis | 7 | 1.10E-02 |
|  |  |  |  |
| **Cluster 14** | **Enrichment Score: 2.47** | **Count** | **P Value** |
| GOTERM_BP_FAT | apoptosis | 19 | 1.70E-03 |
| GOTERM_BP_FAT | programmed cell death | 19 | 2.10E-03 |
| GOTERM_BP_FAT | cell death | 19 | 5.70E-03 |
| GOTERM_BP_FAT | death | 19 | 6.80E-03 |
|  |  |  |  |
| **Cluster 15** | **Enrichment Score: 2.43** | **Count** | **P Value** |
| GOTERM_BP_FAT | regulation of cardiac muscle tissue development | 4 | 2.10E-03 |
| GOTERM_BP_FAT | regulation of cardiac muscle growth | 4 | 2.10E-03 |
| GOTERM_BP_FAT | regulation of cardiac muscle cell proliferation | 4 | 2.10E-03 |
| GOTERM_BP_FAT | regulation of heart growth | 4 | 2.70E-03 |
| GOTERM_BP_FAT | negative regulation of cardiac muscle cell proliferation | 3 | 2.90E-03 |
| GOTERM_BP_FAT | regulation of organ growth | 4 | 3.20E-02 |
|  |  |  |  |
| **Cluster 16** | **Enrichment Score: 2.35** | **Count** | **P Value** |
| GOTERM_MF_FAT | channel activity | 18 | 1.90E-03 |
| GOTERM_MF_FAT | passive transmembrane transporter activity | 18 | 1.90E-03 |
| GOTERM_MF_FAT | ion channel activity | 17 | 2.50E-03 |
| GOTERM_MF_FAT | substrate specific channel activity | 17 | 3.30E-03 |
| SP_PIR_KEYWORDS | ionic channel | 16 | 4.80E-03 |
| GOTERM_MF_FAT | gated channel activity | 13 | 1.30E-02 |
| GOTERM_MF_FAT | cation channel activity | 12 | 1.90E-02 |
|  |  |  |  |
| **Cluster 17** | **Enrichment Score: 2.05** | **Count** | **P Value** |
| GOTERM_BP_FAT | antigen processing and presentation of peptide antigen via MHC class I | 5 | 1.60E-03 |
| GOTERM_BP_FAT | antigen processing and presentation of peptide antigen | 5 | 1.10E-02 |
| GOTERM_BP_FAT | antigen processing and presentation | 6 | 3.90E-02 |
|  |  |  |  |
| **Cluster 18** | **Enrichment Score: 2.04** | **Count** | **P Value** |
| GOTERM_BP_FAT | somite specification | 3 | 1.50E-03 |
| GOTERM_BP_FAT | segment specification | 3 | 1.60E-02 |
| GOTERM_BP_FAT | embryonic pattern specification | 4 | 3.20E-02 |
|  |  |  |  |
| **Cluster 19** | **Enrichment Score: 1.87** | **Count** | **P Value** |
| GOTERM_MF_FAT | voltage-gated ion channel activity | 10 | 1.20E-02 |
| GOTERM_MF_FAT | voltage-gated channel activity | 10 | 1.20E-02 |
| GOTERM_MF_FAT | gated channel activity | 13 | 1.30E-02 |
| SP_PIR_KEYWORDS | voltage-gated channel | 9 | 1.80E-02 |
|  |  |  |  |
| **Cluster 20** | **Enrichment Score: 1.82** | **Count** | **P Value** |
| GOTERM_BP_FAT | somitogenesis | 5 | 6.10E-03 |
| GOTERM_BP_FAT | segmentation | 5 | 1.80E-02 |
| GOTERM_BP_FAT | embryonic pattern specification | 4 | 3.20E-02 |
