# Supplementary material for: Interplay of miR-137 and EZH2 contributes to the genome-wide redistribution of H3K27me3 underlying the Pb-induced memory impairment
Source: Cell Death Dis. 2019 Sep 11;10(9):671. doi: 10.1038/s41419-019-1912-7 (PMC6739382; doi:10.1038/s41419-019-1912-7)
Supplement: Supplementary file 2 — Supplementary Table 1 [file 41419_2019_1912_MOESM2_ESM.docx]

**Table S1. Primers used in this study**

| **Primers** | **Sequences (5’-3’)** | **Methods** |
| --- | --- | --- |
| EZH1F | CCAATACATCCGCCTCTGCTA | qPCR |
| EZH1R | CTGGACTAGCTTTCTGTTTCGTG | qPCR |
| UTXF | TCTAAGAATCTCGGGTAATGAGG | qPCR |
| UTXR | ATAGGCTCAAGAACCCGAAGA | qPCR |
| JMJD3F | ACTGCAACGAATGCGATGTG | qPCR |
| JMJD3R | GGCTGCATTCTCACTTGTAAC | qPCR |
| EZH2F | TGGGAAGAAATCTGAGAAGG | qPCR |
| EZH2R | TGGGTCTGCTACTGTTATTCG | qPCR |
| DicerF | GGGAAATGTGACCCAGACGAA | qPCR |
| DicerR | CAATCCACCACAATCTCACAAGG | qPCR |
| DroshaF | GCAAGAGTATGCCATCACCAA | qPCR |
| DroshaR | CTCAAGTGCGTCCATTGCTG | qPCR |
| Exportin5F | CCACCCAAGTCAGTTTCTACG | qPCR |
| Exportin5R | ACAGGTCCAGTGTCAATAGCAG | qPCR |
| ActinF | CCTGAAGTACCCCATTGAAC | qPCR |
| ActinR | GAGGTCTTTACGGATGTCAAC | qPCR |
| Pri-137F | CAAGAGTTCTTTCTGGTGGTG | qPCR |
| Pri-137R | GAAGATCCAGAACGAAACCA | qPCR |
| Wnt9bF | CCTGCCCTCTTCAACTTTACC | qPCR |
| Wnt9bR | AGCGGCGTTATTGGTCTGTC | qPCR |
| Wnt6F | GGGGTGGATGGGTGAGTTTAG | qPCR |
| Wnt6R | AAGGAGGGATGCGAGGTTTC | qPCR |
| Gsk3bF | CCTGCCCTCTTCAACTTTACC | qPCR |
| Gsk3bR | AGCGGCGTTATTGGTCTGTC | qPCR |
| miR-124-3p | GCTAAGGCACGCGGTG | miRNA profiling |
| miR-137-3p | GCCGGCTTATTGCTTAAGAATAC | miRNA profiling |
| miR-101-3p | GCGCGCTACAGTACTGTGATA | miRNA profiling |
| miR-26b-5p | GCGCCTTCAAGTAATTCAGG | miRNA profiling |
| miR-26a-5p | GCGCTTCAAGTAATCCAGGA | miRNA profiling |
| miR-138-5p | GCCAGCTGGTGTTGTGAATC | miRNA profiling |
| miR-144-3p | GCGCGCCTACAGTATAGATGA | miRNA profiling |
| miR-25-3p | CGCATTGCACTTGTCTCG | miRNA profiling |
| miR-214-3p | CTTGACAGCAGGCACAGAC | miRNA profiling |
| miR-92b-3p | GCTATTGCACTCGTCCCG | miRNA profiling |
| miR-30d-5p | GGCTGTAAACATCCCCGAC | miRNA profiling |
| miRNA-R | GTGCAGGGTCCGAGGT | miRNA profiling |
| U6 snRNAF | CTCGCTTCGGCAGCACA | miRNA profiling |
| U6 snRNAR | AACGCTTCACGAATTTGCGT | miRNA profiling |
| 5S rRNAF | TCTCGTCTGATCTCGGAAGC | miRNA profiling |
| 5S rRNAR | AGCCTACAGCACCCGGTATT | miRNA profiling |
| Wnt9bCF | GCCATCGACCAAGGTGTCA | ChIP |
| Wnt9bCR | AGTCTGGTTCCTCCTCCTGTG | ChIP |
| Wnt6CF | GTCAAACGTCTCCCAGCTAGTC | ChIP |
| Wnt6CR | TTGCCTCCGTAGGGTTGTC | ChIP |
| miR-137CF | TAGGCTGTAGTCGGTGGGAAG | ChIP |
| miR-137CR | CAGGCAGACCAACTCACTCATC | ChIP |
| GAPDHCF | CTCCATTTCCCTGGTTCCTG | ChIP |
| GAPDHCR | TCCAGGACCCAGAAACCAGA | ChIP |
