# Supplementary figures and images for: Interplay of miR-137 and EZH2 contributes to the genome-wide redistribution of H3K27me3 underlying the Pb-induced memory impairment
Source: Cell Death Dis. 2019 Sep 11;10(9):671. doi: 10.1038/s41419-019-1912-7 (PMC6739382; doi:10.1038/s41419-019-1912-7)

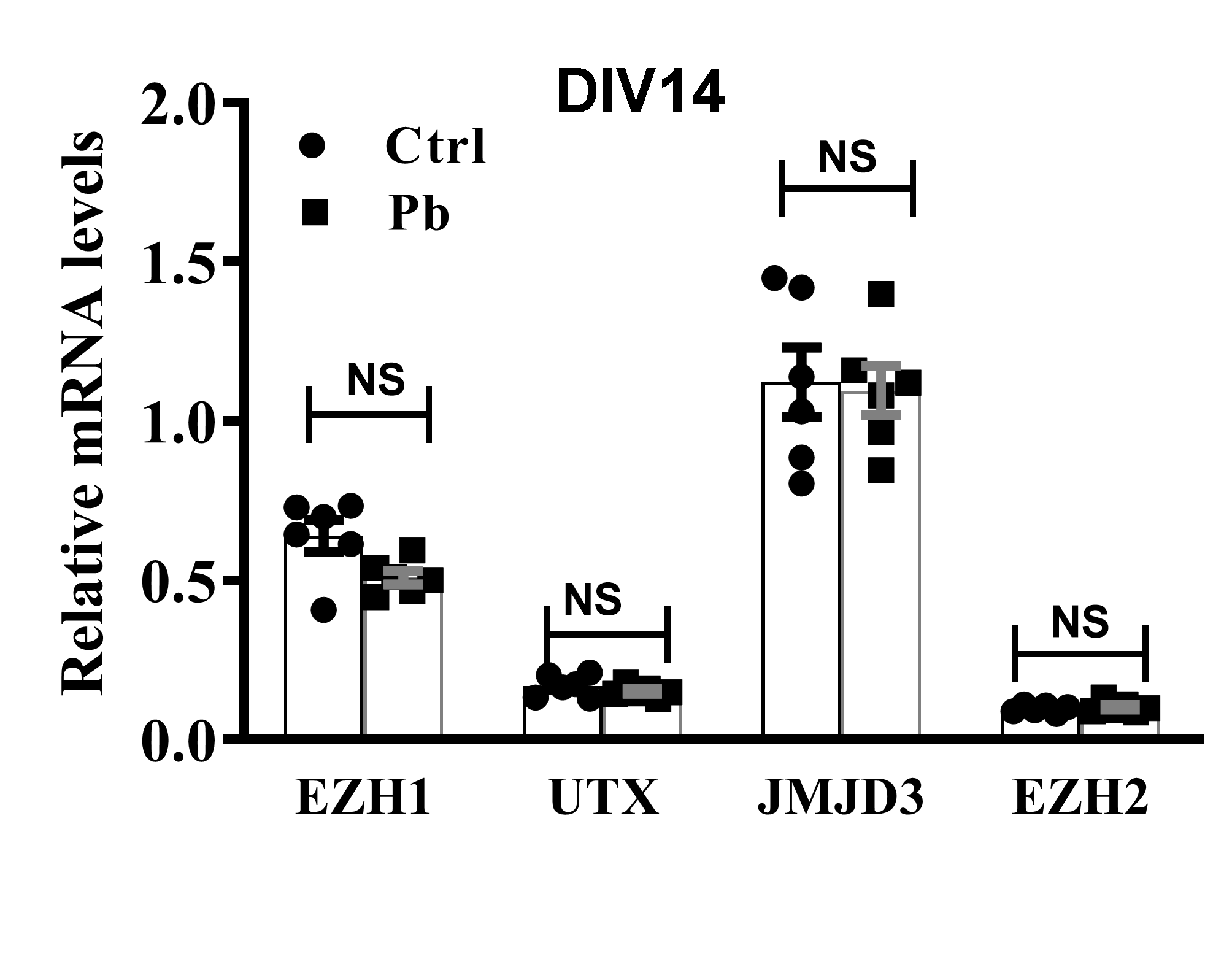

Supplement: Supplementary file 3 — Supplementary Figure 1 [file 41419_2019_1912_MOESM3_ESM.tif]

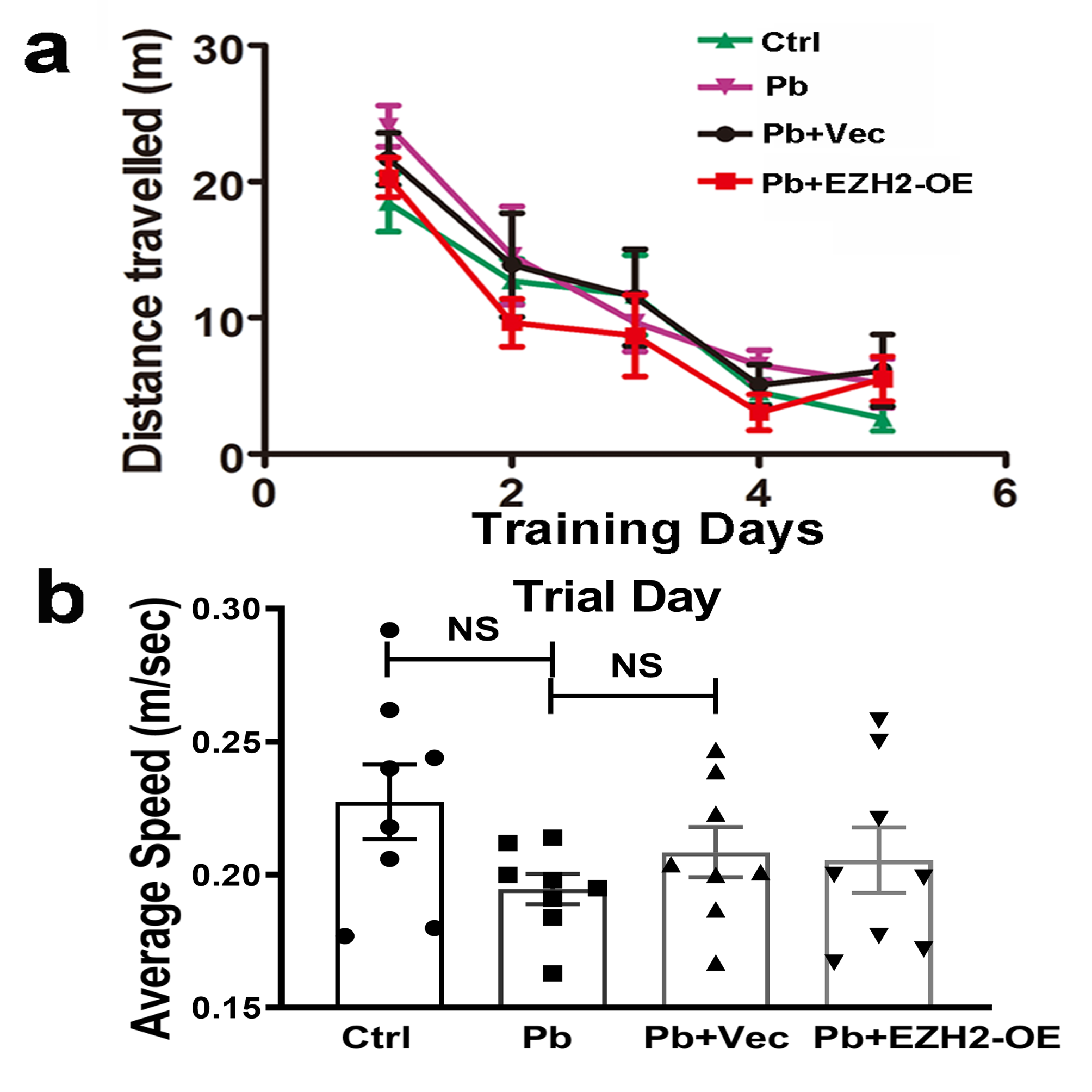

Supplement: Supplementary file 4 — Supplementary Figure 2 [file 41419_2019_1912_MOESM4_ESM.tif]

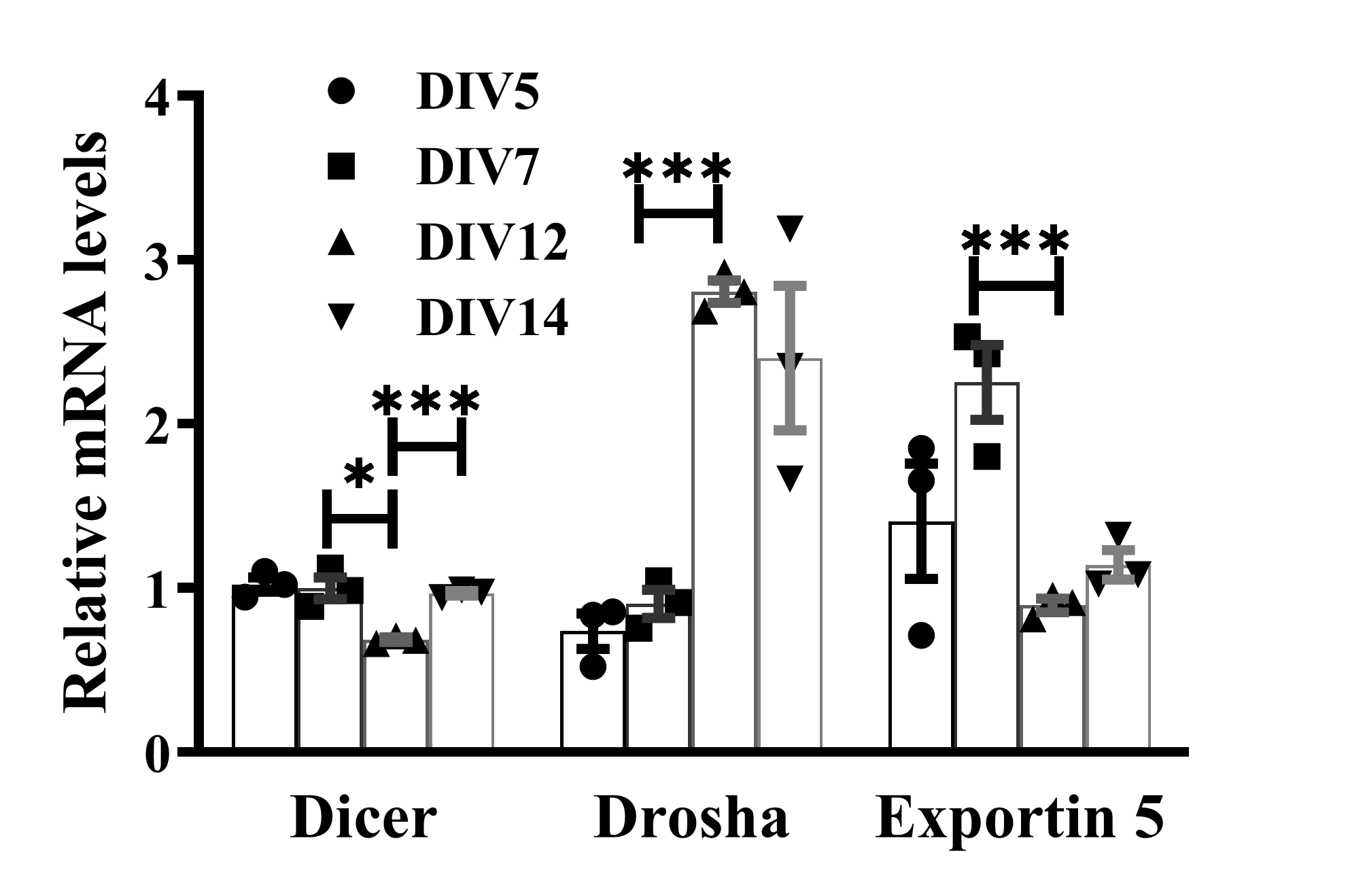

Supplement: Supplementary file 5 — Supplementary Figure 3 [file 41419_2019_1912_MOESM5_ESM.tif]

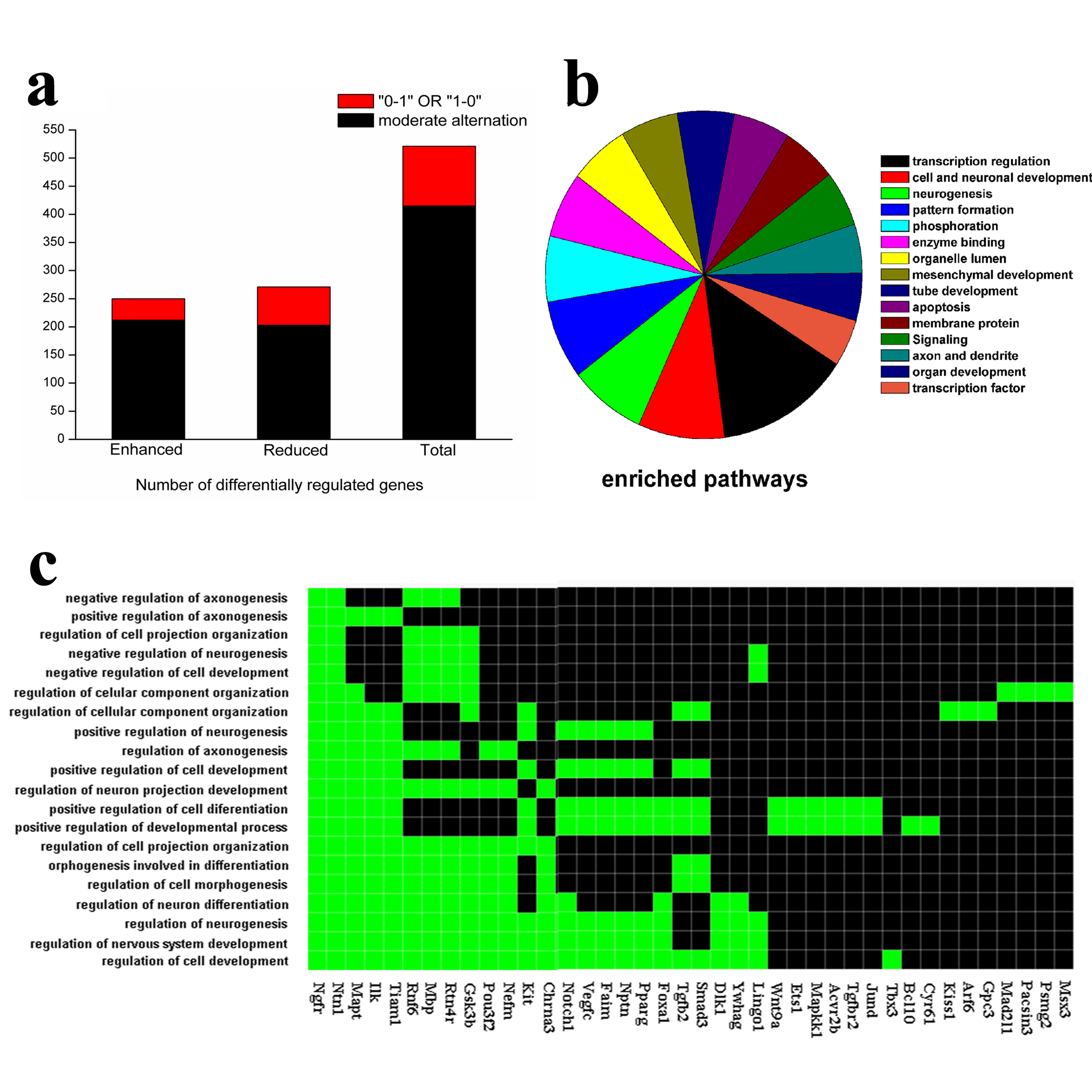

Supplement: Supplementary file 6 — Supplementary Figure 4 [file 41419_2019_1912_MOESM6_ESM.tif]

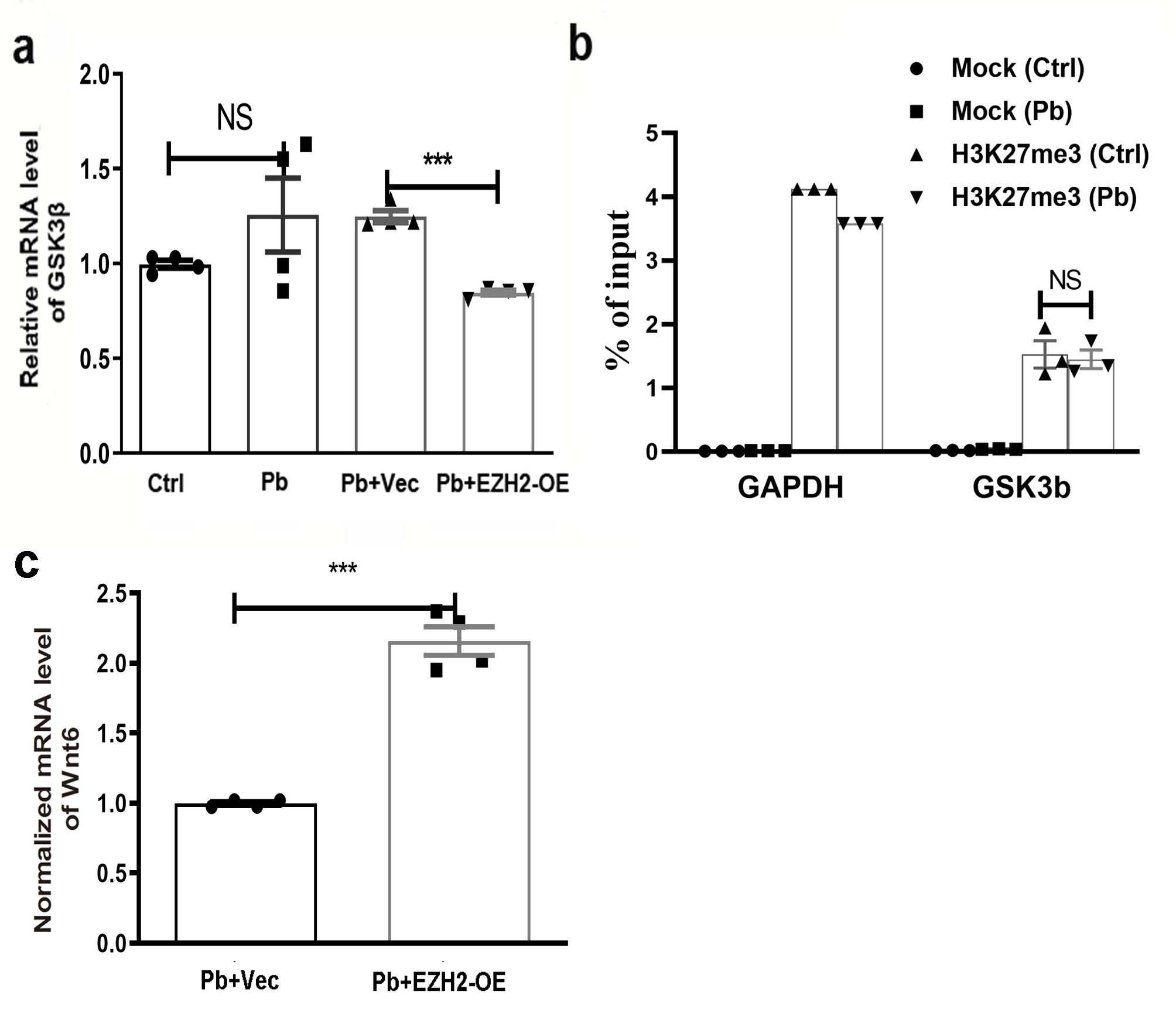

Supplement: Supplementary file 7 — Supplementary Figure 5 [file 41419_2019_1912_MOESM7_ESM.tif]

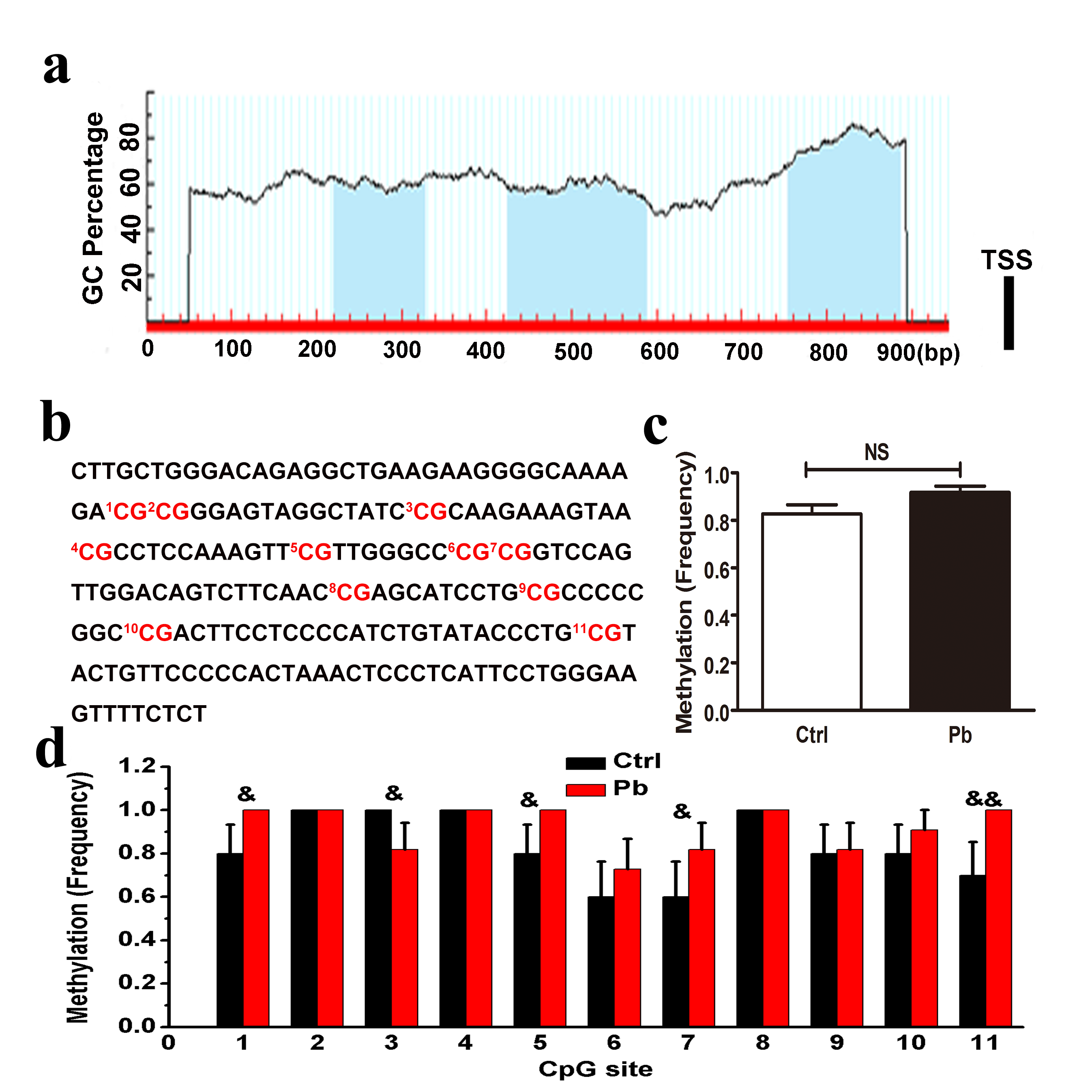

Supplement: Supplementary file 8 — Supplementary Figure 6 [file 41419_2019_1912_MOESM8_ESM.tif]
