# Supplementary material for: Interplay of miR-137 and EZH2 contributes to the genome-wide redistribution of H3K27me3 underlying the Pb-induced memory impairment
Source: Cell Death Dis. 2019 Sep 11;10(9):671. doi: 10.1038/s41419-019-1912-7 (PMC6739382; doi:10.1038/s41419-019-1912-7)
Supplement: Supplementary file 9 — Dataset 1 [file 41419_2019_1912_MOESM9_ESM.pdf]

List of genes regulated by H3K27me3 in the absence of Pb

Accession	Symbol	Gene Name
NM_133411	Abcc4	multidrug resistance-associated protein 4
NM_001107186	Abl2	tyrosine-protein kinase ABL2
NM_022190	Acan	aggrecan core protein
NM_001012013	Acbd4	acyl-CoA-binding domain-containing protein 4
NM_001126079	Acbd7	acyl-CoA-binding domain-containing protein 7
NM_001106508	Acoxl	acyl-coenzyme A oxidase-like protein
NM_012893	Actg2	actin, gamma-enteric smooth muscle
NM_001170325	Actn2	actinin alpha 2
NM_001039028	Actr1b	ARP1 actin-related protein 1 homolog B
NM_001107239	Adcy1	adenylate cyclase type 1
NM_133511	Adcyap1r1	pituitary adenylate cyclase-activating
NM_001013054	Adprhl1	[Protein ADP-ribosylarginine] hydrolase-like
NM_001134744	Agpat5	1-acyl-sn-glycerol-3-phosphate acyltransferase
NM_001007654	Agtrap	type-1 angiotensin II receptor-associated
NM_030986	Ak2	adenylate kinase 2, mitochondrial isoform a
NM_001033967	Ak2	adenylate kinase 2, mitochondrial isoform b
NM_001001801	Akap7	A-kinase anchoring protein 18 ,isoform delta
NM_053896	Aldh1a2	retinal dehydrogenase 2
NM_153301	Alox15b	arachidonate 15-lipoxygenase B
NM_012902	Amh	muellerian-inhibiting factor precursor
NM_001191565	Ankrd33b	ankyrin repeat domain-containing protein 33B
NM_001008523	Aox4	aldehyde oxidase 4
NM_031008	Ap2a2	AP-2 complex subunit alpha-2
NM_031779	Apba1	amyloid beta A4 precursor protein-binding family
NM_012779	Aqp5	aquaporin-5
NM_024152	Arf6	ADP-ribosylation factor 6
NM_001004242	Arhgap8	rho GTPase-activating protein 8
NM_001012198	Arhgap9	rho GTPase-activating protein 9 isoform 2

NM_001080789	Arhgap9	rho GTPase-activating protein 9 isoform 1
NM_001106061	Arhgef3	rho guanine nucleotide exchange factor 3
NM_001173981	Arid3c	AT-rich interactive domain-containing protein
NM_001037767	Arpc5l	actin-related protein 2/3 complex subunit 5-like
NM_001047881	Arsi	arylsulfatase I precursor
NM_198735	Art2b	ADP-ribosyltransferase 2b
NM_053397	Artn	artemin precursor
NM_001108420	Asb13	ankyrin repeat and SOCS box-containing 13
NM_001106389	Asf1a	ASF1 anti-silencing function 1 homolog A
NM_001035002	Atad1	ATPase family AAA domain-containing protein 1
NM_024403	Atf4	cyclic AMP-dependent transcription factor ATF-4
NM_012913	Atp1b3	sodium/potassium-transporting ATPase subunit
NM_023093	Atp5a1	ATP synthase subunit alpha, mitochondrial
NM_001106068	B3gnt3	UDP-GlcNAc:betaGal
NM_001107113	Bach1	transcription regulator protein BACH1
NM_022300	Basp1	brain acid soluble protein 1
NM_001025767	Blnk	B-cell linker protein
NM_001128187	Bnipl	BCL2/adenovirus E1B 19kD interacting protein
NM_001007707	Brp16	brain protein 16
NM_001166344	Btnl4	butyrophilin subfamily 3 member A2
NM_212489	Btnl8	butyrophilin-like 8
NM_001105949	C1ql2	complement C1q-like protein 2
NM_001106555	C8g	complement component C8 gamma chain
NM_175595	Cacna2d3	voltage-dependent calcium channel subunit
NM_053351	Cacng2	voltage-dependent calcium channel gamma-2
NM_080694	Cacng6	voltage-dependent calcium channel gamma-6
NM_138513	Calcb	calcitonin gene-related peptide 2 precursor
NM_031338	Camkk2	calcium/calmodulin-dependent protein kinase
NM_019174	Car4	carbonic anhydrase 4 precursor
NM_001130554	Card10	caspase recruitment domain-containing protein

NM_001107071	Cbx2	chromobox protein homolog 2
NM_199117	Cbx7	chromobox protein homolog 7
NM_001014091	Ccdc33	coiled-coil domain-containing protein 33
NM_053662	Ccnl1	cyclin-L1
NM_001166577	Cd300e	CMRF35-like molecule 2
NM_017124	Cd37	leukocyte antigen CD37
NM_013169	Cd3d	T-cell surface glycoprotein CD3 delta chain
NM_001077646	Cd3g	T-cell surface glycoprotein CD3 gamma chain
NM_134360	Cd40	tumor necrosis factor receptor superfamily
NM_022269	Cd55	decay accelerating factor 1
NM_017125	Cd63	CD63 antigen
NM_001015016	Cd72	B-cell differentiation antigen CD72
NM_001048044	Cdc42ep3	CDC42 effector protein (Rho GTPase binding) 3
NM_138889	Cdh13	cadherin-13
NM_131902	Cdkn2c	cyclin-dependent kinase 4 inhibitor C
NM_001025682	Cdr2	cerebellar degeneration-related protein 2
NM_024125	Cebpb	CCAAT/enhancer-binding protein beta
NM_001100514	Cep76	centrosomal protein 76kDa
NM_019164	Chad	chondroadherin precursor
NM_001170593	Chat	choline O-acetyltransferase
NM_021655	Chga	chromogranin-A
NM_052805	Chrna3	neuronal acetylcholine receptor subunit alpha-3
NM_001106268	Chsy1	chondroitin sulfate synthase 1
NM_001107307	Cilp2	cartilage intermediate layer protein 2
NM_053327	Clcnka	chloride channel protein ClC-Ka
NM_031702	Cldn7	claudin-7
NM_001107501	Clip3	CAP-Gly domain-containing linker protein 3
NM_022218	Cmklr1	chemokine-like receptor 1
NM_001109300	Cmtm7	CKLF-like MARVEL transmembrane domain-containing
NM_001108355	Cnot6l	CCR4-NOT transcription complex subunit 6-like

NM_001107236	Cobl	protein cordon-bleu
NM_001108710	Coch	cochlin
NM_001025721	Colec12	collectin-12
NM_182473	Corin	atrial natriuretic peptide-converting enzyme
NM_001109327	Coro1c	coronin-1C
NM_001002808	Cpa5	carboxypeptidase A5
NM_001105716	Crabp1	cellular retinoic acid-binding protein 1
NM_001004085	Crat	carnitine O-acetyltransferase
NM_133381	Crebbp	CREB-binding protein
NM_001024783	Creld1	cysteine-rich with EGF-like domain protein 1
NM_022501	Crip2	cysteine-rich protein 2
NM_017074	Cth	cystathionine gamma-lyase
NM_181087	Cyp26b1	cytochrome P450, family 26, subfamily b,
NM_001107495	Cyp2s1	cytochrome P450 2S1
NM_031327	Cyr61	protein CYR61 precursor
NM_001009644	Dbx1	homeobox protein DBX1
NM_030993	Ddn	dendrin
NM_001108246	Ddx3x	ATP-dependent RNA helicase DDX3X
NM_031801	Deaf1	deformed epidermal autoregulatory factor 1
NM_001029916	Depdc7	DEP domain-containing protein 7
NM_181088	Dfnb31	whirlin
NM_001105832	Dlx3	distal-less homeobox 3
NM_012943	Dlx5	homeobox protein DLX-5
NM_001173357	Dmkn	dermokine
NM_053706	Dmrt1	doublesex- and mab-3-related transcription
NM_001107597	Dmrt2	doublesex- and mab-3-related transcription
NM_053693	Dmtf1	cyclin-D-binding Myb-like transcription factor
NM_001024342	Dnai1	dynein intermediate chain 1, axonemal
NM_001108694	Dnajc11	dnaJ homolog subfamily C member 11
NM_001014194	Dnajc16	dnaJ homolog subfamily C member 16 precursor

NM_001130062	Dok7	protein Dok-7
NM_012546	Drd1a	D(1A) dopamine receptor
NM_001108141	Dscaml1	Down syndrome cell adhesion molecule-like 1
NM_024141	Duox2	dual oxidase 2 precursor
NM_001107767	Duoxa1	dual oxidase maturation factor 1
NM_001191965	Duoxa2	dual oxidase maturation factor 2
NM_001007006	Dusp13	testis and skeletal muscle-specific dual
NM_001037973	Dusp9	dual specificity protein phosphatase 9
NM_001172056	Dvl2	dishevelled 2
NM_019226	Dync1h1	cytoplasmic dynein 1 heavy chain 1
NM_001108506	Ebf3	transcription factor COE3
NM_001191076	Ebf4	transcription factor COE4
NM_001127541	Efcab4a	EF-hand calcium-binding domain-containing
NM_012842	Egf	pro-epidermal growth factor precursor
NM_053633	Egr2	early growth response protein 2
NM_001107602	Elovl3	elongation of very long chain fatty acids
NM_053927	Epb4.1l3	band 4.1-like protein 3
NM_138541	Epcam	epithelial cell adhesion molecule precursor
NM_001105994	Ephx4	epoxide hydrolase 4
NM_021687	Erbb4	receptor tyrosine-protein kinase erbB-4
NM_022604	Esm1	endothelial cell-specific molecule 1 precursor
NM_001107423	Esrp2	epithelial splicing regulatory protein 2
NM_001108343	Etnk2	ethanolamine kinase 2
NM_133537	Expi	extracellular peptidase inhibitor
NM_001109323	F8a1	factor VIII intron 22 protein
NM_001134834	Fahd2a	fumarylacetoacetate hydrolase domain-containing
NM_001106296	Fam57b	hypothetical protein LOC293493
NM_001014178	Fam69b	hypothetical protein LOC362090
NM_001106566	Fam73b	hypothetical protein LOC296623
NM_001014046	Fam82a2	regulator of microtubule dynamics protein 3

NM_001127578	Fam91a1	hypothetical protein LOC689997
NM_001108233	Farp2	FERM, RhoGEF and pleckstrin domain-containing
NM_001025730	Fbxw5	F-box/WD repeat-containing protein 5
NM_053843	Fcgr2a	low affinity immunoglobulin gamma Fc region
NM_001100682	Fcrla	Fc receptor-like A precursor
NM_144753	Fev	protein FEV
NM_001109224	Fezf1	fez family zinc finger protein 1
NM_130753	Fgf15	fibroblast growth factor 15
NM_130752	Fgf21	fibroblast growth factor 21
NM_133286	Fgf8	fibroblast growth factor 8
NM_001011913	Fignl1	fidgetin-like protein 1
NM_001013248	Foxb1	forkhead box B1
NM_001168584	Foxb2	forkhead box B2
NM_001191846	Foxo1	forkhead box protein O1
NM_031236	Fut1	galactoside 2-alpha-L-fucosyltransferase 1
NM_022005	Fxyd6	FXYD domain-containing ion transport regulator 6
NM_031802	Gabbr2	gamma-aminobutyric acid type B receptor subunit
NM_001039036	Gabpb1	GA repeat binding protein, beta 1
NM_024370	Gabrg3	gamma-aminobutyric acid receptor subunit gamma-3
NM_001005888	Galc	galactocerebrosidase
NM_001025053	Galnt4	polypeptide N-acetylgalactosaminyltransferase 4
NM_022926	Galnt7	N-acetylgalactosaminyltransferase 7
NM_001122644	Galnt9	polypeptide N-acetylgalactosaminyltransferase 9
NM_133293	Gata3	GATA binding protein 3
NM_144730	Gata4	transcription factor GATA-4
NM_019185	Gata6	transcription factor GATA-6
NM_053708	Gbx2	gastrulation brain homeobox 2
NM_019216	Gdf15	growth/differentiation factor 15 precursor
NM_017276	Gdi2	rab GDP dissociation inhibitor beta
NM_001037210	Gipc2	PDZ domain-containing protein GIPC2

NM_001004099	Gjb2	gap junction beta-2 protein
NM_019240	Gjb3	gap junction beta-3 protein
NM_013133	Glra1	glycine receptor subunit alpha-1
NM_001134413	Gltp	glycolipid transfer protein
NM_001107308	Gmip	GEM-interacting protein
NM_001191836	Gnal	guanine nucleotide-binding protein G(olf)
NM_012774	Gpc3	glypican-3 precursor
NM_001107285	Gpc5	glypican 5 precursor
NM_001025147	Gpr160	probable G-protein coupled receptor 160
NM_001108646	Gpr162	probable G-protein coupled receptor 162
NM_001191915	Gpr50	melatonin-related receptor
NM_001012057	Gpt2	alanine aminotransferase 2
NM_019282	Grem1	gremlin-1 precursor
NM_017010	Grin1	glutamate [NMDA] receptor subunit zeta-1
NM_012575	Grin2c	glutamate [NMDA] receptor subunit epsilon-3
NM_001109270	Grrp1	glycine/arginine-rich protein 1
NM_001001512	Gtf2i	general transcription factor II-I
NM_012578	H1f0	histone H1.0
NM_022696	Hand2	heart- and neural crest derivatives-expressed
NM_013064	Hcrtr1	orexin receptor type 1
NM_053447	Hdac2	histone deacetylase 2
NM_001108631	Herc3	probable E3 ubiquitin-protein ligase HERC3
NM_019236	Hes2	transcription factor HES-2
NM_022528	Hif3a	hypoxia-inducible factor 3-alpha
NM_017268	Hmgcs1	hydroxymethylglutaryl-CoA synthase, cytoplasmic
NM_001106303	Hmx2	homeobox protein HMX2
NM_001129878	Hoxa10	homeo box A10
NM_001191087	Hoxa6	homeobox protein Hox-A6
NM_001109233	Hoxa9	homeobox protein Hox-A7
NM_001107042	Hoxb3	homeo box B3

NM_001100787	Hoxb4	homeo box B4
NM_001191925	Hoxb5	homeo box B5
NM_001017480	Hoxb7	homeobox protein Hox-B7
NM_001100497	Hoxb9	homeo box B9
NM_001106796	Hoxc12	homeobox protein Hox-C12
NM_001105884	Hoxd1	homeobox protein Hox-D1
NM_001107094	Hoxd10	homeo box D10
NM_017122	Hpca	neuron-specific calcium-binding protein
NM_001135762	Hpse2	heparanase-2
NM_181370	Hs3st2	heparan sulfate glucosamine 3-O-sulfotransferase
NM_139329	Hsd3b7	3 beta-hydroxysteroid dehydrogenase type 7
NM_001106177	Hsf4	heat shock factor protein 4
NM_053612	Hspb8	heat shock protein beta-8
NM_024395	Htr5b	5-hydroxytryptamine receptor 5B
NM_022938	Htr7	5-hydroxytryptamine receptor 7
NM_031721	Htra1	serine protease HTRA1
NM_001107321	Htra4	probable serine protease HTRA4
NM_013159	Ide	insulin-degrading enzyme
NM_001082477	Igf1	insulin-like growth factor I isoform a
NM_001190163	Igf2	insulin-like growth factor II isoform 2
NM_031511	Igf2	insulin-like growth factor II isoform 1
NM_001107197	Igsf9	protein turtle homolog A precursor
NM_001107237	Ikzf1	IKAROS family zinc finger 1
NM_001107521	Il20ra	interleukin-20 receptor subunit alpha
NM_133409	Ilk	integrin-linked protein kinase
NM_172224	Impa2	inositol monophosphatase 2
NM_134417	Ipmk	inositol polyphosphate multikinase
NM_207617	Iqsec3	IQ motif and SEC7 domain-containing protein 3
NM_001013880	Isyna1	inositol-3-phosphate synthase 1
NM_001014116	Jmjd8	jmjC domain-containing protein 8

NM_138875	Jund	transcription factor jun-D
NM_001008814	Kb21	keratin, type II cuticular Hb1
NM_173095	Kcna1	potassium voltage-gated channel subfamily A
NM_053630	Kcnh4	potassium voltage-gated channel subfamily H
NM_145095	Kcnh8	potassium voltage-gated channel subfamily H
NM_130813	Kcnk15	potassium channel subfamily K member 15
NM_001039516	Kcnk5	potassium channel subfamily K member 5
NM_053806	Kcnk6	potassium channel, subfamily K, member 6
NM_023021	Kcnn4	intermediate conductance calcium-activated
NM_057202	Kif5b	kinesin-1 heavy chain
NM_001048215	Kirrel3	kin of IRRE-like protein 3
NM_023992	Kiss1r	kiSS-1 receptor
NM_022264	Kit	mast/stem cell growth factor receptor
NM_001107164	Klf1	Krueppel-like factor 1
NM_001106054	Klhl1	kelch-like protein 1
NM_001106252	Klk11	kallikrein-11
NM_017063	Kpnb1	importin subunit beta-1
NM_001109326	Krtap14	keratin-associated protein 14
NM_053538	Laptm5	lysosomal-associated transmembrane protein 5
NM_001007556	Lefty2	left-right determination factor 2
NM_001106784	Lgr5	leucine-rich repeat-containing G-protein coupled
NM_139036	Lhx5	LIM/homeobox protein Lhx5
NM_001107837	Lhx6	LIM/homeobox protein Lhx6
NM_001100722	Lingo1	leucine rich repeat and Ig domain containing 1
NM_001143803	LOC100233213	hypothetical protein LOC100233213
NM_001177829	LOC100365935	hypothetical protein LOC100365935
NM_001013941	LOC298795	hypothetical protein LOC298795
NM_001013979	LOC304131	TAK1-like protein
NM_001135992	LOC498276	Fc gamma receptor II beta
NM_001109221	LOC500034	hypothetical protein LOC500034

NM_001162931	LOC502128	POM121 membrane glycoprotein-like 2 isoform 2
NM_001162930	LOC502128	POM121 membrane glycoprotein-like 2 isoform 1
NM_001195277	LOC679651	transmembrane protein 178-like
NM_001109418	LOC680531	hypothetical protein LOC680531
NM_001109489	LOC685964	hypothetical protein LOC685964
NM_001163002	LOC689926	hypothetical protein LOC689926
NM_001109595	LOC690478	hypothetical protein LOC690478
NM_001109616	LOC691024	hypothetical protein LOC691024
NM_001170434	Lrrc32	leucine rich repeat containing 32
NM_021656	Ltb4r	leukotriene B4 receptor 1
NM_001109391	Mab21l2	protein mab-21-like 2
NM_019318	Maf	transcription factor Maf
NM_138503	Map3k2	mitogen-activated protein kinase kinase kinase
NM_001198638	Map7	ensconsin isoform 2
NM_017212	Mapt	microtubule-associated protein tau
NM_001107590	Marveld1	MARVEL domain-containing protein 1
NM_001109132	Marveld3	MARVEL domain-containing protein 3
NM_181089	MAST1	microtubule-associated serine/threonine-protein
NM_001108013	Matn3	matrilin-3
NM_001025289	Mbp	Golli-Mbp isoform 1
NM_001039005	Mcoln2	mucolipin-2
NM_030859	Mdk	midkine precursor
NM_001108837	Meox1	homeobox protein MOX-1
NM_017149	Meox2	homeobox protein MOX-2
NM_022943	Mertk	tyrosine-protein kinase Mer precursor
NM_001107531	Mesp1	mesoderm posterior protein 1
NM_001008518	MGC105649	normal mucosa of esophagus-specific gene 1
NM_001024890	MGC114520	hypothetical protein LOC315915
NM_001191889	Mid2	midline-2
NM_001108737	Mier2	mesoderm induction early response protein 2

NR_031814	Mir10a	
NR_031865	Mir124-3	
NR_031878	Mir132	
NR_031883	Mir137	
NR_031897	Mir181c	
NR_032266	Mir181d	
NR_031909	Mir193	
NR_031925	Mir212	
NR_031850	Mir34a	
NM_020102	Mos	proto-oncogene serine/threonine-protein kinase
NM_001034022	Mprip	myosin phosphatase Rho-interacting protein
NM_022529	Mrpl23	39S ribosomal protein L23, mitochondrial
NM_001108635	Mrpl53	39S ribosomal protein L53, mitochondrial
NM_001106628	Mrps35	28S ribosomal protein S35, mitochondrial
NM_053712	Msx3	homeo box, msh-like 3
NM_001100833	Mtch1	mitochondrial carrier homolog 1
NM_001191558	Mtss1l	MTSS1-like protein
NM_001106257	Mybpc2	myosin-binding protein C, fast-type
NM_057209	Mylk2	myosin light chain kinase 2, skeletal/cardiac
NM_053888	Myt1l	myelin transcription factor 1-like protein
NM_001013059	Ndfip1	NEDD4 family-interacting protein 1
NM_017029	Nefm	neurofilament medium polypeptide
NM_001013134	Nek4	serine/threonine-protein kinase Nek4
NM_001002851	Nenf	neudesin precursor
NM_012865	Nfya	nuclear transcription factor Y subunit alpha
NM_012610	Ngfr	tumor necrosis factor receptor superfamily
NM_001191733	Nhs	Nance-Horan syndrome protein
NM_001170476	Nkx1-2	NK1 homeobox 2
NM_013093	Nkx2-1	homeobox protein Nkx-2.1
NM_001107594	Nkx2-3	homeobox protein Nkx-2.3

NM_053651	Nkx2-5	homeobox protein Nkx-2.5
NM_134336	Nlgn3	neuroligin-3 precursor
NM_001105721	Notch1	neurogenic locus notch homolog protein 1
NM_153293	Npb	neuropeptide B precursor
NM_203340	Npm2	nucleoplasmin-2
NM_019380	Nptn	neuroplastin
NM_031628	Nr4a3	nuclear receptor subfamily 4 group A member 3
NM_024140	Nrgn	neurogranin
NM_001107337	Nsd1	histone-lysine N-methyltransferase, H3 lysine-36
NM_001017452	Nsun7	NOL1/NOP2/Sun domain family, member 7
NM_001106465	Ntng1	netrin-G1
NM_001011891	Nubp2	cytosolic Fe-S cluster assembly factor NUBP2
NM_181363	Nudt6	nucleoside diphosphate-linked moiety X motif 6
NM_021680	Nxph4	neurexophilin-4 precursor
NM_001106269	Olig3	oligodendrocyte transcription factor 2
NM_001107848	Ophn1	oligophrenin-1
NM_001014024	Orai3	protein orai-3
NM_001107565	Oraov1	oral cancer overexpressed 1
NM_001012118	Osr2	protein odd-skipped-related 2
NM_012871	Oxtr	oxytocin receptor
NM_134353	Pabpc1	polyadenylate-binding protein 1
NM_017230	Padi3	protein-arginine deiminase type-3
NM_133531	Pank4	pantothenate kinase 4
NM_001191077	Paqr6	progestin and adipoQ receptor family member 6
NM_001035249	Parl	presenilins-associated rhomboid-like protein,
NM_001107787	Pax1	paired box protein Pax-1
NM_053710	Pax3	paired box 3
NM_001039539	Pax9	paired box protein Pax-9
NM_001169129	Pcdh19	protocadherin-19
NM_001129882	Pcgf5	polycomb group RING finger protein 5

NM_001100506	Pctk3	cell division protein kinase 18
NM_001009542	Pdcd10	programmed cell death protein 10
NM_031317	Pdgfc	platelet-derived growth factor C
NM_012802	Pdgfra	alpha-type platelet-derived growth factor
NM_001004072	Pdha1	pyruvate dehydrogenase E1 component subunit
NM_053826	Pdk1	[Pyruvate dehydrogenase [lipoamide]] kinase
NM_019374	Pdyn	proenkephalin-B preproprotein
NM_130401	Pdzk1ip1	PDZK1-interacting protein 1
NM_001109487	Pfn3	profilin-3
NM_001106198	Pgbd5	piggyBac transposable element-derived protein 5
NM_031784	Pias3	E3 SUMO-protein ligase PIAS3
NM_001105951	Pik3c2b	phosphatidylinositol-4-phosphate 3-kinase C2
NM_022602	Pim3	serine/threonine-protein kinase pim-3
NM_001105845	Plcd3	1-phosphatidylinositol-4,5-bisphosphate
NM_053758	Plce1	1-phosphatidylinositol-4,5-bisphosphate
NM_001134972	Plekhg2	pleckstrin homology domain-containing family G
NM_001108036	Plekhh1	pleckstrin homology domain containing, family H
NM_022533	Pllp	plasmolipin
NM_172085	Pou3f2	POU domain, class 3, transcription factor 2
NM_001108889	Pou4f3	POU class 4 homeobox 3
NM_022538	Ppap2a	lipid phosphate phosphohydrolase 1
NM_013196	Ppara	peroxisome proliferator-activated receptor
NM_001105968	Ppox	protoporphyrinogen oxidase
NM_144746	Ppp2r2d	serine/threonine-protein phosphatase 2A 55 kDa
NM_001108577	Ppp2r4	serine/threonine-protein phosphatase 2A
NM_001106613	Ppp4r2	protein phosphatase 4, regulatory subunit 2
NM_134449	Prkcdbp	protein kinase C delta-binding protein
NM_001033963	Prkx	serine/threonine-protein kinase PRKX
NM_001038588	Prodh2	probable proline dehydrogenase 2
NM_001024305	Prpf38b	pre-mRNA-splicing factor 38B

NM_001109027	Prss33	serine protease 33
NM_001107395	Psd2	PH and SEC7 domain-containing protein 2
NM_019126	Psg19	carcinoembryonic antigen gene family (CGM3)
NM_130430	Psmd9	26S proteasome non-ATPase regulatory subunit 9
NM_001106138	Psmg2	tumor necrosis factor superfamily, member
NM_022516	Ptbp1	polypyrimidine tract-binding protein 1 isoform
NM_053964	Ptf1a	pancreas transcription factor 1 subunit alpha
NM_001108507	Pwwp2b	PWWP domain-containing protein 2B
NM_001108962	R3hdml	R3H domain (binds single-stranded nucleic acids)
NM_001109005	Rab23	ras-related protein Rab-23
NM_031718	Rab2a	ras-related protein Rab-2A
NM_053741	Rap2a	RAS related protein 2a
NM_001108273	Rasgef1c	ras-GEF domain-containing family member 1C
NM_001170531	Rasgrf1	ras-specific guanine nucleotide-releasing factor
NM_001105753	Rasgrf1	ras-specific guanine nucleotide-releasing factor
NM_001106261	Rasip1	ras-interacting protein 1
NM_001106317	Rassf7	ras association domain-containing protein 7
NM_053678	Rax	retinal homeobox protein Rx
NM_013162	Rbp4	retinol-binding protein 4 precursor
NM_001127490	Rfx7	regulatory factor X domain containing 2
NM_001004268	RGD1303271	hypothetical protein LOC313018
NM_001134560	RGD1305627	hypothetical protein LOC314467
NM_001106551	RGD1306208	hypothetical protein LOC296483
NM_001107663	RGD1307225	hypothetical protein LOC310269
NM_001134596	RGD1308299	hypothetical protein LOC367214
NM_001108129	RGD1309188	hypothetical protein LOC315463
NM_001107161	RGD1310262	hypothetical protein LOC304650
NM_001079705	RGD1311558	shootin-1
NM_001127526	RGD1311605	hypothetical protein LOC298841
NM_001109262	RGD1559493	hypothetical protein LOC500516

NM_001108678	RGD1559909	hypothetical protein LOC362592
NM_001106014	RGD1560394	hypothetical protein LOC289728
NM_001109345	RGD1563349	hypothetical protein LOC502727
NM_001109311	RGD1563692	hypothetical protein LOC501185
NM_001109234	RGD1564419	hypothetical protein LOC500128
NM_001109292	RGD1564560	hypothetical protein LOC500988
NM_001109067	RGD1565883	hypothetical protein LOC498193
NM_001134589	RGD1566265	hypothetical protein LOC363487
NM_001013133	Rhobtb2	rho-related BTB domain-containing protein 2
NM_001100488	Rimbp2	RIMS-binding protein 2
NM_053945	Rims2	regulating synaptic membrane exocytosis protein
NM_001106836	Rnf111	E3 ubiquitin-protein ligase Arkadia
NM_001173349	Rnf128	E3 ubiquitin-protein ligase RNF128
NM_001191093	Rnf150	RING finger protein 150
NM_053338	Rrad	GTP-binding protein RAD
NM_001048184	Rragc	ras-related GTP-binding protein C
NM_001106641	Rragd	ras-related GTP-binding protein D
NM_001025740	Rrm2	ribonucleoside-diphosphate reductase subunit M2
NM_001008346	Rrp8	ribosomal RNA-processing protein 8
NM_001008827	RT1-A1	RT1 class Ia, locus A1
NM_001008832	RT1-CE1	RT1 class I, locus CE1
NM_001008833	RT1-CE10	RT1 class I, locus CE10
NM_001033985	RT1-CE14	RT1 class I, locus CE14 isoform 2
NM_001008840	RT1-CE2	RT1 class I, locus CE2
NM_012645	RT1-EC2	class I histocompatibility antigen, Non-RT1.A
NM_001008848	RT1-Ha	RT1 class II, locus Ha
NM_181380	Rtn4rl2	reticulon-4 receptor-like 2 precursor
NM_001109471	S100a7a	protein S100-A15A
NM_022394	Safb	scaffold attachment factor B1
NM_001013985	Sccpdh	probable saccharopine dehydrogenase

NM_198748	Scin	adseverin
NM_017247	Scn10a	sodium channel protein type 10 subunit alpha
NM_001008880	Scn4b	sodium channel subunit beta-4 precursor
NM_012648	Scnn1b	amiloride-sensitive sodium channel subunit beta
NM_022670	Sct	secretin precursor
NM_177929	Sdccag8	serologically defined colon cancer antigen 8
NM_001107637	Sec63	translocation protein SEC63 homolog
NM_001166396	Selv	selenoprotein V
NM_017308	Sema6c	semaphorin-6C precursor
NM_001173429	Sept6	septin-6
NM_022616	Sept7	septin-7 isoform a
NM_001109104	Serp2	stress-associated endoplasmic reticulum protein
NM_001008776	Serpina11	serpin A11 isoform 1
NM_053779	Serpini1	neuroserpin precursor
NM_031647	Sfmbt1	scm-like with four MBT domains protein 1
NM_001105937	Sgsm1	small G protein signaling modulator 1
NM_053360	Sh3kbp1	SH3 domain-containing kinase-binding protein 1
NM_134457	Siah2	E3 ubiquitin-protein ligase SIAH2
NM_021693	Sik1	serine/threonine-protein kinase SIK1
NM_001107641	Sim1	single-minded homolog 1
NM_001004089	Sipa1	signal-induced proliferation-associated protein
NM_053759	Six1	sine oculis-related homeobox 1 homolog
NM_023990	Six3	homeobox protein SIX3
NM_031798	Slc12a2	solute carrier family 12 member 2
NM_134363	Slc12a5	solute carrier family 12 member 5
NM_153625	Slc12a8	solute carrier family 12 member 8
NM_147216	Slc16a2	monocarboxylate transporter 8
NM_053427	Slc17a6	vesicular glutamate transporter 2
NM_031663	Slc18a3	vesicular acetylcholine transporter
NM_001106327	Slc22a20	solute carrier family 22 member 20

NM_019230	Slc22a3	solute carrier family 22 member 3
NM_017316	Slc23a2	solute carrier family 23 member 2
NM_019214	Slc26a4	pendrin
NM_031736	Slc27a2	very long-chain acyl-CoA synthetase
NM_133600	Slc31a1	high affinity copper uptake protein 1
NM_001107522	Slc35d3	solute carrier family 35 member D3
NM_001105950	Slc35f5	solute carrier family 35 member F5
NM_001191920	Slc47a2	multidrug and toxin extrusion protein 2
NM_130746	Slc5a6	sodium-dependent multivitamin transporter
NM_203334	Slc6a5	sodium- and chloride-dependent glycine
NM_017206	Slc6a6	sodium- and chloride-dependent taurine
NM_078620	Slc8a3	sodium/calcium exchanger 3 precursor
NM_001113335	Slc9a2	sodium/hydrogen exchanger 2 isoform 1
NM_022667	Slco2a1	solute carrier organic anion transporter family
NM_022953	Slit1	slit homolog 1 protein precursor
NM_030858	Smad7	mothers against decapentaplegic homolog 7
NM_206851	Smyd2	SET and MYND domain-containing protein 2
NM_001191563	Sorcs1	VPS10 domain-containing receptor SorCS1
NM_001106367	Sorcs3	VPS10 domain-containing receptor SorCS3
NM_019193	Sox10	transcription factor SOX-10
NM_001106850	Sox14	SRY (sex determining region Y)-box 14
NM_001107902	Sox17	transcription factor SOX-17
NM_001106530	Spag4l	SUN domain-containing protein 5
NM_001106125	Spag6l	sperm associated antigen 6-like
NM_199374	Spata18	spermatogenesis-associated protein 18
NM_001108549	Spata5	spermatogenesis-associated protein 5
NM_181388	Spg7	paraplegin
NM_133386	Sphk1	sphingosine kinase 1
NM_001039208	Spns1	protein spinster homolog 1
NM_172067	Spon1	spondin-1 precursor

NM_001106988	Spsb3	SPRY domain-containing SOCS box protein 3
NM_001135711	Srrp	35 kDa SR repressor protein
NM_012659	Sst	somatostatin precursor
NM_175597	Ssx2ip	afadin- and alpha-actinin-binding protein
NM_031704	Stx5	syntaxin-5
NM_031665	Stx6	syntaxin-6
NM_001100750	Suclg2	succinyl-CoA ligase [GDP-forming] subunit beta,
NM_001025125	Sumf2	sulfatase-modifying factor 2
NM_001107341	Susd3	sushi domain-containing protein 3
NM_022191	Syt6	synaptotagmin-6
NM_001025419	Tax1bp3	tax1-binding protein 3
NM_001013245	Tbca	tubulin-specific chaperone A
NM_001191070	Tbr1	T-box brain protein 1
NM_001108322	Tbx1	T-box transcription factor TBX1
NM_001108132	Tbx20	T-box 20
NM_181638	Tbx3	T-box transcription factor TBX3
NM_001107034	Tbx4	T-box transcription factor TBX4
NM_001009964	Tbx5	T-box transcription factor TBX5
NM_001032397	Tcf21	transcription factor 21
NM_001106896	Tcfap2b	transcription factor AP-2-beta
NM_201420	Tcfap2c	transcription factor AP-2 gamma
NM_001098216	Tead3	TEA domain family member 3
NM_201655	Tepp	testis, prostate and placenta-expressed protein
NM_012671	Tgfa	protransforming growth factor alpha
NM_001191840	Tgfb1i1	transforming growth factor beta-1-induced
NM_031131	Tgfb2	transforming growth factor beta-2 precursor
NM_019386	Tgm2	protein-glutamine gamma-glutamyltransferase 2
NM_001100558	Tiam1	T-cell lymphoma invasion and metastasis 1
NM_001172125	Tlx2	T-cell leukemia, homeobox 2
NM_001107015	Tm4sf5	transmembrane 4 L6 family member 5

NM_001108795	Tmeff2	tomoregulin-2
NM_001159625	Tmem116	transmembrane protein 116
NM_001106280	Tmem126b	transmembrane protein 126B
NM_001107476	Tmem150b	transmembrane protein 150B
NM_001191668	Tmem185b	transmembrane protein 185B
NM_001108045	Tmem63c	transmembrane protein 63C
NM_001017455	Tmem80	transmembrane protein 80
NM_001105806	Tmem93	transmembrane protein 93
NM_001127528	Tmprss13	transmembrane protease serine 13
NM_001108998	Tmprss4	transmembrane protease serine 4
NM_153311	Tmprss5	transmembrane protease serine 5
NM_001108873	Tnfrsf10b	tumor necrosis factor receptor superfamily,
NM_001191810	Tns1	tensin 1
NR_024118	Tnxa	
NM_019180	Tpsb2	tryptase beta-2 precursor
NM_013046	Trh	prothyroliberin
NM_130420	Trim9	E3 ubiquitin-protein ligase TRIM9
NM_001134837	Trps1	zinc finger transcription factor Trps1
NM_199088	Tsks	testis-specific serine kinase substrate
NM_001109227	Tspan33	tetraspanin-33
NM_001108815	Tspan7	tetraspanin-7
NM_012808	Tst	thiosulfate sulfurtransferase
NM_001109119	Tubb2a	tubulin beta-2A chain
NM_001025675	Tubb6	tubulin, beta 6
NM_001039163	Tusc5	tumor suppressor candidate 5 homolog
NM_001105723	Ubtf	nucleolar transcription factor 1 isoform 1
NM_001077660	Urg4	up-regulated gene 4
NM_022637	Vax2	ventral anterior homeobox 2
NM_001109546	Vsx1	visual system homeobox 1
NM_001169128	Vsx2	visual system homeobox 2

NM_001109312	Vwc2	brorin
NM_053751	Wap	whey acidic protein precursor
NM_001135894	Wdr25l	WD repeat domain 25-like
NM_001110489	Wdr86	WD repeat-containing protein 86
NM_031716	Wisp1	WNT1-inducible-signaling pathway protein 1
NM_001191556	Wnk2	serine/threonine-protein kinase WNK2
NM_175579	Wnk4	serine/threonine-protein kinase WNK4
NM_001108227	Wnt10a	protein Wnt-10a
NM_001191848	Wnt2b	protein Wnt-2b
NM_001105783	Wnt9a	protein Wnt-9a
NM_001107055	Wnt9b	protein Wnt-9b
NM_001106184	Wwp2	NEDD4-like E3 ubiquitin-protein ligase WWP2
NM_199383	Yipf1	protein YIPF1
NM_001014208	Yipf2	protein YIPF2
NM_001025747	Yipf6	protein YIPF6
NM_175604	Yrdc	yrdC domain-containing protein, mitochondrial
NM_019377	Ywhab	14-3-3 protein beta/alpha
NM_001130537	Zbtb39	zinc finger and BTB domain-containing protein
NM_001170577	Zfp167	zinc finger protein 167
NM_001135088	Zfp385a	zinc finger protein 385A isoform 1
NM_001135089	Zfp385a	zinc finger protein 385A isoform 3
NM_001109470	Zfp385a	zinc finger protein 385A isoform 2
NM_001108725	Zfyve21	zinc finger FYVE domain-containing protein 21
NM_203369	Zmynd11	zinc finger MYND domain-containing protein 11
NM_001030038	Znf518a	zinc finger protein 518A
NM_001024878	Znrf4	zinc/RING finger protein 4
NM_031616	Zranb2	zinc finger Ran-binding domain-containing
