# Supplementary material for: Interplay of miR-137 and EZH2 contributes to the genome-wide redistribution of H3K27me3 underlying the Pb-induced memory impairment
Source: Cell Death Dis. 2019 Sep 11;10(9):671. doi: 10.1038/s41419-019-1912-7 (PMC6739382; doi:10.1038/s41419-019-1912-7)
Supplement: Supplementary file 10 — Dataset 2 [file 41419_2019_1912_MOESM10_ESM.pdf]

List of genes regulated by H3K27me3 upon Pb exposure

Accession	Symbol	GeneName
NM_012690	Abcb4	multidrug resistance protein 2
NM_080582	Abcb6	ATP-binding cassette sub-family B member 6,
NM_133411	Abcc4	multidrug resistance-associated protein 4
NM_001014133	Abcg3l2	ATP-binding cassette, sub-family G (WHITE),
NM_001107186	Abl2	tyrosine-protein kinase ABL2
NM_001105814	Abr	active breakpoint cluster region-related
NM_001005902	Abtb1	ankyrin repeat and BTB/POZ domain-containing
NM_022190	Acan	aggrecan core protein
NM_001012013	Acbd4	acyl-CoA-binding domain-containing protein 4
NM_001170325	Actn2	actinin alpha 2
NM_001039028	Actr1b	ARP1 actin-related protein 1 homolog B
NM_031554	Acvr2b	activin receptor type-2B
NM_001108433	Adamts19	A disintegrin and metalloproteinase with
NM_198761	Adamts5	A disintegrin and metalloproteinase with
NM_001111057	Adarb1	double-stranded RNA-specific editase 1 isoform
NM_001107239	Adcy1	adenylate cyclase type 1
NM_138506	Adra2c	alpha-2C adrenergic receptor
NM_001134744	Agpat5	1-acyl-sn-glycerol-3-phosphate acyltransferase
NM_001007654	Agtrap	type-1 angiotensin II receptor-associated
NM_001134956	Ahdc1	A.T hook DNA-binding motif-containing protein 1
NM_001001801	Akap7	A-kinase anchoring protein 18 ,isoform delta
NM_053896	Aldh1a2	retinal dehydrogenase 2
NM_013059	Alpl	alkaline phosphatase, tissue-nonspecific isozyme
NM_012902	Amh	muellerian-inhibiting factor precursor
NM_001191565	Ankrd33b	ankyrin repeat domain-containing protein 33B
NM_001134699	Ankrd40	ankyrin repeat domain-containing protein 40
NM_001009676	Anks3	ankyrin repeat and SAM domain-containing protein
NM_001108331	Ap1s1	adaptor protein complex AP-1, sigma 1

NM_031008	Ap2a2	AP-2 complex subunit alpha-2
NM_031779	Apba1	amyloid beta A4 precursor protein-binding family
NM_012500	Apeh	acylamino-acid-releasing enzyme
NM_012778	Aqp1	aquaporin-1
NM_173105	Aqp11	aquaporin-11
NM_012779	Aqp5	aquaporin-5
NM_024152	Arf6	ADP-ribosylation factor 6
NM_001106061	Arhgef3	rho guanine nucleotide exchange factor 3
NM_001173981	Arid3c	AT-rich interactive domain-containing protein
NM_001013108	Arih1	ariadne ubiquitin-conjugating enzyme E2 binding
NM_001024906	Arl2bp	ADP-ribosylation factor-like protein 2-binding
NM_001106919	Arpc2	actin-related protein 2/3 complex subunit 2
NM_001106615	Arpc4	actin-related protein 2/3 complex subunit 4
NM_001037767	Arpc5l	actin-related protein 2/3 complex subunit 5-like
NM_001108420	Asb13	ankyrin repeat and SOCS box-containing 13
NM_001106389	Asf1a	ASF1 anti-silencing function 1 homolog A
NM_001035002	Atad1	ATPase family AAA domain-containing protein 1
NM_024403	Atf4	cyclic AMP-dependent transcription factor ATF-4
NM_012913	Atp1b3	sodium/potassium-transporting ATPase subunit
NM_012914	Atp2a3	sarcoplasmic/endoplasmic reticulum calcium
NM_023093	Atp5a1	ATP synthase subunit alpha, mitochondrial
NM_001106068	B3gnt3	UDP-GlcNAc:betaGal
NM_001012018	B4galt4	beta-1,4-galactosyltransferase 4
NM_001107113	Bach1	transcription regulator protein BACH1
NM_001106191	Banp	protein BANP
NM_022300	Basp1	brain acid soluble protein 1
NM_001191586	Bcor	BCL6 co-repressor
NM_031555	Bfsp1	filensin
NM_001009604	Bri3	brain protein I3
NM_001007707	Brp16	brain protein 16

NM_017259	Btg2	protein BTG2
NM_001008524	C1qc	complement C1q subcomponent subunit C precursor
NM_001108838	C1ql1	C1q-related factor
NM_001106555	C8g	complement component C8 gamma chain
NM_001107404	Cables1	CDK5 and ABL1 enzyme substrate 1
NM_175595	Cacna2d3	voltage-dependent calcium channel subunit
NM_012518	Calm3	calmodulin
NM_031338	Camkk2	calcium/calmodulin-dependent protein kinase
NM_001168549	Camsap1	calmodulin regulated spectrin-associated protein
NM_001013191	Cbfb	core-binding factor subunit beta
NM_001107071	Cbx2	chromobox protein homolog 2
NM_001191667	Ccdc64	bicaudal D-related protein 1
NM_001105725	Ccng2	cyclin-G2
NM_053662	Ccnl1	cyclin-L1
NM_022269	Cd55	decay accelerating factor 1
NM_001013103	Cdc34	ubiquitin-conjugating enzyme Cdc34
NM_053743	Cdc37	hsp90 co-chaperone Cdc37
NM_053620	Cdc42bpb	serine/threonine-protein kinase MRCK beta
NM_001048044	Cdc42ep3	CDC42 effector protein (Rho GTPase binding) 3
NM_053891	Cdk5r1	cyclin-dependent kinase 5 activator 1
NM_131902	Cdkn2c	cyclin-dependent kinase 4 inhibitor C
NM_001025682	Cdr2	cerebellar degeneration-related protein 2
NM_024125	Cebpb	CCAAT/enhancer-binding protein beta
NM_012831	Cebpg	CCAAT/enhancer-binding protein gamma
NM_001100514	Cep76	centrosomal protein 76kDa
NM_001105900	Cggbp1	CGG triplet repeat-binding protein 1
NM_019164	Chad	chondroadherin precursor
NM_021655	Chga	chromogranin-A
NM_017127	Chka	choline kinase alpha
NM_052805	Chrna3	neuronal acetylcholine receptor subunit alpha-3

NM_001011955	Chst1	carbohydrate sulfotransferase 1
NM_001107307	Cilp2	cartilage intermediate layer protein 2
NM_053327	Clcnka	chloride channel protein ClC-Ka
NM_031818	Clic4	chloride intracellular channel protein 4
NM_001107501	Clip3	CAP-Gly domain-containing linker protein 3
NM_001109300	Cmtm7	CKLF-like MARVEL transmembrane domain-containing
NM_001011942	Cnnm2	metal transporter CNNM2
NM_001007003	Cnot10	CCR4-NOT transcription complex subunit 10
NM_001108355	Cnot6l	CCR4-NOT transcription complex subunit 6-like
NM_001014232	Cnrip1	CB1 cannabinoid receptor-interacting protein 1
NM_182473	Corin	atrial natriuretic peptide-converting enzyme
NM_001109327	Coro1c	coronin-1C
NM_001002808	Cpa5	carboxypeptidase A5
NM_012836	Cpd	carboxypeptidase D precursor
NM_031766	Cpz	carboxypeptidase Z precursor
NM_001004085	Crat	carnitine O-acetyltransferase
NM_133381	Crebbp	CREB-binding protein
NM_001024783	Creld1	cysteine-rich with EGF-like domain protein 1
NM_022501	Crip2	cysteine-rich protein 2
NM_001014258	Crls1	cardiolipin synthase
NM_053335	Ctbp2	C-terminal-binding protein 2
NM_022266	Ctgf	connective tissue growth factor precursor
NM_001100661	Ctr9	Ctr9, Paf1/RNA polymerase II complex component,
NM_013156	Ctsl1	cathepsin L1 preproprotein
NM_057101	Cyp21a1	steroid 21-hydroxylase
NM_001107495	Cyp2s1	cytochrome P450 2S1
NM_001024779	Cyp2u1	cytochrome P450 2U1
NM_031327	Cyr61	protein CYR61 precursor
NM_031024	Dbn1	drebrin
NM_022297	Ddah1	N(G),N(G)-dimethylarginine

NM_001108246	Ddx3x	ATP-dependent RNA helicase DDX3X
NM_001013198	Ddx50	DEAD (Asp-Glu-Ala-Asp) box polypeptide 50
NM_031801	Deaf1	deformed epidermal autoregulatory factor 1
NM_001029916	Depdc7	DEP domain-containing protein 7
NM_001109577	Derl3	derlin-3
NM_181088	Dfnb31	whirlin
NM_001191597	Dhx15	DEAH (Asp-Glu-Ala-His) box polypeptide 15
NM_032063	Dll1	delta-like protein 1 precursor
NM_001173357	Dmkn	dermokine
NM_053693	Dmtf1	cyclin-D-binding Myb-like transcription factor
NM_001024342	Dnai1	dynein intermediate chain 1, axonemal
NM_001108694	Dnajc11	dnaJ homolog subfamily C member 11
NM_001105759	Dock9	dedicator of cytokinesis protein 9
NM_001130062	Dok7	protein Dok-7
NM_001108141	Dscaml1	Down syndrome cell adhesion molecule-like 1
NM_001107767	Duoxa1	dual oxidase maturation factor 1
NM_001105734	Dusp10	dual specificity protein phosphatase 10
NM_001162408	Dusp13	muscle-restricted dual specificity phosphatase
NM_001007006	Dusp13	testis and skeletal muscle-specific dual
NM_001172056	Dvl2	dishevelled 2
NM_019226	Dync1h1	cytoplasmic dynein 1 heavy chain 1
NM_145772	Dync1li1	cytoplasmic dynein 1 light intermediate chain 1
NM_080697	Dynll2	dynein light chain 2, cytoplasmic
NM_001108506	Ebf3	transcription factor COE3
NM_001191076	Ebf4	transcription factor COE4
NM_001127541	Efcab4a	EF-hand calcium-binding domain-containing
NM_053903	Efna5	ephrin-A5 precursor
NM_053633	Egr2	early growth response protein 2
NM_019137	Egr4	early growth response protein 4
NM_001008773	Eif1a	eukaryotic translation initiation factor 1A

NM_001106867	Eif1b	eukaryotic translation initiation factor 1b
NM_001009391	Enoph1	enolase-phosphatase E1
NM_138541	Epcam	epithelial cell adhesion molecule precursor
NM_001105994	Ephx4	epoxide hydrolase 4
NM_001108343	Etnk2	ethanolamine kinase 2
NM_012555	Ets1	protein C-ets-1
NM_001109323	F8a1	factor VIII intron 22 protein
NM_080895	Faim	fas apoptotic inhibitory molecule 1
NM_080895	Faim	fas apoptotic inhibitory molecule 1
NM_001109885	Fam129b	niban-like protein 1
NM_001012238	Fam20c	dentin matrix protein 4
NM_001014178	Fam69b	hypothetical protein LOC362090
NM_001106566	Fam73b	hypothetical protein LOC296623
NM_001108233	Farp2	FERM, RhoGEF and pleckstrin domain-containing
NM_022272	Fbxl20	F-box/LRR-repeat protein 20
NM_001107203	Fbxo28	F-box only protein 28
NM_001011998	Fbxo9	F-box only protein 9
NM_001107600	Fbxw4	F-box/WD repeat-containing protein 4
NM_001025730	Fbxw5	F-box/WD repeat-containing protein 5
NM_001109224	Fezf1	fez family zinc finger protein 1
NM_130753	Fgf15	fibroblast growth factor 15
NM_130817	Fgf3	fibroblast growth factor 3
NM_001106484	Fign	fidgetin
NM_001108955	Fjx1	four-jointed box protein 1
NM_001134599	Flna	filamin-A
NM_012742	Foxa1	hepatocyte nuclear factor 3-alpha
NM_001013248	Foxb1	forkhead box B1
NM_001191846	Foxo1	forkhead box protein O1
NM_024366	Freq	neuronal calcium sensor 1
NM_001039337	Fubp3	far upstream element-binding protein 3

NM_001025738	Fusip1	FUS interacting protein (serine-arginine rich)
NM_024370	Gabrg3	gamma-aminobutyric acid receptor subunit gamma-3
NM_012563	Gad2	glutamate decarboxylase 2
NM_001005888	Galc	galactocerebrosidase
NM_001025053	Galnt4	polypeptide N-acetylgalactosaminyltransferase 4
NM_022926	Galnt7	N-acetylgalactosaminyltransferase 7
NM_012958	Galr1	galanin receptor type 1
NM_019172	Galr2	galanin receptor type 2
NM_053708	Gbx2	gastrulation brain homeobox 2
NM_019216	Gdf15	growth/differentiation factor 15 precursor
NM_017088	Gdi1	rab GDP dissociation inhibitor alpha
NM_017276	Gdi2	rab GDP dissociation inhibitor beta
NM_001100519	Gga2	golgi associated, gamma adaptin ear containing,
NM_012960	Ggh	gamma-glutamyl hydrolase precursor
NM_001004273	Ggnbp2	gametogenetin-binding protein 2
NM_001037210	Gipc2	PDZ domain-containing protein GIPC2
NM_001004099	Gjb2	gap junction beta-2 protein
NM_019240	Gjb3	gap junction beta-3 protein
NM_001107308	Gmip	GEM-interacting protein
NM_001191836	Gnal	guanine nucleotide-binding protein G(olf)
NM_053765	Gne	bifunctional UDP-N-acetylglucosamine
NM_001007720	Gorasp2	Golgi reassembly-stacking protein 2
NM_012774	Gpc3	glypican-3 precursor
NM_001014108	Gpc4	glypican-4
NM_001034855	Gpr153	probable G-protein coupled receptor 153
NM_001108646	Gpr162	probable G-protein coupled receptor 162
NM_001191915	Gpr50	melatonin-related receptor
NM_001012057	Gpt2	alanine aminotransferase 2
NM_019282	Grem1	gremlin-1 precursor
NM_012575	Grin2c	glutamate [NMDA] receptor subunit epsilon-3

NM_001191873	Gsc	homeobox protein goosecoid
NM_032080	Gsk3b	glycogen synthase kinase-3 beta
NM_001003978	Gspt1	eukaryotic peptide chain release factor
NM_001001512	Gtf2i	general transcription factor II-I
NM_023956	Gucy1a2	guanylate cyclase soluble subunit alpha-2
NM_012578	H1f0	histone H1.0
NM_022674	H2afz	histone H2A.Z
NM_013064	Hcrtr1	orexin receptor type 1
NM_053447	Hdac2	histone deacetylase 2
NM_001108631	Herc3	probable E3 ubiquitin-protein ligase HERC3
NM_001012074	Herc4	probable E3 ubiquitin-protein ligase HERC4
NM_019236	Hes2	transcription factor HES-2
NM_022528	Hif3a	hypoxia-inducible factor 3-alpha
NM_001100986	Hipk1	homeodomain-interacting protein kinase 1
NM_031787	Hipk3	homeodomain-interacting protein kinase 3
NM_017268	Hmgcs1	hydroxymethylglutaryl-CoA synthase, cytoplasmic
NM_031330	Hnrnpab	heterogeneous nuclear ribonucleoprotein A/B
NM_001033696	Hnrpdl	heterogeneous nuclear ribonucleoprotein D-like
NM_013075	Hoxa1	homeobox protein Hox-A1
NM_017112	Hpn	serine protease hepsin
NM_001106392	Hs3st5	heparan sulfate glucosamine 3-O-sulfotransferase
NM_001107778	Hspa12b	heat shock 70 kDa protein 12B
NM_053612	Hspb8	heat shock protein beta-8
NM_022938	Htr7	5-hydroxytryptamine receptor 7
NM_031721	Htra1	serine protease HTRA1
NM_013159	Ide	insulin-degrading enzyme
NM_017183	Il8rb	C-X-C chemokine receptor type 2
NM_133409	Ilk	integrin-linked protein kinase
NM_172224	Impa2	inositol monophosphatase 2
NM_001106083	Ing2	inhibitor of growth protein 2

NM_134417	Ipmk	inositol polyphosphate multikinase
NM_001025422	Irak2	interleukin-1 receptor-associated kinase-like 2
NM_032074	Irs3	insulin receptor substrate 3
NM_181626	Isca1	iron-sulfur cluster assembly 1 homolog,
NM_001014242	Isoc1	isochorismatase domain-containing protein 1
NM_001013880	Isyna1	inositol-3-phosphate synthase 1
NM_001014116	Jmjd8	jmjC domain-containing protein 8
NM_001106630	Jph1	junctophilin-1
NM_001107437	Jph3	junctophilin-3
NM_138875	Jund	transcription factor jun-D
NM_031047	Jup	junction plakoglobin
NM_019270	Kcna3	potassium voltage-gated channel subfamily A
NM_031739	Kcnd3	potassium voltage-gated channel subfamily D
NM_001101003	Kcne1l	potassium voltage-gated channel subfamily E
NM_031358	Kcnj11	ATP-sensitive inward rectifier potassium channel
NM_023021	Kcnn4	intermediate conductance calcium-activated
NM_031597	Kcnq3	potassium voltage-gated channel subfamily KQT
NM_001108515	Kdm2a	lysine-specific demethylase 2A
NM_001109079	Kif26b	kinesin family member 26B
NM_057202	Kif5b	kinesin-1 heavy chain
NM_001048215	Kirrel3	kin of IRRE-like protein 3
NM_023992	Kiss1r	kiSS-1 receptor
NM_022264	Kit	mast/stem cell growth factor receptor
NM_001037354	Klf11	Krueppel-like factor 11
NM_057211	Klf9	Krueppel-like factor 9
NM_001106054	Klhl1	kelch-like protein 1
NM_001106735	Klhl28	kelch-like protein 28
NM_001106252	Klk11	kallikrein-11
NM_017063	Kpnb1	importin subunit beta-1
NM_017068	Lamp2	lysosome-associated membrane glycoprotein 2

NM_001108439	Large	glycosyltransferase-like protein LARGE1
NM_133393	Lfng	beta-1,3-N-acetylglucosaminyltransferase lunatic
NM_173328	Lgr4	leucine-rich repeat-containing G-protein coupled
NM_031713	Lilrb3	leukocyte immunoglobulin-like receptor,
NM_001100722	Lingo1	leucine rich repeat and Ig domain containing 1
NM_053905	Lmnb1	lamin-B1
NM_001103356	LOC100125364	hypothetical protein LOC100125364 precursor
NM_001143803	LOC100233213	hypothetical protein LOC100233213
NM_001177829	LOC100365935	hypothetical protein LOC100365935
NM_001013979	LOC304131	TAK1-like protein
NM_001014007	LOC306766	hypothetical protein LOC306766
NM_001014115	LOC360479	hypothetical protein LOC360479
NM_001037205	LOC360997	similar to ATP-binding cassette, sub-family G
NM_001162931	LOC502128	POM121 membrane glycoprotein-like 2 isoform 2
NM_001162930	LOC502128	POM121 membrane glycoprotein-like 2 isoform 1
NM_001195277	LOC679651	transmembrane protein 178-like
NM_001109418	LOC680531	hypothetical protein LOC680531
NM_001109595	LOC690478	hypothetical protein LOC690478
NM_001109616	LOC691024	hypothetical protein LOC691024
NM_001109627	LOC691153	hypothetical protein LOC691153
NM_030827	Lrp2	low-density lipoprotein receptor-related protein
NM_001008519	Lrpprc	leucine-rich PPR motif-containing protein,
NM_017242	Lsamp	limbic system-associated membrane protein
NM_001106594	Mad2l1	mitotic spindle assembly checkpoint protein
NM_139084	Magi3	membrane-associated guanylate kinase, WW and PDZ
NM_001134971	Man2b2	mannosidase, alpha, class 2B, member 2
NM_031643	Map2k1	dual specificity mitogen-activated protein
NM_138503	Map3k2	mitogen-activated protein kinase kinase kinase
NM_017212	Mapt	microtubule-associated protein tau
NM_001107590	Marveld1	MARVEL domain-containing protein 1

NM_001109132	Marveld3	MARVEL domain-containing protein 3
NM_181089	MAST1	microtubule-associated serine/threonine-protein
NM_021859	Matk	megakaryocyte-associated tyrosine-protein
NM_001108013	Matn3	matrilin-3
NM_001108934	Mblac2	metallo-beta-lactamase domain-containing protein
NM_001025289	Mbp	Golli-Mbp isoform 1
NM_001107618	Mdga1	MAM domain-containing
NM_001191727	Med14	mediator of RNA polymerase II transcription
NM_030860	Mef2d	myocyte-specific enhancer factor 2D
NM_022943	Mertk	tyrosine-protein kinase Mer precursor
NM_001013149	Mesdc1	mesoderm development candidate 1
NM_001191626	Mex3b	RNA-binding protein MEX3B
NM_001024267	MGC109340	signal peptidase complex subunit 3
NM_001024890	MGC114520	hypothetical protein LOC315915
NM_001044292	MGC116202	hypothetical protein LOC688736
NM_001007746	MGC94199	hypothetical protein LOC362483
NM_001191889	Mid2	midline-2
NM_001108737	Mier2	mesoderm induction early response protein 2
NR_031878	Mir132	
NR_031897	Mir181c	
NR_032266	Mir181d	
NR_031909	Mir193	
NR_031925	Mir212	
NR_031850	Mir34a	
NR_031848	Mir34b	
NR_031849	Mir34c	
NR_037325	Mir3549	
NM_001044267	Mknk1	MAP kinase-interacting serine/threonine-protein
NM_001011985	Mknk2	MAP kinase-interacting serine/threonine-protein
NM_001108425	Mocos	molybdenum cofactor sulfurase

NM_001034022	Mprip	myosin phosphatase Rho-interacting protein
NM_022529	Mrpl23	39S ribosomal protein L23, mitochondrial
NM_001108635	Mrpl53	39S ribosomal protein L53, mitochondrial
NM_001106628	Mrps35	28S ribosomal protein S35, mitochondrial
NM_053712	Msx3	homeo box, msh-like 3
NM_001100833	Mtch1	mitochondrial carrier homolog 1
NM_001006960	Mtp18	mitochondrial 18 kDa protein
NM_001100667	Mtx1	metaxin 1
NM_145773	Mxd3	max dimerization protein 3
NM_001106257	Mybpc2	myosin-binding protein C, fast-type
NM_001107344	Mylip	E3 ubiquitin-protein ligase MYLIP
NM_001109678	Nadk	NAD kinase
NM_001107674	Narg1	N-alpha-acetyltransferase 15, NatA auxiliary
NM_001014785	Ncbp1	nuclear cap-binding protein subunit 1
NM_001013059	Ndfip1	NEDD4 family-interacting protein 1
NM_012607	Nefh	neurofilament heavy polypeptide
NM_017029	Nefm	neurofilament medium polypeptide
NM_053691	Nek2	NIMA-related kinase 2
NM_001013134	Nek4	serine/threonine-protein kinase Nek4
NM_001002851	Nenf	neudesin precursor
NM_012987	Nes	nestin
NM_031789	Nfe2l2	nuclear factor erythroid 2-related factor 2
NM_012865	Nfya	nuclear transcription factor Y subunit alpha
NM_012610	Ngfr	tumor necrosis factor receptor superfamily
NM_001191733	Nhs	Nance-Horan syndrome protein
NM_013093	Nkx2-1	homeobox protein Nkx-2.1
NM_134336	Nlgn3	neuroligin-3 precursor
NM_001024360	nod3l	hypothetical protein LOC501101
NM_001012356	Nono	non-POU domain-containing octamer-binding
NM_001105721	Notch1	neurogenic locus notch homolog protein 1

NM_001007800	N-pac	putative oxidoreductase GLYR1
NM_153293	Npb	neuropeptide B precursor
NM_001004231	Npdc1	neural proliferation differentiation and control
NM_019380	Nptn	neuroplastin
NM_024388	Nr4a1	nuclear receptor subfamily 4 group A member 1
NM_031628	Nr4a3	nuclear receptor subfamily 4 group A member 3
NM_001100708	Nrf1	nuclear respiratory factor 1
NM_001107337	Nsd1	histone-lysine N-methyltransferase, H3 lysine-36
NM_053731	Ntn1	netrin-1 precursor
NM_001106465	Ntng1	netrin-G1
NM_001011891	Nubp2	cytosolic Fe-S cluster assembly factor NUBP2
NM_181363	Nudt6	nucleoside diphosphate-linked moiety X motif 6
NM_021680	Nxph4	neurexophilin-4 precursor
NM_001025708	Ogfrl1	opioid growth factor receptor-like protein 1
NM_001107848	Ophn1	oligophrenin-1
NM_001014024	Orai3	protein orai-3
NM_001107565	Oraov1	oral cancer overexpressed 1
NM_001013079	Osbpl2	oxysterol-binding protein-related protein 2
NM_001191700	Otud4	OTU domain-containing protein 4
NM_001037496	Otud5	OTU domain-containing protein 5
NM_012721	P2rx6	P2X purinoceptor 6
NM_134353	Pabpc1	polyadenylate-binding protein 1
NM_001009966	Pacsin3	protein kinase C and casein kinase substrate in
NM_017230	Padi3	protein-arginine deiminase type-3
NM_001108937	Paip1	polyadenylate-binding protein-interacting
NM_133531	Pank4	pantothenate kinase 4
NM_199409	Panx2	pannexin-2
NM_001191077	Paqr6	progestin and adipoQ receptor family member 6
NM_001035249	Parl	presenilins-associated rhomboid-like protein,
NM_033485	Pawr	PRKC apoptosis WT1 regulator protein

NM_001169129	Pcdh19	protocadherin-19
NM_001129882	Pcgf5	polycomb group RING finger protein 5
NM_001009542	Pdcd10	programmed cell death protein 10
NM_031317	Pdgfc	platelet-derived growth factor C
NM_001004072	Pdha1	pyruvate dehydrogenase E1 component subunit
NM_053826	Pdk1	[Pyruvate dehydrogenase [lipoamide]] kinase
NM_001013231	Pea15a	astrocytic phosphoprotein PEA-15
NM_030873	Pfn2	profilin-2
NM_001106198	Pgbd5	piggyBac transposable element-derived protein 5
NM_001106577	Phtf2	putative homeodomain transcription factor 2
NM_031083	Pi4kb	phosphatidylinositol 4-kinase beta
NM_031784	Pias3	E3 SUMO-protein ligase PIAS3
NM_001105951	Pik3c2b	phosphatidylinositol-4-phosphate 3-kinase C2
NM_017034	Pim1	proto-oncogene serine/threonine-protein kinase
NM_022602	Pim3	serine/threonine-protein kinase pim-3
NM_001008369	Pitpnm1	membrane-associated phosphatidylinositol
NM_053624	Pitx1	pituitary homeobox 1
NM_019334	Pitx2	pituitary homeobox 2 isoform 2
NM_001042505	Pitx2	pituitary homeobox 2 isoform 1
NM_017175	Pkn1	serine/threonine-protein kinase N1
NM_001105845	Plcd3	1-phosphatidylinositol-4,5-bisphosphate
NM_053758	Plce1	1-phosphatidylinositol-4,5-bisphosphate
NM_001134972	Plekhg2	pleckstrin homology domain-containing family G
NM_001108036	Plekhh1	pleckstrin homology domain containing, family H
NM_001134637	Plin5	perilipin-5
NM_001142915	Plod2	procollagen-lysine,2-oxoglutarate 5-dioxygenase
NM_001107922	Pm20d2	peptidase M20 domain-containing protein 2
NM_001107272	Pnma2	paraneoplastic antigen MA2
NM_001109468	Polr3g	DNA-directed RNA polymerase III subunit RPC7
NM_172085	Pou3f2	POU domain, class 3, transcription factor 2

NM_022538	Ppap2a	lipid phosphate phosphohydrolase 1
NM_013196	Ppara	peroxisome proliferator-activated receptor
NM_001145367	Pparg	peroxisome proliferator-activated receptor gamma
NM_001145366	Pparg	peroxisome proliferator-activated receptor gamma
NM_176075	Ppargc1b	peroxisome proliferator-activated receptor gamma
NM_198773	Ppm1e	protein phosphatase 1E
NM_001191072	Ppp1r16b	protein phosphatase 1 regulatory inhibitor
NM_144746	Ppp2r2d	serine/threonine-protein phosphatase 2A 55 kDa
NM_001108577	Ppp2r4	serine/threonine-protein phosphatase 2A
NM_181379	Ppp2r5b	serine/threonine-protein phosphatase 2A 56 kDa
NM_001106740	Ppp2r5e	serine/threonine-protein phosphatase 2A 56 kDa
NM_017309	Ppp3r1	calcineurin subunit B type 1
NM_001106613	Ppp4r2	protein phosphatase 4, regulatory subunit 2
NM_001013957	Pqbp1	polyglutamine-binding protein 1
NM_134449	Prkcdbp	protein kinase C delta-binding protein
NM_001033963	Prkx	serine/threonine-protein kinase PRKX
NM_001038588	Prodh2	probable proline dehydrogenase 2
NM_021751	Prom1	prominin 1 isoform 1
NM_001012121	Prr5	proline-rich protein 5
NM_001109116	Prr7	proline-rich protein 7
NM_001109226	Prrt4	proline-rich transmembrane protein 4
NM_001109027	Prss33	serine protease 33
NM_019126	Psg19	carcinoembryonic antigen gene family (CGM3)
NM_130430	Psmd9	26S proteasome non-ATPase regulatory subunit 9
NM_001106138	Psmg2	tumor necrosis factor superfamily, member
NM_022516	Ptbp1	polypyrimidine tract-binding protein 1 isoform
NM_053566	Ptch1	protein patched homolog 1
NM_020073	Pth1r	parathyroid hormone/parathyroid hormone-related
NM_031579	Ptp4a1	protein tyrosine phosphatase type IVA 1
NM_013088	Ptpn11	tyrosine-protein phosphatase non-receptor type

NM_019253	Ptpn5	tyrosine-protein phosphatase non-receptor type
NM_012763	Ptpra	receptor-type tyrosine-protein phosphatase alpha
NM_001108684	Pum1	pumilio homolog 1
NM_001106715	Pum2	pumilio homolog 2
NM_001108507	Pwwp2b	PWWP domain-containing protein 2B
NM_031152	Rab11a	ras-related protein Rab-11A
NM_001109005	Rab23	ras-related protein Rab-23
NM_031718	Rab2a	ras-related protein Rab-2A
NM_053741	Rap2a	RAS related protein 2a
NM_001170531	Rasgrf1	ras-specific guanine nucleotide-releasing factor
NM_001105753	Rasgrf1	ras-specific guanine nucleotide-releasing factor
NM_001106261	Rasip1	ras-interacting protein 1
NM_001108862	Rasl10a	ras-like protein family member 10A
NM_001106317	Rassf7	ras association domain-containing protein 7
NM_031816	Rbbp7	histone-binding protein RBBP7
NM_001198584	RGD1303117	hypothetical protein LOC292764
NM_001004268	RGD1303271	hypothetical protein LOC313018
NM_001108652	RGD1306151	hypothetical protein LOC362455
NM_001107663	RGD1307225	hypothetical protein LOC310269
NM_001108308	RGD1307394	hypothetical protein LOC360667
NM_001017454	RGD1307799	IST1 homolog
NM_001134596	RGD1308299	hypothetical protein LOC367214
NM_001108129	RGD1309188	hypothetical protein LOC315463
NM_001079705	RGD1311558	shootin-1
NM_001108286	RGD1311564	hypothetical protein LOC360590
NM_001127526	RGD1311605	hypothetical protein LOC298841
NM_001108678	RGD1559909	hypothetical protein LOC362592
NM_001106014	RGD1560394	hypothetical protein LOC289728
NM_001109345	RGD1563349	hypothetical protein LOC502727
NM_001109311	RGD1563692	hypothetical protein LOC501185

NM_001109292	RGD1564560	hypothetical protein LOC500988
NM_001030034	Rhbdf1	rhomboid family member 1
NM_001191665	Rilpl1	RILP-like protein 1
NM_053945	Rims2	regulating synaptic membrane exocytosis protein
NM_145881	Rims2	regulating synaptic membrane exocytosis protein
NM_001108052	Rin3	ras and Rab interactor 3
NM_001007641	Rnd3	rho-related GTP-binding protein RhoE precursor
NM_001106836	Rnf111	E3 ubiquitin-protein ligase Arkadia
NM_001173349	Rnf128	E3 ubiquitin-protein ligase RNF128
NM_001191093	Rnf150	RING finger protein 150
NM_001107118	Rnf6	RING finger protein 6
NM_001012124	Rpp25	ribonuclease P protein subunit p25
NM_001048184	Rragc	ras-related GTP-binding protein C
NM_001106641	Rragd	ras-related GTP-binding protein D
NM_001008346	Rrp8	ribosomal RNA-processing protein 8
NM_001107980	Rspo1	R-spondin-1
NM_001008845	RT1-CE7	RT1 class I, locus CE7
NM_053613	Rtn4r	reticulon-4 receptor precursor
NM_181377	Rtn4rl1	reticulon-4 receptor-like 1 precursor
NM_181380	Rtn4rl2	reticulon-4 receptor-like 2 precursor
NM_001105870	Rtp1	receptor-transporting protein 1
NM_022394	Safb	scaffold attachment factor B1
NM_001097581	Sav1	protein salvador homolog 1
NM_001013985	Sccpdh	probable saccharopine dehydrogenase
NM_001008880	Scn4b	sodium channel subunit beta-4 precursor
NM_177929	Sdccag8	serologically defined colon cancer antigen 8
NM_001107637	Sec63	translocation protein SEC63 homolog
NM_001014253	Selt	selenoprotein T precursor
NM_001107091	Sema5b	sema domain, seven thrombospondin repeats (type
NM_022616	Sept7	septin-7 isoform a

NM_001109104	Serp2	stress-associated endoplasmic reticulum protein
NM_053779	Serpini1	neuroserpin precursor
NM_001007735	Sertad1	SERTA domain-containing protein 1
NM_031647	Sfmbt1	scm-like with four MBT domains protein 1
NM_001009720	Sfrs2	serine/arginine-rich splicing factor 2
NM_001105937	Sgsm1	small G protein signaling modulator 1
NM_001137647	Sh3bgrl2	SH3 domain-binding glutamic acid-rich-like
NM_053360	Sh3kbp1	SH3 domain-containing kinase-binding protein 1
NM_001191936	Shisa2	protein shisa-2 homolog
NM_001191922	Shisa6	protein shisa-6 homolog
NM_134457	Siah2	E3 ubiquitin-protein ligase SIAH2
NM_021693	Sik1	serine/threonine-protein kinase SIK1
NM_031798	Slc12a2	solute carrier family 12 member 2
NM_134363	Slc12a5	solute carrier family 12 member 5
NM_001013144	Slc12a7	solute carrier family 12 member 7
NM_153625	Slc12a8	solute carrier family 12 member 8
NM_012716	Slc16a1	monocarboxylate transporter 1
NM_147216	Slc16a2	monocarboxylate transporter 8
NM_032065	Slc1a6	excitatory amino acid transporter 4
NM_177421	Slc22a17	solute carrier family 22 member 17
NM_001106327	Slc22a20	solute carrier family 22 member 20
NM_017316	Slc23a2	solute carrier family 23 member 2
NM_001108051	Slc24a4	sodium/potassium/calcium exchanger 4
NM_001013996	Slc25a37	mitoferrin-1
NM_001100515	Slc25a46	solute carrier family 25 member 46
NM_001105985	Slc30a10	zinc transporter 10
NM_133600	Slc31a1	high affinity copper uptake protein 1
NM_001134687	Slc35e3	solute carrier family 35 member E3
NM_001105950	Slc35f5	solute carrier family 35 member F5
NM_001011952	Slc39a8	zinc transporter ZIP8

NM_001191920	Slc47a2	multidrug and toxin extrusion protein 2
NM_053424	Slc4a4	electrogenic sodium bicarbonate cotransporter 1
NM_130746	Slc5a6	sodium-dependent multivitamin transporter
NM_017206	Slc6a6	sodium- and chloride-dependent taurine
NM_001113335	Slc9a2	sodium/hydrogen exchanger 2 isoform 1
NM_022667	Slco2a1	solute carrier organic anion transporter family
NM_013095	Smad3	mothers against decapentaplegic homolog 3
NM_030858	Smad7	mothers against decapentaplegic homolog 7
NM_001107419	Smarca5	SWI/SNF-related matrix-associated
NM_001108752	Smarcd1	SWI/SNF-related matrix-associated
NM_206851	Smyd2	SET and MYND domain-containing protein 2
NM_001106832	Snx22	sorting nexin-22
NM_001191563	Sorcs1	VPS10 domain-containing receptor SorCS1
NM_001106367	Sorcs3	VPS10 domain-containing receptor SorCS3
NM_019193	Sox10	transcription factor SOX-10
NM_001106850	Sox14	SRY (sex determining region Y)-box 14
NM_031792	Spag4	sperm-associated antigen 4 protein
NM_001106530	Spag4l	SUN domain-containing protein 5
NM_001108549	Spata5	spermatogenesis-associated protein 5
NM_181388	Spg7	paraplegin
NM_001039208	Spns1	protein spinster homolog 1
NM_172067	Spon1	spondin-1 precursor
NM_001106988	Spsb3	SPRY domain-containing SOCS box protein 3
NM_175843	Sqstm1	sequestosome-1 isoform 1
NM_181550	Sqstm1	sequestosome-1 isoform 2
NM_001135711	Srrp	35 kDa SR repressor protein
NM_012747	Stat3	signal transducer and activator of transcription
NM_031704	Stx5	syntaxin-5
NM_031665	Stx6	syntaxin-6
NM_001100750	Suclg2	succinyl-CoA ligase [GDP-forming] subunit beta,

NM_001025125	Sumf2	sulfatase-modifying factor 2
NM_001108883	Suv39h2	histone-lysine N-methyltransferase SUV39H2
NM_019133	Syn1	synapsin-1 isoform a
NM_001004107	Tacc1	transforming, acidic coiled-coil containing
NM_001170455	Tada2b	transcriptional adaptor 2B
NM_001025734	Tada3l	transcriptional adapter 3
NM_001013127	Tagln2	transgelin-2
NM_001025419	Tax1bp3	tax1-binding protein 3
NM_001015022	Tbc1d10a	TBC1 domain family member 10A
NM_181638	Tbx3	T-box transcription factor TBX3
NM_001130077	Tcerg1l	transcription elongation regulator 1-like
NM_201420	Tcfap2c	transcription factor AP-2 gamma
NM_001098216	Tead3	TEA domain family member 3
NM_019194	Tef	thyrotroph embryonic factor
NM_201655	Tepp	testis, prostate and placenta-expressed protein
NM_031131	Tgfb2	transforming growth factor beta-2 precursor
NM_031132	Tgfbr2	TGF-beta receptor type-2 precursor
NM_001100558	Tiam1	T-cell lymphoma invasion and metastasis 1
NM_001108249	Timm17b	translocase of inner mitochondrial membrane 17
NM_001172125	Tlx2	T-cell leukemia, homeobox 2
NM_001107015	Tm4sf5	transmembrane 4 L6 family member 5
NM_001105758	Tmed7	transmembrane emp24 domain-containing protein 7
NM_001134410	Tmem132e	transmembrane protein 132E
NM_001008774	Tmem170b	transmembrane protein 170B
NM_001135712	Tmem185a	transmembrane protein 185A
NM_001191668	Tmem185b	transmembrane protein 185B
NM_001109480	Tmem229a	transmembrane protein 229A
NM_001191610	Tmem60	transmembrane protein 60
NM_001017455	Tmem80	transmembrane protein 80
NM_001109485	Tmem90a	capucin

NM_001105806	Tmem93	transmembrane protein 93
NM_153311	Tmprss5	transmembrane protease serine 5
NR_024118	Tnxa	
NM_001105818	Tp53i13	tumor protein p53-inducible protein 13
NM_001044295	Tpd52l1	tumor protein D52-like 1
NM_001106788	Trabd	traB domain-containing protein
NM_053916	Trim28	transcription intermediary factor 1-beta
NM_031786	Trim3	tripartite motif-containing protein 3
NM_001106453	Trim45	tripartite motif-containing protein 45
NM_130420	Trim9	E3 ubiquitin-protein ligase TRIM9
NM_001109912	Tsc22d1	TSC22 domain family protein 1 isoform 1
NM_022589	Tspan2	tetraspanin-2
NM_012808	Tst	thiosulfate sulfurtransferase
NM_001108663	Tstd2	thiosulfate sulfurtransferase/rhodanese-like
NM_001025675	Tubb6	tubulin, beta 6
NM_001039163	Tusc5	tumor suppressor candidate 5 homolog
NM_001037643	Ube2z	ubiquitin-conjugating enzyme E2 Z
NM_001105723	Ubtf	nucleolar transcription factor 1 isoform 1
NM_019354	Ucp2	mitochondrial uncoupling protein 2
NM_001003709	Ufc1	ubiquitin-fold modifier-conjugating enzyme 1
NM_001077660	Urg4	up-regulated gene 4
NM_145184	Usp15	ubiquitin carboxyl-terminal hydrolase 15
NM_001106120	Usp6nl	USP6 N-terminal-like protein
NM_022637	Vax2	ventral anterior homeobox 2
NM_001107248	Vcl	vinculin
NM_053653	Vegfc	vascular endothelial growth factor C precursor
NM_203338	Vkorc1l1	vitamin K epoxide reductase complex subunit
NM_013155	Vldlr	very low-density lipoprotein receptor precursor
NM_001106092	Vps36	vacuolar protein-sorting-associated protein 36
NM_001108479	Vstm2b	V-set and transmembrane domain-containing

NM_001169128	Vsx2	visual system homeobox 2
NM_001135894	Wdr25l	WD repeat domain 25-like
NM_001037791	Wdr43	WD repeat domain 43
NM_001110489	Wdr86	WD repeat-containing protein 86
NM_001191556	Wnk2	serine/threonine-protein kinase WNK2
NM_001108227	Wnt10a	protein Wnt-10a
NM_001108226	Wnt6	protein Wnt-6
NM_001105783	Wnt9a	protein Wnt-9a
NM_001106184	Wwp2	NEDD4-like E3 ubiquitin-protein ligase WWP2
NM_022231	Xiap	baculoviral IAP repeat-containing protein 4
NM_001105992	Xpr1	xenotropic and polytropic retrovirus receptor 1
NM_022296	Xylt2	xylosyltransferase 2
NM_199383	Yipf1	protein YIPF1
NM_001014208	Yipf2	protein YIPF2
NM_001025747	Yipf6	protein YIPF6
NM_175604	Yrdc	yrdC domain-containing protein, mitochondrial
NM_019377	Ywhab	14-3-3 protein beta/alpha
NM_019376	Ywhag	14-3-3 protein gamma
NM_001106129	Zadh2	zinc-binding alcohol dehydrogenase
NM_001107097	Zbtb11	zinc finger and BTB domain-containing protein
NM_001130537	Zbtb39	zinc finger and BTB domain-containing protein
NM_001170577	Zfp167	zinc finger protein 167
NM_001034831	Zfp384	zinc finger protein 384 isoform 2
NM_133429	Zfp384	zinc finger protein 384 isoform 1
NM_001034830	Zfp384	zinc finger protein 384 isoform 1
NM_001012093	Zfp64	zinc finger protein 64
NM_001109225	Zfp800	zinc finger protein 800
NM_001109017	Zfx	zinc finger X-chromosomal protein
NM_001108725	Zfyve21	zinc finger FYVE domain-containing protein 21
NM_203369	Zmynd11	zinc finger MYND domain-containing protein 11

NM_001024878	Znrf4	zinc/RING finger protein 4
NM_031616	Zranb2	zinc finger Ran-binding domain-containing
NM_053761	Zyx	zyxin
