# Supplementary material for: Interplay of miR-137 and EZH2 contributes to the genome-wide redistribution of H3K27me3 underlying the Pb-induced memory impairment
Source: Cell Death Dis. 2019 Sep 11;10(9):671. doi: 10.1038/s41419-019-1912-7 (PMC6739382; doi:10.1038/s41419-019-1912-7)
Supplement: Supplementary file 12 — Dataset 4 [file 41419_2019_1912_MOESM12_ESM.pdf]

List of genes differentially regulated by H3K27me3 upon Pb exposure

Accession	Symbol	Gene Name
NM_012690	Abcb4	multidrug resistance protein 2
NM_080582	Abcb6	ATP-binding cassette sub-family B member 6,
NM_001005902	Abtb1	ankyrin repeat and BTB/POZ domain-containing
NM_001126079	Acbd7	acyl-CoA-binding domain-containing protein 7
NM_001106508	Acoxl	acyl-coenzyme A oxidase-like protein
NM_001134956	Ahdc1	A.T hook DNA-binding motif-containing protein 1
NM_030986	Ak2	adenylate kinase 2, mitochondrial isoform a
NM_001033967	Ak2	adenylate kinase 2, mitochondrial isoform b
NM_053896	Aldh1a2	retinal dehydrogenase 2
NM_153301	Alox15b	arachidonate 15-lipoxygenase B
NM_013059	Alpl	alkaline phosphatase, tissue-nonspecific isozyme
NM_001134699	Ankrd40	ankyrin repeat domain-containing protein 40
NM_001009676	Anks3	ankyrin repeat and SAM domain-containing protein
NM_001008523	Aox4	aldehyde oxidase 4
NM_001108331	Ap1s1	adaptor protein complex AP-1, sigma 1
NM_012500	Apeh	acylamino-acid-releasing enzyme
NM_001004242	Arhgap8	rho GTPase-activating protein 8
NM_001080789	Arhgap9	rho GTPase-activating protein 9 isoform 1
NM_001012198	Arhgap9	rho GTPase-activating protein 9 isoform 2
NM_001013108	Arih1	ariadne ubiquitin-conjugating enzyme E2 binding
NM_001106919	Arpc2	actin-related protein 2/3 complex subunit 2
NM_001037767	Arpc5l	actin-related protein 2/3 complex subunit 5-like
NM_198735	Art2b	ADP-ribosyltransferase 2b
NM_053397	Artn	artemin precursor
NM_012914	Atp2a3	sarcoplasmic/endoplasmic reticulum calcium
NM_001025767	Blnk	B-cell linker protein
NM_001128187	Bnipl	BCL2/adenovirus E1B 19kD interacting protein
NM_017259	Btg2	protein BTG2

NM_001166344	Btnl4	butyrophilin subfamily 3 member A2
NM_212489	Btnl8	butyrophilin-like 8
NM_001107404	Cables1	CDK5 and ABL1 enzyme substrate 1
NM_080694	Cacng6	voltage-dependent calcium channel gamma-6
NM_138513	Calcb	calcitonin gene-related peptide 2 precursor
NM_012518	Calm3	calmodulin
NM_019174	Car4	carbonic anhydrase 4 precursor
NM_001130554	Card10	caspase recruitment domain-containing protein
NM_001013191	Cbfb	core-binding factor subunit beta
NM_199117	Cbx7	chromobox protein homolog 7
NM_001105725	Ccng2	cyclin-G2
NM_001166577	Cd300e	CMRF35-like molecule 2
NM_017124	Cd37	leukocyte antigen CD37
NM_013169	Cd3d	T-cell surface glycoprotein CD3 delta chain
NM_001077646	Cd3g	T-cell surface glycoprotein CD3 gamma chain
NM_134360	Cd40	tumor necrosis factor receptor superfamily
NM_001015016	Cd72	B-cell differentiation antigen CD72
NM_001013103	Cdc34	ubiquitin-conjugating enzyme Cdc34
NM_053620	Cdc42bpb	serine/threonine-protein kinase MRCK beta
NM_001048044	Cdc42ep3	CDC42 effector protein (Rho GTPase binding) 3
NM_138889	Cdh13	cadherin-13
NM_053891	Cdk5r1	cyclin-dependent kinase 5 activator 1
NM_012831	Cebpg	CCAAT/enhancer-binding protein gamma
NM_001105900	Cggbp1	CGG triplet repeat-binding protein 1
NM_001170593	Chat	choline O-acetyltransferase
NM_017127	Chka	choline kinase alpha
NM_001011955	Chst1	carbohydrate sulfotransferase 1
NM_031702	Cldn7	claudin-7
NM_031818	Clic4	chloride intracellular channel protein 4
NM_022218	Cmklr1	chemokine-like receptor 1

NM_001011942	Cnnm2	metal transporter CNNM2
NM_001014232	Cnrip1	CB1 cannabinoid receptor-interacting protein 1
NM_001107236	Cobl	protein cordon-bleu
NM_001108710	Coch	cochlin
NM_001025721	Colec12	collectin-12
NM_031766	Cpz	carboxypeptidase Z precursor
NM_001105716	Crabp1	cellular retinoic acid-binding protein 1
NM_001004085	Crat	carnitine O-acetyltransferase
NM_001024783	Creld1	cysteine-rich with EGF-like domain protein 1
NM_001014258	Crls1	cardiolipin synthase
NM_017074	Cth	cystathionine gamma-lyase
NM_001100661	Ctr9	Ctr9, Paf1/RNA polymerase II complex component,
NM_013156	Ctsl1	cathepsin L1 preproprotein
NM_022297	Ddah1	N(G),N(G)-dimethylarginine
NM_030993	Ddn	dendrin
NM_001109577	Derl3	derlin-3
NM_032063	Dll1	delta-like protein 1 precursor
NM_001105832	Dlx3	distal-less homeobox 3
NM_012943	Dlx5	homeobox protein DLX-5
NM_053706	Dmrt1	doublesex- and mab-3-related transcription
NM_001107597	Dmrt2	doublesex- and mab-3-related transcription
NM_001105759	Dock9	dedicator of cytokinesis protein 9
NM_012546	Drd1a	D(1A) dopamine receptor
NM_024141	Duox2	dual oxidase 2 precursor
NM_001191965	Duoxa2	dual oxidase maturation factor 2
NM_001037973	Dusp9	dual specificity protein phosphatase 9
NM_012842	Egf	pro-epidermal growth factor precursor
NM_001008773	Eif1a	eukaryotic translation initiation factor 1A
NM_001107602	Elovl3	elongation of very long chain fatty acids
NM_001009391	Enoph1	enolase-phosphatase E1

NM_021687	Erbb4	receptor tyrosine-protein kinase erbB-4
NM_022604	Esm1	endothelial cell-specific molecule 1 precursor
NM_012555	Ets1	protein C-ets-1
NM_133537	Expi	extracellular peptidase inhibitor
NM_001109885	Fam129b	niban-like protein 1
NM_001012238	Fam20c	dentin matrix protein 4
NM_001106296	Fam57b	hypothetical protein LOC293493
NM_001014046	Fam82a2	regulator of microtubule dynamics protein 3
NM_022272	Fbxl20	F-box/LRR-repeat protein 20
NM_001107203	Fbxo28	F-box only protein 28
NM_001011998	Fbxo9	F-box only protein 9
NM_001107600	Fbxw4	F-box/WD repeat-containing protein 4
NM_053843	Fcgr2a	low affinity immunoglobulin gamma Fc region
NM_001100682	Fcrla	Fc receptor-like A precursor
NM_144753	Fev	protein FEV
NM_001109224	Fezf1	fez family zinc finger protein 1
NM_130752	Fgf21	fibroblast growth factor 21
NM_130817	Fgf3	fibroblast growth factor 3
NM_001106484	Fign	fidgetin
NM_001168584	Foxb2	forkhead box B2
NM_024366	Freq	neuronal calcium sensor 1
NM_001025738	Fusip1	FUS interacting protein (serine-arginine rich)
NM_031236	Fut1	galactoside 2-alpha-L-fucosyltransferase 1
NM_022005	Fxyd6	FXYD domain-containing ion transport regulator 6
NM_001039036	Gabpb1	GA repeat binding protein, beta 1
NM_012563	Gad2	glutamate decarboxylase 2
NM_001025053	Galnt4	polypeptide N-acetylgalactosaminyltransferase 4
NM_001122644	Galnt9	polypeptide N-acetylgalactosaminyltransferase 9
NM_133293	Gata3	GATA binding protein 3
NM_144730	Gata4	transcription factor GATA-4

NM_019185	Gata6	transcription factor GATA-6
NM_001004273	Ggnbp2	gametogenetin-binding protein 2
NM_001004099	Gjb2	gap junction beta-2 protein
NM_013133	Glra1	glycine receptor subunit alpha-1
NM_001134413	Gltp	glycolipid transfer protein
NM_053765	Gne	bifunctional UDP-N-acetylglucosamine
NM_001007720	Gorasp2	Golgi reassembly-stacking protein 2
NM_001014108	Gpc4	glypican-4
NM_001034855	Gpr153	probable G-protein coupled receptor 153
NM_017010	Grin1	glutamate [NMDA] receptor subunit zeta-1
NM_001109270	Grrp1	glycine/arginine-rich protein 1
NM_001191873	Gsc	homeobox protein goosecoid
NM_032080	Gsk3b	glycogen synthase kinase-3 beta
NM_001003978	Gspt1	eukaryotic peptide chain release factor
NM_001001512	Gtf2i	general transcription factor II-I
NM_023956	Gucy1a2	guanylate cyclase soluble subunit alpha-2
NM_022674	H2afz	histone H2A.Z
NM_022696	Hand2	heart- and neural crest derivatives-expressed
NM_001012074	Herc4	probable E3 ubiquitin-protein ligase HERC4
NM_001100986	Hipk1	homeodomain-interacting protein kinase 1
NM_031787	Hipk3	homeodomain-interacting protein kinase 3
NM_001106303	Hmx2	homeobox protein HMX2
NM_031330	Hnrnpab	heterogeneous nuclear ribonucleoprotein A/B
NM_001033696	Hnrpdl	heterogeneous nuclear ribonucleoprotein D-like
NM_001191087	Hoxa6	homeobox protein Hox-A6
NM_001109233	Hoxa9	homeobox protein Hox-A7
NM_001107042	Hoxb3	homeo box B3
NM_001100787	Hoxb4	homeo box B4
NM_001191925	Hoxb5	homeo box B5
NM_001017480	Hoxb7	homeobox protein Hox-B7

NM_001100497	Hoxb9	homeo box B9
NM_001106796	Hoxc12	homeobox protein Hox-C12
NM_001105884	Hoxd1	homeobox protein Hox-D1
NM_017122	Hpca	neuron-specific calcium-binding protein
NM_001135762	Hpse2	heparanase-2
NM_181370	Hs3st2	heparan sulfate glucosamine 3-O-sulfotransferase
NM_001106177	Hsf4	heat shock factor protein 4
NM_024395	Htr5b	5-hydroxytryptamine receptor 5B
NM_031721	Htra1	serine protease HTRA1
NM_001107321	Htra4	probable serine protease HTRA4
NM_001082477	Igf1	insulin-like growth factor I isoform a
NM_001107197	Igsf9	protein turtle homolog A precursor
NM_001107237	Ikzf1	IKAROS family zinc finger 1
NM_001107521	Il20ra	interleukin-20 receptor subunit alpha
NM_133409	Ilk	integrin-linked protein kinase
NM_001106083	Ing2	inhibitor of growth protein 2
NM_207617	Iqsec3	IQ motif and SEC7 domain-containing protein 3
NM_001025422	Irak2	interleukin-1 receptor-associated kinase-like 2
NM_181626	Isca1	iron-sulfur cluster assembly 1 homolog,
NM_001014242	Isoc1	isochorismatase domain-containing protein 1
NM_001106630	Jph1	junctophilin-1
NM_001107437	Jph3	junctophilin-3
NM_031047	Jup	junction plakoglobin
NM_001008814	Kb21	keratin, type II cuticular Hb1
NM_019270	Kcna3	potassium voltage-gated channel subfamily A
NM_053630	Kcnh4	potassium voltage-gated channel subfamily H
NM_145095	Kcnh8	potassium voltage-gated channel subfamily H
NM_031358	Kcnj11	ATP-sensitive inward rectifier potassium channel
NM_130813	Kcnk15	potassium channel subfamily K member 15
NM_001039516	Kcnk5	potassium channel subfamily K member 5

NM_031597	Kcnq3	potassium voltage-gated channel subfamily KQT
NM_001109079	Kif26b	kinesin family member 26B
NM_001107164	Klf1	Krueppel-like factor 1
NM_057211	Klf9	Krueppel-like factor 9
NM_001106735	Klhl28	kelch-like protein 28
NM_001109326	Krtap14	keratin-associated protein 14
NM_053538	Laptm5	lysosomal-associated transmembrane protein 5
NM_001007556	Lefty2	left-right determination factor 2
NM_133393	Lfng	beta-1,3-N-acetylglucosaminyltransferase lunatic
NM_139036	Lhx5	LIM/homeobox protein Lhx5
NM_001107837	Lhx6	LIM/homeobox protein Lhx6
NM_001100722	Lingo1	leucine rich repeat and Ig domain containing 1
NM_053905	Lmnb1	lamin-B1
NM_001103356	LOC100125364	hypothetical protein LOC100125364 precursor
NM_001013941	LOC298795	hypothetical protein LOC298795
NM_001014007	LOC306766	hypothetical protein LOC306766
NM_001014115	LOC360479	hypothetical protein LOC360479
NM_001135992	LOC498276	Fc gamma receptor II beta
NM_001109221	LOC500034	hypothetical protein LOC500034
NM_001195277	LOC679651	transmembrane protein 178-like
NM_001109489	LOC685964	hypothetical protein LOC685964
NM_001109627	LOC691153	hypothetical protein LOC691153
NM_001008519	Lrpprc	leucine-rich PPR motif-containing protein,
NM_001170434	Lrrc32	leucine rich repeat containing 32
NM_017242	Lsamp	limbic system-associated membrane protein
NM_021656	Ltb4r	leukotriene B4 receptor 1
NM_001109391	Mab21l2	protein mab-21-like 2
NM_139084	Magi3	membrane-associated guanylate kinase, WW and PDZ
NM_181089	MAST1	microtubule-associated serine/threonine-protein
NM_001039005	Mcoln2	mucolipin-2

NM_001107618	Mdga1	MAM domain-containing
NM_030859	Mdk	midkine precursor
NM_001191727	Med14	mediator of RNA polymerase II transcription
NM_030860	Mef2d	myocyte-specific enhancer factor 2D
NM_001108837	Meox1	homeobox protein MOX-1
NM_017149	Meox2	homeobox protein MOX-2
NM_001107531	Mesp1	mesoderm posterior protein 1
NM_001191626	Mex3b	RNA-binding protein MEX3B
NM_001008518	MGC105649	normal mucosa of esophagus-specific gene 1
NM_001044292	MGC116202	hypothetical protein LOC688736
NM_001007746	MGC94199	hypothetical protein LOC362483
NR_031814	Mir10a	
NR_031883	Mir137	
NR_037325	Mir3549	
NM_001108425	Mocos	molybdenum cofactor sulfurase
NM_020102	Mos	proto-oncogene serine/threonine-protein kinase
NM_001191558	Mtss1l	MTSS1-like protein
NM_001100667	Mtx1	metaxin 1
NM_057209	Mylk2	myosin light chain kinase 2, skeletal/cardiac
NM_053888	Myt1l	myelin transcription factor 1-like protein
NM_001109678	Nadk	NAD kinase
NM_001014785	Ncbp1	nuclear cap-binding protein subunit 1
NM_053691	Nek2	NIMA-related kinase 2
NM_001013134	Nek4	serine/threonine-protein kinase Nek4
NM_012987	Nes	nestin
NM_031789	Nfe2l2	nuclear factor erythroid 2-related factor 2
NM_012865	Nfya	nuclear transcription factor Y subunit alpha
NM_001170476	Nkx1-2	NK1 homeobox 2
NM_001107594	Nkx2-3	homeobox protein Nkx-2.3
NM_053651	Nkx2-5	homeobox protein Nkx-2.5

NM_001024360	nod3l	hypothetical protein LOC501101
NM_001007800	N-pac	putative oxidoreductase GLYR1
NM_203340	Npm2	nucleoplasmin-2
NM_024388	Nr4a1	nuclear receptor subfamily 4 group A member 1
NM_001100708	Nrf1	nuclear respiratory factor 1
NM_053731	Ntn1	netrin-1 precursor
NM_001025708	Ogfrl1	opioid growth factor receptor-like protein 1
NM_001106269	Olig3	oligodendrocyte transcription factor 2
NM_001191700	Otud4	OTU domain-containing protein 4
NM_001037496	Otud5	OTU domain-containing protein 5
NM_012721	P2rx6	P2X purinoceptor 6
NM_001009966	Pacsin3	protein kinase C and casein kinase substrate in
NM_001108937	Paip1	polyadenylate-binding protein-interacting
NM_033485	Pawr	PRKC apoptosis WT1 regulator protein
NM_001107787	Pax1	paired box protein Pax-1
NM_053710	Pax3	paired box 3
NM_001039539	Pax9	paired box protein Pax-9
NM_012802	Pdgfra	alpha-type platelet-derived growth factor
NM_130401	Pdzk1ip1	PDZK1-interacting protein 1
NM_001013231	Pea15a	astrocytic phosphoprotein PEA-15
NM_001109487	Pfn3	profilin-3
NM_017034	Pim1	proto-oncogene serine/threonine-protein kinase
NM_053624	Pitx1	pituitary homeobox 1
NM_019334	Pitx2	pituitary homeobox 2 isoform 2
NM_017175	Pkn1	serine/threonine-protein kinase N1
NM_022533	Pllp	plasmolipin
NM_001142915	Plod2	procollagen-lysine,2-oxoglutarate 5-dioxygenase
NM_001107922	Pm20d2	peptidase M20 domain-containing protein 2
NM_001108889	Pou4f3	POU class 4 homeobox 3
NM_176075	Ppargc1b	peroxisome proliferator-activated receptor gamma

NM_198773	Ppm1e	protein phosphatase 1E
NM_001191072	Ppp1r16b	protein phosphatase 1 regulatory inhibitor
NM_001108577	Ppp2r4	serine/threonine-protein phosphatase 2A
NM_181379	Ppp2r5b	serine/threonine-protein phosphatase 2A 56 kDa
NM_017309	Ppp3r1	calcineurin subunit B type 1
NM_001106613	Ppp4r2	protein phosphatase 4, regulatory subunit 2
NM_021751	Prom1	prominin 1 isoform 1
NM_001012121	Prr5	proline-rich protein 5
NM_001109226	Prrt4	proline-rich transmembrane protein 4
NM_053566	Ptch1	protein patched homolog 1
NM_053964	Ptf1a	pancreas transcription factor 1 subunit alpha
NM_020073	Pth1r	parathyroid hormone/parathyroid hormone-related
NM_031579	Ptp4a1	protein tyrosine phosphatase type IVA 1
NM_001108684	Pum1	pumilio homolog 1
NM_001106715	Pum2	pumilio homolog 2
NM_001108962	R3hdml	R3H domain (binds single-stranded nucleic acids)
NM_031152	Rab11a	ras-related protein Rab-11A
NM_053678	Rax	retinal homeobox protein Rx
NM_013162	Rbp4	retinol-binding protein 4 precursor
NM_001127490	Rfx7	regulatory factor X domain containing 2
NM_001134560	RGD1305627	hypothetical protein LOC314467
NM_001108652	RGD1306151	hypothetical protein LOC362455
NM_001106551	RGD1306208	hypothetical protein LOC296483
NM_001017454	RGD1307799	IST1 homolog
NM_001134596	RGD1308299	hypothetical protein LOC367214
NM_001108286	RGD1311564	hypothetical protein LOC360590
NM_001109262	RGD1559493	hypothetical protein LOC500516
NM_001109345	RGD1563349	hypothetical protein LOC502727
NM_001134589	RGD1566265	hypothetical protein LOC363487
NM_001030034	Rhbdf1	rhomboid family member 1

NM_001013133	Rhobtb2	rho-related BTB domain-containing protein 2
NM_001191665	Rilpl1	RILP-like protein 1
NM_001100488	Rimbp2	RIMS-binding protein 2
NM_001108052	Rin3	ras and Rab interactor 3
NM_001107118	Rnf6	RING finger protein 6
NM_001025740	Rrm2	ribonucleoside-diphosphate reductase subunit M2
NM_001008346	Rrp8	ribosomal RNA-processing protein 8
NM_001008827	RT1-A1	RT1 class Ia, locus A1
NM_001008832	RT1-CE1	RT1 class I, locus CE1
NM_001008833	RT1-CE10	RT1 class I, locus CE10
NM_001033985	RT1-CE14	RT1 class I, locus CE14 isoform 2
NM_012645	RT1-EC2	class I histocompatibility antigen, Non-RT1.A
NM_001109471	S100a7a	protein S100-A15A
NM_001013985	Sccpdh	probable saccharopine dehydrogenase
NM_198748	Scin	adseverin
NM_017247	Scn10a	sodium channel protein type 10 subunit alpha
NM_012648	Scnn1b	amiloride-sensitive sodium channel subunit beta
NM_022670	Sct	secretin precursor
NM_001107637	Sec63	translocation protein SEC63 homolog
NM_001166396	Selv	selenoprotein V
NM_001107091	Sema5b	sema domain, seven thrombospondin repeats (type
NM_017308	Sema6c	semaphorin-6C precursor
NM_001137647	Sh3bgrl2	SH3 domain-binding glutamic acid-rich-like
NM_001191936	Shisa2	protein shisa-2 homolog
NM_001191922	Shisa6	protein shisa-6 homolog
NM_134457	Siah2	E3 ubiquitin-protein ligase SIAH2
NM_001107641	Sim1	single-minded homolog 1
NM_001004089	Sipa1	signal-induced proliferation-associated protein
NM_053759	Six1	sine oculis-related homeobox 1 homolog
NM_023990	Six3	homeobox protein SIX3

NM_001013144	Slc12a7	solute carrier family 12 member 7
NM_012716	Slc16a1	monocarboxylate transporter 1
NM_053427	Slc17a6	vesicular glutamate transporter 2
NM_031663	Slc18a3	vesicular acetylcholine transporter
NM_177421	Slc22a17	solute carrier family 22 member 17
NM_019230	Slc22a3	solute carrier family 22 member 3
NM_017316	Slc23a2	solute carrier family 23 member 2
NM_001108051	Slc24a4	sodium/potassium/calcium exchanger 4
NM_001013996	Slc25a37	mitoferrin-1
NM_019214	Slc26a4	pendrin
NM_031736	Slc27a2	very long-chain acyl-CoA synthetase
NM_001107522	Slc35d3	solute carrier family 35 member D3
NM_001134687	Slc35e3	solute carrier family 35 member E3
NM_053424	Slc4a4	electrogenic sodium bicarbonate cotransporter 1
NM_203334	Slc6a5	sodium- and chloride-dependent glycine
NM_078620	Slc8a3	sodium/calcium exchanger 3 precursor
NM_022953	Slit1	slit homolog 1 protein precursor
NM_013095	Smad3	mothers against decapentaplegic homolog 3
NM_001107419	Smarca5	SWI/SNF-related matrix-associated
NM_001108752	Smarcd1	SWI/SNF-related matrix-associated
NM_001106832	Snx22	sorting nexin-22
NM_001107902	Sox17	transcription factor SOX-17
NM_031792	Spag4	sperm-associated antigen 4 protein
NM_001106125	Spag6l	sperm associated antigen 6-like
NM_199374	Spata18	spermatogenesis-associated protein 18
NM_133386	Sphk1	sphingosine kinase 1
NM_175843	Sqstm1	sequestosome-1 isoform 1
NM_181550	Sqstm1	sequestosome-1 isoform 2
NM_012659	Sst	somatostatin precursor
NM_012747	Stat3	signal transducer and activator of transcription

NM_001108883	Suv39h2	histone-lysine N-methyltransferase SUV39H2
NM_022191	Syt6	synaptotagmin-6
NM_001004107	Tacc1	transforming, acidic coiled-coil containing
NM_001170455	Tada2b	transcriptional adaptor 2B
NM_001013127	Tagln2	transgelin-2
NM_001013245	Tbca	tubulin-specific chaperone A
NM_001191070	Tbr1	T-box brain protein 1
NM_001108322	Tbx1	T-box transcription factor TBX1
NM_001108132	Tbx20	T-box 20
NM_001107034	Tbx4	T-box transcription factor TBX4
NM_001009964	Tbx5	T-box transcription factor TBX5
NM_001130077	Tcerg1l	transcription elongation regulator 1-like
NM_001032397	Tcf21	transcription factor 21
NM_001106896	Tcfap2b	transcription factor AP-2-beta
NM_019194	Tef	thyrotroph embryonic factor
NM_012671	Tgfa	protransforming growth factor alpha
NM_031132	Tgfbr2	TGF-beta receptor type-2 precursor
NM_001105758	Tmed7	transmembrane emp24 domain-containing protein 7
NM_001108795	Tmeff2	tomoregulin-2
NM_001106280	Tmem126b	transmembrane protein 126B
NM_001134410	Tmem132e	transmembrane protein 132E
NM_001107476	Tmem150b	transmembrane protein 150B
NM_001109480	Tmem229a	transmembrane protein 229A
NM_001108045	Tmem63c	transmembrane protein 63C
NM_001127528	Tmprss13	transmembrane protease serine 13
NM_001108998	Tmprss4	transmembrane protease serine 4
NM_001108873	Tnfrsf10b	tumor necrosis factor receptor superfamily,
NM_001191810	Tns1	tensin 1
NM_001105818	Tp53i13	tumor protein p53-inducible protein 13
NM_013046	Trh	prothyroliberin

NM_053916	Trim28	transcription intermediary factor 1-beta
NM_031786	Trim3	tripartite motif-containing protein 3
NM_001106453	Trim45	tripartite motif-containing protein 45
NM_001109912	Tsc22d1	TSC22 domain family protein 1 isoform 1
NM_001109227	Tspan33	tetraspanin-33
NM_001108663	Tstd2	thiosulfate sulfurtransferase/rhodanese-like
NM_001109119	Tubb2a	tubulin beta-2A chain
NM_001037643	Ube2z	ubiquitin-conjugating enzyme E2 Z
NM_001003709	Ufc1	ubiquitin-fold modifier-conjugating enzyme 1
NM_145184	Usp15	ubiquitin carboxyl-terminal hydrolase 15
NM_001106120	Usp6nl	USP6 N-terminal-like protein
NM_013155	Vldlr	very low-density lipoprotein receptor precursor
NM_001109312	Vwc2	brorin
NM_175579	Wnk4	serine/threonine-protein kinase WNK4
NM_001191848	Wnt2b	protein Wnt-2b
NM_001108226	Wnt6	protein Wnt-6
NM_001107055	Wnt9b	protein Wnt-9b
NM_022231	Xiap	baculoviral IAP repeat-containing protein 4
NM_001105992	Xpr1	xenotropic and polytropic retrovirus receptor 1
NM_022296	Xylt2	xylosyltransferase 2
NM_001034831	Zfp384	zinc finger protein 384 isoform 2
NM_001034830	Zfp384	zinc finger protein 384 isoform 1
NM_001109470	Zfp385a	zinc finger protein 385A isoform 2
NM_001012093	Zfp64	zinc finger protein 64
NM_001109225	Zfp800	zinc finger protein 800
NM_001030038	Znf518a	zinc finger protein 518A
NM_053761	Zyx	zyxin
