# Supplementary material for: Interplay of miR-137 and EZH2 contributes to the genome-wide redistribution of H3K27me3 underlying the Pb-induced memory impairment
Source: Cell Death Dis. 2019 Sep 11;10(9):671. doi: 10.1038/s41419-019-1912-7 (PMC6739382; doi:10.1038/s41419-019-1912-7)
Supplement: Supplementary file 13 — Dataset 5 [file 41419_2019_1912_MOESM13_ESM.pdf]

Enriched GO terms upon Pb exposure

Category	Term	Count	%	P-Value
SP_PIR_KEYWORDS	phosphoprotein	165	27.1	1.40E-04
SP_PIR_KEYWORDS	membrane	147	24.1	5.20E-02
GOTERM_CC_FAT	plasma membrane	115	18.9	3.90E-06
SP_PIR_KEYWORDS	nucleus	87	14.3	3.60E-03
GOTERM_BP_FAT	regulation of transcription	86	14.1	3.90E-06
GOTERM_MF_FAT	nucleotide binding	82	13.5	1.10E-02
SP_PIR_KEYWORDS	cytoplasm	82	13.5	3.20E-02
GOTERM_MF_FAT	DNA binding	70	11.5	7.80E-05
GOTERM_MF_FAT	purine ribonucleotide binding	69	11.3	6.20E-03
GOTERM_MF_FAT	ribonucleotide binding	69	11.3	6.40E-03
GOTERM_MF_FAT	purine nucleotide binding	69	11.3	1.80E-02
GOTERM_CC_FAT	plasma membrane part	66	10.8	7.40E-04
GOTERM_MF_FAT	transcription regulator activity	64	10.5	1.20E-06
GOTERM_BP_FAT	regulation of RNA metabolic process	62	10.2	1.10E-03
SP_PIR_KEYWORDS	transport	60	9.9	1.30E-02
GOTERM_BP_FAT	regulation of transcription, DNA-dependent	59	9.7	2.60E-03
GOTERM_MF_FAT	purine nucleoside binding	58	9.5	2.90E-02
GOTERM_MF_FAT	nucleoside binding	58	9.5	3.40E-02
GOTERM_MF_FAT	adenyl ribonucleotide binding	57	9.4	1.10E-02
GOTERM_MF_FAT	adenyl nucleotide binding	57	9.4	3.10E-02
GOTERM_MF_FAT	ATP binding	56	9.2	1.10E-02
GOTERM_CC_FAT	membrane-enclosed lumen	56	9.2	1.20E-02
SP_PIR_KEYWORDS	transcription regulation	55	9	1.20E-08
SP_PIR_KEYWORDS	Transcription	55	9	1.50E-07
GOTERM_BP_FAT	positive regulation of macromolecule metabolic process	55	9	7.70E-07
GOTERM_CC_FAT	cytosol	55	9	1.80E-03
GOTERM_CC_FAT	organelle lumen	55	9	1.10E-02
SP_PIR_KEYWORDS	cell membrane	54	8.9	8.80E-02

GOTERM_BP_FAT	transcription	53	8.7	1.10E-08
GOTERM_CC_FAT	intracellular organelle lumen	53	8.7	1.30E-02
SP_PIR_KEYWORDS	alternative splicing	52	8.5	6.60E-03
UP_SEQ_FEATURE	splice variant	51	8.4	1.90E-02
GOTERM_BP_FAT	intracellular signaling cascade	48	7.9	1.70E-03
GOTERM_CC_FAT	cytoskeleton	48	7.9	1.80E-03
GOTERM_CC_FAT	nuclear lumen	46	7.6	4.00E-03
GOTERM_BP_FAT	positive regulation of nucleobase, nucleoside, nucleotide and nucleic acid metabolic process	44	7.2	1.60E-06
GOTERM_BP_FAT	positive regulation of nitrogen compound metabolic process	44	7.2	3.40E-06
GOTERM_BP_FAT	positive regulation of biosynthetic process	44	7.2	2.20E-05
SP_PIR_KEYWORDS	dna-binding	44	7.2	1.20E-04
GOTERM_BP_FAT	positive regulation of macromolecule biosynthetic process	43	7.1	8.60E-06
GOTERM_MF_FAT	transcription factor activity	43	7.1	1.20E-05
GOTERM_BP_FAT	positive regulation of cellular biosynthetic process	43	7.1	3.10E-05
GOTERM_BP_FAT	phosphate metabolic process	43	7.1	5.00E-03
GOTERM_BP_FAT	phosphorus metabolic process	43	7.1	5.10E-03
SP_PIR_KEYWORDS	atp-binding	42	6.9	3.90E-02
GOTERM_BP_FAT	positive regulation of gene expression	40	6.6	4.10E-06
GOTERM_BP_FAT	positive regulation of transcription	39	6.4	5.80E-06
GOTERM_CC_FAT	nucleoplasm	39	6.4	1.10E-03
GOTERM_BP_FAT	regulation of transcription from RNA polymerase II promoter	38	6.2	1.90E-04
GOTERM_CC_FAT	cell projection	38	6.2	4.20E-03
GOTERM_BP_FAT	neuron differentiation	37	6.1	1.00E-06
GOTERM_BP_FAT	positive regulation of RNA metabolic process	37	6.1	1.20E-06
GOTERM_BP_FAT	phosphorylation	37	6.1	6.90E-03
GOTERM_CC_FAT	Golgi apparatus	36	5.9	3.30E-03
GOTERM_BP_FAT	positive regulation of transcription, DNA-dependent	35	5.7	6.80E-06
GOTERM_BP_FAT	ion transport	35	5.7	7.40E-03
GOTERM_MF_FAT	transcription activator activity	33	5.4	6.50E-10
GOTERM_BP_FAT	protein amino acid phosphorylation	33	5.4	7.10E-03

GOTERM_CC_FAT	cytoskeletal part	33	5.4	2.90E-02
GOTERM_MF_FAT	sequence-specific DNA binding	32	5.3	4.60E-04
GOTERM_BP_FAT	positive regulation of molecular function	32	5.3	6.40E-04
GOTERM_BP_FAT	regulation of cell proliferation	32	5.3	3.10E-02
GOTERM_MF_FAT	protein dimerization activity	31	5.1	2.10E-03
SP_PIR_KEYWORDS	kinase	31	5.1	5.60E-03
GOTERM_BP_FAT	negative regulation of macromolecule metabolic process	31	5.1	3.30E-02
GOTERM_BP_FAT	homeostatic process	31	5.1	1.00E-01
GOTERM_CC_FAT	vesicle	30	4.9	3.70E-02
GOTERM_CC_FAT	cytoplasmic vesicle	29	4.8	3.00E-02
UP_SEQ_FEATURE	nucleotide phosphate-binding region:ATP	29	4.8	3.30E-02
GOTERM_BP_FAT	protein localization	29	4.8	9.50E-02
GOTERM_BP_FAT	death	28	4.6	6.70E-04
GOTERM_BP_FAT	positive regulation of catalytic activity	28	4.6	1.50E-03
GOTERM_MF_FAT	enzyme binding	28	4.6	5.50E-03
GOTERM_MF_FAT	protein kinase activity	28	4.6	2.80E-02
SP_PIR_KEYWORDS	ion transport	28	4.6	3.30E-02
GOTERM_CC_FAT	endomembrane system	28	4.6	9.60E-02
GOTERM_BP_FAT	programmed cell death	27	4.4	2.80E-04
GOTERM_BP_FAT	positive regulation of transcription from RNA polymerase II promoter	27	4.4	3.90E-04
GOTERM_BP_FAT	cell death	27	4.4	1.10E-03
GOTERM_BP_FAT	regulation of phosphorus metabolic process	27	4.4	1.20E-03
GOTERM_BP_FAT	regulation of phosphate metabolic process	27	4.4	1.20E-03
GOTERM_CC_FAT	nucleoplasm part	27	4.4	1.70E-03
GOTERM_BP_FAT	response to endogenous stimulus	27	4.4	5.90E-02
GOTERM_BP_FAT	cell projection organization	26	4.3	3.60E-04
GOTERM_BP_FAT	apoptosis	26	4.3	4.70E-04
GOTERM_CC_FAT	neuron projection	26	4.3	4.20E-03
GOTERM_CC_FAT	cell junction	26	4.3	4.40E-03
SP_PIR_KEYWORDS	lipoprotein	26	4.3	9.30E-03

GOTERM_CC_FAT	membrane-bounded vesicle	26	4.3	5.00E-02
GOTERM_BP_FAT	regulation of cell development	25	4.1	7.50E-07
GOTERM_MF_FAT	protein domain specific binding	25	4.1	3.20E-04
GOTERM_BP_FAT	neuron development	25	4.1	4.80E-04
GOTERM_BP_FAT	response to hormone stimulus	25	4.1	4.90E-02
GOTERM_CC_FAT	cytoplasmic membrane-bounded vesicle	25	4.1	5.20E-02
GOTERM_BP_FAT	regulation of phosphorylation	24	3.9	6.50E-03
GOTERM_BP_FAT	negative regulation of gene expression	24	3.9	1.90E-02
GOTERM_BP_FAT	negative regulation of nucleobase, nucleoside, nucleotide and nucleic acid metabolic process	24	3.9	2.50E-02
GOTERM_BP_FAT	negative regulation of nitrogen compound metabolic process	24	3.9	2.90E-02
GOTERM_BP_FAT	intracellular transport	24	3.9	5.60E-02
SP_PIR_KEYWORDS	activator	23	3.8	5.30E-06
GOTERM_BP_FAT	neuron projection development	23	3.8	9.60E-05
GOTERM_BP_FAT	regulation of cellular protein metabolic process	23	3.8	1.90E-02
GOTERM_BP_FAT	chemical homeostasis	23	3.8	8.80E-02
GOTERM_BP_FAT	regulation of neurogenesis	22	3.6	4.40E-06
GOTERM_BP_FAT	regulation of nervous system development	22	3.6	2.00E-05
GOTERM_BP_FAT	positive regulation of developmental process	22	3.6	1.40E-03
GOTERM_BP_FAT	embryonic morphogenesis	22	3.6	2.20E-03
GOTERM_BP_FAT	cell morphogenesis	22	3.6	4.10E-03
GOTERM_BP_FAT	cellular component morphogenesis	22	3.6	1.20E-02
INTERPRO	Protein kinase, core	22	3.6	2.10E-02
GOTERM_BP_FAT	negative regulation of transcription	22	3.6	2.70E-02
GOTERM_BP_FAT	pattern specification process	21	3.4	1.80E-04
GOTERM_BP_FAT	tube development	21	3.4	2.00E-04
KEGG_PATHWAY	Pathways in cancer	21	3.4	3.40E-03
GOTERM_CC_FAT	cell surface	21	3.4	7.40E-03
INTERPRO	Protein kinase, ATP binding site	21	3.4	1.10E-02
UP_SEQ_FEATURE	binding site:ATP	21	3.4	2.30E-02
GOTERM_BP_FAT	chordate embryonic development	21	3.4	2.30E-02

GOTERM_BP_FAT	embryonic development ending in birth or egg hatching	21	3.4	2.60E-02
GOTERM_MF_FAT	protein serine/threonine kinase activity	21	3.4	3.20E-02
GOTERM_BP_FAT	cell morphogenesis involved in differentiation	20	3.3	4.00E-04
GOTERM_BP_FAT	positive regulation of cell differentiation	20	3.3	6.60E-04
GOTERM_BP_FAT	regulation of transferase activity	20	3.3	4.60E-03
GOTERM_MF_FAT	transcription factor binding	20	3.3	5.90E-03
GOTERM_BP_FAT	negative regulation of RNA metabolic process	20	3.3	1.20E-02
SP_PIR_KEYWORDS	ubl conjugation	20	3.3	3.30E-02
GOTERM_BP_FAT	positive regulation of cell proliferation	20	3.3	5.30E-02
SP_PIR_KEYWORDS	golgi apparatus	20	3.3	6.60E-02
KEGG_PATHWAY	MAPK signaling pathway	19	3.1	2.50E-03
INTERPRO	Serine/threonine protein kinase, active site	19	3.1	3.30E-03
GOTERM_BP_FAT	regulation of protein kinase activity	19	3.1	3.70E-03
GOTERM_BP_FAT	regulation of kinase activity	19	3.1	6.20E-03
GOTERM_BP_FAT	regulation of growth	19	3.1	9.60E-03
GOTERM_BP_FAT	negative regulation of transcription, DNA-dependent	19	3.1	2.00E-02
GOTERM_BP_FAT	cell-cell signaling	19	3.1	2.50E-02
GOTERM_BP_FAT	protein catabolic process	19	3.1	2.70E-02
SP_PIR_KEYWORDS	cytoskeleton	19	3.1	3.10E-02
GOTERM_MF_FAT	passive transmembrane transporter activity	19	3.1	4.60E-02
GOTERM_MF_FAT	channel activity	19	3.1	4.60E-02
SP_PIR_KEYWORDS	cell junction	19	3.1	5.30E-02
GOTERM_CC_FAT	synapse	19	3.1	6.90E-02
GOTERM_CC_FAT	intrinsic to plasma membrane	19	3.1	7.40E-02
GOTERM_MF_FAT	cytoskeletal protein binding	19	3.1	8.80E-02
GOTERM_BP_FAT	neuron projection morphogenesis	18	3	7.30E-04
GOTERM_BP_FAT	cell projection morphogenesis	18	3	2.20E-03
GOTERM_BP_FAT	cell part morphogenesis	18	3	3.40E-03
SP_PIR_KEYWORDS	serine/threonine-protein kinase	18	3	8.30E-03
GOTERM_BP_FAT	negative regulation of molecular function	18	3	1.50E-02

INTERPRO	Serine/threonine protein kinase-related	18	3	2.00E-02
GOTERM_BP_FAT	proteolysis involved in cellular protein catabolic process	18	3	2.70E-02
GOTERM_BP_FAT	cellular protein catabolic process	18	3	2.90E-02
UP_SEQ_FEATURE	domain:Protein kinase	18	3	3.30E-02
GOTERM_BP_FAT	negative regulation of programmed cell death	18	3	5.40E-02
GOTERM_BP_FAT	negative regulation of cell death	18	3	5.50E-02
GOTERM_BP_FAT	regionalization	17	2.8	5.00E-04
GOTERM_BP_FAT	negative regulation of cell differentiation	17	2.8	1.70E-03
GOTERM_BP_FAT	tissue morphogenesis	17	2.8	3.40E-03
UP_SEQ_FEATURE	compositionally biased region:Pro-rich	17	2.8	3.90E-02
GOTERM_MF_FAT	protein homodimerization activity	17	2.8	5.60E-02
GOTERM_BP_FAT	negative regulation of apoptosis	17	2.8	8.30E-02
GOTERM_MF_FAT	substrate specific channel activity	17	2.8	1.00E-01
GOTERM_BP_FAT	regulation of neuron differentiation	16	2.6	4.00E-04
GOTERM_BP_FAT	axonogenesis	16	2.6	1.20E-03
GOTERM_BP_FAT	cell morphogenesis involved in neuron differentiation	16	2.6	3.50E-03
GOTERM_BP_FAT	regulation of cellular component size	16	2.6	9.30E-03
GOTERM_BP_FAT	epithelium development	16	2.6	1.50E-02
GOTERM_BP_FAT	regulation of hydrolase activity	16	2.6	2.70E-02
GOTERM_BP_FAT	skeletal system development	16	2.6	2.80E-02
GOTERM_BP_FAT	negative regulation of cell proliferation	16	2.6	2.80E-02
GOTERM_BP_FAT	regulation of protein modification process	16	2.6	4.20E-02
GOTERM_BP_FAT	enzyme linked receptor protein signaling pathway	16	2.6	4.30E-02
GOTERM_BP_FAT	modification-dependent macromolecule catabolic process	16	2.6	4.80E-02
GOTERM_BP_FAT	modification-dependent protein catabolic process	16	2.6	4.80E-02
GOTERM_BP_FAT	tube morphogenesis	15	2.5	5.80E-04
GOTERM_BP_FAT	regulation of cell motion	15	2.5	3.30E-03
GOTERM_BP_FAT	regulation of locomotion	15	2.5	3.60E-03
GOTERM_BP_FAT	positive regulation of protein kinase activity	15	2.5	4.20E-03
GOTERM_CC_FAT	axon	15	2.5	5.00E-03

INTERPRO	Serine/threonine protein kinase	15	2.5	5.90E-03
GOTERM_BP_FAT	positive regulation of kinase activity	15	2.5	6.00E-03
GOTERM_BP_FAT	positive regulation of transferase activity	15	2.5	8.90E-03
GOTERM_BP_FAT	embryonic organ development	15	2.5	1.20E-02
GOTERM_MF_FAT	protein heterodimerization activity	15	2.5	1.60E-02
SMART	S_TKc	15	2.5	2.30E-02
GOTERM_BP_FAT	regulation of cell cycle	15	2.5	3.30E-02
GOTERM_CC_FAT	cell soma	15	2.5	3.70E-02
GOTERM_CC_FAT	cell projection part	15	2.5	3.80E-02
GOTERM_BP_FAT	protein kinase cascade	15	2.5	5.70E-02
GOTERM_BP_FAT	monovalent inorganic cation transport	15	2.5	9.80E-02
GOTERM_BP_FAT	regulation of cell morphogenesis involved in differentiation	14	2.3	1.30E-05
GOTERM_CC_FAT	anchored to membrane	14	2.3	9.60E-05
GOTERM_BP_FAT	regulation of cell morphogenesis	14	2.3	3.20E-04
GOTERM_MF_FAT	protein kinase binding	14	2.3	1.80E-03
GOTERM_BP_FAT	regulation of cell migration	14	2.3	3.10E-03
GOTERM_MF_FAT	kinase binding	14	2.3	5.90E-03
GOTERM_CC_FAT	basolateral plasma membrane	14	2.3	7.30E-03
GOTERM_CC_FAT	transcription factor complex	14	2.3	8.30E-03
GOTERM_BP_FAT	gland development	14	2.3	1.50E-02
SP_PIR_KEYWORDS	Apoptosis	14	2.3	2.00E-02
GOTERM_BP_FAT	negative regulation of transcription from RNA polymerase II promoter	14	2.3	5.10E-02
GOTERM_CC_FAT	Golgi apparatus part	14	2.3	6.80E-02
GOTERM_BP_FAT	chromatin organization	14	2.3	7.10E-02
GOTERM_BP_FAT	positive regulation of cell development	13	2.1	2.90E-05
GOTERM_BP_FAT	regulation of cell projection organization	13	2.1	3.30E-04
GOTERM_BP_FAT	regulation of cell growth	13	2.1	9.90E-03
GOTERM_BP_FAT	regulation of organelle organization	13	2.1	1.10E-02
GOTERM_MF_FAT	alkali metal ion binding	13	2.1	2.10E-02
SP_PIR_KEYWORDS	ATP	13	2.1	2.80E-02

GOTERM_MF_FAT	phosphatase activity	13	2.1	5.00E-02
GOTERM_CC_FAT	proteinaceous extracellular matrix	13	2.1	5.50E-02
GOTERM_BP_FAT	small GTPase mediated signal transduction	13	2.1	7.30E-02
GOTERM_BP_FAT	negative regulation of catalytic activity	13	2.1	8.30E-02
GOTERM_BP_FAT	regulation of neuron projection development	12	2	2.30E-04
SP_PIR_KEYWORDS	DNA binding	12	2	9.20E-03
GOTERM_BP_FAT	urogenital system development	12	2	1.30E-02
GOTERM_BP_FAT	second-messenger-mediated signaling	12	2	2.40E-02
GOTERM_BP_FAT	positive regulation of cellular component organization	12	2	4.60E-02
KEGG_PATHWAY	Focal adhesion	12	2	5.50E-02
GOTERM_BP_FAT	microtubule-based process	12	2	5.70E-02
GOTERM_CC_FAT	actin cytoskeleton	12	2	8.30E-02
GOTERM_BP_FAT	protein oligomerization	12	2	8.80E-02
SP_PIR_KEYWORDS	ubl conjugation pathway	12	2	9.20E-02
GOTERM_BP_FAT	heart development	12	2	9.30E-02
GOTERM_BP_FAT	growth	12	2	9.70E-02
GOTERM_BP_FAT	positive regulation of neurogenesis	11	1.8	2.50E-04
KEGG_PATHWAY	Neurotrophin signaling pathway	11	1.8	8.00E-03
GOTERM_BP_FAT	negative regulation of cellular component organization	11	1.8	8.50E-03
GOTERM_BP_FAT	anterior/posterior pattern formation	11	1.8	1.40E-02
GOTERM_MF_FAT	structure-specific DNA binding	11	1.8	1.90E-02
KEGG_PATHWAY	Wnt signaling pathway	11	1.8	2.00E-02
GOTERM_CC_FAT	ion channel complex	11	1.8	2.40E-02
GOTERM_BP_FAT	regulation of hormone levels	11	1.8	3.40E-02
GOTERM_CC_FAT	internal side of plasma membrane	11	1.8	3.80E-02
GOTERM_MF_FAT	transcription cofactor activity	11	1.8	4.20E-02
INTERPRO	Nucleotide-binding, alpha-beta plait	11	1.8	4.50E-02
GOTERM_CC_FAT	cell-cell junction	11	1.8	4.60E-02
SP_PIR_KEYWORDS	repressor	11	1.8	4.70E-02
GOTERM_BP_FAT	regulation of cell size	11	1.8	8.40E-02

GOTERM_MF_FAT	enzyme activator activity	11	1.8	8.40E-02
GOTERM_BP_FAT	regulation of axonogenesis	10	1.6	3.60E-04
GOTERM_BP_FAT	protein heterooligomerization	10	1.6	2.20E-03
KEGG_PATHWAY	Melanogenesis	10	1.6	3.00E-03
GOTERM_BP_FAT	regulation of protein transport	10	1.6	5.90E-03
GOTERM_BP_FAT	axon guidance	10	1.6	7.60E-03
GOTERM_BP_FAT	regulation of establishment of protein localization	10	1.6	8.10E-03
GOTERM_BP_FAT	regulation of cytoskeleton organization	10	1.6	9.60E-03
KEGG_PATHWAY	Oocyte meiosis	10	1.6	1.00E-02
GOTERM_BP_FAT	lung development	10	1.6	1.00E-02
GOTERM_MF_FAT	symporter activity	10	1.6	1.10E-02
GOTERM_BP_FAT	respiratory tube development	10	1.6	1.10E-02
GOTERM_BP_FAT	skeletal system morphogenesis	10	1.6	1.30E-02
GOTERM_BP_FAT	respiratory system development	10	1.6	1.50E-02
GOTERM_BP_FAT	regulation of protein localization	10	1.6	2.60E-02
GOTERM_BP_FAT	anion transport	10	1.6	2.90E-02
GOTERM_CC_FAT	apical plasma membrane	10	1.6	3.00E-02
GOTERM_BP_FAT	negative regulation of transport	10	1.6	4.10E-02
INTERPRO	Homeodomain-related	10	1.6	4.60E-02
GOTERM_BP_FAT	anti-apoptosis	10	1.6	5.00E-02
GOTERM_MF_FAT	chromatin binding	10	1.6	5.60E-02
GOTERM_BP_FAT	morphogenesis of an epithelium	10	1.6	6.90E-02
SP_PIR_KEYWORDS	Homeobox	10	1.6	7.30E-02
UP_SEQ_FEATURE	propeptide:Removed in mature form	10	1.6	7.90E-02
GOTERM_CC_FAT	microtubule	10	1.6	8.60E-02
GOTERM_BP_FAT	regulation of synaptic transmission	10	1.6	9.40E-02
GOTERM_BP_FAT	chromatin modification	10	1.6	9.60E-02
SP_PIR_KEYWORDS	gpi-anchor	9	1.5	4.60E-03
GOTERM_BP_FAT	branching morphogenesis of a tube	9	1.5	6.20E-03
GOTERM_BP_FAT	limb morphogenesis	9	1.5	1.30E-02

GOTERM_BP_FAT	appendage morphogenesis	9	1.5	1.30E-02
GOTERM_BP_FAT	limb development	9	1.5	1.60E-02
GOTERM_BP_FAT	appendage development	9	1.5	1.60E-02
GOTERM_BP_FAT	positive regulation of cell motion	9	1.5	1.70E-02
UP_SEQ_FEATURE	compositionally biased region:Poly-Gly	9	1.5	1.80E-02
GOTERM_BP_FAT	negative regulation of growth	9	1.5	1.90E-02
GOTERM_MF_FAT	RNA polymerase II transcription factor activity	9	1.5	2.40E-02
SP_PIR_KEYWORDS	Symport	9	1.5	2.60E-02
UP_SEQ_FEATURE	DNA-binding region:Basic motif	9	1.5	2.80E-02
INTERPRO	PDZ/DHR/GLGF	9	1.5	3.20E-02
GOTERM_BP_FAT	regulation of cellular component biogenesis	9	1.5	3.50E-02
GOTERM_BP_FAT	morphogenesis of a branching structure	9	1.5	3.50E-02
GOTERM_BP_FAT	regulation of MAP kinase activity	9	1.5	3.50E-02
GOTERM_BP_FAT	kidney development	9	1.5	4.00E-02
GOTERM_BP_FAT	regulation of gene-specific transcription	9	1.5	5.00E-02
KEGG_PATHWAY	Cell cycle	9	1.5	5.40E-02
SP_PIR_KEYWORDS	phosphotransferase	9	1.5	6.60E-02
SMART	PDZ	9	1.5	7.30E-02
GOTERM_MF_FAT	growth factor activity	9	1.5	7.30E-02
GOTERM_BP_FAT	positive regulation of hydrolase activity	9	1.5	8.40E-02
GOTERM_BP_FAT	cell fate commitment	9	1.5	9.00E-02
GOTERM_BP_FAT	potassium ion transport	9	1.5	9.60E-02
GOTERM_BP_FAT	mesenchymal cell development	8	1.3	7.90E-04
GOTERM_BP_FAT	mesenchymal cell differentiation	8	1.3	9.00E-04
GOTERM_BP_FAT	mesenchyme development	8	1.3	1.00E-03
KEGG_PATHWAY	Hedgehog signaling pathway	8	1.3	1.40E-03
KEGG_PATHWAY	Long-term potentiation	8	1.3	5.90E-03
GOTERM_BP_FAT	cartilage development	8	1.3	9.90E-03
GOTERM_BP_FAT	gliogenesis	8	1.3	1.10E-02
UP_SEQ_FEATURE	domain:Leucine-zipper	8	1.3	1.20E-02

GOTERM_BP_FAT	embryonic appendage morphogenesis	8	1.3	1.90E-02
GOTERM_BP_FAT	embryonic limb morphogenesis	8	1.3	1.90E-02
KEGG_PATHWAY	TGF-beta signaling pathway	8	1.3	2.20E-02
GOTERM_MF_FAT	transcription coactivator activity	8	1.3	2.60E-02
GOTERM_BP_FAT	positive regulation of cell migration	8	1.3	3.10E-02
GOTERM_MF_FAT	manganese ion binding	8	1.3	3.70E-02
GOTERM_BP_FAT	activation of protein kinase activity	8	1.3	4.40E-02
GOTERM_BP_FAT	positive regulation of locomotion	8	1.3	5.00E-02
GOTERM_BP_FAT	protein amino acid dephosphorylation	8	1.3	5.30E-02
GOTERM_BP_FAT	epidermis development	8	1.3	5.30E-02
GOTERM_BP_FAT	sodium ion transport	8	1.3	6.70E-02
GOTERM_BP_FAT	epithelial cell differentiation	8	1.3	6.70E-02
GOTERM_BP_FAT	ectoderm development	8	1.3	7.60E-02
GOTERM_MF_FAT	anion transmembrane transporter activity	8	1.3	9.90E-02
GOTERM_BP_FAT	regulation of cell adhesion	8	1.3	1.00E-01
GOTERM_BP_FAT	gut development	7	1.1	3.70E-03
KEGG_PATHWAY	Inositol phosphate metabolism	7	1.1	6.60E-03
GOTERM_BP_FAT	negative regulation of neurogenesis	7	1.1	9.00E-03
GOTERM_BP_FAT	negative regulation of cell development	7	1.1	1.10E-02
GOTERM_BP_FAT	glial cell differentiation	7	1.1	1.30E-02
GOTERM_BP_FAT	regulation of actin cytoskeleton organization	7	1.1	1.80E-02
GOTERM_BP_FAT	regulation of actin filament-based process	7	1.1	2.20E-02
GOTERM_MF_FAT	small GTPase binding	7	1.1	3.50E-02
KEGG_PATHWAY	Chronic myeloid leukemia	7	1.1	3.50E-02
GOTERM_BP_FAT	positive regulation of gene-specific transcription	7	1.1	3.60E-02
GOTERM_BP_FAT	endocrine system development	7	1.1	3.60E-02
GOTERM_MF_FAT	kinase regulator activity	7	1.1	4.10E-02
GOTERM_BP_FAT	negative regulation of cell growth	7	1.1	4.50E-02
GOTERM_MF_FAT	solute:cation symporter activity	7	1.1	4.60E-02
KEGG_PATHWAY	Gap junction	7	1.1	4.90E-02

GOTERM_BP_FAT	regulation of synaptic plasticity	7	1.1	5.00E-02
GOTERM_BP_FAT	inorganic anion transport	7	1.1	5.00E-02
GOTERM_MF_FAT	GTPase binding	7	1.1	5.60E-02
GOTERM_BP_FAT	placenta development	7	1.1	6.60E-02
GOTERM_BP_FAT	negative regulation of cell size	7	1.1	6.60E-02
GOTERM_BP_FAT	regulation of specific transcription from RNA polymerase II promoter	7	1.1	7.90E-02
GOTERM_BP_FAT	microtubule-based movement	7	1.1	8.20E-02
GOTERM_MF_FAT	neurotransmitter binding	7	1.1	9.20E-02
GOTERM_MF_FAT	double-stranded DNA binding	7	1.1	1.00E-01
GOTERM_BP_FAT	negative regulation of cell projection organization	6	1	6.80E-03
GOTERM_BP_FAT	regulation of muscle development	6	1	1.50E-02
KEGG_PATHWAY	Notch signaling pathway	6	1	2.10E-02
INTERPRO	Basic-leucine zipper (bZIP) transcription factor	6	1	2.70E-02
GOTERM_BP_FAT	chloride transport	6	1	2.70E-02
GOTERM_CC_FAT	chromatin remodeling complex	6	1	2.80E-02
GOTERM_BP_FAT	phosphoinositide-mediated signaling	6	1	4.90E-02
SMART	BRLZ	6	1	5.00E-02
GOTERM_BP_FAT	positive regulation of cell projection organization	6	1	5.20E-02
GOTERM_MF_FAT	protein kinase regulator activity	6	1	6.00E-02
GOTERM_CC_FAT	growth cone	6	1	6.60E-02
GOTERM_CC_FAT	site of polarized growth	6	1	6.60E-02
UP_SEQ_FEATURE	domain:PDZ	6	1	6.80E-02
KEGG_PATHWAY	Pancreatic cancer	6	1	7.40E-02
KEGG_PATHWAY	Renal cell carcinoma	6	1	7.40E-02
GOTERM_BP_FAT	hindbrain development	6	1	7.60E-02
KEGG_PATHWAY	Phosphatidylinositol signaling system	6	1	7.80E-02
SP_PIR_KEYWORDS	myristate	6	1	7.90E-02
GOTERM_BP_FAT	heart morphogenesis	6	1	8.00E-02
GOTERM_BP_FAT	regulation of protein complex assembly	6	1	8.30E-02
UP_SEQ_FEATURE	compositionally biased region:Poly-Gln	6	1	8.40E-02

GOTERM_CC_FAT	focal adhesion	6	1	8.80E-02
GOTERM_MF_FAT	SH3 domain binding	6	1	8.90E-02
KEGG_PATHWAY	Adherens junction	6	1	9.40E-02
GOTERM_BP_FAT	neural tube development	6	1	1.00E-01
GOTERM_BP_FAT	epithelial to mesenchymal transition	5	0.8	1.20E-03
GOTERM_MF_FAT	transforming growth factor beta receptor binding	5	0.8	1.50E-03
GOTERM_MF_FAT	specific RNA polymerase II transcription factor activity	5	0.8	3.50E-03
GOTERM_CC_FAT	anchored to plasma membrane	5	0.8	5.00E-03
GOTERM_BP_FAT	negative regulation of axonogenesis	5	0.8	1.60E-02
GOTERM_BP_FAT	digestive system development	5	0.8	1.80E-02
GOTERM_BP_FAT	positive regulation of axonogenesis	5	0.8	2.00E-02
GOTERM_BP_FAT	pituitary gland development	5	0.8	2.20E-02
KEGG_PATHWAY	Prion diseases	5	0.8	2.60E-02
GOTERM_BP_FAT	determination of bilateral symmetry	5	0.8	2.90E-02
GOTERM_BP_FAT	determination of symmetry	5	0.8	2.90E-02
GOTERM_BP_FAT	regulation of cell division	5	0.8	3.50E-02
GOTERM_CC_FAT	microvillus	5	0.8	4.00E-02
GOTERM_BP_FAT	negative regulation of specific transcription from RNA polymerase II promoter	5	0.8	4.10E-02
GOTERM_BP_FAT	cell junction organization	5	0.8	4.50E-02
GOTERM_MF_FAT	SMAD binding	5	0.8	5.00E-02
GOTERM_BP_FAT	osteoblast differentiation	5	0.8	5.20E-02
GOTERM_BP_FAT	regulation of striated muscle tissue development	5	0.8	5.20E-02
GOTERM_BP_FAT	diencephalon development	5	0.8	5.20E-02
GOTERM_BP_FAT	activation of adenylate cyclase activity by G-protein signaling pathway	5	0.8	5.50E-02
GOTERM_BP_FAT	regulation of adenylate cyclase activity involved in G-protein signaling	5	0.8	5.50E-02
GOTERM_BP_FAT	positive regulation of adenylate cyclase activity by G-protein signaling pathway	5	0.8	5.50E-02
GOTERM_BP_FAT	glutamine family amino acid metabolic process	5	0.8	5.90E-02
GOTERM_BP_FAT	neuron fate commitment	5	0.8	6.80E-02
GOTERM_BP_FAT	negative regulation of gene-specific transcription	5	0.8	6.80E-02
GOTERM_CC_FAT	trans-Golgi network	5	0.8	7.00E-02

GOTERM_BP_FAT	carbohydrate homeostasis	5	0.8	7.20E-02
GOTERM_BP_FAT	glucose homeostasis	5	0.8	7.20E-02
GOTERM_BP_FAT	regulation of neuronal synaptic plasticity	5	0.8	7.60E-02
GOTERM_BP_FAT	regulation of protein catabolic process	5	0.8	7.60E-02
GOTERM_BP_FAT	negative regulation of hydrolase activity	5	0.8	7.60E-02
KEGG_PATHWAY	Basal cell carcinoma	5	0.8	8.50E-02
GOTERM_BP_FAT	activation of adenylate cyclase activity	5	0.8	8.60E-02
GOTERM_BP_FAT	positive regulation of specific transcription from RNA polymerase II promoter	5	0.8	8.60E-02
GOTERM_BP_FAT	regulation of developmental growth	5	0.8	8.60E-02
UP_SEQ_FEATURE	compositionally biased region:Ala-rich	5	0.8	8.90E-02
GOTERM_BP_FAT	regulation of protein polymerization	5	0.8	9.10E-02
GOTERM_BP_FAT	mesoderm development	5	0.8	9.10E-02
GOTERM_BP_FAT	monocarboxylic acid transport	5	0.8	9.60E-02
GOTERM_BP_FAT	hormone transport	5	0.8	9.60E-02
GOTERM_BP_FAT	positive regulation of protein transport	5	0.8	9.60E-02
GOTERM_BP_FAT	positive regulation of adenylate cyclase activity	5	0.8	9.60E-02
GOTERM_BP_FAT	regulation of proteolysis	5	0.8	9.60E-02
GOTERM_BP_FAT	regulation of mRNA processing	4	0.7	9.80E-03
GOTERM_BP_FAT	cartilage condensation	4	0.7	1.70E-02
GOTERM_BP_FAT	regulation of smoothened signaling pathway	4	0.7	2.30E-02
GOTERM_BP_FAT	positive regulation of developmental growth	4	0.7	2.60E-02
GOTERM_MF_FAT	kinase activator activity	4	0.7	2.90E-02
GOTERM_BP_FAT	autonomic nervous system development	4	0.7	3.00E-02
GOTERM_BP_FAT	microtubule-based transport	4	0.7	3.00E-02
GOTERM_BP_FAT	patterning of blood vessels	4	0.7	3.40E-02
GOTERM_BP_FAT	gut morphogenesis	4	0.7	3.80E-02
GOTERM_BP_FAT	cell-cell junction organization	4	0.7	3.80E-02
INTERPRO	Six-bladed beta-propeller, TolB-like	4	0.7	4.20E-02
GOTERM_BP_FAT	regulation of BMP signaling pathway	4	0.7	4.30E-02
SP_PIR_KEYWORDS	dynein	4	0.7	4.50E-02

GOTERM_MF_FAT	anion:cation symporter activity	4	0.7	4.60E-02
GOTERM_BP_FAT	oligodendrocyte differentiation	4	0.7	4.70E-02
GOTERM_BP_FAT	regulation of axon extension	4	0.7	4.70E-02
GOTERM_BP_FAT	regulation of dephosphorylation	4	0.7	4.70E-02
GOTERM_BP_FAT	positive regulation of protein catabolic process	4	0.7	5.20E-02
GOTERM_CC_FAT	stress fiber	4	0.7	6.10E-02
INTERPRO	Thrombospondin, type 1 repeat	4	0.7	6.20E-02
GOTERM_BP_FAT	digestive tract morphogenesis	4	0.7	6.80E-02
GOTERM_BP_FAT	peptidyl-serine phosphorylation	4	0.7	6.80E-02
GOTERM_CC_FAT	actin filament bundle	4	0.7	7.20E-02
INTERPRO	WW/Rsp5/WWP	4	0.7	7.60E-02
UP_SEQ_FEATURE	region of interest:Ligand-binding	4	0.7	7.80E-02
GOTERM_CC_FAT	actomyosin	4	0.7	8.40E-02
GOTERM_CC_FAT	dynein complex	4	0.7	8.40E-02
SMART	TSP1	4	0.7	9.30E-02
GOTERM_BP_FAT	cytoskeleton-dependent intracellular transport	4	0.7	1.00E-01
GOTERM_BP_FAT	response to endoplasmic reticulum stress	4	0.7	1.00E-01
GOTERM_BP_FAT	regulation of pH	4	0.7	1.00E-01
GOTERM_BP_FAT	compartment specification	3	0.5	6.20E-03
GOTERM_BP_FAT	pathway-restricted SMAD protein phosphorylation	3	0.5	1.00E-02
GOTERM_MF_FAT	ammonia transporter activity	3	0.5	1.50E-02
GOTERM_BP_FAT	ammonium transport	3	0.5	1.50E-02
GOTERM_BP_FAT	somatic stem cell division	3	0.5	2.00E-02
INTERPRO	Yip1 domain	3	0.5	2.30E-02
GOTERM_BP_FAT	rhombomere development	3	0.5	2.60E-02
GOTERM_BP_FAT	regulation of myoblast differentiation	3	0.5	2.60E-02
GOTERM_BP_FAT	foregut morphogenesis	3	0.5	2.60E-02
GOTERM_BP_FAT	regulation of receptor biosynthetic process	3	0.5	2.60E-02
GOTERM_MF_FAT	protein phosphatase type 2A regulator activity	3	0.5	3.30E-02
INTERPRO	B-box, C-terminal	3	0.5	4.10E-02

GOTERM_BP_FAT	osteoblast development	3	0.5	4.90E-02
GOTERM_BP_FAT	stem cell division	3	0.5	4.90E-02
INTERPRO	Zinc finger, MYND-type	3	0.5	5.10E-02
SMART	BBC	3	0.5	5.60E-02
UP_SEQ_FEATURE	region of interest:Linker 2	3	0.5	6.10E-02
GOTERM_BP_FAT	positive regulation of axon extension	3	0.5	6.60E-02
GOTERM_BP_FAT	thyroid gland development	3	0.5	7.50E-02
GOTERM_BP_FAT	regulation of skeletal muscle fiber development	3	0.5	7.50E-02
GOTERM_BP_FAT	glutamine family amino acid catabolic process	3	0.5	7.50E-02
PIR_SUPERFAMILY	PIRSF002350:calmodulin	3	0.5	8.10E-02
GOTERM_BP_FAT	ectodermal gut morphogenesis	3	0.5	8.50E-02
GOTERM_BP_FAT	ectodermal gut development	3	0.5	8.50E-02
GOTERM_BP_FAT	male genitalia development	3	0.5	8.50E-02
GOTERM_BP_FAT	hair follicle morphogenesis	3	0.5	8.50E-02
INTERPRO	Peptidase M14, carboxypeptidase A	3	0.5	8.70E-02
INTERPRO	Zinc finger, ZZ-type	3	0.5	8.70E-02
INTERPRO	Major intrinsic protein	3	0.5	8.70E-02
UP_SEQ_FEATURE	short sequence motif:NPA 2	3	0.5	8.80E-02
UP_SEQ_FEATURE	short sequence motif:NPA 1	3	0.5	8.80E-02
GOTERM_MF_FAT	ligand-dependent nuclear receptor transcription coactivator activity	3	0.5	9.40E-02
GOTERM_MF_FAT	protein kinase activator activity	3	0.5	9.40E-02
GOTERM_BP_FAT	placenta blood vessel development	3	0.5	9.50E-02
GOTERM_BP_FAT	hyperosmotic response	3	0.5	9.50E-02
GOTERM_BP_FAT	neuron recognition	3	0.5	9.50E-02
GOTERM_BP_FAT	inositol metabolic process	3	0.5	9.50E-02
INTERPRO	Speract/scavenger receptor	3	0.5	1.00E-01
GOTERM_MF_FAT	Rab GDP-dissociation inhibitor activity	2	0.3	6.40E-02
INTERPRO	Rab GDI protein	2	0.3	8.20E-02
INTERPRO	MAP Kinase Interacting Kinase	2	0.3	8.20E-02
PIR_SUPERFAMILY	PIRSF019647:GDP dissociation inhibitor XAP-4	2	0.3	8.60E-02

GOTERM_CC_FAT	platelet dense granule membrane	2	0.3	9.40E-02
GOTERM_MF_FAT	galanin receptor activity	2	0.3	9.40E-02
GOTERM_MF_FAT	titin binding	2	0.3	9.40E-02
GOTERM_BP_FAT	lung cell differentiation	2	0.3	9.50E-02
GOTERM_BP_FAT	activation of transmembrane receptor protein tyrosine kinase activity	2	0.3	9.50E-02
GOTERM_BP_FAT	regulation of alkaline phosphatase activity	2	0.3	9.50E-02
GOTERM_BP_FAT	lung epithelial cell differentiation	2	0.3	9.50E-02
