# Supplementary material for: Interplay of miR-137 and EZH2 contributes to the genome-wide redistribution of H3K27me3 underlying the Pb-induced memory impairment
Source: Cell Death Dis. 2019 Sep 11;10(9):671. doi: 10.1038/s41419-019-1912-7 (PMC6739382; doi:10.1038/s41419-019-1912-7)
Supplement: Supplementary file 14 — Dataset 6 [file 41419_2019_1912_MOESM14_ESM.pdf]

# Enriched GO term clusters upon lead exposure

| **Cluster 1** | **Enrichment Score: 5.12** | **Count** | **P-value** |
|---|---|---|---|
| GOTERM_MF_FAT | transcription activator activity | 33 | 6.50E-10 |
| GOTERM_BP_FAT | transcription | 53 | 1.10E-08 |
| SP_PIR_KEYWORDS | transcription regulation | 55 | 1.20E-08 |
| SP_PIR_KEYWORDS | Transcription | 55 | 1.50E-07 |
| GOTERM_BP_FAT | positive regulation of macromolecule metabolic process | 55 | 7.70E-07 |
| GOTERM_BP_FAT | positive regulation of RNA metabolic process | 37 | 1.20E-06 |
| GOTERM_MF_FAT | transcription regulator activity | 64 | 1.20E-06 |
| GOTERM_BP_FAT | positive regulation of nucleobase, nucleoside, nucleotide and nucleic acid metabolic process | 44 | 1.60E-06 |
| GOTERM_BP_FAT | positive regulation of nitrogen compound metabolic process | 44 | 3.40E-06 |
| GOTERM_BP_FAT | regulation of transcription | 86 | 3.90E-06 |
| GOTERM_BP_FAT | positive regulation of gene expression | 40 | 4.10E-06 |
| SP_PIR_KEYWORDS | activator | 23 | 5.30E-06 |
| GOTERM_BP_FAT | positive regulation of transcription | 39 | 5.80E-06 |
| GOTERM_BP_FAT | positive regulation of transcription, DNA-dependent | 35 | 6.80E-06 |
| GOTERM_BP_FAT | positive regulation of macromolecule biosynthetic process | 43 | 8.60E-06 |
| GOTERM_MF_FAT | transcription factor activity | 43 | 1.20E-05 |
| GOTERM_BP_FAT | positive regulation of biosynthetic process | 44 | 2.20E-05 |
| GOTERM_BP_FAT | positive regulation of cellular biosynthetic process | 43 | 3.10E-05 |
| GOTERM_MF_FAT | DNA binding | 70 | 7.80E-05 |
| SP_PIR_KEYWORDS | dna-binding | 44 | 1.20E-04 |
| GOTERM_BP_FAT | regulation of transcription from RNA polymerase II promoter | 38 | 1.90E-04 |
| GOTERM_BP_FAT | positive regulation of transcription from RNA polymerase II promoter | 27 | 3.90E-04 |
| GOTERM_MF_FAT | sequence-specific DNA binding | 32 | 4.60E-04 |
| GOTERM_BP_FAT | regulation of RNA metabolic process | 62 | 1.10E-03 |
| GOTERM_BP_FAT | regulation of transcription, DNA-dependent | 59 | 2.60E-03 |
| SP_PIR_KEYWORDS | nucleus | 87 | 3.60E-03 |
| | | | |
| **Cluster 2** | **Enrichment Score:3.29** | **Count** | **P-value** |

| | | | |
|---|---|---|---|
| GOTERM_BP_FAT | regulation of cell development | 25 | 7.50E-07 |
| GOTERM_BP_FAT | regulation of neurogenesis | 22 | 4.40E-06 |
| GOTERM_BP_FAT | regulation of cell morphogenesis involved in differentiation | 14 | 1.30E-05 |
| GOTERM_BP_FAT | regulation of nervous system development | 22 | 2.00E-05 |
| GOTERM_BP_FAT | positive regulation of cell development | 13 | 2.90E-05 |
| GOTERM_BP_FAT | regulation of neuron projection development | 12 | 2.30E-04 |
| GOTERM_BP_FAT | positive regulation of neurogenesis | 11 | 2.50E-04 |
| GOTERM_BP_FAT | regulation of cell morphogenesis | 14 | 3.20E-04 |
| GOTERM_BP_FAT | regulation of cell projection organization | 13 | 3.30E-04 |
| GOTERM_BP_FAT | regulation of axonogenesis | 10 | 3.60E-04 |
| GOTERM_BP_FAT | regulation of neuron differentiation | 16 | 4.00E-04 |
| GOTERM_BP_FAT | positive regulation of cell differentiation | 20 | 6.60E-04 |
| GOTERM_BP_FAT | positive regulation of developmental process | 22 | 1.40E-03 |
| GOTERM_BP_FAT | negative regulation of cell projection organization | 6 | 6.80E-03 |
| GOTERM_BP_FAT | negative regulation of cellular component organization | 11 | 8.50E-03 |
| GOTERM_BP_FAT | negative regulation of neurogenesis | 7 | 9.00E-03 |
| GOTERM_BP_FAT | negative regulation of cell development | 7 | 1.10E-02 |
| GOTERM_BP_FAT | negative regulation of axonogenesis | 5 | 1.60E-02 |
| GOTERM_BP_FAT | positive regulation of axonogenesis | 5 | 2.00E-02 |
| GOTERM_BP_FAT | positive regulation of cellular component organization | 12 | 4.60E-02 |
| | | | |
| **Cluster 3** | **Enrichment Score:2.93** | **Count** | **P-value** |
| GOTERM_BP_FAT | neuron differentiation | 37 | 1.00E-06 |
| GOTERM_BP_FAT | neuron projection development | 23 | 9.60E-05 |
| GOTERM_BP_FAT | cell projection organization | 26 | 3.60E-04 |
| GOTERM_BP_FAT | cell morphogenesis involved in differentiation | 20 | 4.00E-04 |
| GOTERM_BP_FAT | neuron development | 25 | 4.80E-04 |
| GOTERM_BP_FAT | neuron projection morphogenesis | 18 | 7.30E-04 |
| GOTERM_BP_FAT | axonogenesis | 16 | 1.20E-03 |
| GOTERM_BP_FAT | cell projection morphogenesis | 18 | 2.20E-03 |

| | | | |
|---|---|---|---|
| GOTERM_BP_FAT | cell part morphogenesis | 18 | 3.40E-03 |
| GOTERM_BP_FAT | cell morphogenesis involved in neuron differentiation | 16 | 3.50E-03 |
| GOTERM_BP_FAT | cell morphogenesis | 22 | 4.10E-03 |
| GOTERM_BP_FAT | axon guidance | 10 | 7.60E-03 |
| GOTERM_BP_FAT | cellular component morphogenesis | 22 | 1.20E-02 |
| GOTERM_BP_FAT | cell motion | 19 | 1.40E-01 |
| | | | |
| **Cluster 4** | **Enrichment Score:2.89** | **Count** | **P-value** |
| GOTERM_BP_FAT | pattern specification process | 21 | 1.80E-04 |
| GOTERM_BP_FAT | regionalization | 17 | 5.00E-04 |
| GOTERM_BP_FAT | embryonic morphogenesis | 22 | 2.20E-03 |
| GOTERM_BP_FAT | anterior/posterior pattern formation | 11 | 1.40E-02 |
| | | | |
| **Cluster 5** | **Enrichment Score:2.42** | **Count** | **P-value** |
| GOTERM_BP_FAT | positive regulation of molecular function | 32 | 6.40E-04 |
| GOTERM_BP_FAT | regulation of phosphate metabolic process | 27 | 1.20E-03 |
| GOTERM_BP_FAT | regulation of phosphorus metabolic process | 27 | 1.20E-03 |
| GOTERM_BP_FAT | positive regulation of catalytic activity | 28 | 1.50E-03 |
| GOTERM_BP_FAT | regulation of protein kinase activity | 19 | 3.70E-03 |
| GOTERM_BP_FAT | positive regulation of protein kinase activity | 15 | 4.20E-03 |
| GOTERM_BP_FAT | regulation of transferase activity | 20 | 4.60E-03 |
| GOTERM_BP_FAT | positive regulation of kinase activity | 15 | 6.00E-03 |
| GOTERM_BP_FAT | regulation of kinase activity | 19 | 6.20E-03 |
| GOTERM_BP_FAT | regulation of phosphorylation | 24 | 6.50E-03 |
| GOTERM_BP_FAT | positive regulation of transferase activity | 15 | 8.90E-03 |
| GOTERM_BP_FAT | activation of protein kinase activity | 8 | 4.40E-02 |
| | | | |
| **Cluster 6** | **Enrichment Score:2.41** | **Count** | **P-value** |
| GOTERM_MF_FAT | protein kinase binding | 14 | 1.80E-03 |
| GOTERM_MF_FAT | enzyme binding | 28 | 5.50E-03 |

| | | | |
|---|---|---|---|
| GOTERM_MF_FAT | kinase binding | 14 | 5.90E-03 |
| | | | |
| **Cluster 7** | **Enrichment Score:2.28** | **Count** | **P-value** |
| GOTERM_CC_FAT | nucleoplasm | 39 | 1.10E-03 |
| GOTERM_CC_FAT | nucleoplasm part | 27 | 1.70E-03 |
| GOTERM_CC_FAT | nuclear lumen | 46 | 4.00E-03 |
| GOTERM_CC_FAT | transcription factor complex | 14 | 8.30E-03 |
| GOTERM_CC_FAT | organelle lumen | 55 | 1.10E-02 |
| GOTERM_CC_FAT | membrane-enclosed lumen | 56 | 1.20E-02 |
| GOTERM_CC_FAT | intracellular organelle lumen | 53 | 1.30E-02 |
| | | | |
| **Cluster 8** | **Enrichment Score:2.19** | **Count** | **P-value** |
| GOTERM_BP_FAT | mesenchymal cell development | 8 | 7.90E-04 |
| GOTERM_BP_FAT | mesenchymal cell differentiation | 8 | 9.00E-04 |
| GOTERM_BP_FAT | mesenchyme development | 8 | 1.00E-03 |
| GOTERM_BP_FAT | epithelial to mesenchymal transition | 5 | 1.20E-03 |
| GOTERM_BP_FAT | neural crest cell development | 3 | 2.80E-01 |
| GOTERM_BP_FAT | neural crest cell differentiation | 3 | 2.80E-01 |
| | | | |
| **Cluster 9** | **Enrichment Score:2.17** | **Count** | **P-value** |
| GOTERM_BP_FAT | tube development | 21 | 2.00E-04 |
| GOTERM_BP_FAT | tube morphogenesis | 15 | 5.80E-04 |
| GOTERM_BP_FAT | tissue morphogenesis | 17 | 3.40E-03 |
| GOTERM_BP_FAT | epithelium development | 16 | 1.50E-02 |
| GOTERM_BP_FAT | morphogenesis of an epithelium | 10 | 6.90E-02 |
| GOTERM_BP_FAT | epithelial tube morphogenesis | 6 | 2.30E-01 |
| | | | |
| **Cluster 10** | **Enrichment Score:2.15** | **Count** | **P-value** |
| GOTERM_BP_FAT | programmed cell death | 27 | 2.80E-04 |
| GOTERM_BP_FAT | apoptosis | 26 | 4.70E-04 |

| | | | |
|---|---|---|---|
| GOTERM_BP_FAT | death | 28 | 6.70E-04 |
| GOTERM_BP_FAT | cell death | 27 | 1.10E-03 |
| SP_PIR_KEYWORDS | Apoptosis | 14 | 2.00E-02 |
| GOTERM_BP_FAT | regulation of programmed cell death | 29 | 1.40E-01 |
| GOTERM_BP_FAT | regulation of cell death | 29 | 1.40E-01 |
| GOTERM_BP_FAT | regulation of apoptosis | 28 | 1.70E-01 |
| | | | |
| **Cluster 11** | **Enrichment Score:2.11** | **Count** | **P-value** |
| GOTERM_CC_FAT | anchored to membrane | 14 | 9.60E-05 |
| SP_PIR_KEYWORDS | gpi-anchor | 9 | 4.60E-03 |
| GOTERM_CC_FAT | anchored to plasma membrane | 5 | 5.00E-03 |
| SP_PIR_KEYWORDS | lipoprotein | 26 | 9.30E-03 |
| UP_SEQ_FEATURE | propeptide:Removed in mature form | 10 | 7.90E-02 |
| UP_SEQ_FEATURE | lipid moiety-binding region:GPI-anchor amidated serine | 4 | 1.30E-01 |
| | | | |
| **Cluster 12** | **Enrichment Score:2.04** | **Count** | **P-value** |
| KEGG_PATHWAY | Hedgehog signaling pathway | 8 | 1.40E-03 |
| KEGG_PATHWAY | Melanogenesis | 10 | 3.00E-03 |
| KEGG_PATHWAY | Wnt signaling pathway | 11 | 2.00E-02 |
| KEGG_PATHWAY | Basal cell carcinoma | 5 | 8.50E-02 |
| | | | |
| **Cluster 13** | **Enrichment Score:1.79** | **Count** | **P-value** |
| GOTERM_CC_FAT | neuron projection | 26 | 4.20E-03 |
| GOTERM_CC_FAT | cell projection | 38 | 4.20E-03 |
| GOTERM_CC_FAT | axon | 15 | 5.00E-03 |
| GOTERM_CC_FAT | cell soma | 15 | 3.70E-02 |
| GOTERM_CC_FAT | cell projection part | 15 | 3.80E-02 |
| GOTERM_CC_FAT | dendrite | 12 | 1.40E-01 |
| | | | |
| **Cluster 14** | **Enrichment Score:1.76** | **Count** | **P-value** |

| | | | |
|---|---|---|---|
| GOTERM_BP_FAT | tube development | 21 | 2.00E-04 |
| GOTERM_BP_FAT | gut development | 7 | 3.70E-03 |
| GOTERM_BP_FAT | lung development | 10 | 1.00E-02 |
| GOTERM_BP_FAT | respiratory tube development | 10 | 1.10E-02 |
| GOTERM_BP_FAT | respiratory system development | 10 | 1.50E-02 |
| GOTERM_BP_FAT | gland development | 14 | 1.50E-02 |
| GOTERM_BP_FAT | digestive system development | 5 | 1.80E-02 |
| GOTERM_BP_FAT | determination of bilateral symmetry | 5 | 2.90E-02 |
| GOTERM_BP_FAT | determination of symmetry | 5 | 2.90E-02 |
| GOTERM_BP_FAT | heart development | 12 | 9.30E-02 |
| GOTERM_BP_FAT | determination of left/right symmetry | 4 | 1.10E-01 |
| GOTERM_BP_FAT | gland morphogenesis | 5 | 2.90E-01 |
| | | | |
| **Cluster 15** | **Enrichment Score:1.73** | **Count** | **P-value** |
| GOTERM_MF_FAT | transcription factor binding | 20 | 5.90E-03 |
| GOTERM_MF_FAT | transcription coactivator activity | 8 | 2.60E-02 |
| GOTERM_MF_FAT | transcription cofactor activity | 11 | 4.20E-02 |
