# Supplementary material for: Interplay of miR-137 and EZH2 contributes to the genome-wide redistribution of H3K27me3 underlying the Pb-induced memory impairment
Source: Cell Death Dis. 2019 Sep 11;10(9):671. doi: 10.1038/s41419-019-1912-7 (PMC6739382; doi:10.1038/s41419-019-1912-7)
Supplement: Supplementary file 15 — Dataset 7 [file 41419_2019_1912_MOESM15_ESM.pdf]

Enriched GO clusters for differentially-regulated genes by H3K27me3

**Cluster 1**	**Enrichment Score: 9.81**	**Count**	**P Value**
GOTERM_BP_FAT	positive regulation of nucleobase, nucleoside, nucleotide and nucleic acid metabolic process	46	1.10E-12
GOTERM_BP_FAT	positive regulation of nitrogen compound metabolic process	46	3.00E-12
GOTERM_BP_FAT	positive regulation of cellular biosynthetic process	46	2.10E-11
GOTERM_BP_FAT	positive regulation of macromolecule metabolic process	52	2.50E-11
GOTERM_BP_FAT	positive regulation of biosynthetic process	46	3.80E-11
GOTERM_BP_FAT	positive regulation of macromolecule biosynthetic process	44	4.70E-11
GOTERM_BP_FAT	positive regulation of RNA metabolic process	37	5.40E-11
GOTERM_BP_FAT	positive regulation of gene expression	40	1.30E-10
GOTERM_BP_FAT	positive regulation of transcription, DNA-dependent	36	1.80E-10
GOTERM_BP_FAT	positive regulation of transcription	39	2.40E-10
GOTERM_BP_FAT	positive regulation of transcription from RNA polymerase II promoter	29	4.00E-08
GOTERM_BP_FAT	regulation of transcription from RNA polymerase II promoter	33	5.60E-06
**Cluster 2**	**Enrichment Score: 8.02**	**Count**	**P Value**
GOTERM_BP_FAT	regulation of transcription	73	3.60E-09
GOTERM_BP_FAT	regulation of RNA metabolic process	61	9.60E-09
GOTERM_BP_FAT	regulation of transcription, DNA-dependent	59	2.60E-08
**Cluster 3**	**Enrichment Score: 6.42**	**Count**	**P Value**
SP_PIR_KEYWORD	transcription regulation	40	7.00E-08
GOTERM_BP_FAT	transcription	38	5.90E-07
SP_PIR_KEYWORD	Transcription	39	1.40E-06
**Cluster 4**	**Enrichment Score: 4.63**	**Count**	**P Value**
SP_PIR_KEYWORD	Homeobox	15	5.40E-06
INTERPRO	Homeodomain-related	14	1.20E-05
INTERPRO	Homeobox	14	1.50E-05
INTERPRO	Homeobox, conserved site	12	5.20E-05

SMART	HOX	14	1.40E-04
**Cluster 5**	**Enrichment Score: 4.27**	**Count**	**P Value**
GOTERM_BP_FAT	regulation of cell motion	16	2.00E-05
GOTERM_BP_FAT	regulation of locomotion	15	8.90E-05
GOTERM_BP_FAT	regulation of cell migration	14	9.00E-05
**Cluster 6**	**Enrichment Score: 3.46**	**Count**	**P Value**
GOTERM_BP_FAT	positive regulation of cell motion	11	8.20E-05
GOTERM_BP_FAT	positive regulation of locomotion	10	4.80E-04
GOTERM_BP_FAT	positive regulation of cell migration	9	1.10E-03
**Cluster 7**	**Enrichment Score: 3.17**	**Count**	**P Value**
GOTERM_BP_FAT	mesenchymal cell development	7	6.10E-04
GOTERM_BP_FAT	mesenchymal cell differentiation	7	6.80E-04
GOTERM_BP_FAT	mesenchyme development	7	7.60E-04
**Cluster 8**	**Enrichment Score: 3.12**	**Count**	**P Value**
GOTERM_BP_FAT	regulation of apoptosis	30	6.40E-04
GOTERM_BP_FAT	regulation of programmed cell death	30	7.90E-04
GOTERM_BP_FAT	regulation of cell death	30	8.40E-04
**Cluster 9**	**Enrichment Score: 3.05**	**Count**	**P Value**
GOTERM_BP_FAT	response to steroid hormone stimulus	19	1.00E-04
GOTERM_BP_FAT	response to endogenous stimulus	25	2.50E-03
GOTERM_BP_FAT	response to hormone stimulus	23	2.60E-03
**Cluster 10**	**Enrichment Score: 3**	**Count**	**P Value**
GOTERM_BP_FAT	lung development	10	8.30E-04
GOTERM_BP_FAT	respiratory tube development	10	9.40E-04

GOTERM_BP_FAT	respiratory system development	10	1.30E-03
**Cluster 11**	**Enrichment Score: 2.79**	**Count**	**P Value**
GOTERM_BP_FAT	negative regulation of apoptosis	18	1.40E-03
GOTERM_BP_FAT	negative regulation of programmed cell death	18	1.70E-03
GOTERM_BP_FAT	negative regulation of cell death	18	1.70E-03
**Cluster 12**	**Enrichment Score: 2.66**	**Count**	**P Value**
GOTERM_BP_FAT	cell motion	21	1.20E-03
GOTERM_BP_FAT	cell migration	15	2.20E-03
GOTERM_BP_FAT	localization of cell	17	3.00E-03
GOTERM_BP_FAT	cell motility	17	3.00E-03
**Cluster 13**	**Enrichment Score: 2.55**	**Count**	**P Value**
GOTERM_BP_FAT	limb development	10	3.80E-04
GOTERM_BP_FAT	appendage development	10	3.80E-04
GOTERM_BP_FAT	appendage morphogenesis	8	5.50E-03
GOTERM_BP_FAT	limb morphogenesis	8	5.50E-03
GOTERM_BP_FAT	embryonic appendage morphogenesis	7	1.10E-02
GOTERM_BP_FAT	embryonic limb morphogenesis	7	1.10E-02
**Cluster 14**	**Enrichment Score: 2.47**	**Count**	**P Value**
GOTERM_BP_FAT	apoptosis	19	1.70E-03
GOTERM_BP_FAT	programmed cell death	19	2.10E-03
GOTERM_BP_FAT	cell death	19	5.70E-03
GOTERM_BP_FAT	death	19	6.80E-03
**Cluster 15**	**Enrichment Score: 2.43**	**Count**	**P Value**
GOTERM_BP_FAT	regulation of cardiac muscle tissue development	4	2.10E-03
GOTERM_BP_FAT	regulation of cardiac muscle growth	4	2.10E-03

GOTERM_BP_FAT	regulation of cardiac muscle cell proliferation	4	2.10E-03
GOTERM_BP_FAT	regulation of heart growth	4	2.70E-03
GOTERM_BP_FAT	negative regulation of cardiac muscle cell proliferation	3	2.90E-03
GOTERM_BP_FAT	regulation of organ growth	4	3.20E-02
**Cluster 16**	**Enrichment Score: 2.35**	**Count**	**P Value**
GOTERM_MF_FAT	channel activity	18	1.90E-03
GOTERM_MF_FAT	passive transmembrane transporter activity	18	1.90E-03
GOTERM_MF_FAT	ion channel activity	17	2.50E-03
GOTERM_MF_FAT	substrate specific channel activity	17	3.30E-03
SP_PIR_KEYWORDS	ionic channel	16	4.80E-03
GOTERM_MF_FAT	gated channel activity	13	1.30E-02
GOTERM_MF_FAT	cation channel activity	12	1.90E-02
**Cluster 17**	**Enrichment Score: 2.05**	**Count**	**P Value**
GOTERM_BP_FAT	antigen processing and presentation of peptide antigen via MHC class I	5	1.60E-03
GOTERM_BP_FAT	antigen processing and presentation of peptide antigen	5	1.10E-02
GOTERM_BP_FAT	antigen processing and presentation	6	3.90E-02
**Cluster 18**	**Enrichment Score: 2.04**	**Count**	**P Value**
GOTERM_BP_FAT	somite specification	3	1.50E-03
GOTERM_BP_FAT	segment specification	3	1.60E-02
GOTERM_BP_FAT	embryonic pattern specification	4	3.20E-02
**Cluster 19**	**Enrichment Score: 1.87**	**Count**	**P Value**
GOTERM_MF_FAT	voltage-gated ion channel activity	10	1.20E-02
GOTERM_MF_FAT	voltage-gated channel activity	10	1.20E-02
GOTERM_MF_FAT	gated channel activity	13	1.30E-02
SP_PIR_KEYWORDS	voltage-gated channel	9	1.80E-02

**Cluster 20**	**Enrichment Score: 1.82**	**Count**	**P Value**
GOTERM_BP_FAT	somitogenesis	5	6.10E-03
GOTERM_BP_FAT	segmentation	5	1.80E-02
GOTERM_BP_FAT	embryonic pattern specification	4	3.20E-02
